# Identification of Evening Complex Associated Proteins in *Arabidopsis* by Affinity Purification and Mass Spectrometry* [Fn FN2]

**DOI:** 10.1074/mcp.M115.054064

**Published:** 2015-11-06

**Authors:** He Huang, Sophie Alvarez, Rebecca Bindbeutel, Zhouxin Shen, Michael J. Naldrett, Bradley S. Evans, Steven P. Briggs, Leslie M. Hicks, Steve A. Kay, Dmitri A. Nusinow

**Affiliations:** From the ‡Donald Danforth Plant Science Center, 975 N. Warson Road, St. Louis, Missouri, 63132;; §University of California San Diego, Division of Biological Sciences, Cell and Developmental Biology Section, 9500 Gilman Drive, La Jolla, California 92093-0116;; ¶The University of North Carolina at Chapel Hill, Department of Chemistry, Chapel Hill, North Carolina 27599;; ‖University of Southern California, Molecular and Computational Biology Section, Los Angeles, California 90089

## Abstract

Many species possess an endogenous circadian clock to synchronize internal physiology with an oscillating external environment. In plants, the circadian clock coordinates growth, metabolism and development over daily and seasonal time scales. Many proteins in the circadian network form oscillating complexes that temporally regulate myriad processes, including signal transduction, transcription, protein degradation and post-translational modification. In *Arabidopsis thaliana,* a tripartite complex composed of EARLY FLOWERING 4 (ELF4), EARLY FLOWERING 3 (ELF3), and LUX ARRHYTHMO (LUX), named the evening complex, modulates daily rhythms in gene expression and growth through transcriptional regulation. However, little is known about the physical interactions that connect the circadian system to other pathways. We used affinity purification and mass spectrometry (AP-MS) methods to identify proteins that associate with the evening complex in *A. thaliana.* New connections within the circadian network as well as to light signaling pathways were identified, including linkages between the evening complex, TIMING OF CAB EXPRESSION1 (TOC1), TIME FOR COFFEE (TIC), all phytochromes and TANDEM ZINC KNUCKLE/PLUS3 (TZP). Coupling genetic mutation with affinity purifications tested the roles of phytochrome B (phyB), EARLY FLOWERING 4, and EARLY FLOWERING 3 as nodes connecting the evening complex to clock and light signaling pathways. These experiments establish a hierarchical association between pathways and indicate direct and indirect interactions. Specifically, the results suggested that EARLY FLOWERING 3 and phytochrome B act as hubs connecting the clock and red light signaling pathways. Finally, we characterized a clade of associated nuclear kinases that regulate circadian rhythms, growth, and flowering in *A. thaliana*. Coupling mass spectrometry and genetics is a powerful method to rapidly and directly identify novel components and connections within and between complex signaling pathways.

Each day the rotation of the Earth generates a dynamic environment that oscillates with a period of twenty-four hours. The selective advantage in anticipating these regular light/dark and hot/cold intervals has led to the presence of endogenous circadian oscillators in most terrestrial organisms (1–5). Circadian clocks provide a mechanism by which an organism can optimize cellular physiology and coordinate development with daily and seasonal changes, resulting in a measurable adaptive advantage (6–9). The clock regulates many of the cellular and physiological networks in plants, including photosynthesis, disease resistance, phytohormone, starch metabolism, growth, and photoperiodism pathways (10–14). Clocks are synchronized to the local environment through the perception of daily environmental oscillations in light and temperature (3). Thus, defining the mechanisms that connect the clock to environmental sensory systems is key to understanding the daily control of physiological outputs, such as growth and flowering.

The general architecture of the circadian clock is common in all higher eukaryotes, where multiple interlocking transcriptional feedback loops are coupled with post-transcriptional regulatory mechanisms to generate a robust 24-hour oscillator (2, 4, 15, 16). In plants, the circadian clock is currently described as containing a multiple feedback loop structure regulating specific transcriptional programs in the morning, afternoon and evening (17). The morning loop is composed of the MYB-domain containing transcription factors *CIRCADIAN CLOCK ASSOCIATED 1 (CCA1*) and *LATE ELONGATED HYPOCOTYL (LHY*), which suppress the expression of *PSEUDO-RESPONSE REGULATOR 9* and *7 (PRR9 and PRR7*) (18–21). *PRR9, PRR7,* and *PRR5* feedback and repress the expression of *LHY* and *CCA1* during the day (22–25). In the evening, REVEILLE8 positively regulates the expression of evening-expressed clock genes, *TIMING OF CAB EXPRESSION1 (TOC1,* also known as *PRR1*), *PRR5, EARLY FLOWERING 4 (ELF4),* and the GARP transcription factor *LUX ARRHYTHMO (LUX,* also known as *PHYTOCLOCK 1*) (26). TOC1, CCA1, and LHY reciprocally regulate their expression (27–30). In addition, a multi-protein complex comprised of the proteins encoded by *EARLY FLOWERING 3 (ELF3), ELF4,* and *LUX,* participates in clock regulation (31–43). ELF3 binds directly to both ELF4 and LUX to form a nuclear-localized complex, named the evening complex (EC), whose levels peak at dusk (34, 41, 42). The EC functions as a transcriptional regulator, repressing clock and growth-associated transcription factors to regulate circadian rhythms and hypocotyl elongation (41–45).

In plants, multiple photoreceptors participate in light perception into the circadian clock. Among them are the blue light sensing cryptochromes (*CRY1* and *CRY2*), the red/far-red absorbing phytochromes (*PHYA, PHYB, PHYC, PHYD,* and *PHYE*) (46–50), and a family of three Light, Oxygen and Voltage domain (LOV) containing F-Box proteins, including *ZEITLUPE (ZTL), FLAVIN BINDING, KELCH REPEAT, F-BOX1 (FKF1*), and *LOV KELCH PROTEIN2 (LKP2*) (51–54). Signaling through the photoreceptors is thought to regulate the entrainment and period of rhythms. However, the precise mechanism of light input into the clock is poorly understood. This is due, in part, to redundancy within the photoreceptor families and the complexity of interactions among the light signaling components. The phytochrome family members phyB, phyC, phyD, and phyE can form heterodimers, each with potentially unique photoactive properties (55, 56). In addition, phyA, phyB, phyC, phyD, and phyE each directly bind to CONSTITUTIVE PHOTOMORPHOGENIC 1 (COP1), an E3-ligase that regulates light signaling and circadian clock pathways through protein ubiquitination and degradation (57–63). COP1 forms a complex with the SUPRESSOR OF PHYA-105 family (SPA1–4) which modulate COP1 activity to regulate photomorphogenesis and development (64–66). phyB also binds to SPA1 to indirectly regulate COP1 activity in far-red light conditions (67). Defining the proteins and complexes that associate with clock factors is critical to understanding how light signaling is connected to the circadian network.

ELF3 is linked to light-signaling pathways through phyB and COP1 (34, 61). COP1 and ELF3 together regulate the daily turnover of GIGANTEA (GI), before COP1 directs the degradation of ELF3 in the late evening under short days to regulate flowering and circadian rhythms (61). Loss-of-function alleles in phyB, ELF3, and ELF4 cause similar early flowering, long hypocotyl, and leaf growth phenotypes (34, 35, 68, 69) and the over-expression of ELF3 suppresses the flowering and hypocotyl growth phenotypes of *phyB* mutants, suggesting that ELF3 can directly regulate red light-signaling pathways (34, 70). In addition, the expression of red-light responsive genes is affected in *elf3* mutants, and misexpression of ELF3 or *elf3* mutants causes altered sensitivity to red-light (32, 46, 70–72). Although direct interactions between ELF3 and phyB have been described using yeast two-hybrid assays and *in vitro*, *in vivo* interactions between ELF3 and phyB have not been reported. In addition, whether ELF3 can associate with other phytochrome proteins, either directly or indirectly, is unknown.

To determine how clock components are integrated with cellular pathways, affinity purification and mass spectrometry (AP-MS)^1^ were used to identify proteins that associate with the evening complex, which is a critical regulator of clock, growth, light and flowering pathways. Tandem AP methods have been used to identify protein complexes in diverse organisms (73–77). The 6xHis-3xFlag epitope was chosen as it has been successfully used previously for protein purification and mass spectrometry identification in other systems, including plants (76–78). This tag was introduced into a set of Gateway-cloning compatible vectors for constitutive expression in plants and we developed a selective, reproducible, and rapid purification protocol (∼ 6 h from tissue to completed affinity capture steps) (45, 79, 80). We sought to apply this methodology to identify the protein partners of the evening complex components ELF4 and ELF3 (35). ELF4 is a small (15 kDa) nuclear-localized protein with a single conserved domain of unknown function (DUF-1313) (35, 38, 69, 81, 82). ELF4 regulates the subcellular localization of the evening complex through a direct association with ELF3. ELF3 is a 69 kDa nuclear localized protein without any named domains that is thought to act as a scaffold protein mediating interactions between clock components (ELF4, LUX, NOX and GI) and light signaling components (COP1, phyB and PHYTOCHROME INTERACTING FACTOR 4 (PIF4)) (34, 41, 42, 61, 70). Epitope-tagged ELF4 and ELF3 were expressed from native promoters in mutant backgrounds in *A. thaliana*, and used as bait for AP-MS. New connections between the evening complex and clock proteins TIME FOR COFFEE (TIC), TOC1, and LIGHT-REGULATED WD 1 (LWD1) were identified. ELF4 and ELF3 also coprecipitated many proteins in light-signaling pathways, including phyA-E, COP1-SPA complex, TANDEM ZINC KNUCKLE/PLUS3 (TZP), and PIF7. Multiple new associated proteins were also identified, including a clade of nuclear kinases, an F-box domain containing protein, FT-interacting protein, a transposase that is required for development, proteases, germin proteins and a scaffold protein, RACK1. Through AP-MS in mutant backgrounds, we established a link between the evening complex and light signaling pathways mediated through the interaction between ELF3 and phyB. Finally, we characterize the function of a clade of nuclear kinases and found that they have roles in hypocotyl elongation, circadian rhythms, and flowering.

## EXPERIMENTAL PROCEDURES

### 

#### 

##### Plant Materials

CCA1::LUC, CAB::LUC, *elf4–2, elf4–3, elf3–1, elf3–2* and *phyB-9* plants (41, 68, 80) are in the Columbia background and have been described previously. SALK_017102 (AT2G25670), SALK_064333 (AT3G03940), and SALK_002211 (AT5G18190) were obtained from the Arabidopsis Biological Resource Center whereas GABI_756G08 was obtained from the Nottingham Arabidopsis Stock Centre, all in the Colombia background (83). All genotypes used were generated by crossing and desired plant lines were identified by screening for luciferase, drug resistance, mutant phenotypes, PCR for T-DNA insertion or mutant alleles by CAPS as described (41). Seeds were gas sterilized and plated on 1/2× Murashige and Skoog basal salt medium with 0.8% agar ± 3% (w/v) sucrose (as noted). After stratification for 3 days, plates were transferred to a Percival incubator (Percival-Scientific, Perry, IA) set to a constant temperature of 22 °C. Light entrainment was 12 h light/12 h dark (LD) cycles, with light supplied at 80 μmol/m^2^/s.

##### Construction of HIS_6_–Flag_3_—Constructs

A His_6_-Flag_3_ containing an intervening Tobacco Etch Mosaic Virus cleavage site (sequence 5′-GGAAGAGGATCGCATCACCATCACCATCACGATTATGATATTCCAACTACTGCTAGCGAGAATTTGTATTTTCAGGGTGAGCTCGACTACAAAGACCATGACGGTGATTATAAAGATCATGACATCGACTACAAGGATGACGATGACAAGTAG-3′) was amplified from plasmid pJL212 (gift from Nevan Krogan, UCSF) using primers DN384 (5′-GGGCCTAGGGTCCGGAAGAGGATCGCATCAC-3′) and DN385 (5′-CCCCCTAGGCTACTTGTCATCGTC-3′), which also add AvrII sites. The amplified fragment was cloned into pCR-BluntII TOPO (Invitrogen, Carlsbad, CA), sequenced for verification, digested with AvrII, and cloned into pB7WG2-AvrII digested with AvrII (NEB, Ipswich, MA) to generate pB7HFC (45, 79). To generate pK7HFC, pB7HFC, and pK7WG2 were digested with EcoRI (NEB) and XbaI (NEB), and the fragment containing the His_6_-Flag_3_ sequences from pB7HFC was swapped into pK7WG2 (79). The integrity of all plasmids was verified by sequencing before use.

##### Cloning GFP, ELF4, and ELF3, Sequences into His_6_-Flag_3_vectors

GFP (GFP-F 5′-CACCATGGTGAGCAAGGGCGAGGAGCT-3′, GFP-R-NS 5′-CTTGTACAGCTCGTCCATGCCGAGA-3′), ELF4 (ELF4-F 5′-CACCATGAAGAGGAACGGCGAGACGA-3′, ELF4-R-NS 5′-AGCTCTAGTTCCGGCAGCACC-3′), and ELF3 (ELF3-F 5′-CACCATGAAGAGAGGGAAAGAT-3′, ELF3-R-NS 5′-AGGCTTAGAGGAGTCATAGCG-3′) were amplified to generate pENTR-dTOPO clones without stop codons (41). GFP and ELF4 were cloned by gateway into pB7HFC, and ELF3 was cloned by gateway into pK7HFC, to generate pB7HFC-GFP, pB7HFC-ELF4, and pK7HFC-ELF3, respectively. To generate *ELF4* driven from its own promoter sequences (84), we amplified 1580 bp of the promoter from the end of the 3′UTR of the adjacent gene to the last codon of ELF4 from genomic DNA, using the following primers, ELF4-Promoter-PMEI 5′-GGGTTTAAACTCATGATTTCCTGCGGTAATTATCTT-3′ and ELF4-no stop-reverse 5′-AGCTCTAGTTCCGGCAGCACC-3′. Both pB7HFC-ELF4 and the amplified fragment were digested with PmeI and SpeI, and the ELF4-promoter containing fragment replaced the Cauliflower Mosaic Virus 35S (CaMV35S) promoter-ELF4 fragment, generating pB7HFC-ELF4::ELF4. The resulting construct contains both promoter sequences and the 5′UTR of ELF4. To generate a promoter driven version of ELF3, we amplified 2135 bp from ELF3 promoter that includes 1650 bp of promoter sequence and the 5′ UTR from genomic DNA using the following primers (ELF3p-PmeI-5′-GTTTAAACCCAAAAATTCGCAATCTCCTTTA-3′ and ELF3p-R-SpeI-5′-ACTAGTCACTCACAATTCACAACCTTTTTC-3′). pK7HFC-ELF3 and the amplified fragment were digested with PmeI and SpeI, and the ELF3-promoter containing fragment replaced the Cauliflower Mosaic Virus 35S promoter-fragment, generating pK7HFC-ELF3::ELF3. All vectors were sequenced prior to use to verify integrity. pB7HFC-GFP, pB7HFC-ELF4::ELF4, and pK7HFC-ELF3::ELF3 were transformed into the *CCA1::LUC*, *elf4–3 CAB::LUC,* and *elf3–2 CCA1::LUC* background, respectively, using the floral dip method (85). Transformants were selected based on drug resistance and/or rescue of hypocotyl elongation and circadian reporter phenotype (41).

##### Cloning of the MLKs and H2B-GFP

MLK4 (AT3G13670) was cloned into pENTR-dTOPO using the following primers, 5′-CACCATGCCGGAGCTTCGCCGTGGAGTCCGC-3′ and 5′-AGATACAGTTCGGCCATAGCTTAACG-3′. MLK3 (AT2G26570) and MLK2 (AT3G03940) were cloned into pENTR-dTOPO using 5′-CACCATGCCTGAGCTGCGTAGCAACGCACG-3′, 5′-TGACACAGTTCGACCATAACAAATC-3′, 5′-CACCATGCCAGAGTTAAGAAGTGGAG-3′, and 5′-GCAAACTGTCCGACCATAGCATATTG-3′, respectively. H2B (AT2G28720) was cloned similarly using 5′-CACCATGGCACCAAAAGCCGGAAAG-3′ and 5′-AGAGCTAGTAAACTTAGTAACAGCC-3′. All pENTR clones were verified by sequence, then recombined with pK7FWG2 to generate C-terminal GFP-tagged versions of all the proteins (79).

##### N. benthamiana Transient Transformation and Confocal Microscopy

Overnight saturated cultures of *Agrobacterium tumefaciens* strain GV3101 carrying either MLK-GFP constructs or H2B-GFP were diluted in 10 mm MgCl_2_ (OD600 = 0.8) and kept at room temperature for 1∼2 h. An Agrobacterium culture of 35*S:P19-HA* was also diluted into the same concentration and mixed (at a ratio of 1:1) with each culture of GFP-fusions to suppress gene silencing (86). The cultures were then spot-infiltrated into 4 to 5-week-old *Nicotiana benthamiana* from the abaxial side of leaves. After 48 h, infected leaves were cut into small square pieces, mounted in water and used for confocal microscopy.

Confocal microscopy was performed with a Leica TCS SP8 confocal laser scanning microscope and an HC PL APO CS2 40x/1.10 WATER objective lens (Leica Microsystems, Mannheim, Germany). Light source is provided by the White Light Laser (WLL, power set as 70%). GFP fluorescence was monitored by a 498–600 nm band emission and a 488 nm excitation line of an Ar laser, with 10% transmission value for GFP/H2B controls and 20% transmission value for three kinases. Line average was set as 16 to reduce noise and frame accumulation was set as 2.

##### Luciferase Imaging

After 6 days of entrainment, the plants were sprayed with 5 mm luciferin (Goldbio, Olivette, MO) prepared in 0.01% (v/v) Triton X-100 (Sigma-Aldrich, St. Louis, MO) and transferred to constant light (70 μmol/m^2^/s, wavelengths 400, 430, 450, 530, 630, and 660 at intensity 350, or 20 μmol/m^2^/s 660 nm light (Heliospectra LED lights, Göteborg, Sweden)). The emitted luminescence was recorded over 5 days using a Pixis 1024B CCD camera (Princeton Instruments, Trenton, NJ) driven using Micro-Manager software (87, 88) after a 120–180s delay to diminish delayed fluorescence (89). The images were processed using Metamorph imaging software (Molecular Devices, Sunnyvale, CA), and the data were analyzed by fast Fourier transformed nonlinear least squares (FFT-NLLS) (90) using the Biological Rhythms Analysis Software System 3.0 (BRASS) available at http://www.amillar.org.

##### Hypocotyl Elongation Measurements

Seedlings were grown at 22°C under 12 h day and 12 h night conditions for 7 days before measuring hypocotyl length. For red light experiments *mlk* and *phyB-9* seedlings were grown at 22 °C under either constant red light (660 nm LED light at 25 μmol/m^2^/s (CLF Climatics, Wertingen, Germany)), or in the dark for 4 days. Seedlings were then arrayed, photographed with a ruler, and hypocotyl length was analyzed using NIH ImageJ.

##### Flowering Time Measurement

Seeds were liquid sterilized and placed on 0.5× MS +3% sucrose plates, then stratified for 3 days at 4 °C before being grown under 16 h long day conditions for 10 days in a chamber set at 22 °C, and 100 μmol/m^2^/s light intensity. Seedlings were then transplanted to Pro-Mix FPX soil and grown under the same conditions (except the chamber is at 200 μmol/m^2^/s). The flowering date and basal rosette leaf number of each plant was recorded when the first inflorescence was 1 cm long.

##### Yeast Two-Hybrid Analysis

We used the Matchmaker™ GAL4 Two-Hybrid systems (Clontech, Mountain View, CA) to analyze protein-protein interactions in yeast. Full length cDNAs of TIC (TICpF- 5′-CACCATGGATAGAAATAGAGAAGCTAGAAGAGT-3 and 5′-CTCACCCGCCTGTTGCTTCTGATCCACGGGTTTGACCTGAACCGCAGAGGAAA-3′), TOC1 and TOC1 fragments, described previously (29), were cloned into the pENTR/d-TOPO vector (Invitrogen) for sequencing. Verified cDNA sequences were cloned into either the pAS2-GW or pACT2-GW vector, which are derived from the pAS2–1 and pACT2 plasmids (Clontech) (41), through Gateway LR recombination reactions (Invitrogen). Both the DNA binding domain (DBD) or activating domain (AD)-fused constructs were transformed into *Saccharomyces cerevisiae* strain Y187 (*MAT*α) and the AH109 (*MATa*), respectively, by the Li-Ac transformation protocol according to the yeast handbook (Clontech). The yeast mating and two hybrid procedures were described previously with modifications (91). Two yeast strains of the same optical density (OD_600_) were mixed and incubated in low pH YCM liquid media (1% yeast extract, 1% bactopeptone, 2% dextrose, pH 4.5) for 4.5 h at 30 °C. Afterward, cells were pelleted, washed with ddH_2_O and incubated in regular YPDA liquid media and incubated overnight at 30 °C. Diploid yeast cells were then grown in the CSM -Leu -Trp liquid culture (Sunrise Science, San Diego, CA) supplemented with extra Adenine (with final concentration of 30 mg/L) for selection. After yeast grow to saturation, they were pelleted and washed with ddH_2_O before spotting on both CSM -Leu -Trp -His plates supplemented with 2 mm 3-Amino-1,2,4-triazole (3AT) and CSM -Leu -Trp plates (both plates have 30 mg/L Adenine). Pictures were taken after 4 days of incubation at 30 °C.

##### Experimental Design and Statistical rationale

Four independent biological replicate affinity purifications were performed for the control GFP-HFC and ELF4-HFC purifications. Three independent biological replications were performed from the ELF4-HFC *phyB-9* and ELF4-HFC *elf3–1* purifications. The ELF3-HFC affinity purifications in all genetic backgrounds were performed on two biologically independent samples. The number of biological replicates was chosen to determine reproducibility of identifications from purifications. For bioluminescence reporter assays, 8 ≤ *n* ≤16 seedlings were measured (see figure legends for *n*). For hypocotyl elongation assays, 20 seedlings were measured for each condition and genotype. For flowering measurements, 12 individual plants were potted in a random arrangement to minimize for position effects. All biological assays were performed at least twice in independent experiments to ensure reproducibility of results. All graphs have the mean and error bars reflecting 95% confidence intervals to illustrate the range of observations. Statistical analysis was performed using one-way ANOVA followed by a Bonferroni's test for multiple corrections using GraphPad Prism version 6.00 (GraphPad Software, La Jolla, CA, www.graphpad.com).

##### Tandem Affinity Purification From Plant Tissue

Seedlings were grown on sterilized qualitative filter paper (Whatman, Maidstone, UK) for 10–12 days, at 22 °C in 12:12 light/dark conditions and were harvested at ZT12. 5 g of whole seedlings were harvested and immediately frozen in liquid N_2_ and stored at −80 °C. The seedlings were transferred into a liquid N_2_ chilled 35 ml ball mill and disrupted in a reciprocal mixer mill (30 hz, 45 s, repeated 4 times (Retsch, Haan, Germany)) under liquid nitrogen. Ground tissue was gently resuspended in 12 ml (∼1 packed tissue volume) of SII buffer (100 mm sodium phosphate, pH 8.0, 150 mm NaCl, 5 mm EDTA, 5 mm EGTA, 0.1% Triton X-100, 1 mm PMSF, 1× protease inhibitor mixture (Roche, Basel, Switzerland), 1× Phosphatase Inhibitors II & III (Sigma-Aldrich), and 5 μm MG132 (Peptides International, Louisville, KY)) and sonicated twice at 50% power, 1 s on/off cycles for 20 s total on ice (Fisher Scientific model FB505, with microtip probe). Extracts were first clarified by centrifugation twice at 4 °C for 10 min at ≥20,000 × *g*, then by 0.45 μm filtration. 42 μg of anti-FLAG antibody (F1804, Sigma-Aldrich) crosslinked to protein G coated magnetic beads (Life Technologies, Grand Island, NY) (41) was incubated with the extract for 75 min at 4 °C with rotation. The beads were then washed twice with 10 ml of SII buffer, transferred to low protein binding 1.5 ml centrifuge tubes, and washed three more times with 900 μl of FTH buffer (100 mm sodium phosphate, pH 8.0, 150 mm NaCl, 0.1% Triton X-100). Captured proteins were eluted twice at 4 °C and 30 °C using 400 μl of 500 μg/ml 3xFLAG peptide (Sigma-Aldrich) diluted in FTH buffer. Eluates were combined and depleted using 70 μl Talon magnetic beads (Life Technologies) at 4 °C for 15 min. Talon resin was washed three times with 900 μl FTH buffer, then three times in 900 μl of 25 mm ammonium bicarbonate before all liquid was withdrawn and the samples were stored at −80 °C.

##### Silver Staining

A 4–15% precast SDS-PAGE gel (BioRad, Carlsbad, CA) was rinsed in water, and fixed for ≥ 30 min in a 50% EtOH 10% acetic acid solution, then treated with 30% EtOH for 15 min. ddH_2_O rinsed gels were sensitized with 0.2 g/L sodium thiosulphate for 90 s, then rinsed with ddH_2_O thrice. The sensitized gel was incubated with 2 g/L Silver Nitrate for 25 min, washed twice with ddH_2_O then developed in a solution of 60 g/L sodium carbonate, 20 ml/L of 0.2 g/L sodium thiosulphate solution and 500 μl/L 30% formaldehyde. A 6% acetic acid solution was used to halt development, and the blot was rinsed in ddH_2_O before scanning. This protocol was adapted from (92, 93).

##### Protein Digestion and Identification Using Liquid Chromatography-Tandem Mass Spectrometry (LC-MS/MS)

The proteins binding to the magnetic beads were reduced with 10 mm TCEP in 50 mm ammonium bicarbonate for 1 h at 37 °C, and then alkylated with 20 mm iodoacetamide for 30 min in the dark. Protein samples were then digested with 1 μg of trypsin at 37 °C overnight. After acidification of the samples with formic acid, the supernatant was transferred to a new tube and dried down. The digest was dissolved in 5% ACN/0.1% formic acid and 5 μl were injected for LC-MS/MS analysis.

LC-MS/MS was carried out on an LTQ-Orbitrap Velos Pro (ThermoFisher Scientific, Waltham, MA) as previously described (94, 95) coupled with a U3000 RSLCnano HPLC (ThermoFisher Scientific). The protein digests were first loaded onto a C_18_ trap column (PepMap100, 300 μm ID × 5 mm, 5 μm particle size, 100 Å; ThermoFisher Scientific) at a flow rate of 5 μl/min for 4 min equilibrated with 2% acetonitrile, 0.1% formic acid. Peptide separation was carried out on a C_18_ column (Acclaim PepMap RSLC, 15 cm × 75 μm nanoViper™, C18, 2 μm, 100 Å, ThermoFisher Scientific) at a flow rate of 0.26 μl/min and the following gradient: Time = 0–4 min, 2% B isocratic; 4–8 min, 2–10% B; 8–83 min, 10–25% B; 83–97 min, 25–50% B; 97–105 min, 50–98%. Mobile phase consisted of A, 0.1% formic acid; mobile phase B, 0.1% formic acid in 80:20 acetonitrile/water.

The LTQ-Orbitrap mass analyzer was operated in positive ESI mode using collision induced dissociation (CID) to fragment the HPLC separated peptides. The mass range for the MS survey scan done using the FTMS was 300 to 2000 *m*/*z* with resolving power set to 60,000 @ 400 *m*/*z* and the automatic gain control (AGC) target set to 1,000,000 ions with a maximum fill time of 10 ms. The 20 most intense signals in the survey scans were selected and fragmented in the ion trap using an isolation window of 1.5 *m*/*z*, an AGC target value of 10,000 ions, a maximum fill time of 100 ms, a normalized collision energy of 35 and activation time of 30 ms. Dynamic exclusion was performed with a repeat count of 1, exclusion duration of 60 s, and a minimum MS ion count for triggering MS/MS set to 5000 counts.

##### AP-MS Data Analysis

MS data were extracted by Proteome Discoverer (ThermoFisher Scientific; v.1.4) and converted into mgf. Database searches were done using Mascot (Matrix Science, London, UK; v.2.5.0) using the TAIR10 database (20101214, 35,386 entries) and the cRAP database (http://www.thegpm.org/cRAP/) and assuming the digestion enzyme trypsin and 2 missed cleavages. Mascot was searched with a fragment ion mass tolerance of 0.80 Da and a parent ion tolerance of 15 ppm. Deamidation of asparagine and glutamine, oxidation of methionine and carbamidomethyl of cysteine were specified in Mascot as variable modifications. Scaffold (Proteome Software Inc., Portland, OR; v.4.4.3) was used to validate MS/MS based peptide and protein identifications. Peptide identifications were accepted if they could be established at greater than 95.0% probability by the Peptide Prophet algorithm (96) with Scaffold delta-mass correction. The Scaffold Local FDR was used and only peptides probabilities with FDR<1% were used for further analysis. Protein identifications were accepted if they could be established at greater than 99.0% probability as assigned by the Protein Prophet algorithm (97). Proteins that contained similar peptides and could not be differentiated based on MS/MS analysis alone were grouped to satisfy the principles of parsimony. Proteins sharing significant peptide evidence were grouped into clusters. Only the proteins identified with ≥ 2 unique peptides were further used in the analysis, except when proteins with only one peptide were identified in more than one replicate. The unique Normalized Spectral Abundance Factor (uNSAF) (98) was selected for the quantitative measurement (Scaffold_4.4.3) to estimate the protein abundance of individual proteins in samples. Total unique peptide count refers to all uniquely identified peptides (including modified peptides and those shared with other proteins), whereas exclusive unique peptide counts refers to peptides that are derived from and can be assigned to only a single protein. SAINT analysis (Express Version: exp3.1) was run using default settings with samples kept separate, applying the following filters (unique peptides > 2, protein length included in model, and proteins collapsed to gene ID).

## RESULTS

### 

#### 

##### Characterization of the ELF4-HFC Lines

**Fig. 1.**
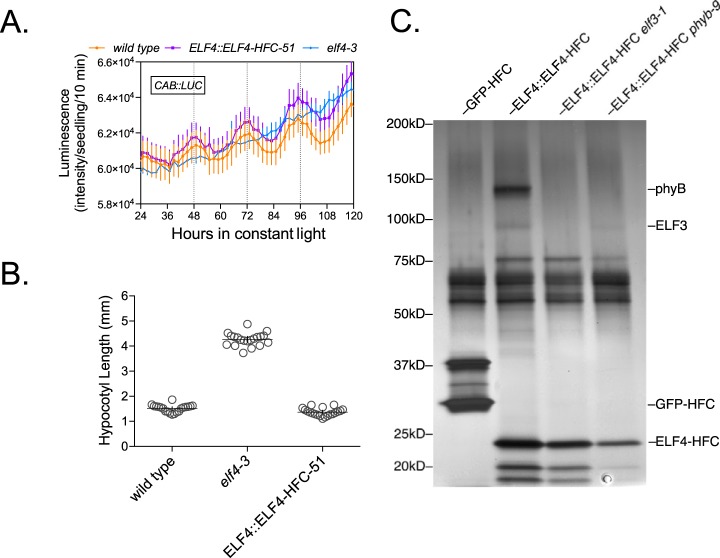
**Characterization of ELF4::ELF4-HFC rescue lines.** *A*, ELF4::ELF4-HFC rescues the arrhythmic circadian phenotype of *elf4–3*. Seedlings were entrained in 12 h light and 12 h dark conditions for 6 days before transferring to constant light. *n* = 16, error bars = 95% confidence interval. Measurements were repeated three times with similar results. *B*, ELF4::ELF4-HFC rescues the increased hypocotyl elongation phenotype of *elf4–3. n* = 20, error bars = 95% confidence interval. *C*, Silver stain of GFP-HFC and ELF4-HFC purifications in multiple backgrounds. The approximate location of the proteins is listed on the right and is based on the gain or loss of bands in the different genetic backgrounds.

A 6xHis-3xFlag-epitope tagged ELF4 was introduced into an *elf4* mutant background allowing selection of lines that complement circadian and growth defects caused by the *elf4* loss-of-function mutation (41). To avoid a long circadian period phenotype that occurs with constitutive over-expression of ELF4 (69), the *CAULIFLOWER MOSAIC VIRUS 35S* (CaMV35S) promoter was replaced with sequences from the endogenous promoter, including 5′ untranslated region sequences (79). The ELF4 promoter driven ELF4 C-terminally tagged with 6xHis-3xFlag was (ELF4::ELF4-HFC) was introduced into a *elf4–3* mutant background that contained a *CHLOROPHYLL A/B* promoter driven *LUCIFERASE* (*CAB::LUC*) reporter, which allowed for the noninvasive monitoring of circadian rhythms (41). Three independent lines were tested for their ability to rescue previously reported arrhythmic circadian rhythm phenotypes (supplemental Fig. S1*A*–S1*C*). Three ELF4::ELF4-HFC plants were characterized that rescued the loss of rhythms observed in the *elf4–3* mutant background (*elf4–3* (1/16), wild type (14/16) ELF4::ELF4-HFC *elf4–3* lines 51, 71, and 72- each with (13/16) rhythmic seedlings) (35, 41). These lines were also tested for rescue of hypocotyl elongation defects under diel growth conditions (mean length- wild type 1.512 ± 0.1451 mm, ELF4::ELF4-HFC *elf4–3* lines 51, 71, and 72- 1.369 ± 0.1526 mm, 1.689 ± 0.1994 mm, and 1.945 ± 0.2372 mm, respectively, *versus elf4–3* 4.267 ± 0.2555 mm, error = standard deviation (S.D.)) (Fig. 1*B* and supplemental Fig. S1*D*) (41). Together, these observations indicated that the tagged ELF4::ELF4-HFC was functional and a single complementing line (line 51) was further evaluated with AP-MS (Fig. 1).

##### AP-MS of ELF4-HFC

ELF4-HFC proteins were purified from 5 g of tissue grown in 12 h light/12 h dark conditions and harvested at the peak of ELF4/evening complex levels (Zeitgeiber time 12, ZT12) (35, 41). Nonspecific proteins were monitored through control experiments using a constitutively expressed GFP-HFC. Tandem affinity purified samples on the final Talon resin were on-bead digested and the resulting peptides were analyzed by nanoLC-MS/MS. Four biologically independent purifications of both ELF4-HFC and GFP-HFC were used to identify proteins that copurify with ELF4 (Fig. 1*C* and Table I). ELF4 peptides were observed in GFP control purifications, however, this is likely because of carryover from prior runs (data not shown). To control for carryover, samples were randomized before LC-MS/MS runs.

**Table I:**
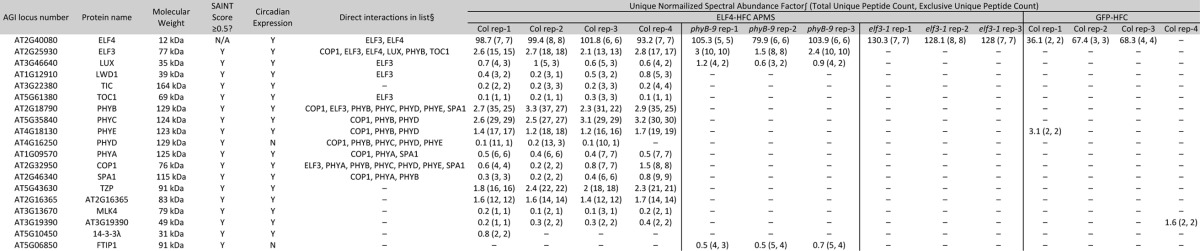
Identification of proteins associating with ELF4::ELF4-HFC #51 in elf4–3, elf4–3 phyB-9, and elf4–3 elf3–1 compared to GFP-HFC in wild type. The unique Normalized Spectral Abundance Factor uses only unique peptides to quantify protein abundance in samples (98). Total unique peptide count refers to all uniquely identified peptides (including those shared with other proteins within the same protein family), whereas exclusive unique peptide count refers to peptides that are derived from and can be assigned to a single protein. ∫ = uNSAF was multiplied by 100 for comparison. § = Direct interaction that can be assigned from either the literature (see main text for references), or this study (See Fig. 2). Circadian expression is derived from supplemental Fig. S2

To identify the protein partners that specifically associated with ELF4 compared with the control bait GFP, a significance analysis of interactome (SAINT) algorithm was employed to identify proteins that were statistically enriched (SAINT score ≥ 0.5) in the ELF4-HFC purifications compared with the negative control GFP purifications (99–101). This list was curated by removing frequently identified contaminant proteins (ribosomal, cytoskeleton, metabolic enzymes and photosynthetic machinery associated proteins) (102), leaving seventeen different proteins as significantly enriched in ELF4-HFC AP relative to GFP-HFC affinity purifications (Table I). These proteins include the other evening complex member ELF3 and LUX, clock proteins TIC, TOC1, and LWD1, photoreceptors phyA, phyB, phyC, phyD, phyE, and light signaling regulators COP1, SPA1, and TZP (103). Novel interactions with the MUT9-LIKE KINASE4 (MLK4 AT3G13670) (104), a granulin repeat family cysteine protease (AT3G19390) (105), 14–3-3 λ protein (106), and a gene annotated as an F-box family protein (AT2G16365) with roles in integrating light and plastid signaling during deetiolation (107) were also identified (Table I).

To further characterize the list of coprecipitated proteins, gene ontology (GO) category enrichment and circadian expression patterns under constant conditions were determined. GO analysis found that the list was enriched in genes associated with the regulation of circadian rhythms (GO:0007623, *p* value = 9.8e-13) and red or far-red light signaling pathways (GO:0010017, *p* value = 1.3e-11) (supplemental Table S1) (108, 109). Because the transcripts of the evening complex cycle (33, 35–37), the genes in the list were analyzed for rhythmic expression from a circadian dataset obtained under constant light and temperature conditions. Of the 17 genes identified, 16 are rhythmic with a 24 hour period expression pattern (adjusted *p* value ≤ 0.05) (supplemental Fig. S2) and 75% have a peak of expression between ZT 8 and 16, similar to the evening complex (110). The coprecipitating proteins are enriched for circadian expression patterns compared with the genome (Chi-square with Yates' correction, two tailed *p* value ≤ 0.0001, Table I). These results are consistent with the previously identified roles of the evening complex in the circadian oscillator network, and suggest that the evening complex is highly connected to red light signaling pathways.

##### AP-MS of ELF4-HFC in the phyB Mutant Background

As phyB was the major associated photoreceptor coprecipitating with ELF4, according to the calculated unique Normalized Spectral Abundance Factor (uNSAF) (98) and total unique peptide counts (Table I), we wanted to determine how phyB contributed to interactions among the identified proteins. Based on the reported interactions between phyB and the other proteins identified in Table I (34, 55, 62, 67, 111, 112), it appeared that phyB could play a critical role connecting the circadian and light pathways. The *phyB-9* mutation was introduced into the ELF4::ELF4-HFC #51 *elf4–3* background and ELF4-HFC was affinity purified from these backgrounds and associated proteins were identified by AP-MS (Fig. 1C and Table I). In this background, ELF4 could still reproducibly precipitate both ELF3 and LUX. However, association with other clock and light signaling pathway proteins, including all of the phytochromes, COP1, SPA1, TZP, LWD1, and TIC was lost. Also, ELF4-HFC now coprecipitated FLOWERING TIME-INTERACTING PROTEIN 1 (FTIP1), a protein that facilitates the transportation of the florigen protein FT, in the absence of phyB (113).

##### AP-MS of ELF4-HFC in the elf3 Mutant Background

ELF4 is a critical component of light and clock signaling pathways, but its function is not fully understood. The *elf3–1* allele was crossed into the ELF4::ELF4-HFC #51 *elf4–3* background to determine which proteins continue to associate with ELF4 in the absence of ELF3. This line showed loss of circadian rhythms (wild type (14/16), *elf4–3 elf3–1* (1/16), and ELF4::ELF4-HFC #51 *elf4–3 elf3–1* (1/16) rhythmic) and elongated hypocotyls (*elf4–3 elf3–1* was 5.385 ± 0.4199 mm and ELF4::ELF4-HFC *elf4–3 elf3–1* was 5.751 ± 0.4196 mm in length, error = S.D.), similar to previous results for *elf3* mutants (supplemental Figs. S3*A* and S3*C*) (31, 41). The protein identification of the affinity purifications was performed on ELF4::ELF4-HFC #51 *elf4–3 elf3–1* in biological triplicate (Fig. 1*C* and Table I). The proteins identified that specifically associated with ELF4 in the absence of ELF3 compared with GFP controls were common contaminants, suggesting that ELF4 mainly associates with and modulates ELF3 containing complexes.

##### AP-MS of ELF3-HFC Reveals Additional Associated Proteins Compared With ELF4

As ELF3 is described to act as a hub protein and may also regulate pathways outside of the evening complex, we hypothesized that affinity purification of ELF3 might identify additional interactions compared with those with ELF4 (41, 61, 70). To identify proteins that associate with ELF3, an ELF3 promoter driven ELF3-HFC (ELF3::ELF3-HFC) expression construct was assembled and transformed into *elf3–2* mutant background that contain the *CCA1::LUC* reporter. Multiple independent transgenic lines that rescued both circadian (wild type (8/8), *elf3–2* (2/8), and ELF3::ELF3-HFC #1, #3 and #15 each had (8/8) rhythmic seedlings) and hypocotyl growth defects (wild type = 1.768 ± 0.2205 mm, ELF3::ELF3-HFC #1 1.356 ± 0.2949 mm, ELF3::ELF3-HFC #3 1.554 ± 0.2313 mm, and ELF3::ELF3-HFC #15 1.667 ± 0.2579 mm, and *elf3–2* 4.630 ± 0.4138 mm in length, error = S.D.) were identified, suggesting that the fusion protein is functional (supplemental Fig. S4*A*–S4*D*). Using a representative line (ELF3::ELF3-HFC #3), ELF3 was purified to identify the associated proteins (Table II). The list of ELF3 coprecipitating proteins (both significantly enriched by SAINT analysis and absent from the list of proteins that precipitated with GFP-HFC) overlapped with most (17/19) of the proteins that coprecipitated with ELF4 (Table II), with six additional proteins identified specifically associating with ELF3. Similar to the ELF4 purifications, this list was enriched in circadian cycling genes (22/25, Table II and supplemental Fig. S2). GO term analysis also found significant enrichment for circadian rhythms (GO:0007623, *p* value = 2.6e-13) and red or far-red light signaling pathways (GO:0010017, *p* value = 2.5e-10) (supplemental Table S2) (108, 109). Notable proteins unique to the ELF3 purifications included the circadian-clock associated MYB domain transcription factor NOX (also known as BROTHER OF LUX ARRHYTHMO) (41, 45, 114), SPA2, SPA3, additional kinases from the MUT9-LIKE KINASE clade (MLKs) (104), the bHLH transcription factor PIF7 (115), GERMIN LIKE PROTEIN 3 (GER3) (116), and the hAT-like transposase DAYSLEEPER (117).

**Table II:**
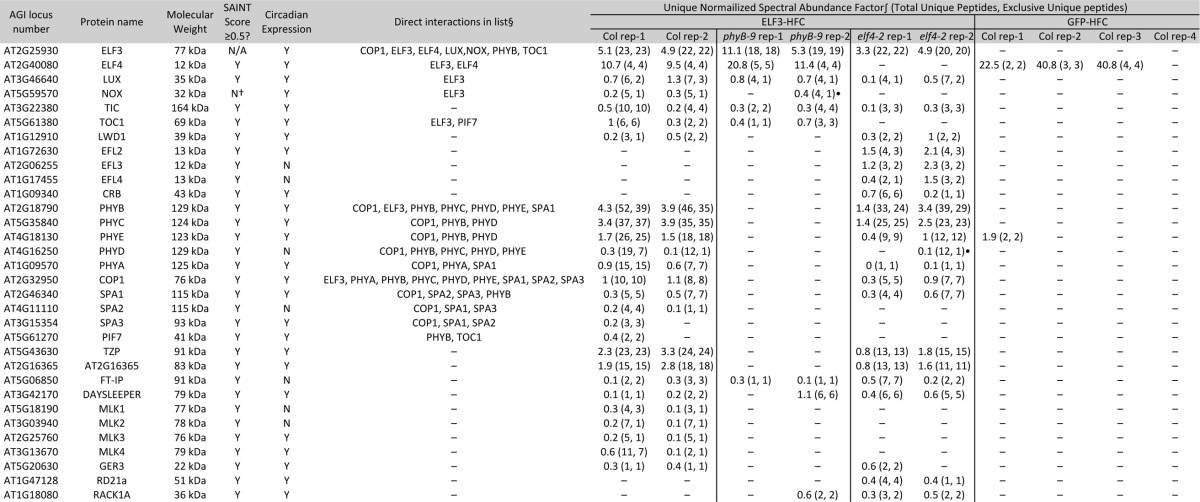
Identification of proteins associating with ELF3::ELF3-HFC #3 in elf3–2, elf3–2 phyB-9, and elf3–2 elf4–2 compared to GFP-HFC in wild type background. Note that the GFP-HFC identifications are shared with Table I. The unique Normalized Spectral Abundance Factor uses only unique peptides to quantify protein abundance in samples (98). Total unique peptide count refers to all uniquely identified peptides (including those shared with other proteins within the same protein family), while exclusive unique peptide count refers to peptides that are derived from and can be assigned to a single protein. ∫ = uNSAF was multiplied by 100 for comparison. § = Direct interaction that can be assigned from either the literature (see main text for references), or this study (See Fig. 2). † = NOX was identified by only one unique peptide, therefore it was excluded from SAINT analysis · = NOX and phyD are included as they each share multiple peptides with another protein in the list (LUX and phyB, respectively).

##### AP-MS of ELF3-HFC in the phyB Mutant Background

Next, ELF3-HFC was purified from the *phyB-9* background to determine which associations required the presence of phyB. Similar to the ELF4-HFC *phyB-9* AP-MS purifications, the results showed loss of interaction with many light signaling pathway components (phytochromes A, C-E, PIF7, COP1, SPAs, and TZP). ELF3 also lost association with LWD1, MLKs, GER3, and AT2G16365, suggesting that their coprecipitation with the evening complex requires the presence of phyB. DAYSLEEPER, TOC1, and TIC continue to coprecipitate, demonstrating that their association does not require *phyB*. In the absence of phyB, peptides are observed for RACK1A, a WD40 domain containing proteins with diverse roles in growth and defense (118–120).

##### AP-MS of ELF3-HFC in the elf4 Mutant Background

Although purification of ELF4 in the absence of ELF3 did not yield any specific interacting proteins, we hypothesized that purifying ELF3-HFC in the *elf4* mutant background might yield additional results. ELF3::ELF3-HFC #3 was crossed into *elf4–2*, and the circadian and growth phenotypes were measured. It was noted that the transgenic line retained rhythmic expression of the reporter (wild type (8/8), *elf4–2* (3/8), ELF3::ELF3-HFC #3 *elf3–2 elf4–2* (8/8) rhythmic) (supplemental Fig. S5*A* and S5*B*). Previous reports demonstrated that over expression of ELF3 cDNAs can restore low amplitude rhythms in *elf4* backgrounds (42). However, the ELF3::ELF3-HFC *elf3–2 elf4–2* line was very similar to the *elf4* mutant background in regards to the lower accuracy of circadian rhythms (much lower amplitude and expanded period range, supplemental Fig. S5*A*, S5*B*, S5*C*) and hypocotyl elongation (mean length- wild type 1.768 ± 0.2205 mm, *elf4–2* 3.092 ± 0.4514 mm, ELF3::ELF3-HFC #3 *elf3–2 elf4–2* 2.664 ± 0.2634 mm, error = S.D.) supplemental Fig. S5*D*), suggesting that the transgenic ELF3 behaved similarly to endogenous ELF3 when combined with the *elf4* mutation. In these purifications, peptides for ELF4, NOX, SPA2, and the most of the MLKs were not observed (identifications of TOC1, SPA3 and MLK2 were below the threshold for reliable identification (unique peptide = 1 in single replicate)) (Table II). However, many new proteins were identified, including the DUF-1313 domain-containing ELF4-like (EFL) proteins EFL2, EFL3, and EFL4. CHLOROPLAST RNA BINDING (CRB), a chloroplast-associated protein with roles in circadian oscillations (121), and the protease RD21a (105) was also observed.

##### ELF3 directly interacts with TOC1

**Fig. 2.**
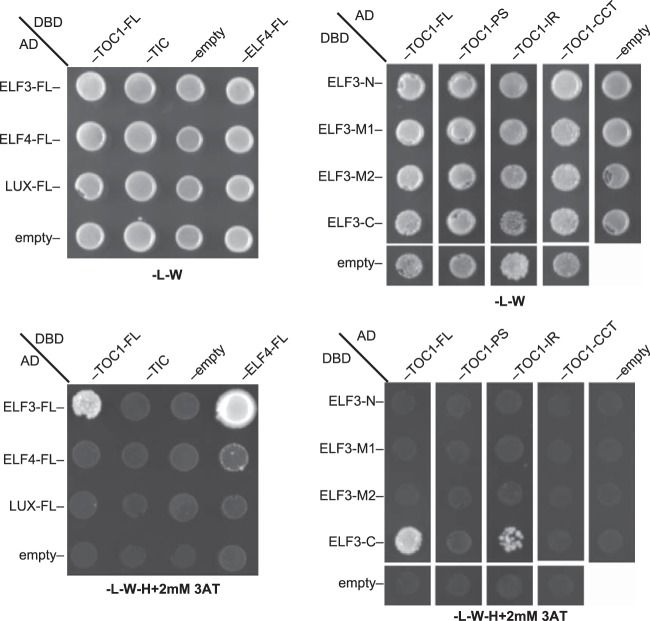
**Yeast two-hybrid analysis of interactions between circadian clock components.** *A*, TOC1 interacts with ELF3 of the evening complex. Full-length ELF3, ELF4, and LUX preys were tested for association with TOC1, TIC and ELF4 baits (ELF3 and ELF4 were a positive control). *B*, The intermediate region of TOC1 interacts with the C-terminal portion of ELF3. ELF3 was divided into four based on conserved regions and tested for interaction with the 3 major regions of TOC1- the pseudoreceiver (PS), intermediate region (IR), and CONSTANS (CO), CO-like, TOC1 (CCT) domain. DBD = GAL4 DNA binding domain, AD = GAL4 activation domain. Empty = empty vector (just DBD or AD) Top panel: -l–W selects for presence of plasmids, bottom -l–W-H+2 mm 3AT selects for interaction. Assays were repeated twice with similar results.

Our MS data confirm many of the direct interactions identified with alternative methods, such as yeast two-hybrid (34, 41, 42, 55, 61, 64, 65, 115, 122). However, some interactions were identified that could be evening complex specific, including TIC and TOC1. Both TIC and TOC1 are evening expressed genes, similar to the evening complex (supplemental Fig. S2). Previous studies identified a genetic interaction between ELF3 and TIC, however, a physical interaction had not yet been described (123). TIC and TOC1 were tested for direct interaction with the ELF3, ELF4 and LUX and found that TOC1 strongly and specifically interacted with ELF3 in yeast (Fig. 2*A*). To determine which regions of ELF3 and TOC1 directed interactions between these proteins, multiple fragments of ELF3 and TOC1 were tested, including the pseudoreceiver (PR) domain in the N terminus, the intermediate region (IR), and the CONSTANS (CO), CO-like, TOC1 (CCT) domain in the C terminus (Fig. 2*B*). Yeast two hybrid analysis mapped the interaction domain to the C-terminal portion of ELF3 and the IR of TOC1 (Fig. 2*B*).

##### Characterization of Novel Evening Complex Associated Kinases

**Fig. 3.**
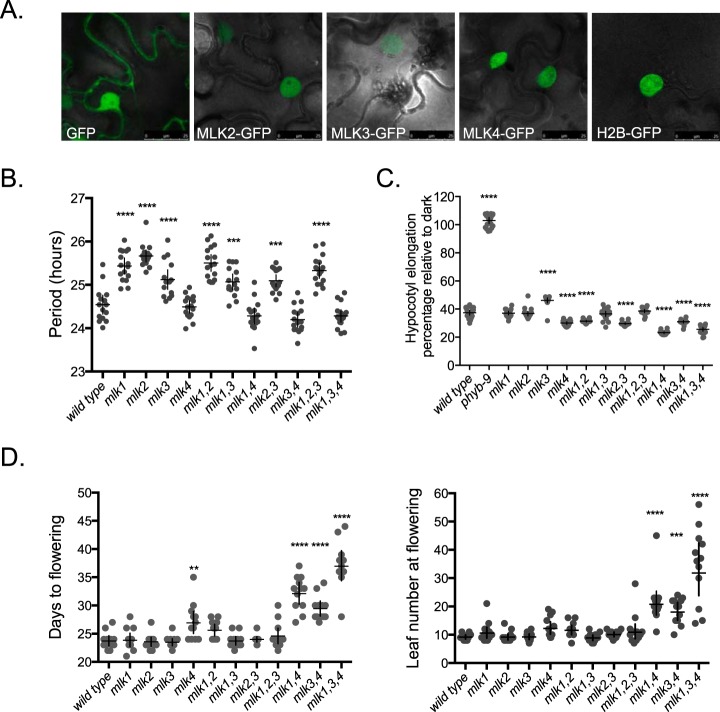
**Characterization of the MUT9-LIKE KINASES.** *A*, MLK2, MLK3, and MLK4 localize to the nucleus. Comparison of GFP, MLK2-GFP, MLK3-GFP, and MLK4-GFP to histone H2B-GFP (nuclear marker). Scale bar is 25 μm. *B*, Combining mutations in the MLKs alters circadian period in *A. thaliana* in response to 20 μmol m^−2^sec^−1^ of 660 nm red light. Circadian period of a *CCA1::LUC* bioluminescent reporter in single, double and triple *mlk* mutants. *n* = 16, line represents mean period, error is 95% confidence interval. Significance analysis is relative to wild type. Measurements were repeated twice with similar results. *C*, MLKs regulate hypocotyl elongation in response to red light. Hypocotyls of 4 day-old seedlings grown under 25 μmol/m^2^/s 660 nm constant red light were normalized to dark controls for each genotype. *n* = 20. Error is 95% confidence interval. Significance analysis is relative to wild type. Measurements were repeated twice in independent biological experiments with similar results. *D*, MLK4 regulates time to flowering. Left graph depicts time to a 1 cm inflorescence for individual plants grown in 16 h days, 8 h nights. Right graph depicts number of leaves when 1 cm inflorescence appears in individual plants grown in 16 h days, 8 h nights. *n* = 12, bar is mean and error is 95% confidence interval. Significance analysis is relative to wild type. Measurements were repeated three times in independent biological experiments with similar results. For all graphs, * ≤ 0.05, ** ≤ 0.01, *** 0.001, **** ≤ 0.0001.

AP-MS identified new proteins that coprecipitate with the evening complex, including all of the MUT9-LIKE KINASEs (MLKs). The MLKs were further characterized for their cellular localization and also for their roles in regulating circadian rhythms, flowering and growth. First, three of the four MLKs (2, 3, and 4) were successfully cloned, and Green Fluorescent Protein (GFP) translational fusions were generated to analyze their subcellular localization by transient expression in leaves of *Nicotiana benthamiana.* Similar to the observations for MLK1, these kinases also localize to the nucleus (Fig. 3*A*) (104). Next, single and higher order T-DNA insertion lines in the MLKs were generated to determine their function *in vivo*. All single mutants were successfully generated, but crossing was unable to generate any double or higher order mutants that contained *mlk2* and *mlk4* together (of 180 seedlings screened, zero were identified). These results suggest that in the absence of either MLK2 or MLK4 activity, the other kinase might be essential for proper development, however screening more seedlings would be required. All remaining combinations were generated in the *CCA1::LUC* background, and these lines were assayed for alterations in circadian rhythms. Because recruitment of the kinases required phyB (Table II), circadian rhythms were analyzed under constant red light conditions. When mutated, *mlk1, mlk2,* and *mlk3* each elongated the free running period of the *CCA1::LUC* reporter (Fig. 3*B*). When higher order mutant combinations were analyzed, the presence of MLK4 was absolutely required for period lengthening, and *mlk1, mlk2,* and *mlk3* exhibited an additive effect on period length (Fig. 3*B*). This suggests that the kinases act nonredundantly to regulate period length. When the effect on hypocotyl elongation in response to red light was analyzed, *mlk4* mutants (either alone or in combination) significantly shortened hypocotyls compared with wild type (Fig. 3*C*). *mlk3* mutants had slightly longer hypocotyls, but this was negated when combined as a double mutant with *mlk1, 2,* or *4.* Neither *mlk1* nor *mlk2* affected hypocotyl elongation alone, but the double mutant was statistically shorter than wild type. The *mlk1,2,3* triple was similar to wild type, whereas the *mlk1,3,4* was significantly shorter. When days to flowering in long day (16 h light/8 h dark) conditions were analyzed, MLK4 was required for proper flowering responses, and flowering was significantly delayed when combined with any other *mlk* mutant (Fig. 3*D*). These results suggest that each individual MLK functions nonredundantly in specific physiological pathways, and higher order mutations can exacerbate or ameliorate specific clock, flowering, and growth phenotypes.

## DISCUSSION

We used AP-MS to identify the protein partners of a key circadian multiprotein complex, the evening complex, *in vivo*. This method has led to the identification of new connections between key clock components, the evening complex, TOC1, TIC, and LWD1. We also find that the evening complex is highly connected to light signaling pathways, copurifying all red-light sensing phytochromes, the COP1-SPA complex, TZP and PIF7. In addition, several new proteins associating with the evening complex were also identified, including an F-box domain containing protein, FT-interacting protein, a transposase that is required for development, proteases, germin proteins, RACK1, and a clade of nuclear kinases that were characterized further. We also combined genetic perturbation with AP-MS to define how key factors mediate interactions among and between pathways. This method has helped to define roles for proteins as scaffolds and gatekeepers, promoting or excluding interactions.

### 

#### 

##### The Evening Complex Associates With Other Clock Proteins Through ELF3

Analysis of the results revealed multiple new interactions between the evening complex and clock-associated proteins, including TIC, TOC1, and LWD1 (Table I and II) (124–126). TIC is a gene of unknown function that regulates circadian rhythms, growth, metal homeostasis and defense (123, 125, 127–129). Previous analysis suggested that ELF3 and TIC genetically interact to maintain circadian rhythms, and with these data we demonstrate a physical interaction between these proteins (123) (Table I and II). TIC was shown to modulate the daily stability of proteins in the plant defense pathways and could be playing a similar role in regulating the components in the clock as well (128). The association of TIC with the evening complex and light signaling pathways suggests that TIC may associate with these components to regulate circadian rhythms, light signaling and plant defense. TOC1 is a member of the PSUDORESPONSE REGULATOR (PRR) family of transcription factors (25, 29, 30, 124, 130) that are critical regulators of growth, circadian rhythms and flowering. Direct binding of TOC1 to the evening complex further connects these critical evening-phased transcriptional regulators, as the evening complex is predicted to both regulate *TOC1* gene expression and TOC1 targets, such as PRR9 (17, 30, 42–45). Association of the evening complex with TOC1 to regulate transcriptional targets may provide another node connecting the evening complex to clock regulation. LWD1 is a light-regulated WD Domain factor that participates in the regulation of multiple clock genes and is required for proper flowering responses (126, 131). LWD1 is recruited to clock gene promoters and regulates their expression through an unknown mechanism. The targeting to specific genes could be through association with the evening complex and indirect recruitment to chromatin. In all cases, the association of these proteins with the evening complex required ELF3, and here we showed that TOC1 directly interacted with ELF3 in yeast.

It is important to note that peptides for GI were not identified, although it was previously shown to directly interact with ELF3 and ELF4 (61, 132). This could be explained by the nature of the association, as ELF4 sequesters GI into nuclear speckles, and speckle-associated ELF3 is predicted to function as an adapter to increase the turnover of GI by the E3-ligase COP1, and as a consequence any GI associated with the complex might be rapidly degraded. This is in contrast to the relatively stable GI-FKF1 complex that was detected using a similar AP-MS approach (77).

##### phyB Links the Evening Complex to Light Signaling Pathways

AP-MS has the capability to identify both direct and indirect interactions. phyB was the major phytochrome identified as coprecipitating with either ELF4 or ELF3. Previous work has shown that many of the light signaling factors interact with each other in a complex network. For example, phyB, C, D, and E can homo and heterodimerize in various combinations and all bind to COP1 (55, 56, 62). Futhermore, phyB also binds to SPA1 *in vivo* and in yeast two-hybrid assays (67). SPA1–4 form a complex with COP1 that regulates photomorphogenesis and development (64–66). Also, phyA can bind to COP1 in yeast two-hybrid assays and *in vivo* (63). PIF7 is a phyB-binding bHLH transcription factor that regulates daily responses to shade and cold (115, 133–135). Because phyB seemed to be the most interconnected of the light signaling proteins, we asked if loss of phyB would alter the composition of proteins coprecipitating with the evening complex members. We found that phyB is a key protein connecting the clock to light pathways through the association of phyB with the evening complex. Our results are consistent with the observation that phyB is the major regulator of red light signaling, and we propose that this is because of phyB acting as a key hub connecting many diverse pathways together, including circadian and light sensing networks.

##### ELF4 Acts as a Gatekeeper, Preventing Other DUF-1313 Proteins From Associating With ELF3

Because ELF4 did not associate with any specific proteins in the absence of ELF3, we tested if loss of *ELF4* would alter the composition of the evening complex. We found that without ELF4, ELF3 would associate with most of the DUF-1313 containing EFL family, suggesting that a function of ELF4 is to prevent other DUF-1313 domain containing proteins from associating with the evening complex. Although the EFLs can associate with the evening complex in the absence of ELF4, they cannot fully complement ELF4 function, which is consistent with genetic analysis of the EFLs (82). In addition to the EFLs, we also observe a gain of association of ELF3 with CRB, a chloroplast RNA binding factor that has been shown to alter circadian rhythms though altered chloroplast function (121). This association with CRB may be because of weaker nuclear recruitment in the *elf4* background, which is consistent with the proposed role of ELF4 regulating the subcellular distribution of the evening complex (42). The altered subcellular localization may have also changed the ability of ELF3 to interact with other nuclear localized factors, such as phyA, TOC1, and the MLKs. However, we cannot exclude the possibility that changes in protein abundance or stability underlies the loss of specific associated proteins from the complex.

##### AP-MS Identifies New Proteins That Associate With the Evening Complex

Several new proteins that coprecipitated with the evening complex were identified, including proteases, a germin, a hAT transposease, a florigen interacting protein, RACK1a, a 14–3-3 λ protein, a gene annotated as an F-box family protein, and a family of MUT9-LIKE kinases. Most of these genes exhibit circadian expression and some have been found to play a role in circadian regulated processes, signaling pathways, and development. The 14–3-3 protein λ, has been previously shown to bind to the brassinosteroid responsive transcription factor BZR1 (136) and the photoactivated form of the blue light photoreceptor PHOTOTROPIN1 (106). RACK1a acts as a scaffold in defense signaling pathways, and in mammalian systems, RACK1 homologs also participate in circadian rhythms (137). GER3 is a germin protein, which is part of a family of proteins with roles in metabolism, defense, stress responses, and growth (116). DAYSLEEPER is an hAT transposase that is required for proper embryonic development and localizes to the nucleus (117, 138). FT-IP1 functions in flowering by regulating the vascular transport of FT, the florigen molecule in *A. thaliana* (113). The F-box domain containing factor has been previously associated with integrating light and plastid signaling during deetiolation to modulate gene expression and chloroplast function (107). The subcellular localization, timing of expression, and functions of the identified proteins overlap with the evening complex and light signaling pathways, suggesting that these newly identified factors could specifically function in clock and photoperception pathways.

We further characterized a clade of protein kinases that coprecipitated with evening complex members in a phyB dependent manner. These kinases belong to a Casein Kinase I (CKI) clade that is found in both unicellular green algae and plants. This family of kinases is similar to OsCK1/EL1, a nuclear kinase that regulates brassinosteroid and gibberellin signaling in rice (139, 140). In *Chlamydomonas reinhardtii*, these kinases are similar in sequence to MUT-9, a nuclear kinase that modulates gene silencing by phosphorylating histone H3 on Thr^3^ (H3T3ph) (141). MUT-9 was also identified in a mutant screen for genes that regulate circadian rhythms in algae (142). Recently, the *A. thaliana* MUT9-LIKE KINASEs were characterized and both MLK1 and MLK2 were found to have roles in modulating H3T3ph levels at pericentromeric chromatin regions in response to osmotic stress (104). We found that like MLK1, the other MLKs (2–4) also localize to the nucleus when transiently expressed in *N. benthamiana* (Fig. 3*A*). We also observed that combining specific T-DNA insertion alleles of the kinases caused delay in time to flowering in long days, altered period length of a circadian reporter in red light, and altered hypocotyl elongation responses to red light (Fig. 3*B*–3*D*). These are all physiological processes that are also regulated by the evening complex and phytochromes. Interestingly, we were unable to generate a combination of two specific kinases (MLK2 and MLK4) by crossing, suggesting that they may be essential for proper development. The kinase mutants appeared to act nonredundantly and specific kinases regulated flowering time (MLK4), circadian period (MLKs1–4) and hypocotyl elongation (MLKs1–4). These kinases could modify distinct targets, or progressively modify a single target to modulate circadian rhythms and output pathways. Although distinct from the specific clade identified in this study, the CK1 family also functions in fungal and animal clocks, and in mammals, multiple CK1s act nonredundantly to phosphorylate circadian clock components, affecting circadian rhythms and physiology (143). Interestingly, mutations in a single *Drosophila melanogaster* CK1 that leads to lower kinase activity can either lengthen or shorten period, reflecting the complex role posttranslational modifications can play in the clock (144). Recently, ELF4 has been shown to be phosphorylated on ser^45^ *in vivo* and this modification site is important for circadian period, temperature compensation, and association with ELF3 (82, 145). Our results suggest that specific MLK combinations may play a role in post-translational modification of light and/or circadian components, or could possibly regulate gene expression directly through H3T3ph chromatin modifications (104, 140, 141). Future studies will focus on identifying substrates of the kinases, and how posttranslational modifications function in clock and light signaling pathways to regulate physiology and phenology.

## CONCLUSIONS

**Fig. 4.**
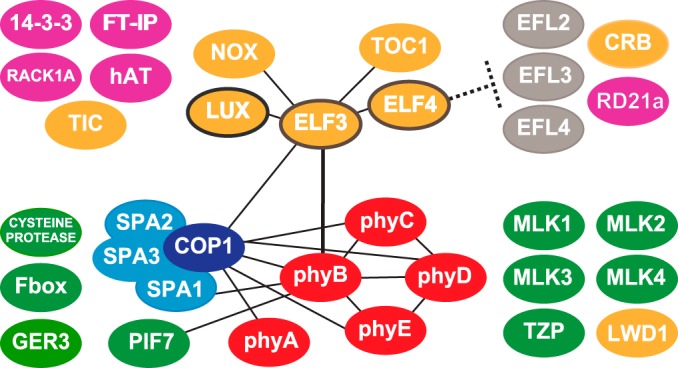
**Graphical summary of affinity purification results- ELF3 and phyB are two major hubs that link clock and light pathways.** Direct interactions identified in this manuscript or reported in the literature are denoted as lines. Clock factors are in yellow, the core evening complex members outlined in brown. In pink are new factors that continue to coprecipitate in the absence of phyB. ELF4 preventing the association of the EFLs and CRB with the evening complex is also shown. The phytochromes are depicted in red and the COP-SPA complex is depicted in blue. In green are the factors that no longer cofractionate with the evening complex in the absence of phyB.

We used AP-MS to identify protein partners of the evening-expressed clock-associated ELF4-ELF3-LUX complex. We identified 32 associated factors, including both unknown and known proteins with roles in the circadian clock, light signaling, and metabolism (Table I and II, as illustrated in Fig. 4). New associations were identified between the evening complex and circadian clock components, including TOC1, LWD1, and TIC. We found that phyB is a critical component stably connecting ELF3 to light signaling pathways *in vivo*, including the other phytochromes (phyA, C, D, and E), TZP, PIF7 and members of the COP1-SPA complex. In addition, we identified novel components within the circadian clock, flowering and growth pathways, including a conserved set of nuclear kinases. By combining genetic analysis and AP-MS, we are able to determine hierarchies of association and further clarify connections among this complex network of proteins. We find that ELF3 and phyB are major hubs connecting the evening complex to circadian and light signaling networks, reflecting their importance in these networks. These methods also identified a role for ELF4 as a gatekeeper, preventing the evening complex from associating with other proteins, including the CRB and the EFLs, likely by a combination of steric interference and maintaining the complex in the nucleus and/or nuclear speckles (42).

In *Arabidopsis thaliana*, the model organism for plant research, a majority of proteins (∼55%) lack functional annotation based on experimental evidence, and the absence of biochemical evidence for function is even greater (146). By using AP-MS to define physical interactions (either direct or indirect) between proteins, we were able to quickly characterize functional roles for a set of novel associated kinases in regulating light and clock outputs. This provides a rapid and robust method to define new connections between pathways, as well as to identify new components within well-studied networks.

## Supplementary Material

Supplemental Data

## Footnotes

^1^ The abbreviations used are:

AP-MS: affinity purification and mass spectrometry
FDR: false discovery rate
GO: gene ontology
RAE: relative amplitude error
SAINT: significance analysis of interactome
SD: standard deviation
uNSAF: unique Normalized Spectral Abundance Factor.
