# Supplementary material for: Identification of Evening Complex Associated Proteins in Arabidopsis by Affinity Purification and Mass Spectrometry
Source: Mol Cell Proteomics. 2015 Nov 6;15(1):201–17. doi: 10.1074/mcp.M115.054064 (PMC4762519; doi:10.1074/mcp.M115.054064)
Supplement: Supplemental Data [file supp_15_1_201__index.html]

Identification of evening complex associated proteins in Arabidopsis by affinity purification and mass spectrometry. — Identification of Evening Complex Associated Proteins in Arabidopsis by Affinity Purification and Mass Spectrometry — Evening Complex Interactome — Supplemental Data 

# Identification of Evening Complex Associated Proteins in *Arabidopsis* by Affinity Purification and Mass Spectrometry

## Supplemental Data

- Supplemental Figures (.pdf, 1.7 MB) - Supplemental Figures
