# Supplementary material for: Identification of Evening Complex Associated Proteins in Arabidopsis by Affinity Purification and Mass Spectrometry
Source: Mol Cell Proteomics. 2015 Nov 6;15(1):201–17. doi: 10.1074/mcp.M115.054064 (PMC4762519; doi:10.1074/mcp.M115.054064)

Supplemental legends and Data for:

Identification of evening complex associated proteins in *Arabidopsis* by affinity purification and mass spectrometry.

He Huang[1], Sophie Alvarez[1‡], Rebecca Bindbeutel[1], Zhouxin Shen[2], Michael J. Naldrett[1‡], Bradley S. Evans[1], Steven P. Briggs[2], Leslie M. Hicks[3], Steve A. Kay[4*], and Dmitri A. Nusinow[1*]

[1] Donald Danforth Plant Science Center, 975 N. Warson Road, St. Louis, MO, 63132, USA

[2] University of California San Diego, Division of Biological Sciences, Cell and Developmental Biology Section, 9500 Gilman Drive, La Jolla, CA 92093-0116, USA.

[3]The University of North Carolina at Chapel Hill, Department of Chemistry, Chapel Hill, NC 27599, USA

[4]University of Southern California, Molecular and Computational Biology Section, Los Angeles, CA 90089, USA.

Figure S1. Characterization of ELF4::ELF4-HFC complementation lines. A) Multiple lines independent ELF4::ELF4-HFC transgenic lines complement the *elf4-3* loss of function allele and restore *CAB::LUC* rhythms. Seedlings were entrained in 12 hours light and 12 hours dark conditions for 6 days before transferring to constant light. n= 16, error bars= 95% confidence interval. B) Scatter plots of average period length of ELF4::ELF4-HFC complementation lines. Measurements were repeated twice in independent biological experiments with similar results. C) Relative amplitude error (RAE) plots of complementation lines. The line at RAE =0.6 represents the upper bounds of values that are considered reliably rhythmic. D) ELF4::ELF4-HFC complementation lines rescue hypocotyl elongation defects of *elf4-3* mutants. Scatter plots show of hypocotyl length of the independent insertion lines. N≥20, line is the mean, and error is 95% confidence interval. Measurements were repeated twice in independent biological experiments with similar results.

Figure S2. Characterization of circadian expression patterns of identified co-interacting proteins. Data was derived from a circadian time course analysis in the *Col* background (110). The genes identified by AP-MS were determined to have a circadian expression pattern if their expression matched a cosine wave with an adjusted p value of ≤ 0.05, and were graphed. Black indicates genes common to both ELF4 and ELF3 pull downs (any genetic background), blue indicates genes specific to ELF4 pulldowns (any genetic background), and red are specific to ELF3 pulldowns (any genetic background).

Figure S3. Characterization of the ELF4::ELF4-HFC *elf3-1 elf4-3* lines. A) ELF4::ELF4-HFC *elf3-1 elf4-3* is arrhythmic. Comparison of wild type, *elf4-3 elf3-1,* ELF4::ELF4-HFC-51 *elf4-3,* and ELF4::ELF4-HFC *elf3-1 elf4-3* in constant light conditions. Seedlings were entrained in 12 hours light and 12 hours dark conditions for 6 days before transferring to constant light. n= 16, error bars= s.e.m. B) RAE plots of wild type, *elf4-3 elf3-1,* ELF4::ELF4-HFC-51 *elf4-3,* and ELF4::ELF4-HFC *elf3-1 elf4-3* lines. The line at RAE =0.6 represents the upper bounds of values that are considered reliably rhythmic. C) Comparison of hypocotyl elongation phenotypes of wild type, *elf4-3 elf3-1,* ELF4::ELF4-HFC-51 *elf4-3,* and ELF4::ELF4-HFC *elf3-1 elf4-3* lines. N=20. Measurements were repeated twice with similar results. Note that *wild type,* ELF4::ELF4-HFC-51 *elf4-3,* and *elf4-3* mutant data for all subpanels are shared with Figure 1 and S1, and were re-graphed for comparison.

Figure S4. Characterization of ELF3::ELF3-HFC complementation lines. A) Multiple lines independent ELF3::ELF3-HFC transgenic lines complement the *elf3-2* loss of function allele and restore *CCA1::LUC* rhythms. Seedlings were entrained in 12 hours light and 12 hours dark conditions for 6 days before transferring to constant light. n= 8, error = 95% confidence interval. Measurements were repeated twice in independent biological experiments with similar results. B) RAE plots of ELF3::ELF3-HFC complementation lines. The line at RAE =0.6 represents the upper bounds of values that are considered reliably rhythmic. C) Scatter plots of the period length of wild type, ELF3::ELF3-HFC #1 *elf3-2,*

ELF3::ELF3-HFC #3 *elf3-2,* and ELF3::ELF3-HFC #15 *elf3-2*. Line is at mean, n= 8, error = 95% confidence interval Measurements were repeated twice in independent biological experiments with similar results. D) ELF3::ELF3-HFC complementation lines also rescue hypocotyl elongation defects of *elf3-2* mutants. Scatter plots show the average and range of hypocotyl length of the independent insertion lines. N= 20. Measurements were repeated twice in independent biological experiments with similar results.

Figure S5. Characterization of the ELF3::ELF3-HFC #3 *elf3-2 elf4-2* lines. A ELF3::ELF3-HFC #3 *elf3-2 elf4-2* has dampened rhythms. Comparison of wild type, *elf4-2,* ELF3::ELF3-HFC #3 *elf3-2,* and ELF3::ELF3-HFC #3 *elf3-2 elf4-2* in constant light conditions. Seedlings were entrained in 12 hours light and 12 hours dark conditions for 6 days before transferring to constant light. error = 95% confidence interval. Measurements were repeated twice in independent biological experiments with similar results. B) RAE plots of wild type, *elf4-2,* ELF3::ELF3-HFC #3 *elf3-2,* and ELF3::ELF3-HFC #3 *elf3-2 elf4-2.* The line at RAE =0.6 represents the upper bounds of values that are considered reliably rhythmic. Measurements were repeated twice in independent biological experiments with similar results. C) Scatter plots of the period length of wild type, *elf4-2,* ELF3::ELF3-HFC #3 *elf3-2,* and ELF3::ELF3-HFC #3 *elf3-2 elf4-2* lines. Line is at mean, n= 8, error = 95% confidence interval. Measurements were repeated twice in independent biological experiments with similar results. D) Characterization ELF3::ELF3-HFC #3 *elf4-2* lines hypocotyl elongation phenotypes. Scatter plots show the average and range of hypocotyl length of the independent insertion lines. N= 20. Measurements were repeated twice in independent biological experiments with similar results. Note that *wild type,* ELF3::ELF3-HFC #3 *elf3-2* and *elf3-2* mutant data in all subpanels are shared with Figure S4, and re-graphed for comparison.

Figure S6. Characterization of the circadian rhythmicity of the *mlk* lines. A) Wild type compared to the *mlk* single mutants. Seedlings were entrained in 12 hours light and 12 hours dark conditions for 6 days before transferring to constant red light. N=15-16, error = 95% confidence interval. Measurements were

repeated twice with similar results. B) RAE plots of wild type and *mlk* single mutants. The line at RAE =0.5 represents the upper bounds of values that are considered reliably rhythmic. Measurements were repeated twice with similar results. C) Wild type compared to the *mlk* double mutants. Seedlings were entrained in 12 hours light and 12 hours dark conditions for 6 days before transferring to constant red light. N=15-16, error = 95% confidence interval. Measurements were repeated twice in independent biological experiments with similar results. D) RAE plots of wild type and *mlk* double mutants. The line at RAE =0.6 represents the upper bounds of values that are considered reliably rhythmic. Measurements were repeated twice in independent biological experiments with similar results. E) Wild type compared to the *mlk* triple mutants. Seedlings were entrained in 12 hours light and 12 hours dark conditions for 6 days before transferring to constant red light. N=15-16, error = 95% confidence interval. Measurements were repeated twice with similar results. F) RAE plots of wild type and *mlk* triple mutants. The line at RAE =0.6 represents the upper bounds of values that are considered reliably rhythmic. Measurements were repeated twice in independent biological experiments with similar results. Note that the data for *wild type* are shared among all panels for comparison.

Table S1. Gene ontology enrichment of proteins that co-precipitate with ELF4-HFC in the elf4-3 background. AGRIGO website (http://bioinfo.cau.edu.cn/agriGO/) output for queried Arabidopsis locus IDs that co-precipitate with ELF4::ELF4-HFC #51.

GO term	Ontology	Description	Number in input list	Number in Backgro	p-value	FDR
GO:0048511	Process	rhythmic process	6	75	9.80E-13	1.10E-10
GO:0007623	Process	circadian rhythm	6	75	9.80E-13	1.10E-10
GO:0010017	Process	red or far-red light signaling pathway	5	41	1.30E-11	9.80E-10
GO:0050789	Process	regulation of biological process	13	3697	1.30E-10	7.10E-09
GO:0009639	Process	response to red or far red light	6	210	3.80E-10	1.70E-08
GO:0065007	Process	biological regulation	13	4188	6.00E-10	2.30E-08
GO:0009416	Process	response to light stimulus	7	596	4.30E-09	1.40E-07
GO:0009314	Process	response to radiation	7	613	5.20E-09	1.50E-07
GO:0050794	Process	regulation of cellular process	11	3375	2.20E-08	5.50E-07
GO:0051716	Process	cellular response to stimulus	7	840	4.40E-08	1.00E-06
GO:0007242	Process	intracellular signaling cascade	6	659	3.00E-07	6.20E-06
GO:0009605	Process	response to external stimulus	5	429	1.10E-06	2.00E-05
GO:0009628	Process	response to abiotic stimulus	7	1471	1.90E-06	3.30E-05
GO:0019219	Process	regulation of nucleobase, nucleoside, nucleotide and nucleic acid metabolic process	7	1847	8.50E-06	0.00012
GO:0031326	Process	regulation of cellular biosynthetic process	7	1881	9.60E-06	0.00012
GO:0009889	Process	regulation of biosynthetic process	7	1881	9.60E-06	0.00012
GO:0051171	Process	regulation of nitrogen compound metabolic process	7	1888	9.80E-06	0.00012
GO:0010556	Process	regulation of macromolecule biosynthetic process	7	1843	8.40E-06	0.00012
GO:0045449	Process	regulation of transcription	7	1802	7.30E-06	0.00012
GO:0007165	Process	signal transduction	6	1228	1.10E-05	0.00012
GO:0006350	Process	transcription	7	1923	1.10E-05	0.00012
GO:0009791	Process	post-embryonic development	5	705	1.20E-05	0.00012
GO:0080090	Process	regulation of primary metabolic process	7	1952	1.20E-05	0.00012
GO:0010468	Process	regulation of gene expression	7	2001	1.40E-05	0.00013
GO:0031323	Process	regulation of cellular metabolic process	7	2036	1.60E-05	0.00014
GO:0060255	Process	regulation of macromolecule metabolic process	7	2060	1.70E-05	0.00015
GO:0032501	Process	multicellular organismal process	7	2094	1.90E-05	0.00016
GO:0019222	Process	regulation of metabolic process	7	2210	2.70E-05	0.00022
GO:0006139	Process	nucleobase, nucleoside, nucleotide and nucleic acid metabolic process	8	3198	3.20E-05	0.00025
GO:0006355	Process	regulation of transcription, DNA-dependent	5	987	5.90E-05	0.00044
GO:0051252	Process	regulation of RNA metabolic process	5	996	6.10E-05	0.00044
GO:0032774	Process	RNA biosynthetic process	5	1040	7.50E-05	0.00051
GO:0006351	Process	transcription, DNA-dependent	5	1039	7.50E-05	0.00051
GO:0006807	Process	nitrogen compound metabolic process	8	3826	0.00012	0.00077
GO:0009987	Process	cellular process	13	11684	0.00015	0.00095
GO:0050896	Process	response to stimulus	8	4057	0.00018	0.0011
GO:0043170	Process	macromolecule metabolic process	10	7127	0.0003	0.0018
GO:0044238	Process	primary metabolic process	11	8995	0.0004	0.0024
GO:0044249	Process	cellular biosynthetic process	8	4925	0.00068	0.0036
GO:0034645	Process	cellular macromolecule biosynthetic process	7	3661	0.00065	0.0036
GO:0009059	Process	macromolecule biosynthetic process	7	3685	0.00068	0.0036
GO:0016070	Process	RNA metabolic process	5	1657	0.00065	0.0036
GO:0048856	Process	anatomical structure development	5	1726	0.00078	0.0041
GO:0044260	Process	cellular macromolecule metabolic process	9	6447	0.0008	0.0041
GO:0009058	Process	biosynthetic process	8	5118	0.00088	0.0044
GO:0010467	Process	gene expression	7	3962	0.0011	0.0051
GO:0007275	Process	multicellular organismal development	5	2020	0.0016	0.0076
GO:0044237	Process	cellular metabolic process	10	8722	0.0016	0.0077
GO:0008152	Process	metabolic process	11	10614	0.0018	0.0084
GO:0032502	Process	developmental process	5	2304	0.0028	0.013

AGRIGO query using these gene IDs
AT2G25930
AT3G46640
AT1G12910
AT3G22380
AT5G61380
AT2G18790
AT5G35840
AT4G18130
AT4G16250
AT1G09570
AT2G32950
AT2G46340
AT5G43630
AT2G16365
AT3G13670
AT3G19390
AT5G10450

Background list contained annotated 37767 genes from TAIR9

Table S2. Gene ontology enrichment of proteins that co-precipitate with ELF3::ELF3-HFC in the elf3-2 background. AGRIGO website (http://bioinfo.cau.edu.cn/agriGO/) output for queried Arabidopsis locus IDs that co-precipitate with ELF3::ELF3-HFC #3.

GO term	Ontology	Description	Number in input list	Number in Backgro	p-value	FDR
GO:0048511	Process	rhythmic process	7	75	2.60E-13	2.90E-11
GO:0007623	Process	circadian rhythm	7	75	2.60E-13	2.90E-11
GO:0009639	Process	response to red or far red light	7	210	2.60E-10	1.40E-08
GO:0010017	Process	red or far-red light signaling pathway	5	41	2.50E-10	1.40E-08
GO:0009416	Process	response to light stimulus	8	596	1.30E-08	5.70E-07
GO:0009314	Process	response to radiation	8	613	1.60E-08	5.90E-07
GO:0009628	Process	response to abiotic stimulus	10	1471	8.40E-08	2.60E-06
GO:0050789	Process	regulation of biological process	14	3697	1.40E-07	3.80E-06
GO:0065007	Process	biological regulation	14	4188	6.60E-07	1.60E-05
GO:0009791	Process	post-embryonic development	7	705	8.90E-07	2.00E-05
GO:0050794	Process	regulation of cellular process	12	3375	3.20E-06	6.40E-05
GO:0045449	Process	regulation of transcription	9	1802	5.50E-06	0.0001
GO:0019219	Process	regulation of nucleobase, nucleoside, nucleotide and nucleic acid metabolic process	9	1847	6.70E-06	0.0001
GO:0031326	Process	regulation of cellular biosynthetic process	9	1881	7.70E-06	0.0001
GO:0009889	Process	regulation of biosynthetic process	9	1881	7.70E-06	0.0001
GO:0051171	Process	regulation of nitrogen compound metabolic process	9	1888	8.00E-06	0.0001
GO:0010556	Process	regulation of macromolecule biosynthetic process	9	1843	6.50E-06	0.0001
GO:0006350	Process	transcription	9	1923	9.20E-06	0.00011
GO:0080090	Process	regulation of primary metabolic process	9	1952	1.00E-05	0.00012
GO:0006139	Process	nucleobase, nucleoside, nucleotide and nucleic acid metabolic process	11	3198	1.30E-05	0.00014
GO:0010468	Process	regulation of gene expression	9	2001	1.30E-05	0.00014
GO:0031323	Process	regulation of cellular metabolic process	9	2036	1.50E-05	0.00015
GO:0060255	Process	regulation of macromolecule metabolic process	9	2060	1.60E-05	0.00015
GO:0032501	Process	multicellular organismal process	9	2094	1.80E-05	0.00017
GO:0044260	Process	cellular macromolecule metabolic process	15	6447	2.10E-05	0.00018
GO:0019222	Process	regulation of metabolic process	9	2210	2.80E-05	0.00024
GO:0051716	Process	cellular response to stimulus	6	840	3.80E-05	0.00031
GO:0006807	Process	nitrogen compound metabolic process	11	3826	7.10E-05	0.00054
GO:0043170	Process	macromolecule metabolic process	15	7127	7.20E-05	0.00054
GO:0007242	Process	intracellular signaling cascade	5	659	0.00014	0.001
GO:0044237	Process	cellular metabolic process	16	8722	0.00019	0.0013
GO:0044238	Process	primary metabolic process	16	8995	0.00028	0.0019
GO:0010467	Process	gene expression	10	3962	0.00049	0.0033
GO:0009987	Process	cellular process	18	11684	0.00053	0.0034
GO:0050896	Process	response to stimulus	10	4057	0.0006	0.0037
GO:0007275	Process	multicellular organismal development	7	2020	0.00069	0.0042
GO:0006355	Process	regulation of transcription, DNA-dependent	5	987	0.00087	0.0051
GO:0051252	Process	regulation of RNA metabolic process	5	996	0.0009	0.0052
GO:0032774	Process	RNA biosynthetic process	5	1040	0.0011	0.006
GO:0006351	Process	transcription, DNA-dependent	5	1039	0.0011	0.006
GO:0034645	Process	cellular macromolecule biosynthetic process	9	3661	0.0013	0.0067
GO:0009059	Process	macromolecule biosynthetic process	9	3685	0.0013	0.0069
GO:0016070	Process	RNA metabolic process	6	1657	0.0014	0.0073
GO:0032502	Process	developmental process	7	2304	0.0015	0.0075
GO:0008152	Process	metabolic process	16	10614	0.002	0.0097
GO:0007165	Process	signal transduction	5	1228	0.0023	0.011
GO:0044249	Process	cellular biosynthetic process	10	4925	0.0027	0.013
GO:0009058	Process	biosynthetic process	10	5118	0.0036	0.016
GO:0048856	Process	anatomical structure development	5	1726	0.0095	0.043

AGRIGO query using these gene IDs

AT2G40080
AT3G46640
AT5G59570
AT3G22380
AT5G61380
AT1G12910
AT1G72630
AT2G06255
AT1G17455
AT1G09340
AT2G18790
AT5G35840
AT4G18130
AT4G16250
AT1G09570
AT2G32950
AT2G46340
AT4G11110
AT3G15354
AT5G61270
AT5G43630
AT2G16365
AT5G06850
AT3G42170
AT3G13670
AT3G03940
AT5G18190
AT2G25760
AT5G20630

Background list contained annotated 37767 genes from TAIR9

Table S3. Peptide and Protein Identification for proteins that were identified in ELF4::ELF4-HFC (all genotypes) and GFP-HFC controls as listed in Table 1.

Biological sample name	Protein name	Protein accession numbers	Database sources	Protein molecular weight (Da)	Protein identification probability	Exclusive unique peptide count	Exclusive unique spectrum count	Total spectrum count	Percentage of total spectra	Percentage sequence coverage	Peptide sequence	Best Peptide identification probability	Best Mascot Ion score	Best Mascot Identity score	Best Mascot Delta Ion score	Calculated +1H Peptide Mass (AMU)
ELF4-HFC Col rep 1	| Symbols: | F-box family protein | chr2:7074940-7079181 FORWARD LENGTH=727	AT2G16365.4	TAIR10_20101214.fasta	82,716.20	100.00%	12	13	15	0.07%	23.70%	ALSGLNPYGLR	99.70%	57.9	30.4	79.8	1,566.86
ELF4-HFC Col rep 1	| Symbols: | F-box family protein | chr2:7074940-7079181 FORWARD LENGTH=727	AT2G16365.4	TAIR10_20101214.fasta	82,716.20	100.00%	12	13	15	0.07%	23.70%	ENNNLFLEILK	99.70%	73	30.2	42.5	1,820.86
ELF4-HFC Col rep 1	| Symbols: | F-box family protein | chr2:7074940-7079181 FORWARD LENGTH=727	AT2G16365.4	TAIR10_20101214.fasta	82,716.20	100.00%	12	13	15	0.07%	23.70%	ETTTTTTTKPSFHEDVGSSSR	99.70%	66.7	30.4	70.7	1,512.84
ELF4-HFC Col rep 1	| Symbols: | F-box family protein | chr2:7074940-7079181 FORWARD LENGTH=727	AT2G16365.4	TAIR10_20101214.fasta	82,716.20	100.00%	12	13	15	0.07%	23.70%	IFGGYLDLFPNQDHSHNR	99.70%	44.9	30.6	81.3	2,146.99
ELF4-HFC Col rep 1	| Symbols: | F-box family protein | chr2:7074940-7079181 FORWARD LENGTH=727	AT2G16365.4	TAIR10_20101214.fasta	82,716.20	100.00%	12	13	15	0.07%	23.70%	IYATVDSVNLPK	99.70%	73.7	29	101	2,568.23
ELF4-HFC Col rep 1	| Symbols: | F-box family protein | chr2:7074940-7079181 FORWARD LENGTH=727	AT2G16365.4	TAIR10_20101214.fasta	82,716.20	100.00%	12	13	15	0.07%	23.70%	LDFPIQEK	99.00%	44.2	30.2	34.5	2,289.06
ELF4-HFC Col rep 1	| Symbols: | F-box family protein | chr2:7074940-7079181 FORWARD LENGTH=727	AT2G16365.4	TAIR10_20101214.fasta	82,716.20	100.00%	12	13	15	0.07%	23.70%	NQEPVVSQSNDLR	99.70%	68.4	29.3	77.3	2,318.98
ELF4-HFC Col rep 1	| Symbols: | F-box family protein | chr2:7074940-7079181 FORWARD LENGTH=727	AT2G16365.4	TAIR10_20101214.fasta	82,716.20	100.00%	12	13	15	0.07%	23.70%	NSSVNPYESAWLGR	99.70%	74.4	30	117	1,512.84
ELF4-HFC Col rep 1	| Symbols: | F-box family protein | chr2:7074940-7079181 FORWARD LENGTH=727	AT2G16365.4	TAIR10_20101214.fasta	82,716.20	100.00%	12	13	15	0.07%	23.70%	QGGDDITLLHPWIQR	99.60%	33.8	30.7	97.4	2,162.98
ELF4-HFC Col rep 1	| Symbols: | F-box family protein | chr2:7074940-7079181 FORWARD LENGTH=727	AT2G16365.4	TAIR10_20101214.fasta	82,716.20	100.00%	12	13	15	0.07%	23.70%	TTQNLLELIRPVR	99.70%	37.1	25	21.2	1,906.94
ELF4-HFC Col rep 1	| Symbols: | F-box family protein | chr2:7074940-7079181 FORWARD LENGTH=727	AT2G16365.4	TAIR10_20101214.fasta	82,716.20	100.00%	12	13	15	0.07%	23.70%	TTSTDQPTGQEASFEPESHKEFEK	99.70%	46.9	29.1	14.9	1,240.71
ELF4-HFC Col rep 1	| Symbols: | F-box family protein | chr2:7074940-7079181 FORWARD LENGTH=727	AT2G16365.4	TAIR10_20101214.fasta	82,716.20	100.00%	12	13	15	0.07%	23.70%	VRLEPEVEASSR	99.20%	38.1	29.1	33	898.4993
ELF4-HFC Col rep 1	| Symbols: ATAN11, LWD1 | Transducin/WD40 repeat-like superfamily protein | chr1:4395114-4396154 REVERSE LENGTH=346	AT1G12910.1	TAIR10_20101214.fasta	39,089.30	100.00%	2	2	4	0.02%	14.70%	DKEHSTIIYESSEPDTPLVR	99.70%	52.5	31.8	89.1	2,302.99
ELF4-HFC Col rep 1	| Symbols: ATAN11, LWD1 | Transducin/WD40 repeat-like superfamily protein | chr1:4395114-4396154 REVERSE LENGTH=346	AT1G12910.1	TAIR10_20101214.fasta	39,089.30	100.00%	2	2	4	0.02%	14.70%	IGTSSTDTTCTIWDIER	99.70%	89.9	29.7	96.6	1,512.84
ELF4-HFC Col rep 1	| Symbols: ATAN11, LWD1 | Transducin/WD40 repeat-like superfamily protein | chr1:4395114-4396154 REVERSE LENGTH=346	AT1G12910.1	TAIR10_20101214.fasta	39,089.30	100.00%	2	2	4	0.02%	14.70%	VEIVQLDESNGEIR	99.70%	61.8	29.9	10.9	2,460.07
ELF4-HFC Col rep 1	| Symbols: COP1, ATCOP1, DET340, FUS1, EMB168 | Transducin/WD40 repeat-like superfamily protein | chr2:13978000-13983282 FORWARD LENGTH=675	AT2G32950.1	TAIR10_20101214.fasta	76,188.00	100.00%	4	4	5	0.02%	6.81%	EVDNLLTLLAER	99.70%	47.9	28.3	99.2	2,146.99
ELF4-HFC Col rep 1	| Symbols: COP1, ATCOP1, DET340, FUS1, EMB168 | Transducin/WD40 repeat-like superfamily protein | chr2:13978000-13983282 FORWARD LENGTH=675	AT2G32950.1	TAIR10_20101214.fasta	76,188.00	100.00%	4	4	5	0.02%	6.81%	FLSNNELASASTDSTLR	99.70%	114	30.5	30.3	1,170.60
ELF4-HFC Col rep 1	| Symbols: COP1, ATCOP1, DET340, FUS1, EMB168 | Transducin/WD40 repeat-like superfamily protein | chr2:13978000-13983282 FORWARD LENGTH=675	AT2G32950.1	TAIR10_20101214.fasta	76,188.00	100.00%	4	4	5	0.02%	6.81%	QKVDELNEVQTDLQYIK	99.00%	28.4	31	30.4	1,906.94
ELF4-HFC Col rep 1	| Symbols: COP1, ATCOP1, DET340, FUS1, EMB168 | Transducin/WD40 repeat-like superfamily protein | chr2:13978000-13983282 FORWARD LENGTH=675	AT2G32950.1	TAIR10_20101214.fasta	76,188.00	100.00%	4	4	5	0.02%	6.81%	VDELNEVQTDLQYIK	99.70%	48.1	31.3	18	1,298.70
ELF4-HFC Col rep 1	| Symbols: ELF3, PYK20 | hydroxyproline-rich glycoprotein family protein | chr2:11059459-11063178 FORWARD LENGTH=695	AT2G25930.1	TAIR10_20101214.fasta	77,204.80	100.00%	15	19	24	0.12%	30.10%	EEEDFAVPVYINSR	99.70%	59.6	29.1	19	898.4993
ELF4-HFC Col rep 1	| Symbols: ELF3, PYK20 | hydroxyproline-rich glycoprotein family protein | chr2:11059459-11063178 FORWARD LENGTH=695	AT2G25930.1	TAIR10_20101214.fasta	77,204.80	100.00%	15	19	24	0.12%	30.10%	IFQSIQEER	99.70%	73	30.1	24.4	1,942.05
ELF4-HFC Col rep 1	| Symbols: ELF3, PYK20 | hydroxyproline-rich glycoprotein family protein | chr2:11059459-11063178 FORWARD LENGTH=695	AT2G25930.1	TAIR10_20101214.fasta	77,204.80	100.00%	15	19	24	0.12%	30.10%	KAIANQQR	99.00%	45.3	25.8	42.7	1,160.64
ELF4-HFC Col rep 1	| Symbols: ELF3, PYK20 | hydroxyproline-rich glycoprotein family protein | chr2:11059459-11063178 FORWARD LENGTH=695	AT2G25930.1	TAIR10_20101214.fasta	77,204.80	100.00%	15	19	24	0.12%	30.10%	LASENAAR	99.00%	56.1	28.7	57	1,346.73
ELF4-HFC Col rep 1	| Symbols: ELF3, PYK20 | hydroxyproline-rich glycoprotein family protein | chr2:11059459-11063178 FORWARD LENGTH=695	AT2G25930.1	TAIR10_20101214.fasta	77,204.80	100.00%	15	19	24	0.12%	30.10%	LHVNDADK	99.00%	44.5	27.4	63.9	2,269.06
ELF4-HFC Col rep 1	| Symbols: ELF3, PYK20 | hydroxyproline-rich glycoprotein family protein | chr2:11059459-11063178 FORWARD LENGTH=695	AT2G25930.1	TAIR10_20101214.fasta	77,204.80	100.00%	15	19	24	0.12%	30.10%	LIAASPDLLLDEISFLGK	99.70%	108	25.9	32.8	2,130.02
ELF4-HFC Col rep 1	| Symbols: ELF3, PYK20 | hydroxyproline-rich glycoprotein family protein | chr2:11059459-11063178 FORWARD LENGTH=695	AT2G25930.1	TAIR10_20101214.fasta	77,204.80	100.00%	15	19	24	0.12%	30.10%	NLSVQHLDSSAANQATEK	99.70%	107	31	55.7	1,319.72
ELF4-HFC Col rep 1	| Symbols: ELF3, PYK20 | hydroxyproline-rich glycoprotein family protein | chr2:11059459-11063178 FORWARD LENGTH=695	AT2G25930.1	TAIR10_20101214.fasta	77,204.80	100.00%	15	19	24	0.12%	30.10%	QGSTGSSPSGPQGISGSK	99.70%	94.3	29.1	12.8	989.5304
ELF4-HFC Col rep 1	| Symbols: ELF3, PYK20 | hydroxyproline-rich glycoprotein family protein | chr2:11059459-11063178 FORWARD LENGTH=695	AT2G25930.1	TAIR10_20101214.fasta	77,204.80	100.00%	15	19	24	0.12%	30.10%	QNVCLATCSKPEVR	99.70%	60.3	30.2	58.8	1,485.73
ELF4-HFC Col rep 1	| Symbols: ELF3, PYK20 | hydroxyproline-rich glycoprotein family protein | chr2:11059459-11063178 FORWARD LENGTH=695	AT2G25930.1	TAIR10_20101214.fasta	77,204.80	100.00%	15	19	24	0.12%	30.10%	SDNEPAPQQQQQPTK	99.70%	74.4	29.6	61.2	1,579.75
ELF4-HFC Col rep 1	| Symbols: ELF3, PYK20 | hydroxyproline-rich glycoprotein family protein | chr2:11059459-11063178 FORWARD LENGTH=695	AT2G25930.1	TAIR10_20101214.fasta	77,204.80	100.00%	15	19	24	0.12%	30.10%	SFRPFAAVDEDSNINNAPEQTMTTTTTTTR	99.70%	40.3	31	26.8	1,748.91
ELF4-HFC Col rep 1	| Symbols: ELF3, PYK20 | hydroxyproline-rich glycoprotein family protein | chr2:11059459-11063178 FORWARD LENGTH=695	AT2G25930.1	TAIR10_20101214.fasta	77,204.80	100.00%	15	19	24	0.12%	30.10%	SNNTSTLVHPGPSSQPCGVER	99.70%	37.4	30.4	33.2	1,552.92
ELF4-HFC Col rep 1	| Symbols: ELF3, PYK20 | hydroxyproline-rich glycoprotein family protein | chr2:11059459-11063178 FORWARD LENGTH=695	AT2G25930.1	TAIR10_20101214.fasta	77,204.80	100.00%	15	19	24	0.12%	30.10%	TTVTQTTR	99.00%	40.8	28.3	40.5	2,710.21
ELF4-HFC Col rep 1	| Symbols: ELF3, PYK20 | hydroxyproline-rich glycoprotein family protein | chr2:11059459-11063178 FORWARD LENGTH=695	AT2G25930.1	TAIR10_20101214.fasta	77,204.80	100.00%	15	19	24	0.12%	30.10%	VFAVQLFELHR	99.70%	45.3	28.8	33.9	1,371.72
ELF4-HFC Col rep 1	| Symbols: ELF3, PYK20 | hydroxyproline-rich glycoprotein family protein | chr2:11059459-11063178 FORWARD LENGTH=695	AT2G25930.1	TAIR10_20101214.fasta	77,204.80	100.00%	15	19	24	0.12%	30.10%	VNDYNASLR	99.70%	74.9	28.8	46.8	2,316.14
ELF4-HFC Col rep 1	| Symbols: ELF4 | Protein of unknown function (DUF1313) | chr2:16734545-16734880 REVERSE LENGTH=111	AT2G40080.1	TAIR10_20101214.fasta	12,375.40	100.00%	7	27	143	0.68%	79.30%	NGHDGGGAAGTR	99.70%	50.2	25.7	82.4	1,955.90
ELF4-HFC Col rep 1	| Symbols: ELF4 | Protein of unknown function (DUF1313) | chr2:16734545-16734880 REVERSE LENGTH=111	AT2G40080.1	TAIR10_20101214.fasta	12,375.40	100.00%	7	27	143	0.68%	79.30%	NVAEEAEQGEDPAMWENLDR	99.70%	107	26.4	52.4	1,600.82
ELF4-HFC Col rep 1	| Symbols: ELF4 | Protein of unknown function (DUF1313) | chr2:16734545-16734880 REVERSE LENGTH=111	AT2G40080.1	TAIR10_20101214.fasta	12,375.40	100.00%	7	27	143	0.68%	79.30%	NVALIQELNGNISK	99.70%	128	28.7	33.4	1,385.76
ELF4-HFC Col rep 1	| Symbols: ELF4 | Protein of unknown function (DUF1313) | chr2:16734545-16734880 REVERSE LENGTH=111	AT2G40080.1	TAIR10_20101214.fasta	12,375.40	100.00%	7	27	143	0.68%	79.30%	QVQSVLDR	99.00%	53.7	29.2	103	1,825.89
ELF4-HFC Col rep 1	| Symbols: ELF4 | Protein of unknown function (DUF1313) | chr2:16734545-16734880 REVERSE LENGTH=111	AT2G40080.1	TAIR10_20101214.fasta	12,375.40	100.00%	7	27	143	0.68%	79.30%	RNVAEEAEQGEDPAMWENLDR	99.70%	67.3	28	18.1	2,063.07
ELF4-HFC Col rep 1	| Symbols: ELF4 | Protein of unknown function (DUF1313) | chr2:16734545-16734880 REVERSE LENGTH=111	AT2G40080.1	TAIR10_20101214.fasta	12,375.40	100.00%	7	27	143	0.68%	79.30%	SLIQQVNDNHQSR	99.70%	75.8	30.2	37.4	1,806.91
ELF4-HFC Col rep 1	| Symbols: ELF4 | Protein of unknown function (DUF1313) | chr2:16734545-16734880 REVERSE LENGTH=111	AT2G40080.1	TAIR10_20101214.fasta	12,375.40	100.00%	7	27	143	0.68%	79.30%	VVNMYSDLNTSFSSGFHGGK	99.70%	86.6	27.8	49.4	1,668.78
ELF4-HFC Col rep 1	| Symbols: PCL1, LUX | Homeodomain-like superfamily protein | chr3:17183248-17184219 FORWARD LENGTH=323	AT3G46640.1,AT3G46640.2	TAIR10_20101214.fasta	34,648.50	100.00%	3	3	4	0.02%	15.50%	ASETTLSSLR	99.70%	81.3	28.9	46.1	1,149.59
ELF4-HFC Col rep 1	| Symbols: PCL1, LUX | Homeodomain-like superfamily protein | chr3:17183248-17184219 FORWARD LENGTH=323	AT3G46640.1,AT3G46640.2	TAIR10_20101214.fasta	34,648.50	100.00%	3	3	4	0.02%	15.50%	ENVASHLQK	99.70%	52.2	28.1	28.8	928.5323
ELF4-HFC Col rep 1	| Symbols: PCL1, LUX | Homeodomain-like superfamily protein | chr3:17183248-17184219 FORWARD LENGTH=323	AT3G46640.1,AT3G46640.2	TAIR10_20101214.fasta	34,648.50	100.00%	3	3	4	0.02%	15.50%	GGSSGPNTSSSNNNVEEEDR	99.70%	92.9	25	40.8	831.4318
ELF4-HFC Col rep 1	| Symbols: PCL1, LUX | Homeodomain-like superfamily protein | chr3:17183248-17184219 FORWARD LENGTH=323	AT3G46640.1,AT3G46640.2	TAIR10_20101214.fasta	34,648.50	100.00%	3	3	4	0.02%	15.50%	GGSSGPNTSSSNNNVEEEDRVGSSSPGSDSK	99.70%	44.2	25.5	33.6	911.4582
ELF4-HFC Col rep 1	| Symbols: PHYA, FHY2, FRE1, HY8 | phytochrome A | chr1:3095498-3099216 REVERSE LENGTH=1122	AT1G09570.1	TAIR10_20101214.fasta	124,503.20	100.00%	6	6	7	0.03%	8.65%	ADAGPISLVVNACASR	99.70%	56.8	29.5	92.4	1,915.08
ELF4-HFC Col rep 1	| Symbols: PHYA, FHY2, FRE1, HY8 | phytochrome A | chr1:3095498-3099216 REVERSE LENGTH=1122	AT1G09570.1	TAIR10_20101214.fasta	124,503.20	100.00%	6	6	7	0.03%	8.65%	ALGFGDVSLLNPILVHCR	99.70%	36.6	28.3	103	1,912.94
ELF4-HFC Col rep 1	| Symbols: PHYA, FHY2, FRE1, HY8 | phytochrome A | chr1:3095498-3099216 REVERSE LENGTH=1122	AT1G09570.1	TAIR10_20101214.fasta	124,503.20	100.00%	6	6	7	0.03%	8.65%	LIETATVPILAVDSDGLVNGWNTK	99.70%	75.6	28.9	84.2	1,618.77
ELF4-HFC Col rep 1	| Symbols: PHYA, FHY2, FRE1, HY8 | phytochrome A | chr1:3095498-3099216 REVERSE LENGTH=1122	AT1G09570.1	TAIR10_20101214.fasta	124,503.20	100.00%	6	6	7	0.03%	8.65%	LIQPFGCLLALDEK	99.70%	45	29.9	50.1	1,661.81
ELF4-HFC Col rep 1	| Symbols: PHYA, FHY2, FRE1, HY8 | phytochrome A | chr1:3095498-3099216 REVERSE LENGTH=1122	AT1G09570.1	TAIR10_20101214.fasta	124,503.20	100.00%	6	6	7	0.03%	8.65%	SLFTAPSASALQK	99.70%	60.5	29.6	64	1,695.79
ELF4-HFC Col rep 1	| Symbols: PHYA, FHY2, FRE1, HY8 | phytochrome A | chr1:3095498-3099216 REVERSE LENGTH=1122	AT1G09570.1	TAIR10_20101214.fasta	124,503.20	100.00%	6	6	7	0.03%	8.65%	TSAKPFYAIIHR	98.60%	25.7	29.2	33.1	3,332.53
ELF4-HFC Col rep 1	| Symbols: PHYB, HY3, OOP1 | phytochrome B | chr2:8140079-8144151 FORWARD LENGTH=1172	AT2G18790.1	TAIR10_20101214.fasta	129,333.90	100.00%	25	35	52	0.25%	39.40%	AIQQYTVDAR	99.70%	60.3	29.8	30.1	2,224.04
ELF4-HFC Col rep 1	| Symbols: PHYB, HY3, OOP1 | phytochrome B | chr2:8140079-8144151 FORWARD LENGTH=1172	AT2G18790.1	TAIR10_20101214.fasta	129,333.90	100.00%	25	35	52	0.25%	39.40%	AISQLQALPGGDIK	99.70%	85.8	26.5	31.6	907.4844
ELF4-HFC Col rep 1	| Symbols: PHYB, HY3, OOP1 | phytochrome B | chr2:8140079-8144151 FORWARD LENGTH=1172	AT2G18790.1	TAIR10_20101214.fasta	129,333.90	100.00%	25	35	52	0.25%	39.40%	AVFVVVNACSSK	99.70%	83.4	29.6	31.6	1,358.76
ELF4-HFC Col rep 1	| Symbols: PHYB, HY3, OOP1 | phytochrome B | chr2:8140079-8144151 FORWARD LENGTH=1172	AT2G18790.1	TAIR10_20101214.fasta	129,333.90	100.00%	25	35	52	0.25%	39.40%	CDGAAFLYHGK	99.70%	45.9	27.5	58.6	1,051.52
ELF4-HFC Col rep 1	| Symbols: PHYB, HY3, OOP1 | phytochrome B | chr2:8140079-8144151 FORWARD LENGTH=1172	AT2G18790.1	TAIR10_20101214.fasta	129,333.90	100.00%	25	35	52	0.25%	39.40%	DYLNNIVGVCFVGQDVTSQK	99.70%	104	31.5	47.8	1,069.48
ELF4-HFC Col rep 1	| Symbols: PHYB, HY3, OOP1 | phytochrome B | chr2:8140079-8144151 FORWARD LENGTH=1172	AT2G18790.1	TAIR10_20101214.fasta	129,333.90	100.00%	25	35	52	0.25%	39.40%	EITLLNPVWIHSK	99.70%	76.2	26.9	103	2,302.99
ELF4-HFC Col rep 1	| Symbols: PHYB, HY3, OOP1 | phytochrome B | chr2:8140079-8144151 FORWARD LENGTH=1172	AT2G18790.1	TAIR10_20101214.fasta	129,333.90	100.00%	25	35	52	0.25%	39.40%	ELAYICQVIK	99.70%	45.6	28.8	38.5	1,513.82
ELF4-HFC Col rep 1	| Symbols: PHYB, HY3, OOP1 | phytochrome B | chr2:8140079-8144151 FORWARD LENGTH=1172	AT2G18790.1	TAIR10_20101214.fasta	129,333.90	100.00%	25	35	52	0.25%	39.40%	ENEATVNK	99.00%	39.9	28.5	13.7	944.516
ELF4-HFC Col rep 1	| Symbols: PHYB, HY3, OOP1 | phytochrome B | chr2:8140079-8144151 FORWARD LENGTH=1172	AT2G18790.1	TAIR10_20101214.fasta	129,333.90	100.00%	25	35	52	0.25%	39.40%	FANSLLEATDLNEDQK	99.70%	126	31.1	59	2,475.08
ELF4-HFC Col rep 1	| Symbols: PHYB, HY3, OOP1 | phytochrome B | chr2:8140079-8144151 FORWARD LENGTH=1172	AT2G18790.1	TAIR10_20101214.fasta	129,333.90	100.00%	25	35	52	0.25%	39.40%	FHEDEHGEVVAESK	99.70%	97.6	27.3	65	1,538.77
ELF4-HFC Col rep 1	| Symbols: PHYB, HY3, OOP1 | phytochrome B | chr2:8140079-8144151 FORWARD LENGTH=1172	AT2G18790.1	TAIR10_20101214.fasta	129,333.90	100.00%	25	35	52	0.25%	39.40%	FINIQGDYK	99.70%	61	28	29.6	2,163.97
ELF4-HFC Col rep 1	| Symbols: PHYB, HY3, OOP1 | phytochrome B | chr2:8140079-8144151 FORWARD LENGTH=1172	AT2G18790.1	TAIR10_20101214.fasta	129,333.90	100.00%	25	35	52	0.25%	39.40%	GGEEEPSSSHTPNNR	99.70%	69.3	25.4	66	1,064.56
ELF4-HFC Col rep 1	| Symbols: PHYB, HY3, OOP1 | phytochrome B | chr2:8140079-8144151 FORWARD LENGTH=1172	AT2G18790.1	TAIR10_20101214.fasta	129,333.90	100.00%	25	35	52	0.25%	39.40%	IVGDMDLESIEDGSFVLK	99.70%	89.2	31	28.9	1,025.54
ELF4-HFC Col rep 1	| Symbols: PHYB, HY3, OOP1 | phytochrome B | chr2:8140079-8144151 FORWARD LENGTH=1172	AT2G18790.1	TAIR10_20101214.fasta	129,333.90	100.00%	25	35	52	0.25%	39.40%	LHAVFEQSGESGK	99.70%	67.8	29.2	90.1	2,036.84
ELF4-HFC Col rep 1	| Symbols: PHYB, HY3, OOP1 | phytochrome B | chr2:8140079-8144151 FORWARD LENGTH=1172	AT2G18790.1	TAIR10_20101214.fasta	129,333.90	100.00%	25	35	52	0.25%	39.40%	LIETATVPIFAVDAGGCINGWNAK	99.30%	44.2	31.3	42.8	3,025.28
ELF4-HFC Col rep 1	| Symbols: PHYB, HY3, OOP1 | phytochrome B | chr2:8140079-8144151 FORWARD LENGTH=1172	AT2G18790.1	TAIR10_20101214.fasta	129,333.90	100.00%	25	35	52	0.25%	39.40%	LLCDTVVESVR	99.70%	48.4	30.6	40.2	1,600.81
ELF4-HFC Col rep 1	| Symbols: PHYB, HY3, OOP1 | phytochrome B | chr2:8140079-8144151 FORWARD LENGTH=1172	AT2G18790.1	TAIR10_20101214.fasta	129,333.90	100.00%	25	35	52	0.25%	39.40%	MIVDCNATPVLVVQDDR	99.70%	52.8	30.7	34	1,981.07
ELF4-HFC Col rep 1	| Symbols: PHYB, HY3, OOP1 | phytochrome B | chr2:8140079-8144151 FORWARD LENGTH=1172	AT2G18790.1	TAIR10_20101214.fasta	129,333.90	100.00%	25	35	52	0.25%	39.40%	NTGKPFYAILHR	99.70%	58.9	29.6	0	2,527.33
ELF4-HFC Col rep 1	| Symbols: PHYB, HY3, OOP1 | phytochrome B | chr2:8140079-8144151 FORWARD LENGTH=1172	AT2G18790.1	TAIR10_20101214.fasta	129,333.90	100.00%	25	35	52	0.25%	39.40%	QLLETSVSCEK	99.70%	57.8	30.6	32.8	1,616.87
ELF4-HFC Col rep 1	| Symbols: PHYB, HY3, OOP1 | phytochrome B | chr2:8140079-8144151 FORWARD LENGTH=1172	AT2G18790.1	TAIR10_20101214.fasta	129,333.90	100.00%	25	35	52	0.25%	39.40%	RDDLEPYIGLHYPATDIPQASR	99.70%	38.9	31.4	52.3	1,320.72
ELF4-HFC Col rep 1	| Symbols: PHYB, HY3, OOP1 | phytochrome B | chr2:8140079-8144151 FORWARD LENGTH=1172	AT2G18790.1	TAIR10_20101214.fasta	129,333.90	100.00%	25	35	52	0.25%	39.40%	RQDTECFTK	99.00%	34.2	26.2	13	1,403.78
ELF4-HFC Col rep 1	| Symbols: PHYB, HY3, OOP1 | phytochrome B | chr2:8140079-8144151 FORWARD LENGTH=1172	AT2G18790.1	TAIR10_20101214.fasta	129,333.90	100.00%	25	35	52	0.25%	39.40%	SFDYSQSLK	99.70%	44.8	26.6	50.8	1,164.60
ELF4-HFC Col rep 1	| Symbols: PHYB, HY3, OOP1 | phytochrome B | chr2:8140079-8144151 FORWARD LENGTH=1172	AT2G18790.1	TAIR10_20101214.fasta	129,333.90	100.00%	25	35	52	0.25%	39.40%	SIEVFGDQIR	99.70%	60.4	30.5	74.6	1,410.80
ELF4-HFC Col rep 1	| Symbols: PHYB, HY3, OOP1 | phytochrome B | chr2:8140079-8144151 FORWARD LENGTH=1172	AT2G18790.1	TAIR10_20101214.fasta	129,333.90	100.00%	25	35	52	0.25%	39.40%	SLFTSSSSILLER	99.70%	72.7	28.4	72.6	1,280.67
ELF4-HFC Col rep 1	| Symbols: PHYB, HY3, OOP1 | phytochrome B | chr2:8140079-8144151 FORWARD LENGTH=1172	AT2G18790.1	TAIR10_20101214.fasta	129,333.90	100.00%	25	35	52	0.25%	39.40%	SLVSDLIYK	99.70%	63.2	26.3	31.5	1,238.56
ELF4-HFC Col rep 1	| Symbols: PHYB, HY3, OOP1 | phytochrome B | chr2:8140079-8144151 FORWARD LENGTH=1172	AT2G18790.1	TAIR10_20101214.fasta	129,333.90	100.00%	25	35	52	0.25%	39.40%	SLVSDLIYKENEATVNK	99.70%	40.8	30.3	0	2,257.08
ELF4-HFC Col rep 1	| Symbols: PHYB, HY3, OOP1 | phytochrome B | chr2:8140079-8144151 FORWARD LENGTH=1172	AT2G18790.1	TAIR10_20101214.fasta	129,333.90	100.00%	25	35	52	0.25%	39.40%	SSFQAFLEVVK	99.70%	70.9	27.5	57.7	1,549.87
ELF4-HFC Col rep 1	| Symbols: PHYB, HY3, OOP1 | phytochrome B | chr2:8140079-8144151 FORWARD LENGTH=1172	AT2G18790.1	TAIR10_20101214.fasta	129,333.90	100.00%	25	35	52	0.25%	39.40%	TEDPALSIAGAVQSQK	99.70%	108	29.4	34.1	1,236.67
ELF4-HFC Col rep 1	| Symbols: PHYB, HY3, OOP1 | phytochrome B | chr2:8140079-8144151 FORWARD LENGTH=1172	AT2G18790.1	TAIR10_20101214.fasta	129,333.90	100.00%	25	35	52	0.25%	39.40%	TFSPELQGK	98.50%	33.3	30	31.3	904.437
ELF4-HFC Col rep 1	| Symbols: PHYB, HY3, OOP1 | phytochrome B | chr2:8140079-8144151 FORWARD LENGTH=1172	AT2G18790.1	TAIR10_20101214.fasta	129,333.90	100.00%	25	35	52	0.25%	39.40%	TTTYGSSVPEQQITAYLSR	99.70%	61.9	31	81.8	1,808.86
ELF4-HFC Col rep 1	| Symbols: PHYB, HY3, OOP1 | phytochrome B | chr2:8140079-8144151 FORWARD LENGTH=1172	AT2G18790.1	TAIR10_20101214.fasta	129,333.90	100.00%	25	35	52	0.25%	39.40%	VIGAFCFLQIPSPELQQALAVQR	99.70%	51.9	28.7	90	1,612.72
ELF4-HFC Col rep 1	| Symbols: PHYB, HY3, OOP1 | phytochrome B | chr2:8140079-8144151 FORWARD LENGTH=1172	AT2G18790.1	TAIR10_20101214.fasta	129,333.90	100.00%	25	35	52	0.25%	39.40%	VVDGVVQPCR	99.60%	42.9	27.2	42.8	1,097.56
ELF4-HFC Col rep 1	| Symbols: PHYB, HY3, OOP1 | phytochrome B | chr2:8140079-8144151 FORWARD LENGTH=1172	AT2G18790.1	TAIR10_20101214.fasta	129,333.90	100.00%	25	35	52	0.25%	39.40%	WTSPEGLGLSVCR	99.70%	47.6	29.8	56.1	1,597.68
ELF4-HFC Col rep 1	| Symbols: PHYB, HY3, OOP1 | phytochrome B | chr2:8140079-8144151 FORWARD LENGTH=1172	AT2G18790.1	TAIR10_20101214.fasta	129,333.90	100.00%	25	35	52	0.25%	39.40%	YAPSQEWVEIHLSQLSK	99.70%	62.4	30.7	77.9	1,982.96
ELF4-HFC Col rep 1	| Symbols: PHYB, HY3, OOP1 | phytochrome B | chr2:8140079-8144151 FORWARD LENGTH=1172	AT2G18790.1	TAIR10_20101214.fasta	129,333.90	100.00%	25	35	52	0.25%	39.40%	YYPLGVAPSEVQIK	99.70%	66.5	29.6	61.2	1,388.68
ELF4-HFC Col rep 1	| Symbols: PHYC | phytochrome C | chr5:14008049-14011619 FORWARD LENGTH=1111	AT5G35840.1	TAIR10_20101214.fasta	123,724.60	100.00%	29	34	38	0.18%	34.40%	AAEVTGLAVEQAIGKPVSDLVEDDSVETVK	99.70%	85.6	30.5	43.8	2,517.28
ELF4-HFC Col rep 1	| Symbols: PHYC | phytochrome C | chr5:14008049-14011619 FORWARD LENGTH=1111	AT5G35840.1	TAIR10_20101214.fasta	123,724.60	100.00%	29	34	38	0.18%	34.40%	AISFLQDLLHSSGLSEDQK	99.70%	71.4	30.9	34.4	1,290.67
ELF4-HFC Col rep 1	| Symbols: PHYC | phytochrome C | chr5:14008049-14011619 FORWARD LENGTH=1111	AT5G35840.1	TAIR10_20101214.fasta	123,724.60	100.00%	29	34	38	0.18%	34.40%	AISFLQDLLHSSGLSEDQKR	99.70%	32.8	30.4	52.8	1,960.95
ELF4-HFC Col rep 1	| Symbols: PHYC | phytochrome C | chr5:14008049-14011619 FORWARD LENGTH=1111	AT5G35840.1	TAIR10_20101214.fasta	123,724.60	100.00%	29	34	38	0.18%	34.40%	AVDFGEISILNPITLHCR	99.70%	58.9	30	45.3	1,416.77
ELF4-HFC Col rep 1	| Symbols: PHYC | phytochrome C | chr5:14008049-14011619 FORWARD LENGTH=1111	AT5G35840.1	TAIR10_20101214.fasta	123,724.60	100.00%	29	34	38	0.18%	34.40%	CDGAALYYR	99.70%	54.2	25	50.7	1,293.64
ELF4-HFC Col rep 1	| Symbols: PHYC | phytochrome C | chr5:14008049-14011619 FORWARD LENGTH=1111	AT5G35840.1	TAIR10_20101214.fasta	123,724.60	100.00%	29	34	38	0.18%	34.40%	DGSFIEALLSANK	99.70%	108	29.8	0	2,527.26
ELF4-HFC Col rep 1	| Symbols: PHYC | phytochrome C | chr5:14008049-14011619 FORWARD LENGTH=1111	AT5G35840.1	TAIR10_20101214.fasta	123,724.60	100.00%	29	34	38	0.18%	34.40%	DGSFIEALLSANKR	99.70%	49.5	29.9	6.7	1,184.54
ELF4-HFC Col rep 1	| Symbols: PHYC | phytochrome C | chr5:14008049-14011619 FORWARD LENGTH=1111	AT5G35840.1	TAIR10_20101214.fasta	123,724.60	100.00%	29	34	38	0.18%	34.40%	DLIDWVLK	99.00%	38.7	28.8	31.4	1,074.51
ELF4-HFC Col rep 1	| Symbols: PHYC | phytochrome C | chr5:14008049-14011619 FORWARD LENGTH=1111	AT5G35840.1	TAIR10_20101214.fasta	123,724.60	100.00%	29	34	38	0.18%	34.40%	DNLWSLGVTPTETQIR	99.70%	53.9	30.5	41.1	1,163.61
ELF4-HFC Col rep 1	| Symbols: PHYC | phytochrome C | chr5:14008049-14011619 FORWARD LENGTH=1111	AT5G35840.1	TAIR10_20101214.fasta	123,724.60	100.00%	29	34	38	0.18%	34.40%	EAESAVLLK	99.70%	59.2	30.5	67.1	1,439.77
ELF4-HFC Col rep 1	| Symbols: PHYC | phytochrome C | chr5:14008049-14011619 FORWARD LENGTH=1111	AT5G35840.1	TAIR10_20101214.fasta	123,724.60	100.00%	29	34	38	0.18%	34.40%	EALTIGTDVK	97.80%	34.1	29.8	50.8	1,037.59
ELF4-HFC Col rep 1	| Symbols: PHYC | phytochrome C | chr5:14008049-14011619 FORWARD LENGTH=1111	AT5G35840.1	TAIR10_20101214.fasta	123,724.60	100.00%	29	34	38	0.18%	34.40%	EGLGLHITQK	99.70%	41.2	25.9	33.7	1,923.01
ELF4-HFC Col rep 1	| Symbols: PHYC | phytochrome C | chr5:14008049-14011619 FORWARD LENGTH=1111	AT5G35840.1	TAIR10_20101214.fasta	123,724.60	100.00%	29	34	38	0.18%	34.40%	EVSELTGYDR	99.70%	54.9	27.5	54.8	1,254.67
ELF4-HFC Col rep 1	| Symbols: PHYC | phytochrome C | chr5:14008049-14011619 FORWARD LENGTH=1111	AT5G35840.1	TAIR10_20101214.fasta	123,724.60	100.00%	29	34	38	0.18%	34.40%	GSLQEEHSK	99.70%	52.6	25.9	94	1,614.83
ELF4-HFC Col rep 1	| Symbols: PHYC | phytochrome C | chr5:14008049-14011619 FORWARD LENGTH=1111	AT5G35840.1	TAIR10_20101214.fasta	123,724.60	100.00%	29	34	38	0.18%	34.40%	IEEGLVIDLEPVSPDEVPVTAAGALR	99.70%	37.9	29.1	29.3	1,006.52
ELF4-HFC Col rep 1	| Symbols: PHYC | phytochrome C | chr5:14008049-14011619 FORWARD LENGTH=1111	AT5G35840.1	TAIR10_20101214.fasta	123,724.60	100.00%	29	34	38	0.18%	34.40%	IGFNAVISGQK	99.70%	63.5	28.6	53.7	2,102.04
ELF4-HFC Col rep 1	| Symbols: PHYC | phytochrome C | chr5:14008049-14011619 FORWARD LENGTH=1111	AT5G35840.1	TAIR10_20101214.fasta	123,724.60	100.00%	29	34	38	0.18%	34.40%	IIHPAPGLPEDLVR	99.70%	57.8	26	48.7	2,585.39
ELF4-HFC Col rep 1	| Symbols: PHYC | phytochrome C | chr5:14008049-14011619 FORWARD LENGTH=1111	AT5G35840.1	TAIR10_20101214.fasta	123,724.60	100.00%	29	34	38	0.18%	34.40%	ILQTQSVLCDMLFR	99.70%	65.2	29.8	37.9	1,128.58
ELF4-HFC Col rep 1	| Symbols: PHYC | phytochrome C | chr5:14008049-14011619 FORWARD LENGTH=1111	AT5G35840.1	TAIR10_20101214.fasta	123,724.60	100.00%	29	34	38	0.18%	34.40%	LIDTAAVPIFAVDASGVINGWNSK	99.70%	61.7	30.7	37.7	1,461.72
ELF4-HFC Col rep 1	| Symbols: PHYC | phytochrome C | chr5:14008049-14011619 FORWARD LENGTH=1111	AT5G35840.1	TAIR10_20101214.fasta	123,724.60	100.00%	29	34	38	0.18%	34.40%	LLFGFYHR	99.00%	38.8	27.6	48.5	2,015.02
ELF4-HFC Col rep 1	| Symbols: PHYC | phytochrome C | chr5:14008049-14011619 FORWARD LENGTH=1111	AT5G35840.1	TAIR10_20101214.fasta	123,724.60	100.00%	29	34	38	0.18%	34.40%	LQQILSETLLSSIR	99.70%	118	26.2	58.8	1,563.84
ELF4-HFC Col rep 1	| Symbols: PHYC | phytochrome C | chr5:14008049-14011619 FORWARD LENGTH=1111	AT5G35840.1	TAIR10_20101214.fasta	123,724.60	100.00%	29	34	38	0.18%	34.40%	NAPIGIVTQSPNIMDLVK	99.70%	60	28.7	77.2	3,069.58
ELF4-HFC Col rep 1	| Symbols: PHYC | phytochrome C | chr5:14008049-14011619 FORWARD LENGTH=1111	AT5G35840.1	TAIR10_20101214.fasta	123,724.60	100.00%	29	34	38	0.18%	34.40%	SLFLSPGCSALEK	99.70%	65.2	30.2	62	2,088.06
ELF4-HFC Col rep 1	| Symbols: PHYC | phytochrome C | chr5:14008049-14011619 FORWARD LENGTH=1111	AT5G35840.1	TAIR10_20101214.fasta	123,724.60	100.00%	29	34	38	0.18%	34.40%	SLSQPISLSGSTLR	99.70%	85.2	29.3	31.1	2,244.16
ELF4-HFC Col rep 1	| Symbols: PHYC | phytochrome C | chr5:14008049-14011619 FORWARD LENGTH=1111	AT5G35840.1	TAIR10_20101214.fasta	123,724.60	100.00%	29	34	38	0.18%	34.40%	SSPVELVVNTCCSR	99.70%	70.8	29.1	50.5	2,055.07
ELF4-HFC Col rep 1	| Symbols: PHYC | phytochrome C | chr5:14008049-14011619 FORWARD LENGTH=1111	AT5G35840.1	TAIR10_20101214.fasta	123,724.60	100.00%	29	34	38	0.18%	34.40%	SSSKPFYAILHR	99.70%	48	29.4	46.4	1,088.48
ELF4-HFC Col rep 1	| Symbols: PHYC | phytochrome C | chr5:14008049-14011619 FORWARD LENGTH=1111	AT5G35840.1	TAIR10_20101214.fasta	123,724.60	100.00%	29	34	38	0.18%	34.40%	TVVDVPLVDNR	99.70%	48.7	26.9	92.2	1,364.71
ELF4-HFC Col rep 1	| Symbols: PHYC | phytochrome C | chr5:14008049-14011619 FORWARD LENGTH=1111	AT5G35840.1	TAIR10_20101214.fasta	123,724.60	100.00%	29	34	38	0.18%	34.40%	VSSQVLVDAK	99.70%	65.7	29.5	34.1	1,520.81
ELF4-HFC Col rep 1	| Symbols: PHYC | phytochrome C | chr5:14008049-14011619 FORWARD LENGTH=1111	AT5G35840.1	TAIR10_20101214.fasta	123,724.60	100.00%	29	34	38	0.18%	34.40%	YACEFLTQVFGVQINK	99.70%	65.4	30.5	16.2	1,001.57
ELF4-HFC Col rep 1	| Symbols: PHYD | phytochrome D | chr4:9195602-9199486 REVERSE LENGTH=1164	AT4G16250.1	TAIR10_20101214.fasta	129,270.30	100.00%	1	1	12	0.06%	11.00%	AIQQYTVDAR	99.70%	60.3	29.8	39.2	1,829.94
ELF4-HFC Col rep 1	| Symbols: PHYD | phytochrome D | chr4:9195602-9199486 REVERSE LENGTH=1164	AT4G16250.1	TAIR10_20101214.fasta	129,270.30	100.00%	1	1	12	0.06%	11.00%	FHEDEHGEVVAESK	99.70%	97.6	27.3	39.9	959.5408
ELF4-HFC Col rep 1	| Symbols: PHYD | phytochrome D | chr4:9195602-9199486 REVERSE LENGTH=1164	AT4G16250.1	TAIR10_20101214.fasta	129,270.30	100.00%	1	1	12	0.06%	11.00%	FINIQGDYK	99.70%	61	28	23.2	1,046.57
ELF4-HFC Col rep 1	| Symbols: PHYD | phytochrome D | chr4:9195602-9199486 REVERSE LENGTH=1164	AT4G16250.1	TAIR10_20101214.fasta	129,270.30	100.00%	1	1	12	0.06%	11.00%	LHAVFEQSGESGK	99.70%	67.8	29.2	26.8	1,095.62
ELF4-HFC Col rep 1	| Symbols: PHYD | phytochrome D | chr4:9195602-9199486 REVERSE LENGTH=1164	AT4G16250.1	TAIR10_20101214.fasta	129,270.30	100.00%	1	1	12	0.06%	11.00%	LLCDTVVESVR	99.70%	48.4	30.6	38	1,168.55
ELF4-HFC Col rep 1	| Symbols: PHYD | phytochrome D | chr4:9195602-9199486 REVERSE LENGTH=1164	AT4G16250.1	TAIR10_20101214.fasta	129,270.30	100.00%	1	1	12	0.06%	11.00%	NDLEPYIGLHYPATDIPQASR	99.70%	46.8	31.3	37	1,014.49
ELF4-HFC Col rep 1	| Symbols: PHYD | phytochrome D | chr4:9195602-9199486 REVERSE LENGTH=1164	AT4G16250.1	TAIR10_20101214.fasta	129,270.30	100.00%	1	1	12	0.06%	11.00%	QLLETSVSCEK	99.70%	57.8	30.6	31.5	2,689.43
ELF4-HFC Col rep 1	| Symbols: PHYD | phytochrome D | chr4:9195602-9199486 REVERSE LENGTH=1164	AT4G16250.1	TAIR10_20101214.fasta	129,270.30	100.00%	1	1	12	0.06%	11.00%	RNDLEPYIGLHYPATDIPQASR	99.70%	38.9	31.4	44.7	1,133.63
ELF4-HFC Col rep 1	| Symbols: PHYD | phytochrome D | chr4:9195602-9199486 REVERSE LENGTH=1164	AT4G16250.1	TAIR10_20101214.fasta	129,270.30	100.00%	1	1	12	0.06%	11.00%	SFDYSQSLK	99.70%	44.8	26.6	50.2	1,526.87
ELF4-HFC Col rep 1	| Symbols: PHYD | phytochrome D | chr4:9195602-9199486 REVERSE LENGTH=1164	AT4G16250.1	TAIR10_20101214.fasta	129,270.30	100.00%	1	1	12	0.06%	11.00%	TEDPALSIAGAVQSQK	99.70%	108	29.4	60	1,739.88
ELF4-HFC Col rep 1	| Symbols: PHYD | phytochrome D | chr4:9195602-9199486 REVERSE LENGTH=1164	AT4G16250.1	TAIR10_20101214.fasta	129,270.30	100.00%	1	1	12	0.06%	11.00%	WTSPEGLGLSVCR	99.70%	47.6	29.8	1.74	2,459.28
ELF4-HFC Col rep 1	| Symbols: PHYE | phytochrome E | chr4:10042312-10045948 REVERSE LENGTH=1112	AT4G18130.1	TAIR10_20101214.fasta	122,519.10	100.00%	17	18	20	0.10%	25.20%	AALESLLCK	99.70%	48.8	30.1	28.5	1,052.57
ELF4-HFC Col rep 1	| Symbols: PHYE | phytochrome E | chr4:10042312-10045948 REVERSE LENGTH=1112	AT4G18130.1	TAIR10_20101214.fasta	122,519.10	100.00%	17	18	20	0.10%	25.20%	AASFTEISLLNPVLVHSR	99.70%	53.6	27.2	111	1,600.93
ELF4-HFC Col rep 1	| Symbols: PHYE | phytochrome E | chr4:10042312-10045948 REVERSE LENGTH=1112	AT4G18130.1	TAIR10_20101214.fasta	122,519.10	100.00%	17	18	20	0.10%	25.20%	DGWVTPDGLGLK	99.40%	38.8	28.2	55.9	1,926.04
ELF4-HFC Col rep 1	| Symbols: PHYE | phytochrome E | chr4:10042312-10045948 REVERSE LENGTH=1112	AT4G18130.1	TAIR10_20101214.fasta	122,519.10	100.00%	17	18	20	0.10%	25.20%	ESAQSLNELTYVR	99.70%	54.8	30.6	39.7	1,408.71
ELF4-HFC Col rep 1	| Symbols: PHYE | phytochrome E | chr4:10042312-10045948 REVERSE LENGTH=1112	AT4G18130.1	TAIR10_20101214.fasta	122,519.10	100.00%	17	18	20	0.10%	25.20%	ESFTSSRPVLSGNGVAR	99.70%	45.6	31.6	75.2	1,445.80
ELF4-HFC Col rep 1	| Symbols: PHYE | phytochrome E | chr4:10042312-10045948 REVERSE LENGTH=1112	AT4G18130.1	TAIR10_20101214.fasta	122,519.10	100.00%	17	18	20	0.10%	25.20%	FGQNNHPDYSSDVCVLVNSCTSR	99.50%	37.7	27.4	64.2	1,607.75
ELF4-HFC Col rep 1	| Symbols: PHYE | phytochrome E | chr4:10042312-10045948 REVERSE LENGTH=1112	AT4G18130.1	TAIR10_20101214.fasta	122,519.10	100.00%	17	18	20	0.10%	25.20%	GGLVQPFGCLIAVEEPSFR	99.70%	73.5	31.2	35	1,405.76
ELF4-HFC Col rep 1	| Symbols: PHYE | phytochrome E | chr4:10042312-10045948 REVERSE LENGTH=1112	AT4G18130.1	TAIR10_20101214.fasta	122,519.10	100.00%	17	18	20	0.10%	25.20%	LLESSEISASQR	99.70%	68.5	30.5	42.1	1,226.67
ELF4-HFC Col rep 1	| Symbols: PHYE | phytochrome E | chr4:10042312-10045948 REVERSE LENGTH=1112	AT4G18130.1	TAIR10_20101214.fasta	122,519.10	100.00%	17	18	20	0.10%	25.20%	LQSLPGGDIGALCDTVVEDVQR	99.70%	106	31.8	50.4	1,045.59
ELF4-HFC Col rep 1	| Symbols: PHYE | phytochrome E | chr4:10042312-10045948 REVERSE LENGTH=1112	AT4G18130.1	TAIR10_20101214.fasta	122,519.10	100.00%	17	18	20	0.10%	25.20%	NIVNHAPFPNSWVGISISPGQELSR	96.20%	34.6	31	61.9	1,916.96
ELF4-HFC Col rep 1	| Symbols: PHYE | phytochrome E | chr4:10042312-10045948 REVERSE LENGTH=1112	AT4G18130.1	TAIR10_20101214.fasta	122,519.10	100.00%	17	18	20	0.10%	25.20%	QITTIIESTDLK	99.70%	46.7	28.6	50.8	1,164.60
ELF4-HFC Col rep 1	| Symbols: PHYE | phytochrome E | chr4:10042312-10045948 REVERSE LENGTH=1112	AT4G18130.1	TAIR10_20101214.fasta	122,519.10	100.00%	17	18	20	0.10%	25.20%	SGDPALTLAGAVQSQK	99.70%	61.3	29.3	90	1,612.72
ELF4-HFC Col rep 1	| Symbols: PHYE | phytochrome E | chr4:10042312-10045948 REVERSE LENGTH=1112	AT4G18130.1	TAIR10_20101214.fasta	122,519.10	100.00%	17	18	20	0.10%	25.20%	SLADEIVQEESR	99.70%	86.8	29.4	42.8	1,097.56
ELF4-HFC Col rep 1	| Symbols: PHYE | phytochrome E | chr4:10042312-10045948 REVERSE LENGTH=1112	AT4G18130.1	TAIR10_20101214.fasta	122,519.10	100.00%	17	18	20	0.10%	25.20%	SNTAQYSVDAALFADFAQSIYTGK	99.70%	82.2	31.8	61.2	1,388.68
ELF4-HFC Col rep 1	| Symbols: PHYE | phytochrome E | chr4:10042312-10045948 REVERSE LENGTH=1112	AT4G18130.1	TAIR10_20101214.fasta	122,519.10	100.00%	17	18	20	0.10%	25.20%	TLFTPSSGASLSK	99.70%	46.3	28.9	34.4	1,290.67
ELF4-HFC Col rep 1	| Symbols: PHYE | phytochrome E | chr4:10042312-10045948 REVERSE LENGTH=1112	AT4G18130.1	TAIR10_20101214.fasta	122,519.10	100.00%	17	18	20	0.10%	25.20%	VIETATAPIFGVDSSGCINGWNK	99.70%	69.9	31.3	0.7	2,371.16
ELF4-HFC Col rep 1	| Symbols: PHYE | phytochrome E | chr4:10042312-10045948 REVERSE LENGTH=1112	AT4G18130.1	TAIR10_20101214.fasta	122,519.10	100.00%	17	18	20	0.10%	25.20%	YIEASLTANK	99.70%	51.9	28.8	50.7	1,293.64
ELF4-HFC Col rep 1	| Symbols: SPA1 | SPA (suppressor of phyA-105) protein family | chr2:19022572-19026821 REVERSE LENGTH=1029	AT2G46340.1	TAIR10_20101214.fasta	114,689.10	100.00%	3	3	4	0.02%	4.28%	NDLESDVDEDLNR	99.70%	58.7	25.4	0	2,527.26
ELF4-HFC Col rep 1	| Symbols: SPA1 | SPA (suppressor of phyA-105) protein family | chr2:19022572-19026821 REVERSE LENGTH=1029	AT2G46340.1	TAIR10_20101214.fasta	114,689.10	100.00%	3	3	4	0.02%	4.28%	NQETPPEFVSDQDLGSK	99.70%	66.5	29.2	31.4	1,074.51
ELF4-HFC Col rep 1	| Symbols: SPA1 | SPA (suppressor of phyA-105) protein family | chr2:19022572-19026821 REVERSE LENGTH=1029	AT2G46340.1	TAIR10_20101214.fasta	114,689.10	100.00%	3	3	4	0.02%	4.28%	TPVSSSNFSQLLLK	99.70%	66.4	28.6	94	1,614.83
ELF4-HFC Col rep 1	| Symbols: TIC | time for coffee | chr3:7913181-7918879 FORWARD LENGTH=1550	AT3G22380.1,AT3G22380.2	TAIR10_20101214.fasta	164,452.80	100.00%	2	2	4	0.02%	2.06%	DSPEEEGPVELPEAAR	99.70%	46.3	29.2	37.7	1,461.72
ELF4-HFC Col rep 1	| Symbols: TIC | time for coffee | chr3:7913181-7918879 FORWARD LENGTH=1550	AT3G22380.1,AT3G22380.2	TAIR10_20101214.fasta	164,452.80	100.00%	2	2	4	0.02%	2.06%	SSPSPAPVSPLVSTWK	99.70%	49.1	28.7	32.7	1,004.54
ELF4-HFC Col rep 1	| Symbols: TZP | zinc knuckle (CCHC-type) family protein | chr5:17527261-17530207 FORWARD LENGTH=831	AT5G43630.1	TAIR10_20101214.fasta	90,615.60	100.00%	16	17	20	0.10%	22.50%	AVFDAVQVLR	99.70%	70.9	28.4	50.9	1,954.08
ELF4-HFC Col rep 1	| Symbols: TZP | zinc knuckle (CCHC-type) family protein | chr5:17527261-17530207 FORWARD LENGTH=831	AT5G43630.1	TAIR10_20101214.fasta	90,615.60	100.00%	16	17	20	0.10%	22.50%	AWWQSAGK	99.00%	41.4	28.8	17.1	1,257.65
ELF4-HFC Col rep 1	| Symbols: TZP | zinc knuckle (CCHC-type) family protein | chr5:17527261-17530207 FORWARD LENGTH=831	AT5G43630.1	TAIR10_20101214.fasta	90,615.60	100.00%	16	17	20	0.10%	22.50%	CFQLSHWAATCPNAPLYGSGAEGR	99.70%	44.4	29.5	52.5	1,509.75
ELF4-HFC Col rep 1	| Symbols: TZP | zinc knuckle (CCHC-type) family protein | chr5:17527261-17530207 FORWARD LENGTH=831	AT5G43630.1	TAIR10_20101214.fasta	90,615.60	100.00%	16	17	20	0.10%	22.50%	DCLEVTDTELR	99.70%	52.5	28.4	38.7	1,764.89

ELF4-HFC Col rep 1	Symbols: TZP | zinc knuckle (CCHC-type) family protein | chr5:17527261-17530207 FORWARD LENGTH=831	AT5G43630.1	TAIR10_20101214.fasta	90,615.60	100.00%	16	17	20	0.10%	22.50%	DLLVNEQLR	99.70%	44	26.1	1.16	2,657.14
ELF4-HFC Col rep 1	Symbols: TZP | zinc knuckle (CCHC-type) family protein | chr5:17527261-17530207 FORWARD LENGTH=831	AT5G43630.1	TAIR10_20101214.fasta	90,615.60	100.00%	16	17	20	0.10%	22.50%	EANASASDAAK	99.70%	46	26.9	63.2	2,076.06
ELF4-HFC Col rep 1	Symbols: TZP | zinc knuckle (CCHC-type) family protein | chr5:17527261-17530207 FORWARD LENGTH=831	AT5G43630.1	TAIR10_20101214.fasta	90,615.60	100.00%	16	17	20	0.10%	22.50%	EEASSLCIR	99.70%	45.4	28.9	58.5	1,319.68
ELF4-HFC Col rep 1	Symbols: TZP | zinc knuckle (CCHC-type) family protein | chr5:17527261-17530207 FORWARD LENGTH=831	AT5G43630.1	TAIR10_20101214.fasta	90,615.60	100.00%	16	17	20	0.10%	22.50%	GDDLSSSGNEIGPVAEPNISSGK	99.70%	89.4	30.5	104	2,342.17
ELF4-HFC Col rep 1	Symbols: TZP | zinc knuckle (CCHC-type) family protein | chr5:17527261-17530207 FORWARD LENGTH=831	AT5G43630.1	TAIR10_20101214.fasta	90,615.60	100.00%	16	17	20	0.10%	22.50%	GDYLPEGEAGPSGSYR	99.70%	53.6	27	5.99	2,720.38
ELF4-HFC Col rep 1	Symbols: TZP | zinc knuckle (CCHC-type) family protein | chr5:17527261-17530207 FORWARD LENGTH=831	AT5G43630.1	TAIR10_20101214.fasta	90,615.60	100.00%	16	17	20	0.10%	22.50%	KRPGFEEQLIFGSK	99.70%	40.4	28.8	32.4	1,361.75
ELF4-HFC Col rep 1	Symbols: TZP | zinc knuckle (CCHC-type) family protein | chr5:17527261-17530207 FORWARD LENGTH=831	AT5G43630.1	TAIR10_20101214.fasta	90,615.60	100.00%	16	17	20	0.10%	22.50%	LPISGFTDVPR	99.70%	71	29.4	53.5	1,542.81
ELF4-HFC Col rep 1	Symbols: TZP | zinc knuckle (CCHC-type) family protein | chr5:17527261-17530207 FORWARD LENGTH=831	AT5G43630.1	TAIR10_20101214.fasta	90,615.60	100.00%	16	17	20	0.10%	22.50%	NGREEASSLCIR	99.60%	38.3	28.6	69.1	1,375.67
ELF4-HFC Col rep 1	Symbols: TZP | zinc knuckle (CCHC-type) family protein | chr5:17527261-17530207 FORWARD LENGTH=831	AT5G43630.1	TAIR10_20101214.fasta	90,615.60	100.00%	16	17	20	0.10%	22.50%	RPGFEEQLIFGSK	99.70%	41.2	30	80.2	2,568.23
ELF4-HFC Col rep 1	Symbols: TZP | zinc knuckle (CCHC-type) family protein | chr5:17527261-17530207 FORWARD LENGTH=831	AT5G43630.1	TAIR10_20101214.fasta	90,615.60	100.00%	16	17	20	0.10%	22.50%	SVSGLEGFFLR	99.70%	57.3	27.8	29.3	1,295.68
ELF4-HFC Col rep 1	Symbols: TZP | zinc knuckle (CCHC-type) family protein | chr5:17527261-17530207 FORWARD LENGTH=831	AT5G43630.1	TAIR10_20101214.fasta	90,615.60	100.00%	16	17	20	0.10%	22.50%	TLNQECLGSTSK	99.70%	58.8	29.5	16.9	2,437.17
ELF4-HFC Col rep 1	Symbols: TZP | zinc knuckle (CCHC-type) family protein | chr5:17527261-17530207 FORWARD LENGTH=831	AT5G43630.1	TAIR10_20101214.fasta	90,615.60	100.00%	16	17	20	0.10%	22.50%	TSGYDGIPSAEELSR	99.70%	64.2	29.8	34.9	1,109.58
ELF4-HFC Col rep 2	Symbols: | F-box family protein | chr2:7074940-7079181 FORWARD LENGTH=727	AT2G16365.4	TAIR10_20101214.fasta	82,716.20	100.00%	14	15	17	0.08%	27.20%	ALSGLNPYGLR	99.70%	54.7	30.4	50.4	1,533.67
ELF4-HFC Col rep 2	Symbols: | F-box family protein | chr2:7074940-7079181 FORWARD LENGTH=727	AT2G16365.4	TAIR10_20101214.fasta	82,716.20	100.00%	14	15	17	0.08%	27.20%	ENNNLFLEILK	99.70%	53.5	29.6	61.6	1,890.87
ELF4-HFC Col rep 2	Symbols: | F-box family protein | chr2:7074940-7079181 FORWARD LENGTH=727	AT2G16365.4	TAIR10_20101214.fasta	82,716.20	100.00%	14	15	17	0.08%	27.20%	ETTTTTTTKPSFHEDVGSSSR	99.70%	84.3	30.4	52.1	1,520.83
ELF4-HFC Col rep 2	Symbols: | F-box family protein | chr2:7074940-7079181 FORWARD LENGTH=727	AT2G16365.4	TAIR10_20101214.fasta	82,716.20	100.00%	14	15	17	0.08%	27.20%	FHDGETNCSK	99.70%	36.2	25	40	1,724.80
ELF4-HFC Col rep 2	Symbols: | F-box family protein | chr2:7074940-7079181 FORWARD LENGTH=727	AT2G16365.4	TAIR10_20101214.fasta	82,716.20	100.00%	14	15	17	0.08%	27.20%	IFGGYLDLFPNQDHSHNR	99.70%	41.7	30.8	39.5	1,639.87
ELF4-HFC Col rep 2	Symbols: | F-box family protein | chr2:7074940-7079181 FORWARD LENGTH=727	AT2G16365.4	TAIR10_20101214.fasta	82,716.20	100.00%	14	15	17	0.08%	27.20%	IYATVDSVNLPK	99.70%	77.3	29	54.6	1,117.64
ELF4-HFC Col rep 2	Symbols: | F-box family protein | chr2:7074940-7079181 FORWARD LENGTH=727	AT2G16365.4	TAIR10_20101214.fasta	82,716.20	100.00%	14	15	17	0.08%	27.20%	LDFPIQEK	99.00%	39.5	30.2	20.6	933.4577
ELF4-HFC Col rep 2	Symbols: | F-box family protein | chr2:7074940-7079181 FORWARD LENGTH=727	AT2G16365.4	TAIR10_20101214.fasta	82,716.20	100.00%	14	15	17	0.08%	27.20%	LQTIHLSGSISSSSTK	99.70%	47.5	30.4	33.8	2,650.19
ELF4-HFC Col rep 2	Symbols: | F-box family protein | chr2:7074940-7079181 FORWARD LENGTH=727	AT2G16365.4	TAIR10_20101214.fasta	82,716.20	100.00%	14	15	17	0.08%	27.20%	NQEPVVSQSNDLR	99.70%	57.3	29.3	37	1,350.62
ELF4-HFC Col rep 2	Symbols: | F-box family protein | chr2:7074940-7079181 FORWARD LENGTH=727	AT2G16365.4	TAIR10_20101214.fasta	82,716.20	100.00%	14	15	17	0.08%	27.20%	NSSVNPYESAWLGR	99.70%	85.4	30.2	21.1	1,099.61
ELF4-HFC Col rep 2	Symbols: | F-box family protein | chr2:7074940-7079181 FORWARD LENGTH=727	AT2G16365.4	TAIR10_20101214.fasta	82,716.20	100.00%	14	15	17	0.08%	27.20%	QGGDDITLLHPWIQR	99.10%	29.7	30.6	44.9	1,034.47
ELF4-HFC Col rep 2	Symbols: | F-box family protein | chr2:7074940-7079181 FORWARD LENGTH=727	AT2G16365.4	TAIR10_20101214.fasta	82,716.20	100.00%	14	15	17	0.08%	27.20%	TTQNLLELIRPVR	99.70%	37.5	25	31.2	1,064.50
ELF4-HFC Col rep 2	Symbols: | F-box family protein | chr2:7074940-7079181 FORWARD LENGTH=727	AT2G16365.4	TAIR10_20101214.fasta	82,716.20	100.00%	14	15	17	0.08%	27.20%	TTSTDQPTGQEASFEPESHKEFEK	99.70%	52.4	28.9	87.7	2,230.05
ELF4-HFC Col rep 2	Symbols: | F-box family protein | chr2:7074940-7079181 FORWARD LENGTH=727	AT2G16365.4	TAIR10_20101214.fasta	82,716.20	100.00%	14	15	17	0.08%	27.20%	VRLEPEVEASSR	99.70%	58.6	29.4	42.2	1,654.73
ELF4-HFC Col rep 2	Symbols: | Granulin repeat cysteine protease family protein | chr3:6723024-6724768 FORWARD LENGTH=452	AT3G19390.1	TAIR10_20101214.fasta	49,308.20	100.00%	2	2	2	0.01%	7.08%	ALANQPISVAIEAGGR	99.70%	76.8	27.3	38.9	1,635.89
ELF4-HFC Col rep 2	Symbols: | Granulin repeat cysteine protease family protein | chr3:6723024-6724768 FORWARD LENGTH=452	AT3G19390.1	TAIR10_20101214.fasta	49,308.20	100.00%	2	2	2	0.01%	7.08%	VVTIDGYEDVPQNDEK	99.70%	51.7	29.9	62.8	1,201.66
ELF4-HFC Col rep 2	Symbols: | Protein kinase family protein | chr3:4469434-4473234 FORWARD LENGTH=703	AT3G13670.1	TAIR10_20101214.fasta	78,640.00	99.60%	1	1	2	0.01%	6.40%	GYVHGDVKPENFLLGQPSTSQEK	98.60%	31.5	31.6	12.9	1,392.65
ELF4-HFC Col rep 2	Symbols: | Protein kinase family protein | chr3:4469434-4473234 FORWARD LENGTH=703	AT3G13670.1	TAIR10_20101214.fasta	78,640.00	99.60%	1	1	2	0.01%	6.40%	NSGYSEQVVELDFLYPSEGIHR	99.70%	58.2	31.5	32.7	1,507.79
ELF4-HFC Col rep 2	Symbols: COP1, ATCOP1, DET340, FUS1, EMB168 | Transducin/WD40 repeat-like superfamily protein | chr2:13978000-13983282 FORWARD LENGTH=675	AT2G32950.1	TAIR10_20101214.fasta	76,188.00	100.00%	2	2	2	0.01%	4.44%	FLSNNELASASTDSTLR	99.70%	94.5	30.6	36.3	1,211.64
ELF4-HFC Col rep 2	Symbols: COP1, ATCOP1, DET340, FUS1, EMB168 | Transducin/WD40 repeat-like superfamily protein | chr2:13978000-13983282 FORWARD LENGTH=675	AT2G32950.1	TAIR10_20101214.fasta	76,188.00	100.00%	2	2	2	0.01%	4.44%	NQIGFNSNSLSIR	99.70%	46.6	30.6	53.1	1,337.64
ELF4-HFC Col rep 2	Symbols: ELF3, PYK20 | hydroxyproline-rich glycoprotein family protein | chr2:11059459-11063178 FORWARD LENGTH=695	AT2G25930.1	TAIR10_20101214.fasta	77,204.80	100.00%	18	24	28	0.14%	35.00%	EEEDFAVPVYINSR	99.70%	46.4	29.2	37	1,581.74
ELF4-HFC Col rep 2	Symbols: ELF3, PYK20 | hydroxyproline-rich glycoprotein family protein | chr2:11059459-11063178 FORWARD LENGTH=695	AT2G25930.1	TAIR10_20101214.fasta	77,204.80	100.00%	18	24	28	0.14%	35.00%	FQEVNQTGSK	99.70%	57.3	29.3	40.1	1,160.64
ELF4-HFC Col rep 2	Symbols: ELF3, PYK20 | hydroxyproline-rich glycoprotein family protein | chr2:11059459-11063178 FORWARD LENGTH=695	AT2G25930.1	TAIR10_20101214.fasta	77,204.80	100.00%	18	24	28	0.14%	35.00%	IFQSIQEER	99.70%	46.1	30.4	37.7	1,346.73
ELF4-HFC Col rep 2	Symbols: ELF3, PYK20 | hydroxyproline-rich glycoprotein family protein | chr2:11059459-11063178 FORWARD LENGTH=695	AT2G25930.1	TAIR10_20101214.fasta	77,204.80	100.00%	18	24	28	0.14%	35.00%	KAIANQQR	97.20%	26.4	25.1	75.6	2,269.06
ELF4-HFC Col rep 2	Symbols: ELF3, PYK20 | hydroxyproline-rich glycoprotein family protein | chr2:11059459-11063178 FORWARD LENGTH=695	AT2G25930.1	TAIR10_20101214.fasta	77,204.80	100.00%	18	24	28	0.14%	35.00%	LASENAAR	99.00%	48.9	28.7	32.3	1,194.48
ELF4-HFC Col rep 2	Symbols: ELF3, PYK20 | hydroxyproline-rich glycoprotein family protein | chr2:11059459-11063178 FORWARD LENGTH=695	AT2G25930.1	TAIR10_20101214.fasta	77,204.80	100.00%	18	24	28	0.14%	35.00%	LHVNDADK	99.00%	37	26.8	24.4	2,130.02
ELF4-HFC Col rep 2	Symbols: ELF3, PYK20 | hydroxyproline-rich glycoprotein family protein | chr2:11059459-11063178 FORWARD LENGTH=695	AT2G25930.1	TAIR10_20101214.fasta	77,204.80	100.00%	18	24	28	0.14%	35.00%	LIAASPDLLLDEISFLGK	99.70%	98.9	25.4	59.5	1,319.72
ELF4-HFC Col rep 2	Symbols: ELF3, PYK20 | hydroxyproline-rich glycoprotein family protein | chr2:11059459-11063178 FORWARD LENGTH=695	AT2G25930.1	TAIR10_20101214.fasta	77,204.80	100.00%	18	24	28	0.14%	35.00%	MALYEQLSIPSQR	99.70%	40	30.9	14.7	989.5304
ELF4-HFC Col rep 2	Symbols: ELF3, PYK20 | hydroxyproline-rich glycoprotein family protein | chr2:11059459-11063178 FORWARD LENGTH=695	AT2G25930.1	TAIR10_20101214.fasta	77,204.80	100.00%	18	24	28	0.14%	35.00%	MESSAENVVGR	99.70%	76.1	27.9	43.7	1,645.88
ELF4-HFC Col rep 2	Symbols: ELF3, PYK20 | hydroxyproline-rich glycoprotein family protein | chr2:11059459-11063178 FORWARD LENGTH=695	AT2G25930.1	TAIR10_20101214.fasta	77,204.80	100.00%	18	24	28	0.14%	35.00%	NLSVQHLDSSAANQATEK	99.70%	101	31	42.6	1,485.73
ELF4-HFC Col rep 2	Symbols: ELF3, PYK20 | hydroxyproline-rich glycoprotein family protein | chr2:11059459-11063178 FORWARD LENGTH=695	AT2G25930.1	TAIR10_20101214.fasta	77,204.80	100.00%	18	24	28	0.14%	35.00%	QGSTGSSPSGPQGISGSK	99.70%	103	29.1	72.8	1,579.75
ELF4-HFC Col rep 2	Symbols: ELF3, PYK20 | hydroxyproline-rich glycoprotein family protein | chr2:11059459-11063178 FORWARD LENGTH=695	AT2G25930.1	TAIR10_20101214.fasta	77,204.80	100.00%	18	24	28	0.14%	35.00%	QNVCLATCSKPEVR	99.70%	60.9	30.2	21	1,748.91
ELF4-HFC Col rep 2	Symbols: ELF3, PYK20 | hydroxyproline-rich glycoprotein family protein | chr2:11059459-11063178 FORWARD LENGTH=695	AT2G25930.1	TAIR10_20101214.fasta	77,204.80	100.00%	18	24	28	0.14%	35.00%	SDNEPAPQQQQQPTK	99.70%	70	29.6	26.6	1,552.92
ELF4-HFC Col rep 2	Symbols: ELF3, PYK20 | hydroxyproline-rich glycoprotein family protein | chr2:11059459-11063178 FORWARD LENGTH=695	AT2G25930.1	TAIR10_20101214.fasta	77,204.80	100.00%	18	24	28	0.14%	35.00%	SFRPFAAVDEDSNINNAPEQTMTTTTTTTR	99.70%	56.5	30.9	42.5	2,710.21
ELF4-HFC Col rep 2	Symbols: ELF3, PYK20 | hydroxyproline-rich glycoprotein family protein | chr2:11059459-11063178 FORWARD LENGTH=695	AT2G25930.1	TAIR10_20101214.fasta	77,204.80	100.00%	18	24	28	0.14%	35.00%	SNNTSTLVHPGPSSQPCGVER	99.70%	45.5	30.3	48.6	1,371.72
ELF4-HFC Col rep 2	Symbols: ELF3, PYK20 | hydroxyproline-rich glycoprotein family protein | chr2:11059459-11063178 FORWARD LENGTH=695	AT2G25930.1	TAIR10_20101214.fasta	77,204.80	100.00%	18	24	28	0.14%	35.00%	TTVTQTTR	99.00%	32.6	28.3	72.9	1,566.86
ELF4-HFC Col rep 2	Symbols: ELF3, PYK20 | hydroxyproline-rich glycoprotein family protein | chr2:11059459-11063178 FORWARD LENGTH=695	AT2G25930.1	TAIR10_20101214.fasta	77,204.80	100.00%	18	24	28	0.14%	35.00%	VFAVQLFELHR	99.70%	31.6	28.4	44.7	1,820.86
ELF4-HFC Col rep 2	Symbols: ELF3, PYK20 | hydroxyproline-rich glycoprotein family protein | chr2:11059459-11063178 FORWARD LENGTH=695	AT2G25930.1	TAIR10_20101214.fasta	77,204.80	100.00%	18	24	28	0.14%	35.00%	VNDYNASLR	99.70%	72.2	29.2	13.7	2,530.26
ELF4-HFC Col rep 2	Symbols: ELF4 | Protein of unknown function (DUF1313) | chr2:16734545-16734880 REVERSE LENGTH=111	AT2G40080.1	TAIR10_20101214.fasta	12,375.40	100.00%	8	27	165	0.81%	79.30%	NGHDGGGAAGTR	99.70%	44.7	25	48.5	2,539.21
ELF4-HFC Col rep 2	Symbols: ELF4 | Protein of unknown function (DUF1313) | chr2:16734545-16734880 REVERSE LENGTH=111	AT2G40080.1	TAIR10_20101214.fasta	12,375.40	100.00%	8	27	165	0.81%	79.30%	NVAEEAEQGEDPAMWENLDR	99.70%	103	25.8	93.3	1,825.89
ELF4-HFC Col rep 2	Symbols: ELF4 | Protein of unknown function (DUF1313) | chr2:16734545-16734880 REVERSE LENGTH=111	AT2G40080.1	TAIR10_20101214.fasta	12,375.40	100.00%	8	27	165	0.81%	79.30%	NVALIQELNGNISK	99.70%	133	28.7	36.4	1,449.74
ELF4-HFC Col rep 2	Symbols: ELF4 | Protein of unknown function (DUF1313) | chr2:16734545-16734880 REVERSE LENGTH=111	AT2G40080.1	TAIR10_20101214.fasta	12,375.40	100.00%	8	27	165	0.81%	79.30%	QVQSVLDR	99.00%	38.8	29.2	40.7	1,668.78
ELF4-HFC Col rep 2	Symbols: ELF4 | Protein of unknown function (DUF1313) | chr2:16734545-16734880 REVERSE LENGTH=111	AT2G40080.1	TAIR10_20101214.fasta	12,375.40	100.00%	8	27	165	0.81%	79.30%	RNVAEEAEQGEDPAMWENLDR	99.70%	85.2	28.1	33.2	1,137.55
ELF4-HFC Col rep 2	Symbols: ELF4 | Protein of unknown function (DUF1313) | chr2:16734545-16734880 REVERSE LENGTH=111	AT2G40080.1	TAIR10_20101214.fasta	12,375.40	100.00%	8	27	165	0.81%	79.30%	SLIQQVNDNHQSR	99.70%	79.2	30.1	20.2	1,149.59
ELF4-HFC Col rep 2	Symbols: ELF4 | Protein of unknown function (DUF1313) | chr2:16734545-16734880 REVERSE LENGTH=111	AT2G40080.1	TAIR10_20101214.fasta	12,375.40	100.00%	8	27	165	0.81%	79.30%	VVNMYSDLNTSFSSGFHGGK	99.70%	107	29.4	7.13	928.5323
ELF4-HFC Col rep 2	Symbols: ELF4 | Protein of unknown function (DUF1313) | chr2:16734545-16734880 REVERSE LENGTH=111	AT2G40080.1	TAIR10_20101214.fasta	12,375.40	100.00%	8	27	165	0.81%	79.30%	VVNMYSDLNTSFSSGFHGGKNGHDGGGAAGTR	99.70%	33.3	29	27.6	831.4318
ELF4-HFC Col rep 2	Symbols: PCL1, LUX | Homeodomain-like superfamily protein | chr3:17183248-17184219 FORWARD LENGTH=323	AT3G46640.1,AT3G46640.2	TAIR10_20101214.fasta	34,648.50	100.00%	3	5	7	0.03%	18.30%	ASETTLSSLR	99.70%	91	29.7	24.4	911.4582
ELF4-HFC Col rep 2	Symbols: PCL1, LUX | Homeodomain-like superfamily protein | chr3:17183248-17184219 FORWARD LENGTH=323	AT3G46640.1,AT3G46640.2	TAIR10_20101214.fasta	34,648.50	100.00%	3	5	7	0.03%	18.30%	ENVASHLQK	99.50%	36.4	28.1	85.8	1,915.08
ELF4-HFC Col rep 2	Symbols: PCL1, LUX | Homeodomain-like superfamily protein | chr3:17183248-17184219 FORWARD LENGTH=323	AT3G46640.1,AT3G46640.2	TAIR10_20101214.fasta	34,648.50	100.00%	3	5	7	0.03%	18.30%	FVDVVAHLGIK	99.70%	47.8	25	37.1	1,551.78
ELF4-HFC Col rep 2	Symbols: PCL1, LUX | Homeodomain-like superfamily protein | chr3:17183248-17184219 FORWARD LENGTH=323	AT3G46640.1,AT3G46640.2	TAIR10_20101214.fasta	34,648.50	100.00%	3	5	7	0.03%	18.30%	GGSSGPNTSSSNNNVEEEDR	99.70%	87	25	65.8	1,178.55
ELF4-HFC Col rep 2	Symbols: PCL1, LUX | Homeodomain-like superfamily protein | chr3:17183248-17184219 FORWARD LENGTH=323	AT3G46640.1,AT3G46640.2	TAIR10_20101214.fasta	34,648.50	100.00%	3	5	7	0.03%	18.30%	LVWTPQLHK	99.70%	34.9	25.4	92.3	1,912.94
ELF4-HFC Col rep 2	Symbols: PHYA, FHY2, FRE1, HY8 | phytochrome A | chr1:3095498-3099216 REVERSE LENGTH=1122	AT1G09570.1	TAIR10_20101214.fasta	124,503.20	100.00%	6	7	7	0.03%	6.33%	ADAGPISLVVNACASR	99.70%	78.9	29.4	91.7	1,618.77
ELF4-HFC Col rep 2	Symbols: PHYA, FHY2, FRE1, HY8 | phytochrome A | chr1:3095498-3099216 REVERSE LENGTH=1122	AT1G09570.1	TAIR10_20101214.fasta	124,503.20	100.00%	6	7	7	0.03%	6.33%	IIAQTTVDAK	99.70%	41	29	51	1,661.81
ELF4-HFC Col rep 2	Symbols: PHYA, FHY2, FRE1, HY8 | phytochrome A | chr1:3095498-3099216 REVERSE LENGTH=1122	AT1G09570.1	TAIR10_20101214.fasta	124,503.20	100.00%	6	7	7	0.03%	6.33%	SLFTAPSASALQK	99.70%	49.2	29.3	59.2	1,695.79
ELF4-HFC Col rep 2	Symbols: PHYA, FHY2, FRE1, HY8 | phytochrome A | chr1:3095498-3099216 REVERSE LENGTH=1122	AT1G09570.1	TAIR10_20101214.fasta	124,503.20	100.00%	6	7	7	0.03%	6.33%	SSFIITAELAAANK	99.70%	67.2	28.7	0.93	3,334.50
ELF4-HFC Col rep 2	Symbols: PHYA, FHY2, FRE1, HY8 | phytochrome A | chr1:3095498-3099216 REVERSE LENGTH=1122	AT1G09570.1	TAIR10_20101214.fasta	124,503.20	100.00%	6	7	7	0.03%	6.33%	VSFAFFTR	99.00%	51.6	29.3	43.8	2,224.04
ELF4-HFC Col rep 2	Symbols: PHYA, FHY2, FRE1, HY8 | phytochrome A | chr1:3095498-3099216 REVERSE LENGTH=1122	AT1G09570.1	TAIR10_20101214.fasta	124,503.20	100.00%	6	7	7	0.03%	6.33%	YVECLLCVSK	99.70%	41	28.4	23.2	907.4844
ELF4-HFC Col rep 2	Symbols: PHYB, HY3, OOP1 | phytochrome B | chr2:8140079-8144151 FORWARD LENGTH=1172	AT2G18790.1	TAIR10_20101214.fasta	129,333.90	100.00%	27	41	70	0.34%	43.00%	AIQQYTVDAR	99.70%	58.3	30	17.2	1,358.76
ELF4-HFC Col rep 2	Symbols: PHYB, HY3, OOP1 | phytochrome B | chr2:8140079-8144151 FORWARD LENGTH=1172	AT2G18790.1	TAIR10_20101214.fasta	129,333.90	100.00%	27	41	70	0.34%	43.00%	AISQLQALPGGDIK	99.70%	93.2	26.1	57.5	1,051.52
ELF4-HFC Col rep 2	Symbols: PHYB, HY3, OOP1 | phytochrome B | chr2:8140079-8144151 FORWARD LENGTH=1172	AT2G18790.1	TAIR10_20101214.fasta	129,333.90	100.00%	27	41	70	0.34%	43.00%	AVFVVVNACSSK	99.70%	87	29.3	34.4	1,070.46
ELF4-HFC Col rep 2	Symbols: PHYB, HY3, OOP1 | phytochrome B | chr2:8140079-8144151 FORWARD LENGTH=1172	AT2G18790.1	TAIR10_20101214.fasta	129,333.90	100.00%	27	41	70	0.34%	43.00%	CDGAAFLYHGK	99.70%	65.3	27.6	103	2,318.98
ELF4-HFC Col rep 2	Symbols: PHYB, HY3, OOP1 | phytochrome B | chr2:8140079-8144151 FORWARD LENGTH=1172	AT2G18790.1	TAIR10_20101214.fasta	129,333.90	100.00%	27	41	70	0.34%	43.00%	DYLNNIVGVCFVGQDVTSQK	99.70%	96.3	31.4	39.9	1,513.82
ELF4-HFC Col rep 2	Symbols: PHYB, HY3, OOP1 | phytochrome B | chr2:8140079-8144151 FORWARD LENGTH=1172	AT2G18790.1	TAIR10_20101214.fasta	129,333.90	100.00%	27	41	70	0.34%	43.00%	EITLLNPVWIHSK	99.70%	61.3	26.7	0	944.516
ELF4-HFC Col rep 2	Symbols: PHYB, HY3, OOP1 | phytochrome B | chr2:8140079-8144151 FORWARD LENGTH=1172	AT2G18790.1	TAIR10_20101214.fasta	129,333.90	100.00%	27	41	70	0.34%	43.00%	ELAYICQVIK	99.70%	55.4	28.6	73.6	2,475.08
ELF4-HFC Col rep 2	Symbols: PHYB, HY3, OOP1 | phytochrome B | chr2:8140079-8144151 FORWARD LENGTH=1172	AT2G18790.1	TAIR10_20101214.fasta	129,333.90	100.00%	27	41	70	0.34%	43.00%	ENEATVNK	99.00%	34.7	27.4	15.4	1,539.75
ELF4-HFC Col rep 2	Symbols: PHYB, HY3, OOP1 | phytochrome B | chr2:8140079-8144151 FORWARD LENGTH=1172	AT2G18790.1	TAIR10_20101214.fasta	129,333.90	100.00%	27	41	70	0.34%	43.00%	FANSLLEATDLNEDQK	99.70%	117	30.2	107	2,146.99
ELF4-HFC Col rep 2	Symbols: PHYB, HY3, OOP1 | phytochrome B | chr2:8140079-8144151 FORWARD LENGTH=1172	AT2G18790.1	TAIR10_20101214.fasta	129,333.90	100.00%	27	41	70	0.34%	43.00%	FHEDEHGEVVAESK	99.70%	112	27.2	3.29	3,214.42
ELF4-HFC Col rep 2	Symbols: PHYB, HY3, OOP1 | phytochrome B | chr2:8140079-8144151 FORWARD LENGTH=1172	AT2G18790.1	TAIR10_20101214.fasta	129,333.90	100.00%	27	41	70	0.34%	43.00%	FINIQGDYK	99.70%	55.5	28	54.2	1,064.56
ELF4-HFC Col rep 2	Symbols: PHYB, HY3, OOP1 | phytochrome B | chr2:8140079-8144151 FORWARD LENGTH=1172	AT2G18790.1	TAIR10_20101214.fasta	129,333.90	100.00%	27	41	70	0.34%	43.00%	GGEEEPSSSHTPNNR	99.70%	61.7	25.2	19.5	1,025.54
ELF4-HFC Col rep 2	Symbols: PHYB, HY3, OOP1 | phytochrome B | chr2:8140079-8144151 FORWARD LENGTH=1172	AT2G18790.1	TAIR10_20101214.fasta	129,333.90	100.00%	27	41	70	0.34%	43.00%	HHPEDKDDGQR	97.30%	31	25	40.6	1,197.70
ELF4-HFC Col rep 2	Symbols: PHYB, HY3, OOP1 | phytochrome B | chr2:8140079-8144151 FORWARD LENGTH=1172	AT2G18790.1	TAIR10_20101214.fasta	129,333.90	100.00%	27	41	70	0.34%	43.00%	IAELTGLSVEEAMGK	99.70%	62.2	31.1	77	2,036.84
ELF4-HFC Col rep 2	Symbols: PHYB, HY3, OOP1 | phytochrome B | chr2:8140079-8144151 FORWARD LENGTH=1172	AT2G18790.1	TAIR10_20101214.fasta	129,333.90	100.00%	27	41	70	0.34%	43.00%	IDVGVVIDLEPAR	99.70%	77.3	27	26.6	1,121.65
ELF4-HFC Col rep 2	Symbols: PHYB, HY3, OOP1 | phytochrome B | chr2:8140079-8144151 FORWARD LENGTH=1172	AT2G18790.1	TAIR10_20101214.fasta	129,333.90	100.00%	27	41	70	0.34%	43.00%	IQQLLAEFLLSIIR	99.70%	108	25	66	1,600.81
ELF4-HFC Col rep 2	Symbols: PHYB, HY3, OOP1 | phytochrome B | chr2:8140079-8144151 FORWARD LENGTH=1172	AT2G18790.1	TAIR10_20101214.fasta	129,333.90	100.00%	27	41	70	0.34%	43.00%	IVGDMDLESIEDGSFVLK	99.70%	106	31.2	26.7	1,059.60
ELF4-HFC Col rep 2	Symbols: PHYB, HY3, OOP1 | phytochrome B | chr2:8140079-8144151 FORWARD LENGTH=1172	AT2G18790.1	TAIR10_20101214.fasta	129,333.90	100.00%	27	41	70	0.34%	43.00%	LHAVFEQSGESGK	99.70%	66.6	29.2	42.3	1,320.72
ELF4-HFC Col rep 2	Symbols: PHYB, HY3, OOP1 | phytochrome B | chr2:8140079-8144151 FORWARD LENGTH=1172	AT2G18790.1	TAIR10_20101214.fasta	129,333.90	100.00%	27	41	70	0.34%	43.00%	LIETATVPIFAVDAGGCINGWNAK	99.70%	69.1	31.1	57.6	1,435.78
ELF4-HFC Col rep 2	Symbols: PHYB, HY3, OOP1 | phytochrome B | chr2:8140079-8144151 FORWARD LENGTH=1172	AT2G18790.1	TAIR10_20101214.fasta	129,333.90	100.00%	27	41	70	0.34%	43.00%	LLCDTVVESVR	99.70%	72.2	30.6	31.5	974.5093
ELF4-HFC Col rep 2	Symbols: PHYB, HY3, OOP1 | phytochrome B | chr2:8140079-8144151 FORWARD LENGTH=1172	AT2G18790.1	TAIR10_20101214.fasta	129,333.90	100.00%	27	41	70	0.34%	43.00%	MIVDCNATPVLVVQDDR	99.70%	109	30.7	26.9	1,270.62
ELF4-HFC Col rep 2	Symbols: PHYB, HY3, OOP1 | phytochrome B | chr2:8140079-8144151 FORWARD LENGTH=1172	AT2G18790.1	TAIR10_20101214.fasta	129,333.90	100.00%	27	41	70	0.34%	43.00%	NTGKPFYAILHR	99.70%	69.9	29.3	50.5	1,164.60
ELF4-HFC Col rep 2	Symbols: PHYB, HY3, OOP1 | phytochrome B | chr2:8140079-8144151 FORWARD LENGTH=1172	AT2G18790.1	TAIR10_20101214.fasta	129,333.90	100.00%	27	41	70	0.34%	43.00%	QLLETSVSCEK	99.70%	59.4	30.6	83.2	1,410.80
ELF4-HFC Col rep 2	Symbols: PHYB, HY3, OOP1 | phytochrome B | chr2:8140079-8144151 FORWARD LENGTH=1172	AT2G18790.1	TAIR10_20101214.fasta	129,333.90	100.00%	27	41	70	0.34%	43.00%	RDDLEPYIGLHYPATDIPQASR	99.70%	39	31.3	75.4	1,280.67
ELF4-HFC Col rep 2	Symbols: PHYB, HY3, OOP1 | phytochrome B | chr2:8140079-8144151 FORWARD LENGTH=1172	AT2G18790.1	TAIR10_20101214.fasta	129,333.90	100.00%	27	41	70	0.34%	43.00%	RQDTECFTK	99.50%	32.1	26.1	43.5	1,238.56
ELF4-HFC Col rep 2	Symbols: PHYB, HY3, OOP1 | phytochrome B | chr2:8140079-8144151 FORWARD LENGTH=1172	AT2G18790.1	TAIR10_20101214.fasta	129,333.90	100.00%	27	41	70	0.34%	43.00%	SFDYSQSLK	99.70%	43.9	26.6	91.9	2,256.10
ELF4-HFC Col rep 2	Symbols: PHYB, HY3, OOP1 | phytochrome B | chr2:8140079-8144151 FORWARD LENGTH=1172	AT2G18790.1	TAIR10_20101214.fasta	129,333.90	100.00%	27	41	70	0.34%	43.00%	SIEVFGDQIR	99.70%	45.6	30.5	47.2	1,549.87
ELF4-HFC Col rep 2	Symbols: PHYB, HY3, OOP1 | phytochrome B | chr2:8140079-8144151 FORWARD LENGTH=1172	AT2G18790.1	TAIR10_20101214.fasta	129,333.90	100.00%	27	41	70	0.34%	43.00%	SLFTSSSSILLER	99.70%	76.3	28.2	43.5	1,236.67
ELF4-HFC Col rep 2	Symbols: PHYB, HY3, OOP1 | phytochrome B | chr2:8140079-8144151 FORWARD LENGTH=1172	AT2G18790.1	TAIR10_20101214.fasta	129,333.90	100.00%	27	41	70	0.34%	43.00%	SLVSDLIYK	99.70%	55.3	26.1	27	904.437
ELF4-HFC Col rep 2	Symbols: PHYB, HY3, OOP1 | phytochrome B | chr2:8140079-8144151 FORWARD LENGTH=1172	AT2G18790.1	TAIR10_20101214.fasta	129,333.90	100.00%	27	41	70	0.34%	43.00%	SSFQAFLEVVK	99.70%	62.8	26.8	79.9	1,808.86
ELF4-HFC Col rep 2	Symbols: PHYB, HY3, OOP1 | phytochrome B | chr2:8140079-8144151 FORWARD LENGTH=1172	AT2G18790.1	TAIR10_20101214.fasta	129,333.90	100.00%	27	41	70	0.34%	43.00%	TEDPALSIAGAVQSQK	99.70%	101	29.4	103	1,612.72
ELF4-HFC Col rep 2	Symbols: PHYB, HY3, OOP1 | phytochrome B | chr2:8140079-8144151 FORWARD LENGTH=1172	AT2G18790.1	TAIR10_20101214.fasta	129,333.90	100.00%	27	41	70	0.34%	43.00%	TTTYGSSVPEQQITAYLSR	99.70%	69.8	31.1	39.6	1,097.56
ELF4-HFC Col rep 2	Symbols: PHYB, HY3, OOP1 | phytochrome B | chr2:8140079-8144151 FORWARD LENGTH=1172	AT2G18790.1	TAIR10_20101214.fasta	129,333.90	100.00%	27	41	70	0.34%	43.00%	VIGAFCFLQIPSPELQQALAVQR	99.70%	89.3	28.7	47.2	1,597.68
ELF4-HFC Col rep 2	Symbols: PHYB, HY3, OOP1 | phytochrome B | chr2:8140079-8144151 FORWARD LENGTH=1172	AT2G18790.1	TAIR10_20101214.fasta	129,333.90	100.00%	27	41	70	0.34%	43.00%	VSGVGGSGGGR	99.70%	41.2	28.4	23	1,333.59
ELF4-HFC Col rep 2	Symbols: PHYB, HY3, OOP1 | phytochrome B | chr2:8140079-8144151 FORWARD LENGTH=1172	AT2G18790.1	TAIR10_20101214.fasta	129,333.90	100.00%	27	41	70	0.34%	43.00%	VVDGVVQPCR	98.10%	31.1	27.5	56.7	1,563.79
ELF4-HFC Col rep 2	Symbols: PHYB, HY3, OOP1 | phytochrome B | chr2:8140079-8144151 FORWARD LENGTH=1172	AT2G18790.1	TAIR10_20101214.fasta	129,333.90	100.00%	27	41	70	0.34%	43.00%	YAPSQEWVEIHLSQLSK	99.70%	60.2	30.7	65.6	1,395.78
ELF4-HFC Col rep 2	Symbols: PHYB, HY3, OOP1 | phytochrome B | chr2:8140079-8144151 FORWARD LENGTH=1172	AT2G18790.1	TAIR10_20101214.fasta	129,333.90	100.00%	27	41	70	0.34%	43.00%	YYPLGVAPSEVQIK	99.70%	63.4	29.6	104	1,657.00
ELF4-HFC Col rep 2	Symbols: PHYC | phytochrome C | chr5:14008049-14011619 FORWARD LENGTH=1111	AT5G35840.1	TAIR10_20101214.fasta	123,724.60	100.00%	27	34	41	0.20%	30.50%	AAEVTGLAVEQAIGKPVSDLVEDDSVETVK	99.70%	72.2	30.6	96.6	1,982.96
ELF4-HFC Col rep 2	Symbols: PHYC | phytochrome C | chr5:14008049-14011619 FORWARD LENGTH=1111	AT5G35840.1	TAIR10_20101214.fasta	123,724.60	100.00%	27	34	41	0.20%	30.50%	AISFLQDLLHSSGLSEDQK	99.70%	104	30.8	58.9	1,388.68
ELF4-HFC Col rep 2	Symbols: PHYC | phytochrome C | chr5:14008049-14011619 FORWARD LENGTH=1111	AT5G35840.1	TAIR10_20101214.fasta	123,724.60	100.00%	27	34	41	0.20%	30.50%	AISFLQDLLHSSGLSEDQKR	99.70%	45.8	30.4	66.3	2,517.28
ELF4-HFC Col rep 2	Symbols: PHYC | phytochrome C | chr5:14008049-14011619 FORWARD LENGTH=1111	AT5G35840.1	TAIR10_20101214.fasta	123,724.60	100.00%	27	34	41	0.20%	30.50%	AVDFGEISILNPITLHCR	99.70%	65.8	30	49.9	1,290.67
ELF4-HFC Col rep 2	Symbols: PHYC | phytochrome C | chr5:14008049-14011619 FORWARD LENGTH=1111	AT5G35840.1	TAIR10_20101214.fasta	123,724.60	100.00%	27	34	41	0.20%	30.50%	CDGAALYYR	99.70%	40.6	25	107	1,960.95
ELF4-HFC Col rep 2	Symbols: PHYC | phytochrome C | chr5:14008049-14011619 FORWARD LENGTH=1111	AT5G35840.1	TAIR10_20101214.fasta	123,724.60	100.00%	27	34	41	0.20%	30.50%	DGSFIEALLSANK	99.70%	96.7	30	61.1	1,416.77
ELF4-HFC Col rep 2	Symbols: PHYC | phytochrome C | chr5:14008049-14011619 FORWARD LENGTH=1111	AT5G35840.1	TAIR10_20101214.fasta	123,724.60	100.00%	27	34	41	0.20%	30.50%	DGSFIEALLSANKR	96.30%	24.7	29.5	51.8	1,293.64
ELF4-HFC Col rep 2	Symbols: PHYC | phytochrome C | chr5:14008049-14011619 FORWARD LENGTH=1111	AT5G35840.1	TAIR10_20101214.fasta	123,724.60	100.00%	27	34	41	0.20%	30.50%	DLIDWVLK	99.00%	50.5	28.8	0	2,527.26
ELF4-HFC Col rep 2	Symbols: PHYC | phytochrome C | chr5:14008049-14011619 FORWARD LENGTH=1111	AT5G35840.1	TAIR10_20101214.fasta	123,724.60	100.00%	27	34	41	0.20%	30.50%	DNLWSLGVTPTETQIR	99.70%	74.2	30.5	13.3	1,184.54
ELF4-HFC Col rep 2	Symbols: PHYC | phytochrome C | chr5:14008049-14011619 FORWARD LENGTH=1111	AT5G35840.1	TAIR10_20101214.fasta	123,724.60	100.00%	27	34	41	0.20%	30.50%	EAESAVLLK	99.70%	41.3	29.4	32.9	1,074.51
ELF4-HFC Col rep 2	Symbols: PHYC | phytochrome C | chr5:14008049-14011619 FORWARD LENGTH=1111	AT5G35840.1	TAIR10_20101214.fasta	123,724.60	100.00%	27	34	41	0.20%	30.50%	EALTIGTDVK	99.30%	33.8	29.8	31.4	1,163.61
ELF4-HFC Col rep 2	Symbols: PHYC | phytochrome C | chr5:14008049-14011619 FORWARD LENGTH=1111	AT5G35840.1	TAIR10_20101214.fasta	123,724.60	100.00%	27	34	41	0.20%	30.50%	EGLGLHITQK	99.40%	31.2	25.9	68.8	1,439.77
ELF4-HFC Col rep 2	Symbols: PHYC | phytochrome C | chr5:14008049-14011619 FORWARD LENGTH=1111	AT5G35840.1	TAIR10_20101214.fasta	123,724.60	100.00%	27	34	41	0.20%	30.50%	EVSELTGYDR	99.70%	54.6	27.7	42.4	1,037.59
ELF4-HFC Col rep 2	Symbols: PHYC | phytochrome C | chr5:14008049-14011619 FORWARD LENGTH=1111	AT5G35840.1	TAIR10_20101214.fasta	123,724.60	100.00%	27	34	41	0.20%	30.50%	GSLQEEHSK	99.70%	47	25.6	45.7	1,254.67
ELF4-HFC Col rep 2	Symbols: PHYC | phytochrome C | chr5:14008049-14011619 FORWARD LENGTH=1111	AT5G35840.1	TAIR10_20101214.fasta	123,724.60	100.00%	27	34	41	0.20%	30.50%	IGFNAVISGQK	99.70%	76.3	28.6	88.5	1,614.83
ELF4-HFC Col rep 2	Symbols: PHYC | phytochrome C | chr5:14008049-14011619 FORWARD LENGTH=1111	AT5G35840.1	TAIR10_20101214.fasta	123,724.60	100.00%	27	34	41	0.20%	30.50%	IIHPAPGLPEDLVR	99.70%	43.8	26	64.7	2,102.04
ELF4-HFC Col rep 2	Symbols: PHYC | phytochrome C | chr5:14008049-14011619 FORWARD LENGTH=1111	AT5G35840.1	TAIR10_20101214.fasta	123,724.60	100.00%	27	34	41	0.20%	30.50%	ILQTQSVLCDMLFR	99.70%	38.1	29.9	85.3	2,585.39
ELF4-HFC Col rep 2	Symbols: PHYC | phytochrome C | chr5:14008049-14011619 FORWARD LENGTH=1111	AT5G35840.1	TAIR10_20101214.fasta	123,724.60	100.00%	27	34	41	0.20%	30.50%	LHGNFEESER	97.00%	23.3	25.8	20.9	889.4487
ELF4-HFC Col rep 2	Symbols: PHYC | phytochrome C | chr5:14008049-14011619 FORWARD LENGTH=1111	AT5G35840.1	TAIR10_20101214.fasta	123,724.60	100.00%	27	34	41	0.20%	30.50%	LIDTAAVPIFAVDASGVINGWNSK	99.70%	91	30.7	27.6	1,128.58
ELF4-HFC Col rep 2	Symbols: PHYC | phytochrome C | chr5:14008049-14011619 FORWARD LENGTH=1111	AT5G35840.1	TAIR10_20101214.fasta	123,724.60	100.00%	27	34	41	0.20%	30.50%	LLFGFYHR	99.00%	41.7	27.6	51.9	2,015.02
ELF4-HFC Col rep 2	Symbols: PHYC | phytochrome C | chr5:14008049-14011619 FORWARD LENGTH=1111	AT5G35840.1	TAIR10_20101214.fasta	123,724.60	100.00%	27	34	41	0.20%	30.50%	LQQILSETLLSSIR	99.70%	109	25.3	60.8	1,563.84
ELF4-HFC Col rep 2	Symbols: PHYC | phytochrome C | chr5:14008049-14011619 FORWARD LENGTH=1111	AT5G35840.1	TAIR10_20101214.fasta	123,724.60	100.00%	27	34	41	0.20%	30.50%	NAPIGIVTQSPNIMDLVK	99.70%	65.5	28.7	66.1	3,069.58
ELF4-HFC Col rep 2	Symbols: PHYC | phytochrome C | chr5:14008049-14011619 FORWARD LENGTH=1111	AT5G35840.1	TAIR10_20101214.fasta	123,724.60	100.00%	27	34	41	0.20%	30.50%	SSPVELVVNTCCSR	99.70%	57.9	29.4	91.3	2,088.06
ELF4-HFC Col rep 2	Symbols: PHYC | phytochrome C | chr5:14008049-14011619 FORWARD LENGTH=1111	AT5G35840.1	TAIR10_20101214.fasta	123,724.60	100.00%	27	34	41	0.20%	30.50%	SSSKPFYAILHR	99.70%	48.9	29.1	39.3	2,244.16
ELF4-HFC Col rep 2	Symbols: PHYC | phytochrome C | chr5:14008049-14011619 FORWARD LENGTH=1111	AT5G35840.1	TAIR10_20101214.fasta	123,724.60	100.00%	27	34	41	0.20%	30.50%	TVVDVPLVDNR	99.70%	62.1	26.9	56.7	2,055.07
ELF4-HFC Col rep 2	Symbols: PHYC | phytochrome C | chr5:14008049-14011619 FORWARD LENGTH=1111	AT5G35840.1	TAIR10_20101214.fasta	123,724.60	100.00%	27	34	41	0.20%	30.50%	VSSQVLVDAK	99.70%	61	29.5	38.5	1,088.48
ELF4-HFC Col rep 2	Symbols: PHYC | phytochrome C | chr5:14008049-14011619 FORWARD LENGTH=1111	AT5G35840.1	TAIR10_20101214.fasta	123,724.60	100.00%	27	34	41	0.20%	30.50%	YACEFLTQVFGVQINK	99.70%	109	30.5	82.6	1,364.71
ELF4-HFC Col rep 2	Symbols: PHYD | phytochrome D | chr4:9195602-9199486 REVERSE LENGTH=1164	AT4G16250.1	TAIR10_20101214.fasta	129,270.30	100.00%	3	3	15	0.07%	13.00%	AIQQYTVDAR	99.70%	58.3	30	16.7	1,520.81
ELF4-HFC Col rep 2	Symbols: PHYD | phytochrome D | chr4:9195602-9199486 REVERSE LENGTH=1164	AT4G16250.1	TAIR10_20101214.fasta	129,270.30	100.00%	3	3	15	0.07%	13.00%	FHEDEHGEVVAESK	99.70%	112	27.2	23.2	1,001.57
ELF4-HFC Col rep 2	Symbols: PHYD | phytochrome D | chr4:9195602-9199486 REVERSE LENGTH=1164	AT4G16250.1	TAIR10_20101214.fasta	129,270.30	100.00%	3	3	15	0.07%	13.00%	FINIQGDYK	99.70%	55.5	28	60.4	1,829.94
ELF4-HFC Col rep 2	Symbols: PHYD | phytochrome D | chr4:9195602-9199486 REVERSE LENGTH=1164	AT4G16250.1	TAIR10_20101214.fasta	129,270.30	100.00%	3	3	15	0.07%	13.00%	HHPEDKDDGQR	97.30%	31	25	20.9	959.5408
ELF4-HFC Col rep 2	Symbols: PHYD | phytochrome D | chr4:9195602-9199486 REVERSE LENGTH=1164	AT4G16250.1	TAIR10_20101214.fasta	129,270.30	100.00%	3	3	15	0.07%	13.00%	HTSAAEQAQSSANK	99.70%	55.8	28.2	23.4	1,046.57
ELF4-HFC Col rep 2	Symbols: PHYD | phytochrome D | chr4:9195602-9199486 REVERSE LENGTH=1164	AT4G16250.1	TAIR10_20101214.fasta	129,270.30	100.00%	3	3	15	0.07%	13.00%	LHAVFEQSGESGK	99.70%	66.6	29.2	23.1	1,095.62
ELF4-HFC Col rep 2	Symbols: PHYD | phytochrome D | chr4:9195602-9199486 REVERSE LENGTH=1164	AT4G16250.1	TAIR10_20101214.fasta	129,270.30	100.00%	3	3	15	0.07%	13.00%	LLCDTVVESVR	99.70%	72.2	30.6	44.4	1,168.55
ELF4-HFC Col rep 2	Symbols: PHYD | phytochrome D | chr4:9195602-9199486 REVERSE LENGTH=1164	AT4G16250.1	TAIR10_20101214.fasta	129,270.30	100.00%	3	3	15	0.07%	13.00%	NDLEPYIGLHYPATDIPQASR	99.70%	50	31.3	29.7	1,014.49
ELF4-HFC Col rep 2	Symbols: PHYD | phytochrome D | chr4:9195602-9199486 REVERSE LENGTH=1164	AT4G16250.1	TAIR10_20101214.fasta	129,270.30	100.00%	3	3	15	0.07%	13.00%	QLLETSVSCEK	99.70%	59.4	30.6	52.1	1,133.63
ELF4-HFC Col rep 2	Symbols: PHYD | phytochrome D | chr4:9195602-9199486 REVERSE LENGTH=1164	AT4G16250.1	TAIR10_20101214.fasta	129,270.30	100.00%	3	3	15	0.07%	13.00%	RNDLEPYIGLHYPATDIPQASR	99.70%	39	31.3	28.3	1,526.87
ELF4-HFC Col rep 2	Symbols: PHYD | phytochrome D | chr4:9195602-9199486 REVERSE LENGTH=1164	AT4G16250.1	TAIR10_20101214.fasta	129,270.30	100.00%	3	3	15	0.07%	13.00%	SEVLTIGTDLR	99.70%	59.2	29.8	34.6	1,739.88
ELF4-HFC Col rep 2	Symbols: PHYD | phytochrome D | chr4:9195602-9199486 REVERSE LENGTH=1164	AT4G16250.1	TAIR10_20101214.fasta	129,270.30	100.00%	3	3	15	0.07%	13.00%	SFDYSQSLK	99.70%	43.9	26.6	6.04	1,217.55
ELF4-HFC Col rep 2	Symbols: PHYD | phytochrome D | chr4:9195602-9199486 REVERSE LENGTH=1164	AT4G16250.1	TAIR10_20101214.fasta	129,270.30	100.00%	3	3	15	0.07%	13.00%	TEDPALSIAGAVQSQK	99.70%	101	29.4	12.5	2,459.28
ELF4-HFC Col rep 2	Symbols: PHYE | phytochrome E | chr4:10042312-10045948 REVERSE LENGTH=1112	AT4G18130.1	TAIR10_20101214.fasta	122,519.10	100.00%	18	19	20	0.10%	24.70%	AALESLLCK	99.70%	56.3	30.1	32	1,052.57
ELF4-HFC Col rep 2	Symbols: PHYE | phytochrome E | chr4:10042312-10045948 REVERSE LENGTH=1112	AT4G18130.1	TAIR10_20101214.fasta	122,519.10	100.00%	18	19	20	0.10%	24.70%	AASFTEISLLNPVLVHSR	99.70%	36.5	27	107	1,600.93
ELF4-HFC Col rep 2	Symbols: PHYE | phytochrome E | chr4:10042312-10045948 REVERSE LENGTH=1112	AT4G18130.1	TAIR10_20101214.fasta	122,519.10	100.00%	18	19	20	0.10%	24.70%	DGWVTPDGLGLK	95.70%	27.2	27.7	64.7	1,926.04
ELF4-HFC Col rep 2	Symbols: PHYE | phytochrome E | chr4:10042312-10045948 REVERSE LENGTH=1112	AT4G18130.1	TAIR10_20101214.fasta	122,519.10	100.00%	18	19	20	0.10%	24.70%	ESAQSLNELTYVR	99.70%	52.7	30.5	53.7	1,607.75
ELF4-HFC Col rep 2	Symbols: PHYE | phytochrome E | chr4:10042312-10045948 REVERSE LENGTH=1112	AT4G18130.1	TAIR10_20101214.fasta	122,519.10	100.00%	18	19	20	0.10%	24.70%	ESFTSSRPVLSGNGVAR	99.70%	52.6	31.5	38.2	1,405.76
ELF4-HFC Col rep 2	Symbols: PHYE | phytochrome E | chr4:10042312-10045948 REVERSE LENGTH=1112	AT4G18130.1	TAIR10_20101214.fasta	122,519.10	100.00%	18	19	20	0.10%	24.70%	FGQNNHPDYSSDVCVLVNSCTSR	96.50%	32.5	28.1	54.3	1,226.67
ELF4-HFC Col rep 2	Symbols: PHYE | phytochrome E | chr4:10042312-10045948 REVERSE LENGTH=1112	AT4G18130.1	TAIR10_20101214.fasta	122,519.10	100.00%	18	19	20	0.10%	24.70%	ILGLSDNSSDFLGLLSLPSTSHSGEFDK	99.70%	78.1	32	47.3	1,045.59
ELF4-HFC Col rep 2	Symbols: PHYE | phytochrome E | chr4:10042312-10045948 REVERSE LENGTH=1112	AT4G18130.1	TAIR10_20101214.fasta	122,519.10	100.00%	18	19	20	0.10%	24.70%	LLESSEISASQR	99.70%	73.5	30.5	100	1,916.96
ELF4-HFC Col rep 2	Symbols: PHYE | phytochrome E | chr4:10042312-10045948 REVERSE LENGTH=1112	AT4G18130.1	TAIR10_20101214.fasta	122,519.10	100.00%	18	19	20	0.10%	24.70%	LQLILADLLR	99.70%	50.6	25	50.5	1,164.60

ELF4-HFC Col rep 2	| Symbols: PHYE | phytochrome E | chr4:10042312-10045948 REVERSE LENGTH=1112	AT4G18130.1	TAIR10_20101214.fasta	122,519.10	100.00%	18	19	20	0.10%	24.70%	NIVNHAPFPNSWVGISISPGQELSR	99.70%	47.6	30.8	103	1,612.72
ELF4-HFC Col rep 2	| Symbols: PHYE | phytochrome E | chr4:10042312-10045948 REVERSE LENGTH=1112	AT4G18130.1	TAIR10_20101214.fasta	122,519.10	100.00%	18	19	20	0.10%	24.70%	QITTIIESTDLK	99.70%	63.9	28.7	39.6	1,097.56
ELF4-HFC Col rep 2	| Symbols: PHYE | phytochrome E | chr4:10042312-10045948 REVERSE LENGTH=1112	AT4G18130.1	TAIR10_20101214.fasta	122,519.10	100.00%	18	19	20	0.10%	24.70%	RPLCLVNSTLR	99.70%	38.1	28.5	23	1,333.59
ELF4-HFC Col rep 2	| Symbols: PHYE | phytochrome E | chr4:10042312-10045948 REVERSE LENGTH=1112	AT4G18130.1	TAIR10_20101214.fasta	122,519.10	100.00%	18	19	20	0.10%	24.70%	SGDPALTLAGAVQSQK	99.70%	39.4	29.5	47.2	1,429.67
ELF4-HFC Col rep 2	| Symbols: PHYE | phytochrome E | chr4:10042312-10045948 REVERSE LENGTH=1112	AT4G18130.1	TAIR10_20101214.fasta	122,519.10	100.00%	18	19	20	0.10%	24.70%	SLADEIVQEESR	99.70%	69.4	29.4	58.9	1,388.68
ELF4-HFC Col rep 2	| Symbols: PHYE | phytochrome E | chr4:10042312-10045948 REVERSE LENGTH=1112	AT4G18130.1	TAIR10_20101214.fasta	122,519.10	100.00%	18	19	20	0.10%	24.70%	SSFTAFLEVAK	99.70%	75.5	27.3	49.9	1,290.67
ELF4-HFC Col rep 2	| Symbols: PHYE | phytochrome E | chr4:10042312-10045948 REVERSE LENGTH=1112	AT4G18130.1	TAIR10_20101214.fasta	122,519.10	100.00%	18	19	20	0.10%	24.70%	TLFTPSSGASLSK	99.70%	53.8	29.1	0.74	2,371.16
ELF4-HFC Col rep 2	| Symbols: PHYE | phytochrome E | chr4:10042312-10045948 REVERSE LENGTH=1112	AT4G18130.1	TAIR10_20101214.fasta	122,519.10	100.00%	18	19	20	0.10%	24.70%	VIETATAPIFGVDSSGCINGWNK	99.70%	61.9	31.5	51.8	1,293.64
ELF4-HFC Col rep 2	| Symbols: PHYE | phytochrome E | chr4:10042312-10045948 REVERSE LENGTH=1112	AT4G18130.1	TAIR10_20101214.fasta	122,519.10	100.00%	18	19	20	0.10%	24.70%	YIEASLTANK	99.70%	51.8	28.8	0	2,527.26
ELF4-HFC Col rep 2	| Symbols: SPA1 | SPA (suppressor of phyA-105) protein family | chr2:19022572-19026821 REVERSE LENGTH=1029	AT2G46340.1	TAIR10_20101214.fasta	114,689.10	100.00%	2	2	3	0.01%	2.92%	NDLESDVDEDLNR	99.70%	62.2	25.4	50.6	1,203.66
ELF4-HFC Col rep 2	| Symbols: SPA1 | SPA (suppressor of phyA-105) protein family | chr2:19022572-19026821 REVERSE LENGTH=1029	AT2G46340.1	TAIR10_20101214.fasta	114,689.10	100.00%	2	2	3	0.01%	2.92%	NQETPPEFVSDQDLGSK	99.70%	48.3	29.3	32.9	1,074.51
ELF4-HFC Col rep 2	| Symbols: TIC | time for coffee | chr3:7913181-7918879 FORWARD LENGTH=1550	AT3G22380.1,AT3G22380.2	TAIR10_20101214.fasta	164,452.80	100.00%	3	3	5	0.02%	3.10%	DSPEEEGPVELPEAAR	99.70%	45.4	29.4	88.5	1,614.83
ELF4-HFC Col rep 2	| Symbols: TIC | time for coffee | chr3:7913181-7918879 FORWARD LENGTH=1550	AT3G22380.1,AT3G22380.2	TAIR10_20101214.fasta	164,452.80	100.00%	3	3	5	0.02%	3.10%	GGQASTTTHSASQPSK	99.70%	71.6	28.4	44.4	1,004.54
ELF4-HFC Col rep 2	| Symbols: TIC | time for coffee | chr3:7913181-7918879 FORWARD LENGTH=1550	AT3G22380.1,AT3G22380.2	TAIR10_20101214.fasta	164,452.80	100.00%	3	3	5	0.02%	3.10%	SSPSPAPVSPLVSTWK	99.70%	65.8	28.7	33.8	1,954.08
ELF4-HFC Col rep 2	| Symbols: TZP | zinc knuckle (CCHC-type) family protein | chr5:17527261-17530207 FORWARD LENGTH=831	AT5G43630.1	TAIR10_20101214.fasta	90,615.60	100.00%	22	26	30	0.15%	35.40%	AVFDAVQVLR	99.70%	77.2	28.4	9.05	1,257.65
ELF4-HFC Col rep 2	| Symbols: TZP | zinc knuckle (CCHC-type) family protein | chr5:17527261-17530207 FORWARD LENGTH=831	AT5G43630.1	TAIR10_20101214.fasta	90,615.60	100.00%	22	26	30	0.15%	35.40%	AWWQSAGK	99.00%	44.9	28.7	44.6	1,509.75
ELF4-HFC Col rep 2	| Symbols: TZP | zinc knuckle (CCHC-type) family protein | chr5:17527261-17530207 FORWARD LENGTH=831	AT5G43630.1	TAIR10_20101214.fasta	90,615.60	100.00%	22	26	30	0.15%	35.40%	CADISFTGK	99.70%	37.1	25.4	43.3	1,764.89
ELF4-HFC Col rep 2	| Symbols: TZP | zinc knuckle (CCHC-type) family protein | chr5:17527261-17530207 FORWARD LENGTH=831	AT5G43630.1	TAIR10_20101214.fasta	90,615.60	100.00%	22	26	30	0.15%	35.40%	CFQLSHWAATCPNAPLYGSGAEGR	99.70%	46.7	29.7	31.9	2,656.15
ELF4-HFC Col rep 2	| Symbols: TZP | zinc knuckle (CCHC-type) family protein | chr5:17527261-17530207 FORWARD LENGTH=831	AT5G43630.1	TAIR10_20101214.fasta	90,615.60	100.00%	22	26	30	0.15%	35.40%	DCLEVTDTELR	99.70%	67.3	28.2	75	2,936.45
ELF4-HFC Col rep 2	| Symbols: TZP | zinc knuckle (CCHC-type) family protein | chr5:17527261-17530207 FORWARD LENGTH=831	AT5G43630.1	TAIR10_20101214.fasta	90,615.60	100.00%	22	26	30	0.15%	35.40%	DLLVNEQLR	99.70%	40.9	26.1	58.7	1,319.68
ELF4-HFC Col rep 2	| Symbols: TZP | zinc knuckle (CCHC-type) family protein | chr5:17527261-17530207 FORWARD LENGTH=831	AT5G43630.1	TAIR10_20101214.fasta	90,615.60	100.00%	22	26	30	0.15%	35.40%	DLVQNISVR	99.70%	53	28.8	41.4	1,167.75
ELF4-HFC Col rep 2	| Symbols: TZP | zinc knuckle (CCHC-type) family protein | chr5:17527261-17530207 FORWARD LENGTH=831	AT5G43630.1	TAIR10_20101214.fasta	90,615.60	100.00%	22	26	30	0.15%	35.40%	EANASASDAAK	99.70%	47.2	26.8	41.8	2,719.40
ELF4-HFC Col rep 2	| Symbols: TZP | zinc knuckle (CCHC-type) family protein | chr5:17527261-17530207 FORWARD LENGTH=831	AT5G43630.1	TAIR10_20101214.fasta	90,615.60	100.00%	22	26	30	0.15%	35.40%	EEASSLCIR	99.70%	47.8	28.6	49.7	1,361.75
ELF4-HFC Col rep 2	| Symbols: TZP | zinc knuckle (CCHC-type) family protein | chr5:17527261-17530207 FORWARD LENGTH=831	AT5G43630.1	TAIR10_20101214.fasta	90,615.60	100.00%	22	26	30	0.15%	35.40%	FAAVDAITELVWSPSNGLSLR	99.70%	77.7	30.5	23.3	1,328.75
ELF4-HFC Col rep 2	| Symbols: TZP | zinc knuckle (CCHC-type) family protein | chr5:17527261-17530207 FORWARD LENGTH=831	AT5G43630.1	TAIR10_20101214.fasta	90,615.60	100.00%	22	26	30	0.15%	35.40%	GDDLSSSGNEIGPVAEPNISSGK	99.70%	102	30.6	30	1,542.81
ELF4-HFC Col rep 2	| Symbols: TZP | zinc knuckle (CCHC-type) family protein | chr5:17527261-17530207 FORWARD LENGTH=831	AT5G43630.1	TAIR10_20101214.fasta	90,615.60	100.00%	22	26	30	0.15%	35.40%	GDYLPEGEAGPSGSYR	99.70%	56.8	27.2	53.8	1,375.67
ELF4-HFC Col rep 2	| Symbols: TZP | zinc knuckle (CCHC-type) family protein | chr5:17527261-17530207 FORWARD LENGTH=831	AT5G43630.1	TAIR10_20101214.fasta	90,615.60	100.00%	22	26	30	0.15%	35.40%	GNEEDNSPFVALTTTSNANGHGQVNAIVDQQQLSPCCVK	99.70%	87.1	30.6	63.6	1,199.63
ELF4-HFC Col rep 2	| Symbols: TZP | zinc knuckle (CCHC-type) family protein | chr5:17527261-17530207 FORWARD LENGTH=831	AT5G43630.1	TAIR10_20101214.fasta	90,615.60	100.00%	22	26	30	0.15%	35.40%	GVTCLVESQFISNQDFLEEELK	99.70%	94.3	31.6	30.9	1,295.68
ELF4-HFC Col rep 2	| Symbols: TZP | zinc knuckle (CCHC-type) family protein | chr5:17527261-17530207 FORWARD LENGTH=831	AT5G43630.1	TAIR10_20101214.fasta	90,615.60	100.00%	22	26	30	0.15%	35.40%	IDGDTEGQSSR	99.70%	80.6	25.5	13.8	2,437.17
ELF4-HFC Col rep 2	| Symbols: TZP | zinc knuckle (CCHC-type) family protein | chr5:17527261-17530207 FORWARD LENGTH=831	AT5G43630.1	TAIR10_20101214.fasta	90,615.60	100.00%	22	26	30	0.15%	35.40%	KRPGFEEQLIFGSK	99.70%	58.5	28.8	33.2	1,109.58
ELF4-HFC Col rep 2	| Symbols: TZP | zinc knuckle (CCHC-type) family protein | chr5:17527261-17530207 FORWARD LENGTH=831	AT5G43630.1	TAIR10_20101214.fasta	90,615.60	100.00%	22	26	30	0.15%	35.40%	LPISGFTDVPR	99.70%	83.2	29.4	56.4	1,533.67
ELF4-HFC Col rep 2	| Symbols: TZP | zinc knuckle (CCHC-type) family protein | chr5:17527261-17530207 FORWARD LENGTH=831	AT5G43630.1	TAIR10_20101214.fasta	90,615.60	100.00%	22	26	30	0.15%	35.40%	NGREEASSLCIR	99.70%	48.2	28.6	42.8	1,890.87
ELF4-HFC Col rep 2	| Symbols: TZP | zinc knuckle (CCHC-type) family protein | chr5:17527261-17530207 FORWARD LENGTH=831	AT5G43630.1	TAIR10_20101214.fasta	90,615.60	100.00%	22	26	30	0.15%	35.40%	RPGFEEQLIFGSK	99.70%	42	29.8	30.2	1,724.80
ELF4-HFC Col rep 2	| Symbols: TZP | zinc knuckle (CCHC-type) family protein | chr5:17527261-17530207 FORWARD LENGTH=831	AT5G43630.1	TAIR10_20101214.fasta	90,615.60	100.00%	22	26	30	0.15%	35.40%	SVSGLEGFFLR	99.70%	61.8	27.8	68	1,544.73
ELF4-HFC Col rep 2	| Symbols: TZP | zinc knuckle (CCHC-type) family protein | chr5:17527261-17530207 FORWARD LENGTH=831	AT5G43630.1	TAIR10_20101214.fasta	90,615.60	100.00%	22	26	30	0.15%	35.40%	TSGYDGIPSAEELSR	99.70%	49.3	29.8	53.1	1,639.87
ELF4-HFC Col rep 2	| Symbols: TZP | zinc knuckle (CCHC-type) family protein | chr5:17527261-17530207 FORWARD LENGTH=831	AT5G43630.1	TAIR10_20101214.fasta	90,615.60	100.00%	22	26	30	0.15%	35.40%	VSPCLTNR	97.20%	29.9	28.6	56	1,117.64
ELF4-HFC Col rep 3	| Symbols: | F-box family protein | chr2:7074940-7079181 FORWARD LENGTH=727	AT2G16365.4	TAIR10_20101214.fasta	82,716.20	100.00%	12	15	16	0.08%	23.80%	ALSGLNPYGLR	99.70%	54.6	30.1	26.1	933.4577
ELF4-HFC Col rep 3	| Symbols: | F-box family protein | chr2:7074940-7079181 FORWARD LENGTH=727	AT2G16365.4	TAIR10_20101214.fasta	82,716.20	100.00%	12	15	16	0.08%	23.80%	ENNNLFLEILK	99.70%	67.1	30.2	26	998.4613
ELF4-HFC Col rep 3	| Symbols: | F-box family protein | chr2:7074940-7079181 FORWARD LENGTH=727	AT2G16365.4	TAIR10_20101214.fasta	82,716.20	100.00%	12	15	16	0.08%	23.80%	ETTTTTTTKPSFHEDVGSSSR	99.70%	40.3	30.4	43.5	2,650.19
ELF4-HFC Col rep 3	| Symbols: | F-box family protein | chr2:7074940-7079181 FORWARD LENGTH=727	AT2G16365.4	TAIR10_20101214.fasta	82,716.20	100.00%	12	15	16	0.08%	23.80%	IFGGYLDLFPNQDHSHNR	99.70%	47.3	30.7	38.8	1,350.62
ELF4-HFC Col rep 3	| Symbols: | F-box family protein | chr2:7074940-7079181 FORWARD LENGTH=727	AT2G16365.4	TAIR10_20101214.fasta	82,716.20	100.00%	12	15	16	0.08%	23.80%	IYATVDSVNLPK	99.70%	62.7	29.5	18.8	1,099.61
ELF4-HFC Col rep 3	| Symbols: | F-box family protein | chr2:7074940-7079181 FORWARD LENGTH=727	AT2G16365.4	TAIR10_20101214.fasta	82,716.20	100.00%	12	15	16	0.08%	23.80%	LDFPIQEK	99.00%	42.5	30.2	35.9	1,043.58
ELF4-HFC Col rep 3	| Symbols: | F-box family protein | chr2:7074940-7079181 FORWARD LENGTH=727	AT2G16365.4	TAIR10_20101214.fasta	82,716.20	100.00%	12	15	16	0.08%	23.80%	LEPEVEASSR	98.40%	32.7	27.9	38.3	1,034.47
ELF4-HFC Col rep 3	| Symbols: | F-box family protein | chr2:7074940-7079181 FORWARD LENGTH=727	AT2G16365.4	TAIR10_20101214.fasta	82,716.20	100.00%	12	15	16	0.08%	23.80%	LQTIHLSGSISSSSTK	99.70%	41.9	30.5	34.6	1,064.50
ELF4-HFC Col rep 3	| Symbols: | F-box family protein | chr2:7074940-7079181 FORWARD LENGTH=727	AT2G16365.4	TAIR10_20101214.fasta	82,716.20	100.00%	12	15	16	0.08%	23.80%	NSSVNPYESAWLGR	99.70%	62.6	30.2	72.2	2,247.17
ELF4-HFC Col rep 3	| Symbols: | F-box family protein | chr2:7074940-7079181 FORWARD LENGTH=727	AT2G16365.4	TAIR10_20101214.fasta	82,716.20	100.00%	12	15	16	0.08%	23.80%	QGGDDITLLHPWIQR	99.70%	33.1	30.7	98.1	2,230.05
ELF4-HFC Col rep 3	| Symbols: | F-box family protein | chr2:7074940-7079181 FORWARD LENGTH=727	AT2G16365.4	TAIR10_20101214.fasta	82,716.20	100.00%	12	15	16	0.08%	23.80%	TTQNLLELIRPVR	99.70%	35.3	25	50.5	1,654.73
ELF4-HFC Col rep 3	| Symbols: | F-box family protein | chr2:7074940-7079181 FORWARD LENGTH=727	AT2G16365.4	TAIR10_20101214.fasta	82,716.20	100.00%	12	15	16	0.08%	23.80%	TTSTDQPTGQEASFEPESHKEFEK	99.70%	51.8	28.3	4.96	4,202.89
ELF4-HFC Col rep 3	| Symbols: | Granulin repeat cysteine protease family protein | chr3:6723024-6724768 FORWARD LENGTH=452	AT3G19390.1	TAIR10_20101214.fasta	49,308.20	100.00%	2	2	2	0.01%	7.08%	ALANQPISVAIEAGGR	99.70%	55.2	27.4	88.1	2,585.24
ELF4-HFC Col rep 3	| Symbols: | Granulin repeat cysteine protease family protein | chr3:6723024-6724768 FORWARD LENGTH=452	AT3G19390.1	TAIR10_20101214.fasta	49,308.20	100.00%	2	2	2	0.01%	7.08%	VVTIDGYEDVPQNDEK	99.70%	50.2	30	71.4	1,164.51
ELF4-HFC Col rep 3	| Symbols: | Protein kinase family protein | chr3:4469434-4473234 FORWARD LENGTH=703	AT3G13670.1	TAIR10_20101214.fasta	78,640.00	100.00%	1	1	3	0.01%	8.11%	GGFGQVFVGR	99.70%	61.7	28.6	53	1,635.89
ELF4-HFC Col rep 3	| Symbols: | Protein kinase family protein | chr3:4469434-4473234 FORWARD LENGTH=703	AT3G13670.1	TAIR10_20101214.fasta	78,640.00	100.00%	1	1	3	0.01%	8.11%	LVSLFQDLLGENPAIRPINTEGAQK	99.70%	34.4	27.3	74.1	1,201.66
ELF4-HFC Col rep 3	| Symbols: | Protein kinase family protein | chr3:4469434-4473234 FORWARD LENGTH=703	AT3G13670.1	TAIR10_20101214.fasta	78,640.00	100.00%	1	1	3	0.01%	8.11%	NSGYSEQVVELDFLYPSEGIHR	99.70%	77.2	31.4	27.7	1,392.65
ELF4-HFC Col rep 3	| Symbols: | Protein kinase family protein | chr5:6010215-6013724 REVERSE LENGTH=691	AT5G18190.1	TAIR10_20101214.fasta	76,985.50	99.80%	1	1	2	0.01%	5.64%	GGFGQVFVGR	99.70%	61.7	28.6	34	1,507.79
ELF4-HFC Col rep 3	| Symbols: | Protein kinase family protein | chr5:6010215-6013724 REVERSE LENGTH=691	AT5G18190.1	TAIR10_20101214.fasta	76,985.50	99.80%	1	1	2	0.01%	5.64%	LIDLEAEPCEVRPAVGEPAFNQVEAVAEK	99.70%	41	31.6	47.5	1,211.64
ELF4-HFC Col rep 3	| Symbols: ATAN11, LWD1 | Transducin/WD40 repeat-like superfamily protein | chr1:4395114-4396154 REVERSE LENGTH=346	AT1G12910.1	TAIR10_20101214.fasta	39,089.30	100.00%	2	2	5	0.02%	15.30%	DKEHSTIIYESSEPDTPLVR	99.70%	38.8	31.8	19.1	1,581.74
ELF4-HFC Col rep 3	| Symbols: ATAN11, LWD1 | Transducin/WD40 repeat-like superfamily protein | chr1:4395114-4396154 REVERSE LENGTH=346	AT1G12910.1	TAIR10_20101214.fasta	39,089.30	100.00%	2	2	5	0.02%	15.30%	NSEFCGPLTSFDWNEAEPR	99.70%	61.2	26.4	14.8	946.4775
ELF4-HFC Col rep 3	| Symbols: ATAN11, LWD1 | Transducin/WD40 repeat-like superfamily protein | chr1:4395114-4396154 REVERSE LENGTH=346	AT1G12910.1	TAIR10_20101214.fasta	39,089.30	100.00%	2	2	5	0.02%	15.30%	VEIVQLDESNGEIR	99.70%	61.9	29.8	37.2	1,160.64
ELF4-HFC Col rep 3	| Symbols: COP1, ATCOP1, DET340, FUS1, EMB168 | Transducin/WD40 repeat-like superfamily protein | chr2:13978000-13983282 FORWARD LENGTH=675	AT2G32950.1	TAIR10_20101214.fasta	76,188.00	100.00%	7	7	9	0.04%	15.00%	EGYSNGLADFQSVLTTFTR	99.50%	43.9	30.7	47.4	1,346.73
ELF4-HFC Col rep 3	| Symbols: COP1, ATCOP1, DET340, FUS1, EMB168 | Transducin/WD40 repeat-like superfamily protein | chr2:13978000-13983282 FORWARD LENGTH=675	AT2G32950.1	TAIR10_20101214.fasta	76,188.00	100.00%	7	7	9	0.04%	15.00%	EVDNLLTLLAER	99.70%	58.3	28.2	37.3	2,269.06
ELF4-HFC Col rep 3	| Symbols: COP1, ATCOP1, DET340, FUS1, EMB168 | Transducin/WD40 repeat-like superfamily protein | chr2:13978000-13983282 FORWARD LENGTH=675	AT2G32950.1	TAIR10_20101214.fasta	76,188.00	100.00%	7	7	9	0.04%	15.00%	FLSNNELASASTDSTLR	99.70%	118	30.6	25.4	2,130.02
ELF4-HFC Col rep 3	| Symbols: COP1, ATCOP1, DET340, FUS1, EMB168 | Transducin/WD40 repeat-like superfamily protein | chr2:13978000-13983282 FORWARD LENGTH=675	AT2G32950.1	TAIR10_20101214.fasta	76,188.00	100.00%	7	7	9	0.04%	15.00%	NQIGFNSNSLSIR	99.00%	33.7	30.7	49.6	1,319.72
ELF4-HFC Col rep 3	| Symbols: COP1, ATCOP1, DET340, FUS1, EMB168 | Transducin/WD40 repeat-like superfamily protein | chr2:13978000-13983282 FORWARD LENGTH=675	AT2G32950.1	TAIR10_20101214.fasta	76,188.00	100.00%	7	7	9	0.04%	15.00%	QKVDELNEVQTDLQYIK	95.80%	23.4	31	13.2	989.5304
ELF4-HFC Col rep 3	| Symbols: COP1, ATCOP1, DET340, FUS1, EMB168 | Transducin/WD40 repeat-like superfamily protein | chr2:13978000-13983282 FORWARD LENGTH=675	AT2G32950.1	TAIR10_20101214.fasta	76,188.00	100.00%	7	7	9	0.04%	15.00%	SDCPCCSQHLTNNQLYPNFLLDK	99.70%	37.7	28.4	23.6	1,116.55
ELF4-HFC Col rep 3	| Symbols: COP1, ATCOP1, DET340, FUS1, EMB168 | Transducin/WD40 repeat-like superfamily protein | chr2:13978000-13983282 FORWARD LENGTH=675	AT2G32950.1	TAIR10_20101214.fasta	76,188.00	100.00%	7	7	9	0.04%	15.00%	VDELNEVQTDLQYIK	98.50%	45.7	31.3	33.3	1,645.88
ELF4-HFC Col rep 3	| Symbols: ELF3, PYK20 | hydroxyproline-rich glycoprotein family protein | chr2:11059459-11063178 FORWARD LENGTH=695	AT2G25930.1	TAIR10_20101214.fasta	77,204.80	100.00%	13	19	24	0.12%	28.30%	EEEDFAVPVYINSR	99.40%	39	28.9	50.1	1,579.75
ELF4-HFC Col rep 3	| Symbols: ELF3, PYK20 | hydroxyproline-rich glycoprotein family protein | chr2:11059459-11063178 FORWARD LENGTH=695	AT2G25930.1	TAIR10_20101214.fasta	77,204.80	100.00%	13	19	24	0.12%	28.30%	FQEVNQTGSK	99.70%	56.1	29.2	19.6	1,748.91
ELF4-HFC Col rep 3	| Symbols: ELF3, PYK20 | hydroxyproline-rich glycoprotein family protein | chr2:11059459-11063178 FORWARD LENGTH=695	AT2G25930.1	TAIR10_20101214.fasta	77,204.80	100.00%	13	19	24	0.12%	28.30%	IFQSIQEER	99.70%	56	30.4	28.4	1,552.92
ELF4-HFC Col rep 3	| Symbols: ELF3, PYK20 | hydroxyproline-rich glycoprotein family protein | chr2:11059459-11063178 FORWARD LENGTH=695	AT2G25930.1	TAIR10_20101214.fasta	77,204.80	100.00%	13	19	24	0.12%	28.30%	LIAASPDLLLDEISFLGK	99.70%	105	25.9	3.51	2,711.20
ELF4-HFC Col rep 3	| Symbols: ELF3, PYK20 | hydroxyproline-rich glycoprotein family protein | chr2:11059459-11063178 FORWARD LENGTH=695	AT2G25930.1	TAIR10_20101214.fasta	77,204.80	100.00%	13	19	24	0.12%	28.30%	MALYEQLSIPSQR	99.70%	49.6	31.3	52.7	1,566.86
ELF4-HFC Col rep 3	| Symbols: ELF3, PYK20 | hydroxyproline-rich glycoprotein family protein | chr2:11059459-11063178 FORWARD LENGTH=695	AT2G25930.1	TAIR10_20101214.fasta	77,204.80	100.00%	13	19	24	0.12%	28.30%	MESSAENVVGR	99.70%	73.5	27.9	39.3	1,820.86
ELF4-HFC Col rep 3	| Symbols: ELF3, PYK20 | hydroxyproline-rich glycoprotein family protein | chr2:11059459-11063178 FORWARD LENGTH=695	AT2G25930.1	TAIR10_20101214.fasta	77,204.80	100.00%	13	19	24	0.12%	28.30%	NLSVQHLDSSAANQATEK	99.70%	110	31	50.2	1,023.54
ELF4-HFC Col rep 3	| Symbols: ELF3, PYK20 | hydroxyproline-rich glycoprotein family protein | chr2:11059459-11063178 FORWARD LENGTH=695	AT2G25930.1	TAIR10_20101214.fasta	77,204.80	100.00%	13	19	24	0.12%	28.30%	QGSTGSSPSGPQGISGSK	99.70%	96.5	29.1	33	2,723.47
ELF4-HFC Col rep 3	| Symbols: ELF3, PYK20 | hydroxyproline-rich glycoprotein family protein | chr2:11059459-11063178 FORWARD LENGTH=695	AT2G25930.1	TAIR10_20101214.fasta	77,204.80	100.00%	13	19	24	0.12%	28.30%	SDNEPAPQQQQQPTK	99.70%	68.6	29.6	64.4	2,539.21
ELF4-HFC Col rep 3	| Symbols: ELF3, PYK20 | hydroxyproline-rich glycoprotein family protein | chr2:11059459-11063178 FORWARD LENGTH=695	AT2G25930.1	TAIR10_20101214.fasta	77,204.80	100.00%	13	19	24	0.12%	28.30%	SFRPFAAVDEDSNINNAPEQTMTMTTTTTTR	99.70%	59	31	50.2	1,023.54
ELF4-HFC Col rep 3	| Symbols: ELF3, PYK20 | hydroxyproline-rich glycoprotein family protein | chr2:11059459-11063178 FORWARD LENGTH=695	AT2G25930.1	TAIR10_20101214.fasta	77,204.80	100.00%	13	19	24	0.12%	28.30%	SNNTSTLVHPGPSSQPCGVER	99.70%	36.8	30.3	25.2	3,180.59
ELF4-HFC Col rep 3	| Symbols: ELF3, PYK20 | hydroxyproline-rich glycoprotein family protein | chr2:11059459-11063178 FORWARD LENGTH=695	AT2G25930.1	TAIR10_20101214.fasta	77,204.80	100.00%	13	19	24	0.12%	28.30%	VFAVQLFELHR	99.70%	52.6	28.8	30.8	2,316.14
ELF4-HFC Col rep 3	| Symbols: ELF3, PYK20 | hydroxyproline-rich glycoprotein family protein | chr2:11059459-11063178 FORWARD LENGTH=695	AT2G25930.1	TAIR10_20101214.fasta	77,204.80	100.00%	13	19	24	0.12%	28.30%	VNDYNASLR	99.70%	68.9	29	57.6	2,255.97
ELF4-HFC Col rep 3	| Symbols: ELF4 | Protein of unknown function (DUF1313) | chr2:16734545-16734880 REVERSE LENGTH=111	AT2G40080.1	TAIR10_20101214.fasta	12,375.40	100.00%	6	25	183	0.91%	68.50%	NVAEEAEQGEDPAMWENLDR	99.70%	124	26.2	48	1,600.82
ELF4-HFC Col rep 3	| Symbols: ELF4 | Protein of unknown function (DUF1313) | chr2:16734545-16734880 REVERSE LENGTH=111	AT2G40080.1	TAIR10_20101214.fasta	12,375.40	100.00%	6	25	183	0.91%	68.50%	NVALIQELNGNISK	99.70%	120	28.8	34.4	2,107.00
ELF4-HFC Col rep 3	| Symbols: ELF4 | Protein of unknown function (DUF1313) | chr2:16734545-16734880 REVERSE LENGTH=111	AT2G40080.1	TAIR10_20101214.fasta	12,375.40	100.00%	6	25	183	0.91%	68.50%	QVQSVLDR	99.00%	50	29.3	49.7	1,385.76
ELF4-HFC Col rep 3	| Symbols: ELF4 | Protein of unknown function (DUF1313) | chr2:16734545-16734880 REVERSE LENGTH=111	AT2G40080.1	TAIR10_20101214.fasta	12,375.40	100.00%	6	25	183	0.91%	68.50%	RNVAEEAEQGEDPAMWENLDR	99.70%	87.4	28.6	109	1,825.89
ELF4-HFC Col rep 3	| Symbols: ELF4 | Protein of unknown function (DUF1313) | chr2:16734545-16734880 REVERSE LENGTH=111	AT2G40080.1	TAIR10_20101214.fasta	12,375.40	100.00%	6	25	183	0.91%	68.50%	SLIQQVNDNHQSR	99.70%	80.6	29.7	25.4	1,449.74
ELF4-HFC Col rep 3	| Symbols: ELF4 | Protein of unknown function (DUF1313) | chr2:16734545-16734880 REVERSE LENGTH=111	AT2G40080.1	TAIR10_20101214.fasta	12,375.40	100.00%	6	25	183	0.91%	68.50%	VVNMYSDLNTSFSSGFHGGK	99.70%	125	29.6	18.5	2,063.07
ELF4-HFC Col rep 3	| Symbols: PCL1, LUX | Homeodomain-like superfamily protein | chr3:17183248-17184219 FORWARD LENGTH=323	AT3G46640.1,AT3G46640.2	TAIR10_20101214.fasta	34,648.50	100.00%	3	3	5	0.02%	18.30%	ASETTLSSLR	99.70%	76.1	29.7	29	2,824.25
ELF4-HFC Col rep 3	| Symbols: PCL1, LUX | Homeodomain-like superfamily protein | chr3:17183248-17184219 FORWARD LENGTH=323	AT3G46640.1,AT3G46640.2	TAIR10_20101214.fasta	34,648.50	100.00%	3	3	5	0.02%	18.30%	ENVASHLQK	99.70%	37.8	28.1	1.76	1,807.90
ELF4-HFC Col rep 3	| Symbols: PCL1, LUX | Homeodomain-like superfamily protein | chr3:17183248-17184219 FORWARD LENGTH=323	AT3G46640.1,AT3G46640.2	TAIR10_20101214.fasta	34,648.50	100.00%	3	3	5	0.02%	18.30%	FVDVVAHLGIK	99.70%	28.6	25	23.2	1,668.78
ELF4-HFC Col rep 3	| Symbols: PCL1, LUX | Homeodomain-like superfamily protein | chr3:17183248-17184219 FORWARD LENGTH=323	AT3G46640.1,AT3G46640.2	TAIR10_20101214.fasta	34,648.50	100.00%	3	3	5	0.02%	18.30%	GGSSGPNTSSSNNNVEEEDR	99.70%	95.8	25	36.8	1,137.55
ELF4-HFC Col rep 3	| Symbols: PCL1, LUX | Homeodomain-like superfamily protein | chr3:17183248-17184219 FORWARD LENGTH=323	AT3G46640.1,AT3G46640.2	TAIR10_20101214.fasta	34,648.50	100.00%	3	3	5	0.02%	18.30%	LVWTPQLHK	97.60%	25.5	25.4	21.1	1,149.59
ELF4-HFC Col rep 3	| Symbols: PHYA, FHY2, FRE1, HY8 | phytochrome A | chr1:3095498-3099216 REVERSE LENGTH=1122	AT1G09570.1	TAIR10_20101214.fasta	124,503.20	100.00%	7	7	8	0.04%	9.27%	ADAGPISLVVNACASR	99.70%	74.7	29.4	98.3	1,915.08
ELF4-HFC Col rep 3	| Symbols: PHYA, FHY2, FRE1, HY8 | phytochrome A | chr1:3095498-3099216 REVERSE LENGTH=1122	AT1G09570.1	TAIR10_20101214.fasta	124,503.20	100.00%	7	7	8	0.04%	9.27%	ALGFGDVSLLNPILVHCR	99.70%	43	28.1	40.4	1,535.79
ELF4-HFC Col rep 3	| Symbols: PHYA, FHY2, FRE1, HY8 | phytochrome A | chr1:3095498-3099216 REVERSE LENGTH=1122	AT1G09570.1	TAIR10_20101214.fasta	124,503.20	100.00%	7	7	8	0.04%	9.27%	LIETATVPILAVDSDGLVNGWNTK	99.70%	48.7	30.1	57.8	1,178.55
ELF4-HFC Col rep 3	| Symbols: PHYA, FHY2, FRE1, HY8 | phytochrome A | chr1:3095498-3099216 REVERSE LENGTH=1122	AT1G09570.1	TAIR10_20101214.fasta	124,503.20	100.00%	7	7	8	0.04%	9.27%	LIQPFGCLLALDEK	99.70%	49.5	29.9	104	1,912.94
ELF4-HFC Col rep 3	| Symbols: PHYA, FHY2, FRE1, HY8 | phytochrome A | chr1:3095498-3099216 REVERSE LENGTH=1122	AT1G09570.1	TAIR10_20101214.fasta	124,503.20	100.00%	7	7	8	0.04%	9.27%	SSFIITAELAAANK	99.70%	74.2	29.2	84.1	1,618.77
ELF4-HFC Col rep 3	| Symbols: PHYA, FHY2, FRE1, HY8 | phytochrome A | chr1:3095498-3099216 REVERSE LENGTH=1122	AT1G09570.1	TAIR10_20101214.fasta	124,503.20	100.00%	7	7	8	0.04%	9.27%	VSFAFFTR	99.00%	53.8	29.6	59	1,695.79
ELF4-HFC Col rep 3	| Symbols: PHYA, FHY2, FRE1, HY8 | phytochrome A | chr1:3095498-3099216 REVERSE LENGTH=1122	AT1G09570.1	TAIR10_20101214.fasta	124,503.20	100.00%	7	7	8	0.04%	9.27%	YVECLLCVSK	99.70%	40.2	28.2	45.8	3,332.53
ELF4-HFC Col rep 3	| Symbols: PHYB, HY3, OOP1 | phytochrome B | chr2:8140079-8144151 FORWARD LENGTH=1172	AT2G18790.1	TAIR10_20101214.fasta	129,333.90	100.00%	22	32	58	0.29%	36.10%	AIQQYTVDAR	99.70%	52	29.9	27.4	2,224.04
ELF4-HFC Col rep 3	| Symbols: PHYB, HY3, OOP1 | phytochrome B | chr2:8140079-8144151 FORWARD LENGTH=1172	AT2G18790.1	TAIR10_20101214.fasta	129,333.90	100.00%	22	32	58	0.29%	36.10%	AISQLQALPGGDIK	99.70%	96.9	26.2	34.4	1,358.76
ELF4-HFC Col rep 3	| Symbols: PHYB, HY3, OOP1 | phytochrome B | chr2:8140079-8144151 FORWARD LENGTH=1172	AT2G18790.1	TAIR10_20101214.fasta	129,333.90	100.00%	22	32	58	0.29%	36.10%	AVFVVVNACSSK	99.70%	73	29.5	52.9	1,051.52
ELF4-HFC Col rep 3	| Symbols: PHYB, HY3, OOP1 | phytochrome B | chr2:8140079-8144151 FORWARD LENGTH=1172	AT2G18790.1	TAIR10_20101214.fasta	129,333.90	100.00%	22	32	58	0.29%	36.10%	DDLEPYIGLHYPATDIPQASR	96.40%	24.1	31.2	119	2,302.99
ELF4-HFC Col rep 3	| Symbols: PHYB, HY3, OOP1 | phytochrome B | chr2:8140079-8144151 FORWARD LENGTH=1172	AT2G18790.1	TAIR10_20101214.fasta	129,333.90	100.00%	22	32	58	0.29%	36.10%	DYLNNIVGVCFVGQDVTSQK	99.70%	101	31.4	34.7	1,513.82
ELF4-HFC Col rep 3	| Symbols: PHYB, HY3, OOP1 | phytochrome B | chr2:8140079-8144151 FORWARD LENGTH=1172	AT2G18790.1	TAIR10_20101214.fasta	129,333.90	100.00%	22	32	58	0.29%	36.10%	EITLLNPVWIHSK	99.70%	46.5	26.9	14.6	944.516
ELF4-HFC Col rep 3	| Symbols: PHYB, HY3, OOP1 | phytochrome B | chr2:8140079-8144151 FORWARD LENGTH=1172	AT2G18790.1	TAIR10_20101214.fasta	129,333.90	100.00%	22	32	58	0.29%	36.10%	ELAYICQVIK	99.30%	36.3	28.9	70.8	2,459.09
ELF4-HFC Col rep 3	| Symbols: PHYB, HY3, OOP1 | phytochrome B | chr2:8140079-8144151 FORWARD LENGTH=1172	AT2G18790.1	TAIR10_20101214.fasta	129,333.90	100.00%	22	32	58	0.29%	36.10%	FANSLLEATDLNEDQK	99.70%	120	30.5	0.11	1,540.74
ELF4-HFC Col rep 3	| Symbols: PHYB, HY3, OOP1 | phytochrome B | chr2:8140079-8144151 FORWARD LENGTH=1172	AT2G18790.1	TAIR10_20101214.fasta	129,333.90	100.00%	22	32	58	0.29%	36.10%	FHEDEHGEVVAESK	99.70%	122	27.2	122	2,146.99
ELF4-HFC Col rep 3	| Symbols: PHYB, HY3, OOP1 | phytochrome B | chr2:8140079-8144151 FORWARD LENGTH=1172	AT2G18790.1	TAIR10_20101214.fasta	129,333.90	100.00%	22	32	58	0.29%	36.10%	FINIQGDYK	99.70%	39.6	27.9	39	1,064.56
ELF4-HFC Col rep 3	| Symbols: PHYB, HY3, OOP1 | phytochrome B | chr2:8140079-8144151 FORWARD LENGTH=1172	AT2G18790.1	TAIR10_20101214.fasta	129,333.90	100.00%	22	32	58	0.29%	36.10%	IVGDMDLESIEDGSFVLK	99.70%	89.6	31.2	20.2	1,025.54
ELF4-HFC Col rep 3	| Symbols: PHYB, HY3, OOP1 | phytochrome B | chr2:8140079-8144151 FORWARD LENGTH=1172	AT2G18790.1	TAIR10_20101214.fasta	129,333.90	100.00%	22	32	58	0.29%	36.10%	LHAVFEQSGESGK	99.70%	69	29.2	14.1	1,197.70
ELF4-HFC Col rep 3	| Symbols: PHYB, HY3, OOP1 | phytochrome B | chr2:8140079-8144151 FORWARD LENGTH=1172	AT2G18790.1	TAIR10_20101214.fasta	129,333.90	100.00%	22	32	58	0.29%	36.10%	LIETATVPIFAVDAGGCINGWNAK	99.70%	56.9	31.2	89.4	2,036.84
ELF4-HFC Col rep 3	| Symbols: PHYB, HY3, OOP1 | phytochrome B | chr2:8140079-8144151 FORWARD LENGTH=1172	AT2G18790.1	TAIR10_20101214.fasta	129,333.90	100.00%	22	32	58	0.29%	36.10%	LLCDTVVESVR	99.70%	63.7	30.6	14.5	1,121.65
ELF4-HFC Col rep 3	| Symbols: PHYB, HY3, OOP1 | phytochrome B | chr2:8140079-8144151 FORWARD LENGTH=1172	AT2G18790.1	TAIR10_20101214.fasta	129,333.90	100.00%	22	32	58	0.29%	36.10%	MIVDCNATPVLVVQDDR	99.70%	118	30.4	65.9	1,600.81
ELF4-HFC Col rep 3	| Symbols: PHYB, HY3, OOP1 | phytochrome B | chr2:8140079-8144151 FORWARD LENGTH=1172	AT2G18790.1	TAIR10_20101214.fasta	129,333.90	100.00%	22	32	58	0.29%	36.10%	NTGKPFYAILHR	99.70%	44.3	29.6	38.1	1,981.07
ELF4-HFC Col rep 3	| Symbols: PHYB, HY3, OOP1 | phytochrome B | chr2:8140079-8144151 FORWARD LENGTH=1172	AT2G18790.1	TAIR10_20101214.fasta	129,333.90	100.00%	22	32	58	0.29%	36.10%	QLLETSVSCEK	99.70%	64.2	30.6	8.69	2,527.33
ELF4-HFC Col rep 3	| Symbols: PHYB, HY3, OOP1 | phytochrome B | chr2:8140079-8144151 FORWARD LENGTH=1172	AT2G18790.1	TAIR10_20101214.fasta	129,333.90	100.00%	22	32	58	0.29%	36.10%	RDDLEPYIGLHYPATDIPQASR	99.70%	49.9	31.4	35	1,616.87
ELF4-HFC Col rep 3	| Symbols: PHYB, HY3, OOP1 | phytochrome B | chr2:8140079-8144151 FORWARD LENGTH=1172	AT2G18790.1	TAIR10_20101214.fasta	129,333.90	100.00%	22	32	58	0.29%	36.10%	RQDTECFTK	99.70%	31.6	26.1	63.6	1,435.78
ELF4-HFC Col rep 3	| Symbols: PHYB, HY3, OOP1 | phytochrome B | chr2:8140079-8144151 FORWARD LENGTH=1172	AT2G18790.1	TAIR10_20101214.fasta	129,333.90	100.00%	22	32	58	0.29%	36.10%	SFDYSQSLK	99.70%	43.6	26.6	42.3	974.5093
ELF4-HFC Col rep 3	| Symbols: PHYB, HY3, OOP1 | phytochrome B | chr2:8140079-8144151 FORWARD LENGTH=1172	AT2G18790.1	TAIR10_20101214.fasta	129,333.90	100.00%	22	32	58	0.29%	36.10%	SIEVFGDQIR	99.70%	44.7	30.2	32.2	1,270.62
ELF4-HFC Col rep 3	| Symbols: PHYB, HY3, OOP1 | phytochrome B | chr2:8140079-8144151 FORWARD LENGTH=1172	AT2G18790.1	TAIR10_20101214.fasta	129,333.90	100.00%	22	32	58	0.29%	36.10%	SLFTSSSSILLER	99.70%	75.6	28.2	43.2	1,164.60
ELF4-HFC Col rep 3	| Symbols: PHYB, HY3, OOP1 | phytochrome B | chr2:8140079-8144151 FORWARD LENGTH=1172	AT2G18790.1	TAIR10_20101214.fasta	129,333.90	100.00%	22	32	58	0.29%	36.10%	SLVSDLIYK	99.70%	46.5	26.3	87	1,410.80
ELF4-HFC Col rep 3	| Symbols: PHYB, HY3, OOP1 | phytochrome B | chr2:8140079-8144151 FORWARD LENGTH=1172	AT2G18790.1	TAIR10_20101214.fasta	129,333.90	100.00%	22	32	58	0.29%	36.10%	SLVSDLIYKENEATVNK	99.70%	55.9	30.4	61.7	1,280.67
ELF4-HFC Col rep 3	| Symbols: PHYB, HY3, OOP1 | phytochrome B | chr2:8140079-8144151 FORWARD LENGTH=1172	AT2G18790.1	TAIR10_20101214.fasta	129,333.90	100.00%	22	32	58	0.29%	36.10%	SSFQAFLEVVK	99.70%	67	26.8	0	2,371.16
ELF4-HFC Col rep 3	| Symbols: PHYB, HY3, OOP1 | phytochrome B | chr2:8140079-8144151 FORWARD LENGTH=1172	AT2G18790.1	TAIR10_20101214.fasta	129,333.90	100.00%	22	32	58	0.29%	36.10%	TEDPALSIAGAVQSQK	99.70%	107	29.4	0	2,257.08
ELF4-HFC Col rep 3	| Symbols: PHYB, HY3, OOP1 | phytochrome B | chr2:8140079-8144151 FORWARD LENGTH=1172	AT2G18790.1	TAIR10_20101214.fasta	129,333.90	100.00%	22	32	58	0.29%	36.10%	TFSPELQGK	99.70%	35.6	30	34.2	1,549.87
ELF4-HFC Col rep 3	| Symbols: PHYB, HY3, OOP1 | phytochrome B | chr2:8140079-8144151 FORWARD LENGTH=1172	AT2G18790.1	TAIR10_20101214.fasta	129,333.90	100.00%	22	32	58	0.29%	36.10%	TTTYGSSVPEQQITAYLSR	99.70%	55.5	31	20.5	1,236.67
ELF4-HFC Col rep 3	| Symbols: PHYB, HY3, OOP1 | phytochrome B | chr2:8140079-8144151 FORWARD LENGTH=1172	AT2G18790.1	TAIR10_20101214.fasta	129,333.90	100.00%	22	32	58	0.29%	36.10%	VIGAFCFLQIPSPELQQALAVQR	99.70%	51.7	28.4	75.9	1,808.86
ELF4-HFC Col rep 3	| Symbols: PHYB, HY3, OOP1 | phytochrome B | chr2:8140079-8144151 FORWARD LENGTH=1172	AT2G18790.1	TAIR10_20101214.fasta	129,333.90	100.00%	22	32	58	0.29%	36.10%	VVDGVVQPCR	99.70%	55	27.4	117	1,612.72
ELF4-HFC Col rep 3	| Symbols: PHYB, HY3, OOP1 | phytochrome B | chr2:8140079-8144151 FORWARD LENGTH=1172	AT2G18790.1	TAIR10_20101214.fasta	129,333.90	100.00%	22	32	58	0.29%	36.10%	YAPSQEWVEIHLSQLSK	99.70%	47.5	30.7	26.6	1,097.56
ELF4-HFC Col rep 3	| Symbols: PHYB, HY3, OOP1 | phytochrome B | chr2:8140079-8144151 FORWARD LENGTH=1172	AT2G18790.1	TAIR10_20101214.fasta	129,333.90	100.00%	22	32	58	0.29%	36.10%	YYPLGVAPSEVQIK	99.70%	68.1	29.6	74.7	1,982.96
ELF4-HFC Col rep 3	| Symbols: PHYC | phytochrome C | chr5:14008049-14011619 FORWARD LENGTH=1111	AT5G35840.1	TAIR10_20101214.fasta	123,724.60	100.00%	29	41	55	0.27%	34.30%	AAEVTGLAVEQAIGKPVSDLVEDDSVETVK	99.70%	77.1	30.7	61	1,388.68
ELF4-HFC Col rep 3	| Symbols: PHYC | phytochrome C | chr5:14008049-14011619 FORWARD LENGTH=1111	AT5G35840.1	TAIR10_20101214.fasta	123,724.60	100.00%	29	41	55	0.27%	34.30%	AISFLQDLLHSSGLSEDQK	99.70%	105	31	51.8	2,517.28
ELF4-HFC Col rep 3	| Symbols: PHYC | phytochrome C | chr5:14008049-14011619 FORWARD LENGTH=1111	AT5G35840.1	TAIR10_20101214.fasta	123,724.60	100.00%	29	41	55	0.27%	34.30%	AISFLQDLLHSSGLSEDQKR	99.70%	39.4	30.4	47.6	1,290.67
ELF4-HFC Col rep 3	| Symbols: PHYC | phytochrome C | chr5:14008049-14011619 FORWARD LENGTH=1111	AT5G35840.1	TAIR10_20101214.fasta	123,724.60	100.00%	29	41	55	0.27%	34.30%	AVDFGEISILNPITLHCR	99.70%	115	30	105	1,944.95
ELF4-HFC Col rep 3	| Symbols: PHYC | phytochrome C | chr5:14008049-14011619 FORWARD LENGTH=1111	AT5G35840.1	TAIR10_20101214.fasta	123,724.60	100.00%	29	41	55	0.27%	34.30%	CDGAALYYR	99.70%	62.8	25	37.9	1,416.77
ELF4-HFC Col rep 3	| Symbols: PHYC | phytochrome C | chr5:14008049-14011619 FORWARD LENGTH=1111	AT5G35840.1	TAIR10_20101214.fasta	123,724.60	100.00%	29	41	55	0.27%	34.30%	DGSFIEALLSANK	99.70%	84.7	30.2	57	1,293.64
ELF4-HFC Col rep 3	| Symbols: PHYC | phytochrome C | chr5:14008049-14011619 FORWARD LENGTH=1111	AT5G35840.1	TAIR10_20101214.fasta	123,724.60	100.00%	29	41	55	0.27%	34.30%	DGSFIEALLSANKR	99.70%	32.8	29.7	0	2,527.26
ELF4-HFC Col rep 3	| Symbols: PHYC | phytochrome C | chr5:14008049-14011619 FORWARD LENGTH=1111	AT5G35840.1	TAIR10_20101214.fasta	123,724.60	100.00%	29	41	55	0.27%	34.30%	DLIDWVLK	99.00%	47.4	28.8	5.58	1,184.54
ELF4-HFC Col rep 3	| Symbols: PHYC | phytochrome C | chr5:14008049-14011619 FORWARD LENGTH=1111	AT5G35840.1	TAIR10_20101214.fasta	123,724.60	100.00%	29	41	55	0.27%	34.30%	DNLWSLGVTPTETQIR	99.70%	47	30.5	31.1	1,074.51
ELF4-HFC Col rep 3	| Symbols: PHYC | phytochrome C | chr5:14008049-14011619 FORWARD LENGTH=1111	AT5G35840.1	TAIR10_20101214.fasta	123,724.60	100.00%	29	41	55	0.27%	34.30%	EAESAVLLK	99.70%	47	30.5	25.6	1,163.61
ELF4-HFC Col rep 3	| Symbols: PHYC | phytochrome C | chr5:14008049-14011619 FORWARD LENGTH=1111	AT5G35840.1	TAIR10_20101214.fasta	123,724.60	100.00%	29	41	55	0.27%	34.30%	EALTIGTDVK	99.70%	36.5	30	69.9	1,439.77
ELF4-HFC Col rep 3	| Symbols: PHYC | phytochrome C | chr5:14008049-14011619 FORWARD LENGTH=1111	AT5G35840.1	TAIR10_20101214.fasta	123,724.60	100.00%	29	41	55	0.27%	34.30%	EGLGLHITQK	99.70%	53.4	25.9	30	1,037.59
ELF4-HFC Col rep 3	| Symbols: PHYC | phytochrome C | chr5:14008049-14011619 FORWARD LENGTH=1111	AT5G35840.1	TAIR10_20101214.fasta	123,724.60	100.00%	29	41	55	0.27%	34.30%	EVSELTGYDR	99.70%	39.9	27.7	49.6	1,923.01
ELF4-HFC Col rep 3	| Symbols: PHYC | phytochrome C | chr5:14008049-14011619 FORWARD LENGTH=1111	AT5G35840.1	TAIR10_20101214.fasta	123,724.60	100.00%	29	41	55	0.27%	34.30%	HLWGLVVCHHASPR	98.90%	28	29.9	51.3	1,254.67
ELF4-HFC Col rep 3	| Symbols: PHYC | phytochrome C | chr5:14008049-14011619 FORWARD LENGTH=1111	AT5G35840.1	TAIR10_20101214.fasta	123,724.60	100.00%	29	41	55	0.27%	34.30%	IEEGLVIDLEPVSPDEVPVTAAGALR	99.70%	63	28.9	93.8	1,614.83
ELF4-HFC Col rep 3	| Symbols: PHYC | phytochrome C | chr5:14008049-14011619 FORWARD LENGTH=1111	AT5G35840.1	TAIR10_20101214.fasta	123,724.60	100.00%	29	41	55	0.27%	34.30%	IGFNAVISGQK	99.70%	66.8	28.6	29.8	1,006.52
ELF4-HFC Col rep 3	| Symbols: PHYC | phytochrome C | chr5:14008049-14011619 FORWARD LENGTH=1111	AT5G35840.1	TAIR10_20101214.fasta	123,724.60	100.00%	29	41	55	0.27%	34.30%	IIHPAPGLPEDLVR	99.70%	49.3	26	53.8	2,102.04
ELF4-HFC Col rep 3	| Symbols: PHYC | phytochrome C | chr5:14008049-14011619 FORWARD LENGTH=1111	AT5G35840.1	TAIR10_20101214.fasta	123,724.60	100.00%	29	41	55	0.27%	34.30%	ILLGEVFTTDDYGCCLK	99.70%	71.9	30.2	49.5	2,585.39
ELF4-HFC Col rep 3	| Symbols: PHYC | phytochrome C | chr5:14008049-14011619 FORWARD LENGTH=1111	AT5G35840.1	TAIR10_20101214.fasta	123,724.60	100.00%	29	41	55	0.27%	34.30%	LHGNFEESER	99.70%	35.4	25.8	52	1,128.58
ELF4-HFC Col rep 3	| Symbols: PHYC | phytochrome C | chr5:14008049-14011619 FORWARD LENGTH=1111	AT5G35840.1	TAIR10_20101214.fasta	123,724.60	100.00%	29	41	55	0.27%	34.30%	LIDTAAVPIFAVDASGVINGWNSK	99.70%	88.6	30.7	43.5	2,015.02
ELF4-HFC Col rep 3	| Symbols: PHYC | phytochrome C | chr5:14008049-14011619 FORWARD LENGTH=1111	AT5G35840.1	TAIR10_20101214.fasta	123,724.60	100.00%	29	41	55	0.27%	34.30%	LLFGFYHR	99.00%	37	27.6	60.5	1,563.84
ELF4-HFC Col rep 3	| Symbols: PHYC | phytochrome C | chr5:14008049-14011619 FORWARD LENGTH=1111	AT5G35840.1	TAIR10_20101214.fasta	123,724.60	100.00%	29	41	55	0.27%	34.30%	LQQILSETLLSSIR	99.70%	113	26	75.9	3,069.58
ELF4-HFC Col rep 3	| Symbols: PHYC | phytochrome C | chr5:14008049-14011619 FORWARD LENGTH=1111	AT5G35840.1	TAIR10_20101214.fasta	123,724.60	100.00%	29	41	55	0.27%	34.30%	NAPIGIVTQSPNIMDLVK	99.70%	70.8	29	98.9	2,088.06
ELF4-HFC Col rep 3	| Symbols: PHYC | phytochrome C | chr5:14008049-14011619 FORWARD LENGTH=1111	AT5G35840.1	TAIR10_20101214.fasta	123,724.60	100.00%	29	41	55	0.27%	34.30%	SSPVELVVNTCCSR	99.70%	56	29.1	34.5	2,244.16
ELF4-HFC Col rep 3	| Symbols: PHYC | phytochrome C | chr5:14008049-14011619 FORWARD LENGTH=1111	AT5G35840.1	TAIR10_20101214.fasta	123,724.60	100.00%	29	41	55	0.27%	34.30%	SSSKPFYAILHR	99.70%	49.6	29.5	106	2,055.07
ELF4-HFC Col rep 3	| Symbols: PHYC | phytochrome C | chr5:14008049-14011619 FORWARD LENGTH=1111	AT5G35840.1	TAIR10_20101214.fasta	123,724.60	100.00%	29	41	55	0.27%	34.30%	TLTENYSR	99.00%	48.5	27.7	54.1	1,088.48
ELF4-HFC Col rep 3	| Symbols: PHYC | phytochrome C | chr5:14008049-14011619 FORWARD LENGTH=1111	AT5G35840.1	TAIR10_20101214.fasta	123,724.60	100.00%	29	41	55	0.27%	34.30%	TVVDVPLVDNR	99.70%	52.2	26.9	69.1	1,364.71

ELF4-HFC Col rep 3	| Symbols: PHYC | phytochrome C | chr5:14008049-14011619 FORWARD LENGTH=1111	AT5G35840.1	TAIR10_20101214.fasta	123,724.60	100.00%	29	41	55	0.27%	34.30%	VSSQVLVDAK	99.70%	67.7	29.5	24.3	1,520.81
ELF4-HFC Col rep 3	| Symbols: PHYC | phytochrome C | chr5:14008049-14011619 FORWARD LENGTH=1111	AT5G35840.1	TAIR10_20101214.fasta	123,724.60	100.00%	29	41	55	0.27%	34.30%	YACEFLTQVFGVQINK	99.70%	76.2	30.6	23.3	1,001.57
ELF4-HFC Col rep 3	| Symbols: PHYD | phytochrome D | chr4:9195602-9199486 REVERSE LENGTH=1164	AT4G16250.1	TAIR10_20101214.fasta	129,270.30	99.90%	1	1	17	0.08%	9.88%	AIQQYTVDAR	99.70%	52	29.9	34.5	1,829.94
ELF4-HFC Col rep 3	| Symbols: PHYD | phytochrome D | chr4:9195602-9199486 REVERSE LENGTH=1164	AT4G16250.1	TAIR10_20101214.fasta	129,270.30	99.90%	1	1	17	0.08%	9.88%	FHEDEHGEVVAESK	99.70%	122	27.2	23.7	959.5408
ELF4-HFC Col rep 3	| Symbols: PHYD | phytochrome D | chr4:9195602-9199486 REVERSE LENGTH=1164	AT4G16250.1	TAIR10_20101214.fasta	129,270.30	99.90%	1	1	17	0.08%	9.88%	FINIQGDYK	99.70%	39.6	27.9	24.9	1,046.57
ELF4-HFC Col rep 3	| Symbols: PHYD | phytochrome D | chr4:9195602-9199486 REVERSE LENGTH=1164	AT4G16250.1	TAIR10_20101214.fasta	129,270.30	99.90%	1	1	17	0.08%	9.88%	LHAVFEQSGESGK	99.70%	69	29.2	39.8	1,095.62
ELF4-HFC Col rep 3	| Symbols: PHYD | phytochrome D | chr4:9195602-9199486 REVERSE LENGTH=1164	AT4G16250.1	TAIR10_20101214.fasta	129,270.30	99.90%	1	1	17	0.08%	9.88%	LLCDTVVESVR	99.70%	63.7	30.6	26.3	1,168.55
ELF4-HFC Col rep 3	| Symbols: PHYD | phytochrome D | chr4:9195602-9199486 REVERSE LENGTH=1164	AT4G16250.1	TAIR10_20101214.fasta	129,270.30	99.90%	1	1	17	0.08%	9.88%	NDLEPYIGLHYPATDIPQASR	99.70%	40.1	31.2	11.2	1,668.85
ELF4-HFC Col rep 3	| Symbols: PHYD | phytochrome D | chr4:9195602-9199486 REVERSE LENGTH=1164	AT4G16250.1	TAIR10_20101214.fasta	129,270.30	99.90%	1	1	17	0.08%	9.88%	QLLETSVSCEK	99.70%	64.2	30.6	55.2	2,689.43
ELF4-HFC Col rep 3	| Symbols: PHYD | phytochrome D | chr4:9195602-9199486 REVERSE LENGTH=1164	AT4G16250.1	TAIR10_20101214.fasta	129,270.30	99.90%	1	1	17	0.08%	9.88%	RNDLEPYIGLHYPATDIPQASR	99.70%	49.9	31.4	52	1,133.63
ELF4-HFC Col rep 3	| Symbols: PHYD | phytochrome D | chr4:9195602-9199486 REVERSE LENGTH=1164	AT4G16250.1	TAIR10_20101214.fasta	129,270.30	99.90%	1	1	17	0.08%	9.88%	SFDYSQSLK	99.70%	43.6	26.6	43.7	1,526.87
ELF4-HFC Col rep 3	| Symbols: PHYD | phytochrome D | chr4:9195602-9199486 REVERSE LENGTH=1164	AT4G16250.1	TAIR10_20101214.fasta	129,270.30	99.90%	1	1	17	0.08%	9.88%	TEDPALSIAGAVQSQK	99.70%	107	29.4	71.3	2,003.95
ELF4-HFC Col rep 3	| Symbols: PHYE | phytochrome E | chr4:10042312-10045948 REVERSE LENGTH=1112	AT4G18130.1	TAIR10_20101214.fasta	122,519.10	100.00%	16	18	22	0.11%	21.60%	AALESLLCK	99.70%	45.5	30.2	24.3	1,217.55
ELF4-HFC Col rep 3	| Symbols: PHYE | phytochrome E | chr4:10042312-10045948 REVERSE LENGTH=1112	AT4G18130.1	TAIR10_20101214.fasta	122,519.10	100.00%	16	18	22	0.11%	21.60%	AASFTEISLLNPVLVHSR	99.70%	62.9	27	12	2,459.28
ELF4-HFC Col rep 3	| Symbols: PHYE | phytochrome E | chr4:10042312-10045948 REVERSE LENGTH=1112	AT4G18130.1	TAIR10_20101214.fasta	122,519.10	100.00%	16	18	22	0.11%	21.60%	DGWVTPDGLGLK	99.10%	32.1	27.7	26.6	1,052.57
ELF4-HFC Col rep 3	| Symbols: PHYE | phytochrome E | chr4:10042312-10045948 REVERSE LENGTH=1112	AT4G18130.1	TAIR10_20101214.fasta	122,519.10	100.00%	16	18	22	0.11%	21.60%	DYTENIIGVCFVGQDITSEK	99.70%	57.4	30.8	104	1,600.93
ELF4-HFC Col rep 3	| Symbols: PHYE | phytochrome E | chr4:10042312-10045948 REVERSE LENGTH=1112	AT4G18130.1	TAIR10_20101214.fasta	122,519.10	100.00%	16	18	22	0.11%	21.60%	ESAQSLNELTYVR	99.70%	61.3	30.6	70.8	1,926.04
ELF4-HFC Col rep 3	| Symbols: PHYE | phytochrome E | chr4:10042312-10045948 REVERSE LENGTH=1112	AT4G18130.1	TAIR10_20101214.fasta	122,519.10	100.00%	16	18	22	0.11%	21.60%	ESFTSSRPVLSGNGVAR	99.70%	38.9	31.6	50.2	1,607.75
ELF4-HFC Col rep 3	| Symbols: PHYE | phytochrome E | chr4:10042312-10045948 REVERSE LENGTH=1112	AT4G18130.1	TAIR10_20101214.fasta	122,519.10	100.00%	16	18	22	0.11%	21.60%	FGQNNHPDYSSDVCVLVNSCTSR	98.60%	23	27.4	39.5	1,405.76
ELF4-HFC Col rep 3	| Symbols: PHYE | phytochrome E | chr4:10042312-10045948 REVERSE LENGTH=1112	AT4G18130.1	TAIR10_20101214.fasta	122,519.10	100.00%	16	18	22	0.11%	21.60%	GGLVQPFGCLIAVEEPSFR	99.70%	71.7	30.9	4.82	983.4792
ELF4-HFC Col rep 3	| Symbols: PHYE | phytochrome E | chr4:10042312-10045948 REVERSE LENGTH=1112	AT4G18130.1	TAIR10_20101214.fasta	122,519.10	100.00%	16	18	22	0.11%	21.60%	LLESSEISASQR	99.70%	73.8	30.7	45.2	1,226.67
ELF4-HFC Col rep 3	| Symbols: PHYE | phytochrome E | chr4:10042312-10045948 REVERSE LENGTH=1112	AT4G18130.1	TAIR10_20101214.fasta	122,519.10	100.00%	16	18	22	0.11%	21.60%	NIVNHAPFPNSWVGISISPGQELSR	99.70%	57.7	31.1	54.1	1,045.59
ELF4-HFC Col rep 3	| Symbols: PHYE | phytochrome E | chr4:10042312-10045948 REVERSE LENGTH=1112	AT4G18130.1	TAIR10_20101214.fasta	122,519.10	100.00%	16	18	22	0.11%	21.60%	QFLETSDACEK	99.70%	32.4	25.7	63.8	1,916.96
ELF4-HFC Col rep 3	| Symbols: PHYE | phytochrome E | chr4:10042312-10045948 REVERSE LENGTH=1112	AT4G18130.1	TAIR10_20101214.fasta	122,519.10	100.00%	16	18	22	0.11%	21.60%	QITTIIESTDLK	99.70%	82.6	28.9	43.2	1,164.60
ELF4-HFC Col rep 3	| Symbols: PHYE | phytochrome E | chr4:10042312-10045948 REVERSE LENGTH=1112	AT4G18130.1	TAIR10_20101214.fasta	122,519.10	100.00%	16	18	22	0.11%	21.60%	SGDPALTLAGAVQSQK	99.70%	50.7	29.2	117	1,612.72
ELF4-HFC Col rep 3	| Symbols: PHYE | phytochrome E | chr4:10042312-10045948 REVERSE LENGTH=1112	AT4G18130.1	TAIR10_20101214.fasta	122,519.10	100.00%	16	18	22	0.11%	21.60%	SLADEIVQEESR	99.70%	71.4	29.4	26.6	1,097.56
ELF4-HFC Col rep 3	| Symbols: PHYE | phytochrome E | chr4:10042312-10045948 REVERSE LENGTH=1112	AT4G18130.1	TAIR10_20101214.fasta	122,519.10	100.00%	16	18	22	0.11%	21.60%	SSFTAFLEVAK	99.70%	56.9	27.3	61	1,388.68
ELF4-HFC Col rep 3	| Symbols: PHYE | phytochrome E | chr4:10042312-10045948 REVERSE LENGTH=1112	AT4G18130.1	TAIR10_20101214.fasta	122,519.10	100.00%	16	18	22	0.11%	21.60%	YIEASLTANK	99.70%	45.2	28.8	47.6	1,290.67
ELF4-HFC Col rep 3	| Symbols: SPA1 | SPA (suppressor of phyA-105) protein family | chr2:19022572-19026821 REVERSE LENGTH=1029	AT2G46340.1	TAIR10_20101214.fasta	114,689.10	100.00%	6	6	7	0.03%	10.00%	NDLESDVDEDLNR	99.70%	58.7	25.5	3.6	2,371.16
ELF4-HFC Col rep 3	| Symbols: SPA1 | SPA (suppressor of phyA-105) protein family | chr2:19022572-19026821 REVERSE LENGTH=1029	AT2G46340.1	TAIR10_20101214.fasta	114,689.10	100.00%	6	6	7	0.03%	10.00%	NQETPPEFVSDQDLGSK	99.70%	48.8	29.1	57	1,293.64
ELF4-HFC Col rep 3	| Symbols: SPA1 | SPA (suppressor of phyA-105) protein family | chr2:19022572-19026821 REVERSE LENGTH=1029	AT2G46340.1	TAIR10_20101214.fasta	114,689.10	100.00%	6	6	7	0.03%	10.00%	NYLASTDYDGVVQIWDAGTGQGFSQYTEHQK	99.70%	113	30.6	0	2,527.26
ELF4-HFC Col rep 3	| Symbols: SPA1 | SPA (suppressor of phyA-105) protein family | chr2:19022572-19026821 REVERSE LENGTH=1029	AT2G46340.1	TAIR10_20101214.fasta	114,689.10	100.00%	6	6	7	0.03%	10.00%	SSDQLEVFFEGLCK	99.70%	70.2	28.8	31.1	1,074.51
ELF4-HFC Col rep 3	| Symbols: SPA1 | SPA (suppressor of phyA-105) protein family | chr2:19022572-19026821 REVERSE LENGTH=1029	AT2G46340.1	TAIR10_20101214.fasta	114,689.10	100.00%	6	6	7	0.03%	10.00%	TPVSSSNFSQLLLK	99.70%	92.8	28.6	93.8	1,614.83
ELF4-HFC Col rep 3	| Symbols: SPA1 | SPA (suppressor of phyA-105) protein family | chr2:19022572-19026821 REVERSE LENGTH=1029	AT2G46340.1	TAIR10_20101214.fasta	114,689.10	100.00%	6	6	7	0.03%	10.00%	VAEETVATNNIQLK	99.70%	56.7	29.9	34	1,004.54
ELF4-HFC Col rep 3	| Symbols: TIC | time for coffee | chr3:7913181-7918879 FORWARD LENGTH=1550	AT3G22380.1,AT3G22380.2	TAIR10_20101214.fasta	164,452.80	100.00%	3	3	5	0.02%	3.35%	DSPEEEGPVELPEAAR	99.70%	55.8	29.1	48.9	1,954.08
ELF4-HFC Col rep 3	| Symbols: TIC | time for coffee | chr3:7913181-7918879 FORWARD LENGTH=1550	AT3G22380.1,AT3G22380.2	TAIR10_20101214.fasta	164,452.80	100.00%	3	3	5	0.02%	3.35%	SSGSGEGNSSVLDSIIPLTR	99.70%	67.5	31.3	12.6	1,257.65
ELF4-HFC Col rep 3	| Symbols: TIC | time for coffee | chr3:7913181-7918879 FORWARD LENGTH=1550	AT3G22380.1,AT3G22380.2	TAIR10_20101214.fasta	164,452.80	100.00%	3	3	5	0.02%	3.35%	SSPSPAPVSPLVSTWK	99.70%	65.7	28.8	55.4	2,288.08
ELF4-HFC Col rep 3	| Symbols: TOC1, APRR1, PRR1, AtTOC1 | CCT motif -containing response regulator protein | chr5:24675540-24678176 FORWARD LENGTH=618	AT5G61380.1	TAIR10_20101214.fasta	69,195.40	100.00%	3	3	3	0.01%	9.22%	FQVVASEGINNTK	99.70%	75.3	30.2	52.6	1,509.75
ELF4-HFC Col rep 3	| Symbols: TOC1, APRR1, PRR1, AtTOC1 | CCT motif -containing response regulator protein | chr5:24675540-24678176 FORWARD LENGTH=618	AT5G61380.1	TAIR10_20101214.fasta	69,195.40	100.00%	3	3	3	0.01%	9.22%	GNLSHQENEWSVATAPVHAR	99.70%	40	30.9	34.1	1,764.89
ELF4-HFC Col rep 3	| Symbols: TOC1, APRR1, PRR1, AtTOC1 | CCT motif -containing response regulator protein | chr5:24675540-24678176 FORWARD LENGTH=618	AT5G61380.1	TAIR10_20101214.fasta	69,195.40	100.00%	3	3	3	0.01%	9.22%	GTEQYHSQGETLQNGASYPHSLER	97.90%	34.1	29.6	22.4	2,656.15
ELF4-HFC Col rep 3	| Symbols: TZP | zinc knuckle (CCHC-type) family protein | chr5:17527261-17530207 FORWARD LENGTH=831	AT5G43630.1	TAIR10_20101214.fasta	90,615.60	100.00%	18	22	27	0.13%	26.70%	AVFDAVQVLR	99.70%	77	28.4	65.9	2,076.06
ELF4-HFC Col rep 3	| Symbols: TZP | zinc knuckle (CCHC-type) family protein | chr5:17527261-17530207 FORWARD LENGTH=831	AT5G43630.1	TAIR10_20101214.fasta	90,615.60	100.00%	18	22	27	0.13%	26.70%	AWWQSAGK	99.00%	46.9	28.8	57.6	1,319.68
ELF4-HFC Col rep 3	| Symbols: TZP | zinc knuckle (CCHC-type) family protein | chr5:17527261-17530207 FORWARD LENGTH=831	AT5G43630.1	TAIR10_20101214.fasta	90,615.60	100.00%	18	22	27	0.13%	26.70%	CFQLSHWAATCPNAPLYGSGAEGR	99.70%	62.2	29.6	47.7	2,719.40
ELF4-HFC Col rep 3	| Symbols: TZP | zinc knuckle (CCHC-type) family protein | chr5:17527261-17530207 FORWARD LENGTH=831	AT5G43630.1	TAIR10_20101214.fasta	90,615.60	100.00%	18	22	27	0.13%	26.70%	DCLEVTDTELR	99.70%	76.5	28.1	11.8	1,327.58
ELF4-HFC Col rep 3	| Symbols: TZP | zinc knuckle (CCHC-type) family protein | chr5:17527261-17530207 FORWARD LENGTH=831	AT5G43630.1	TAIR10_20101214.fasta	90,615.60	100.00%	18	22	27	0.13%	26.70%	DLLVNEQLR	99.70%	42.3	26.1	64.5	1,361.75
ELF4-HFC Col rep 3	| Symbols: TZP | zinc knuckle (CCHC-type) family protein | chr5:17527261-17530207 FORWARD LENGTH=831	AT5G43630.1	TAIR10_20101214.fasta	90,615.60	100.00%	18	22	27	0.13%	26.70%	DLVQNISVR	99.70%	51.5	28.9	39.3	1,542.81
ELF4-HFC Col rep 3	| Symbols: TZP | zinc knuckle (CCHC-type) family protein | chr5:17527261-17530207 FORWARD LENGTH=831	AT5G43630.1	TAIR10_20101214.fasta	90,615.60	100.00%	18	22	27	0.13%	26.70%	EEASSLCIR	99.70%	45	28.6	61.5	1,375.67
ELF4-HFC Col rep 3	| Symbols: TZP | zinc knuckle (CCHC-type) family protein | chr5:17527261-17530207 FORWARD LENGTH=831	AT5G43630.1	TAIR10_20101214.fasta	90,615.60	100.00%	18	22	27	0.13%	26.70%	GDDLSSSGNEIGPVAEPNISSGK	99.70%	94.8	30.5	50.3	1,199.63
ELF4-HFC Col rep 3	| Symbols: TZP | zinc knuckle (CCHC-type) family protein | chr5:17527261-17530207 FORWARD LENGTH=831	AT5G43630.1	TAIR10_20101214.fasta	90,615.60	100.00%	18	22	27	0.13%	26.70%	GDYLPEGEAGPSGSYR	99.70%	59.9	27	36.6	1,109.58
ELF4-HFC Col rep 3	| Symbols: TZP | zinc knuckle (CCHC-type) family protein | chr5:17527261-17530207 FORWARD LENGTH=831	AT5G43630.1	TAIR10_20101214.fasta	90,615.60	100.00%	18	22	27	0.13%	26.70%	GVTCLVESQFISNQDFLEEELK	99.70%	99.7	31.5	51.3	1,533.67
ELF4-HFC Col rep 3	| Symbols: TZP | zinc knuckle (CCHC-type) family protein | chr5:17527261-17530207 FORWARD LENGTH=831	AT5G43630.1	TAIR10_20101214.fasta	90,615.60	100.00%	18	22	27	0.13%	26.70%	KRPGFEEQLIFGSK	99.70%	37.9	28.8	44.9	1,890.87
ELF4-HFC Col rep 3	| Symbols: TZP | zinc knuckle (CCHC-type) family protein | chr5:17527261-17530207 FORWARD LENGTH=831	AT5G43630.1	TAIR10_20101214.fasta	90,615.60	100.00%	18	22	27	0.13%	26.70%	LPISGFTDVPR	99.70%	73.4	29.4	3.1	3,479.57
ELF4-HFC Col rep 3	| Symbols: TZP | zinc knuckle (CCHC-type) family protein | chr5:17527261-17530207 FORWARD LENGTH=831	AT5G43630.1	TAIR10_20101214.fasta	90,615.60	100.00%	18	22	27	0.13%	26.70%	NGREEASSLCIR	99.70%	38.8	28.7	64.5	1,658.77
ELF4-HFC Col rep 3	| Symbols: TZP | zinc knuckle (CCHC-type) family protein | chr5:17527261-17530207 FORWARD LENGTH=831	AT5G43630.1	TAIR10_20101214.fasta	90,615.60	100.00%	18	22	27	0.13%	26.70%	NTGFQSFFQSIYCPK	99.70%	87.3	29.4	83.7	1,520.83
ELF4-HFC Col rep 3	| Symbols: TZP | zinc knuckle (CCHC-type) family protein | chr5:17527261-17530207 FORWARD LENGTH=831	AT5G43630.1	TAIR10_20101214.fasta	90,615.60	100.00%	18	22	27	0.13%	26.70%	RPGFEEQLIFGSK	99.70%	48.8	30	45.5	1,529.82
ELF4-HFC Col rep 3	| Symbols: TZP | zinc knuckle (CCHC-type) family protein | chr5:17527261-17530207 FORWARD LENGTH=831	AT5G43630.1	TAIR10_20101214.fasta	90,615.60	100.00%	18	22	27	0.13%	26.70%	SVSGLEGFFLR	99.70%	57.4	27.9	55.4	1,724.80
ELF4-HFC Col rep 3	| Symbols: TZP | zinc knuckle (CCHC-type) family protein | chr5:17527261-17530207 FORWARD LENGTH=831	AT5G43630.1	TAIR10_20101214.fasta	90,615.60	100.00%	18	22	27	0.13%	26.70%	TLNQECLGSTSK	99.70%	61.7	29.5	62.3	1,975.99
ELF4-HFC Col rep 3	| Symbols: TZP | zinc knuckle (CCHC-type) family protein | chr5:17527261-17530207 FORWARD LENGTH=831	AT5G43630.1	TAIR10_20101214.fasta	90,615.60	100.00%	18	22	27	0.13%	26.70%	TSGYDGIPSAEELSR	99.70%	54	29.8	57.2	1,639.87
ELF4-HFC Col rep 4	| Symbols: | F-box family protein | chr2:7074940-7079181 FORWARD LENGTH=727	AT2G16365.4	TAIR10_20101214.fasta	82,716.20	100.00%	14	16	19	0.10%	27.20%	ALSGLNPYGLR	99.70%	62.7	30.3	56.1	1,406.73
ELF4-HFC Col rep 4	| Symbols: | F-box family protein | chr2:7074940-7079181 FORWARD LENGTH=727	AT2G16365.4	TAIR10_20101214.fasta	82,716.20	100.00%	14	16	19	0.10%	27.20%	ENNNLFLEILK	99.70%	80.6	29.6	32.8	2,203.06
ELF4-HFC Col rep 4	| Symbols: | F-box family protein | chr2:7074940-7079181 FORWARD LENGTH=727	AT2G16365.4	TAIR10_20101214.fasta	82,716.20	100.00%	14	16	19	0.10%	27.20%	ETTTTTTTKPSFHEDVGSSSR	99.70%	57.1	30.5	1.63	2,690.21
ELF4-HFC Col rep 4	| Symbols: | F-box family protein | chr2:7074940-7079181 FORWARD LENGTH=727	AT2G16365.4	TAIR10_20101214.fasta	82,716.20	100.00%	14	16	19	0.10%	27.20%	FHDGETNCSK	99.00%	30.7	25	56.8	1,117.64
ELF4-HFC Col rep 4	| Symbols: | F-box family protein | chr2:7074940-7079181 FORWARD LENGTH=727	AT2G16365.4	TAIR10_20101214.fasta	82,716.20	100.00%	14	16	19	0.10%	27.20%	IFGGYLDLFPNQDHSHNR	99.70%	44.8	30.6	30.9	933.4577
ELF4-HFC Col rep 4	| Symbols: | F-box family protein | chr2:7074940-7079181 FORWARD LENGTH=727	AT2G16365.4	TAIR10_20101214.fasta	82,716.20	100.00%	14	16	19	0.10%	27.20%	IYATVDSVNLPK	99.70%	71.3	29	56.2	2,650.19
ELF4-HFC Col rep 4	| Symbols: | F-box family protein | chr2:7074940-7079181 FORWARD LENGTH=727	AT2G16365.4	TAIR10_20101214.fasta	82,716.20	100.00%	14	16	19	0.10%	27.20%	LDFPIQEK	99.00%	34.3	30.2	54.2	1,350.62
ELF4-HFC Col rep 4	| Symbols: | F-box family protein | chr2:7074940-7079181 FORWARD LENGTH=727	AT2G16365.4	TAIR10_20101214.fasta	82,716.20	100.00%	14	16	19	0.10%	27.20%	LQTIHLSGSISSSSTK	99.70%	78.1	30.5	19.2	1,099.61
ELF4-HFC Col rep 4	| Symbols: | F-box family protein | chr2:7074940-7079181 FORWARD LENGTH=727	AT2G16365.4	TAIR10_20101214.fasta	82,716.20	100.00%	14	16	19	0.10%	27.20%	NQEPVVSQSNDLR	99.70%	58.6	29.3	34.7	1,043.58
ELF4-HFC Col rep 4	| Symbols: | F-box family protein | chr2:7074940-7079181 FORWARD LENGTH=727	AT2G16365.4	TAIR10_20101214.fasta	82,716.20	100.00%	14	16	19	0.10%	27.20%	NSSVNPYESAWLGR	99.70%	72	30	32.1	1,064.50
ELF4-HFC Col rep 4	| Symbols: | F-box family protein | chr2:7074940-7079181 FORWARD LENGTH=727	AT2G16365.4	TAIR10_20101214.fasta	82,716.20	100.00%	14	16	19	0.10%	27.20%	QGGDDITLLHPWIQR	98.20%	28.2	30.6	89.4	2,230.05
ELF4-HFC Col rep 4	| Symbols: | F-box family protein | chr2:7074940-7079181 FORWARD LENGTH=727	AT2G16365.4	TAIR10_20101214.fasta	82,716.20	100.00%	14	16	19	0.10%	27.20%	TTQNLLELIRPVR	99.70%	30.9	25	54.1	1,654.73
ELF4-HFC Col rep 4	| Symbols: | F-box family protein | chr2:7074940-7079181 FORWARD LENGTH=727	AT2G16365.4	TAIR10_20101214.fasta	82,716.20	100.00%	14	16	19	0.10%	27.20%	TTSTDQPTGQEASFEPESHKEFEK	99.70%	35.9	28.9	95.7	2,585.24
ELF4-HFC Col rep 4	| Symbols: | F-box family protein | chr2:7074940-7079181 FORWARD LENGTH=727	AT2G16365.4	TAIR10_20101214.fasta	82,716.20	100.00%	14	16	19	0.10%	27.20%	VRLEPEVEASSR	99.70%	48.2	29.1	30.4	1,635.89
ELF4-HFC Col rep 4	| Symbols: | Granulin repeat cysteine protease family protein | chr3:6723024-6724768 FORWARD LENGTH=452	AT3G19390.1	TAIR10_20101214.fasta	49,308.20	100.00%	2	2	3	0.02%	7.08%	ALANQPISVAIEAGGR	99.70%	74.8	27.4	65.5	1,201.66
ELF4-HFC Col rep 4	| Symbols: | Granulin repeat cysteine protease family protein | chr3:6723024-6724768 FORWARD LENGTH=452	AT3G19390.1	TAIR10_20101214.fasta	49,308.20	100.00%	2	2	3	0.02%	7.08%	VVTIDGYEDVPQNDEK	99.40%	37.5	30.1	27.3	1,392.65
ELF4-HFC Col rep 4	| Symbols: | Protein kinase family protein | chr3:4469434-4473234 FORWARD LENGTH=703	AT3G13670.1	TAIR10_20101214.fasta	78,640.00	99.90%	1	1	3	0.02%	6.69%	LVSLFQDLLGENPAIRPINTEGAQK	99.70%	57.5	27.1	77.2	1,823.84
ELF4-HFC Col rep 4	| Symbols: | Protein kinase family protein | chr3:4469434-4473234 FORWARD LENGTH=703	AT3G13670.1	TAIR10_20101214.fasta	78,640.00	99.90%	1	1	3	0.02%	6.69%	NSGYSEQVVELDFLYPSEGIHR	99.60%	44.2	31.7	40.5	1,507.79
ELF4-HFC Col rep 4	| Symbols: ATAN11, LWD1 | Transducin/WD40 repeat-like superfamily protein | chr1:4395114-4396154 REVERSE LENGTH=346	AT1G12910.1	TAIR10_20101214.fasta	39,089.30	100.00%	3	3	6	0.03%	23.40%	DKEHSTIIYESSEPDTPLVR	99.70%	51.9	31.8	33.4	1,211.64
ELF4-HFC Col rep 4	| Symbols: ATAN11, LWD1 | Transducin/WD40 repeat-like superfamily protein | chr1:4395114-4396154 REVERSE LENGTH=346	AT1G12910.1	TAIR10_20101214.fasta	39,089.30	100.00%	3	3	6	0.03%	23.40%	FPALPVVELQR	99.70%	33.4	25	58.2	1,337.64
ELF4-HFC Col rep 4	| Symbols: ATAN11, LWD1 | Transducin/WD40 repeat-like superfamily protein | chr1:4395114-4396154 REVERSE LENGTH=346	AT1G12910.1	TAIR10_20101214.fasta	39,089.30	100.00%	3	3	6	0.03%	23.40%	IGTSSTDTTCTIWDIER	99.70%	103	29.9	31.5	1,581.74
ELF4-HFC Col rep 4	| Symbols: ATAN11, LWD1 | Transducin/WD40 repeat-like superfamily protein | chr1:4395114-4396154 REVERSE LENGTH=346	AT1G12910.1	TAIR10_20101214.fasta	39,089.30	100.00%	3	3	6	0.03%	23.40%	NSEFCGPLTSFDWNEAEPR	99.70%	65.6	26.6	46.9	1,160.64
ELF4-HFC Col rep 4	| Symbols: ATAN11, LWD1 | Transducin/WD40 repeat-like superfamily protein | chr1:4395114-4396154 REVERSE LENGTH=346	AT1G12910.1	TAIR10_20101214.fasta	39,089.30	100.00%	3	3	6	0.03%	23.40%	VEIVQLDESNGEIR	99.70%	53.6	29.8	66	1,346.73
ELF4-HFC Col rep 4	| Symbols: COP1, ATCOP1, DET340, FUS1, EMB168 | Transducin/WD40 repeat-like superfamily protein | chr2:13978000-13983282 FORWARD LENGTH=675	AT2G32950.1	TAIR10_20101214.fasta	76,188.00	100.00%	8	9	15	0.08%	17.90%	EGYSNGLADFQSVLTTFTR	99.70%	87.4	30.8	50.2	2,269.06
ELF4-HFC Col rep 4	| Symbols: COP1, ATCOP1, DET340, FUS1, EMB168 | Transducin/WD40 repeat-like superfamily protein | chr2:13978000-13983282 FORWARD LENGTH=675	AT2G32950.1	TAIR10_20101214.fasta	76,188.00	100.00%	8	9	15	0.08%	17.90%	EVDNLLTLLAER	99.70%	70.7	28.1	25.2	1,194.48
ELF4-HFC Col rep 4	| Symbols: COP1, ATCOP1, DET340, FUS1, EMB168 | Transducin/WD40 repeat-like superfamily protein | chr2:13978000-13983282 FORWARD LENGTH=675	AT2G32950.1	TAIR10_20101214.fasta	76,188.00	100.00%	8	9	15	0.08%	17.90%	FLSNNELASASTDSTLR	99.70%	96.5	30.5	32.6	2,130.02
ELF4-HFC Col rep 4	| Symbols: COP1, ATCOP1, DET340, FUS1, EMB168 | Transducin/WD40 repeat-like superfamily protein | chr2:13978000-13983282 FORWARD LENGTH=675	AT2G32950.1	TAIR10_20101214.fasta	76,188.00	100.00%	8	9	15	0.08%	17.90%	GGNFVGNYQNK	99.70%	36.5	28.8	55.7	1,319.72
ELF4-HFC Col rep 4	| Symbols: COP1, ATCOP1, DET340, FUS1, EMB168 | Transducin/WD40 repeat-like superfamily protein | chr2:13978000-13983282 FORWARD LENGTH=675	AT2G32950.1	TAIR10_20101214.fasta	76,188.00	100.00%	8	9	15	0.08%	17.90%	IHAQFNDLQECYLQK	99.70%	62.4	30.6	5	989.5304
ELF4-HFC Col rep 4	| Symbols: COP1, ATCOP1, DET340, FUS1, EMB168 | Transducin/WD40 repeat-like superfamily protein | chr2:13978000-13983282 FORWARD LENGTH=675	AT2G32950.1	TAIR10_20101214.fasta	76,188.00	100.00%	8	9	15	0.08%	17.90%	SDCPCCSQHLTNNQLYPNFLLDK	95.50%	21.8	28.8	67.3	1,645.88
ELF4-HFC Col rep 4	| Symbols: COP1, ATCOP1, DET340, FUS1, EMB168 | Transducin/WD40 repeat-like superfamily protein | chr2:13978000-13983282 FORWARD LENGTH=675	AT2G32950.1	TAIR10_20101214.fasta	76,188.00	100.00%	8	9	15	0.08%	17.90%	TASPLDQFR	99.70%	45.2	30.4	48.2	1,485.73
ELF4-HFC Col rep 4	| Symbols: COP1, ATCOP1, DET340, FUS1, EMB168 | Transducin/WD40 repeat-like superfamily protein | chr2:13978000-13983282 FORWARD LENGTH=675	AT2G32950.1	TAIR10_20101214.fasta	76,188.00	100.00%	8	9	15	0.08%	17.90%	VDELNEVQTDLQYIK	99.70%	64.1	31.2	61.2	1,579.75
ELF4-HFC Col rep 4	| Symbols: ELF3, PYK20 | hydroxyproline-rich glycoprotein family protein | chr2:11059459-11063178 FORWARD LENGTH=695	AT2G25930.1	TAIR10_20101214.fasta	77,204.80	100.00%	17	23	30	0.15%	30.50%	EEEDFAVPVYINSR	99.70%	53.3	29.2	23.1	1,748.91
ELF4-HFC Col rep 4	| Symbols: ELF3, PYK20 | hydroxyproline-rich glycoprotein family protein | chr2:11059459-11063178 FORWARD LENGTH=695	AT2G25930.1	TAIR10_20101214.fasta	77,204.80	100.00%	17	23	30	0.15%	30.50%	FGDHGTMNSR	99.30%	31.8	25	23.7	1,552.92
ELF4-HFC Col rep 4	| Symbols: ELF3, PYK20 | hydroxyproline-rich glycoprotein family protein | chr2:11059459-11063178 FORWARD LENGTH=695	AT2G25930.1	TAIR10_20101214.fasta	77,204.80	100.00%	17	23	30	0.15%	30.50%	FQEVNQTGSK	99.70%	65.7	29.3	30.1	2,710.21
ELF4-HFC Col rep 4	| Symbols: ELF3, PYK20 | hydroxyproline-rich glycoprotein family protein | chr2:11059459-11063178 FORWARD LENGTH=695	AT2G25930.1	TAIR10_20101214.fasta	77,204.80	100.00%	17	23	30	0.15%	30.50%	IFQSIQEER	99.70%	64.8	29.9	39.7	1,371.72
ELF4-HFC Col rep 4	| Symbols: ELF3, PYK20 | hydroxyproline-rich glycoprotein family protein | chr2:11059459-11063178 FORWARD LENGTH=695	AT2G25930.1	TAIR10_20101214.fasta	77,204.80	100.00%	17	23	30	0.15%	30.50%	KAIANQQR	99.00%	41.5	25.2	67.1	1,566.86
ELF4-HFC Col rep 4	| Symbols: ELF3, PYK20 | hydroxyproline-rich glycoprotein family protein | chr2:11059459-11063178 FORWARD LENGTH=695	AT2G25930.1	TAIR10_20101214.fasta	77,204.80	100.00%	17	23	30	0.15%	30.50%	LASENAAR	99.00%	57.8	28.7	32	1,820.86
ELF4-HFC Col rep 4	| Symbols: ELF3, PYK20 | hydroxyproline-rich glycoprotein family protein | chr2:11059459-11063178 FORWARD LENGTH=695	AT2G25930.1	TAIR10_20101214.fasta	77,204.80	100.00%	17	23	30	0.15%	30.50%	LHVNDADK	99.00%	44.5	26.8	54.7	2,723.47
ELF4-HFC Col rep 4	| Symbols: ELF3, PYK20 | hydroxyproline-rich glycoprotein family protein | chr2:11059459-11063178 FORWARD LENGTH=695	AT2G25930.1	TAIR10_20101214.fasta	77,204.80	100.00%	17	23	30	0.15%	30.50%	LIAASPDLLLDEISFLGK	99.70%	104	25.4	4.41	2,540.19
ELF4-HFC Col rep 4	| Symbols: ELF3, PYK20 | hydroxyproline-rich glycoprotein family protein | chr2:11059459-11063178 FORWARD LENGTH=695	AT2G25930.1	TAIR10_20101214.fasta	77,204.80	100.00%	17	23	30	0.15%	30.50%	MALYEQLSIPSQR	99.70%	40.4	30.6	43.6	2,316.14
ELF4-HFC Col rep 4	| Symbols: ELF3, PYK20 | hydroxyproline-rich glycoprotein family protein | chr2:11059459-11063178 FORWARD LENGTH=695	AT2G25930.1	TAIR10_20101214.fasta	77,204.80	100.00%	17	23	30	0.15%	30.50%	NLSVQHLDSSAANQATEK	99.70%	121	31	33.4	1,268.74
ELF4-HFC Col rep 4	| Symbols: ELF3, PYK20 | hydroxyproline-rich glycoprotein family protein | chr2:11059459-11063178 FORWARD LENGTH=695	AT2G25930.1	TAIR10_20101214.fasta	77,204.80	100.00%	17	23	30	0.15%	30.50%	QGSTGSSPSGPQGISGSK	99.70%	92.3	29.1	95.1	1,955.90
ELF4-HFC Col rep 4	| Symbols: ELF3, PYK20 | hydroxyproline-rich glycoprotein family protein | chr2:11059459-11063178 FORWARD LENGTH=695	AT2G25930.1	TAIR10_20101214.fasta	77,204.80	100.00%	17	23	30	0.15%	30.50%	QNVCLATCSKPEVR	99.70%	57.1	30.2	62.6	2,255.97
ELF4-HFC Col rep 4	| Symbols: ELF3, PYK20 | hydroxyproline-rich glycoprotein family protein | chr2:11059459-11063178 FORWARD LENGTH=695	AT2G25930.1	TAIR10_20101214.fasta	77,204.80	100.00%	17	23	30	0.15%	30.50%	SDNEPAPQQQQQPTK	99.70%	70.9	29.6	45	1,600.82
ELF4-HFC Col rep 4	| Symbols: ELF3, PYK20 | hydroxyproline-rich glycoprotein family protein | chr2:11059459-11063178 FORWARD LENGTH=695	AT2G25930.1	TAIR10_20101214.fasta	77,204.80	100.00%	17	23	30	0.15%	30.50%	SNNTSTLVHPGPSSQPCGVER	99.70%	46.9	30.5	43.5	2,107.00
ELF4-HFC Col rep 4	| Symbols: ELF3, PYK20 | hydroxyproline-rich glycoprotein family protein | chr2:11059459-11063178 FORWARD LENGTH=695	AT2G25930.1	TAIR10_20101214.fasta	77,204.80	100.00%	17	23	30	0.15%	30.50%	TTVTQTTR	94.50%	27.2	28.3	58.4	1,385.76
ELF4-HFC Col rep 4	| Symbols: ELF3, PYK20 | hydroxyproline-rich glycoprotein family protein | chr2:11059459-11063178 FORWARD LENGTH=695	AT2G25930.1	TAIR10_20101214.fasta	77,204.80	100.00%	17	23	30	0.15%	30.50%	VFAVQLFELHR	99.70%	67.3	28.4	82.7	1,825.89
ELF4-HFC Col rep 4	| Symbols: ELF3, PYK20 | hydroxyproline-rich glycoprotein family protein | chr2:11059459-11063178 FORWARD LENGTH=695	AT2G25930.1	TAIR10_20101214.fasta	77,204.80	100.00%	17	23	30	0.15%	30.50%	VNDYNASLR	99.70%	71.8	28.9	25.2	1,197.56
ELF4-HFC Col rep 4	| Symbols: ELF4 | Protein of unknown function (DUF1313) | chr2:16734545-16734880 REVERSE LENGTH=111	AT2G40080.1	TAIR10_20101214.fasta	12,375.40	100.00%	7	26	158	0.80%	79.30%	NGHDGGGAAGTR	99.70%	50.3	25.6	53.7	1,906.91
ELF4-HFC Col rep 4	| Symbols: ELF4 | Protein of unknown function (DUF1313) | chr2:16734545-16734880 REVERSE LENGTH=111	AT2G40080.1	TAIR10_20101214.fasta	12,375.40	100.00%	7	26	158	0.80%	79.30%	NVAEEAEQGEDPAMWENLDR	99.70%	104	25	21.8	2,824.25
ELF4-HFC Col rep 4	| Symbols: ELF4 | Protein of unknown function (DUF1313) | chr2:16734545-16734880 REVERSE LENGTH=111	AT2G40080.1	TAIR10_20101214.fasta	12,375.40	100.00%	7	26	158	0.80%	79.30%	NVALIQELNGNISK	99.70%	132	28.4	25.5	1,034.53
ELF4-HFC Col rep 4	| Symbols: ELF4 | Protein of unknown function (DUF1313) | chr2:16734545-16734880 REVERSE LENGTH=111	AT2G40080.1	TAIR10_20101214.fasta	12,375.40	100.00%	7	26	158	0.80%	79.30%	QVQSVLDR	99.00%	55.2	29.3	52	1,806.91
ELF4-HFC Col rep 4	| Symbols: ELF4 | Protein of unknown function (DUF1313) | chr2:16734545-16734880 REVERSE LENGTH=111	AT2G40080.1	TAIR10_20101214.fasta	12,375.40	100.00%	7	26	158	0.80%	79.30%	RNVAEEAEQGEDPAMWENLDR	99.70%	73.3	28.1	42.9	1,668.78
ELF4-HFC Col rep 4	| Symbols: ELF4 | Protein of unknown function (DUF1313) | chr2:16734545-16734880 REVERSE LENGTH=111	AT2G40080.1	TAIR10_20101214.fasta	12,375.40	100.00%	7	26	158	0.80%	79.30%	SLIQQVNDNHQSR	99.70%	73.9	30	19.3	1,137.47
ELF4-HFC Col rep 4	| Symbols: ELF4 | Protein of unknown function (DUF1313) | chr2:16734545-16734880 REVERSE LENGTH=111	AT2G40080.1	TAIR10_20101214.fasta	12,375.40	100.00%	7	26	158	0.80%	79.30%	VVNMYSDLNTSFSSGFHGGK	99.70%	102	29.7	46.1	1,137.55
ELF4-HFC Col rep 4	| Symbols: PCL1, LUX | Homeodomain-like superfamily protein | chr3:17183248-17184219 FORWARD LENGTH=323	AT3G46640.1,AT3G46640.2	TAIR10_20101214.fasta	34,648.50	100.00%	2	3	5	0.03%	15.20%	ENVASHLQK	99.70%	44.6	28.1	40.6	1,149.59
ELF4-HFC Col rep 4	| Symbols: PCL1, LUX | Homeodomain-like superfamily protein | chr3:17183248-17184219 FORWARD LENGTH=323	AT3G46640.1,AT3G46640.2	TAIR10_20101214.fasta	34,648.50	100.00%	2	3	5	0.03%	15.20%	FVDVVAHLGIK	99.70%	45.8	25	27.9	928.5323
ELF4-HFC Col rep 4	| Symbols: PCL1, LUX | Homeodomain-like superfamily protein | chr3:17183248-17184219 FORWARD LENGTH=323	AT3G46640.1,AT3G46640.2	TAIR10_20101214.fasta	34,648.50	100.00%	2	3	5	0.03%	15.20%	GGSSGPNTSSSNNNVEEEDR	99.70%	92.1	25	42.8	831.4318
ELF4-HFC Col rep 4	| Symbols: PCL1, LUX | Homeodomain-like superfamily protein | chr3:17183248-17184219 FORWARD LENGTH=323	AT3G46640.1,AT3G46640.2	TAIR10_20101214.fasta	34,648.50	100.00%	2	3	5	0.03%	15.20%	LVWTPQLHK	99.70%	35.2	25.5	32.7	911.4582
ELF4-HFC Col rep 4	| Symbols: PHYA, FHY2, FRE1, HY8 | phytochrome A | chr1:3095498-3099216 REVERSE LENGTH=1122	AT1G09570.1	TAIR10_20101214.fasta	124,503.20	100.00%	7	7	9	0.05%	9.27%	ADAGPISLVVNACASR	99.70%	67	29.5	95.5	1,915.08
ELF4-HFC Col rep 4	| Symbols: PHYA, FHY2, FRE1, HY8 | phytochrome A | chr1:3095498-3099216 REVERSE LENGTH=1122	AT1G09570.1	TAIR10_20101214.fasta	124,503.20	100.00%	7	7	9	0.05%	9.27%	ALGFGDVSLLNPILVHCR	99.70%	44.7	28.1	26.7	1,551.78
ELF4-HFC Col rep 4	| Symbols: PHYA, FHY2, FRE1, HY8 | phytochrome A | chr1:3095498-3099216 REVERSE LENGTH=1122	AT1G09570.1	TAIR10_20101214.fasta	124,503.20	100.00%	7	7	9	0.05%	9.27%	LIETATVPILAVDSDGLVNGWNTK	99.70%	42.7	28.9	113	1,912.94
ELF4-HFC Col rep 4	| Symbols: PHYA, FHY2, FRE1, HY8 | phytochrome A | chr1:3095498-3099216 REVERSE LENGTH=1122	AT1G09570.1	TAIR10_20101214.fasta	124,503.20	100.00%	7	7	9	0.05%	9.27%	LIQPFGCLLALDEK	99.70%	42.5	29.9	80	1,618.77
ELF4-HFC Col rep 4	| Symbols: PHYA, FHY2, FRE1, HY8 | phytochrome A | chr1:3095498-3099216 REVERSE LENGTH=1122	AT1G09570.1	TAIR10_20101214.fasta	124,503.20	100.00%	7	7	9	0.05%	9.27%	SSFIITAELAAANK	99.70%	83.1	28.7	41.7	1,661.81
ELF4-HFC Col rep 4	| Symbols: PHYA, FHY2, FRE1, HY8 | phytochrome A | chr1:3095498-3099216 REVERSE LENGTH=1122	AT1G09570.1	TAIR10_20101214.fasta	124,503.20	100.00%	7	7	9	0.05%	9.27%	VSFAFFTR	99.00%	58.2	29.6	53.2	1,695.79
ELF4-HFC Col rep 4	| Symbols: PHYA, FHY2, FRE1, HY8 | phytochrome A | chr1:3095498-3099216 REVERSE LENGTH=1122	AT1G09570.1	TAIR10_20101214.fasta	124,503.20	100.00%	7	7	9	0.05%	9.27%	YVECLLCVSK	99.70%	46.3	28	38.8	2,224.04
ELF4-HFC Col rep 4	| Symbols: PHYB, HY3, OOP1 | phytochrome B | chr2:8140079-8144151 FORWARD LENGTH=1172	AT2G18790.1	TAIR10_20101214.fasta	129,333.90	100.00%	25	41	65	0.33%	40.70%	AIQQYTVDAR	99.70%	69.9	30	18.8	907.4844
ELF4-HFC Col rep 4	| Symbols: PHYB, HY3, OOP1 | phytochrome B | chr2:8140079-8144151 FORWARD LENGTH=1172	AT2G18790.1	TAIR10_20101214.fasta	129,333.90	100.00%	25	41	65	0.33%	40.70%	AISQLQALPGGDIK	99.70%	109	26.1	47.8	1,358.76
ELF4-HFC Col rep 4	| Symbols: PHYB, HY3, OOP1 | phytochrome B | chr2:8140079-8144151 FORWARD LENGTH=1172	AT2G18790.1	TAIR10_20101214.fasta	129,333.90	100.00%	25	41	65	0.33%	40.70%	AVFVVVNACSSK	99.70%	74.1	29.6	57.8	1,051.52
ELF4-HFC Col rep 4	| Symbols: PHYB, HY3, OOP1 | phytochrome B | chr2:8140079-8144151 FORWARD LENGTH=1172	AT2G18790.1	TAIR10_20101214.fasta	129,333.90	100.00%	25	41	65	0.33%	40.70%	CDGAAFLYHGK	99.70%	38.3	27.5	43.6	1,069.48
ELF4-HFC Col rep 4	| Symbols: PHYB, HY3, OOP1 | phytochrome B | chr2:8140079-8144151 FORWARD LENGTH=1172	AT2G18790.1	TAIR10_20101214.fasta	129,333.90	100.00%	25	41	65	0.33%	40.70%	DYLNNIVGVCFVGQDVTSQK	99.70%	98.2	31.4	104	2,318.98
ELF4-HFC Col rep 4	| Symbols: PHYB, HY3, OOP1 | phytochrome B | chr2:8140079-8144151 FORWARD LENGTH=1172	AT2G18790.1	TAIR10_20101214.fasta	129,333.90	100.00%	25	41	65	0.33%	40.70%	EITLLNPVWIHSK	99.70%	59.5	26.8	34.3	1,513.82
ELF4-HFC Col rep 4	| Symbols: PHYB, HY3, OOP1 | phytochrome B | chr2:8140079-8144151 FORWARD LENGTH=1172	AT2G18790.1	TAIR10_20101214.fasta	129,333.90	100.00%	25	41	65	0.33%	40.70%	ELAYICQVIK	99.70%	52.3	28.6	15.1	944.516
ELF4-HFC Col rep 4	| Symbols: PHYB, HY3, OOP1 | phytochrome B | chr2:8140079-8144151 FORWARD LENGTH=1172	AT2G18790.1	TAIR10_20101214.fasta	129,333.90	100.00%	25	41	65	0.33%	40.70%	FANSLLEATDLNEDQK	99.70%	100	31	63.3	2,475.08
ELF4-HFC Col rep 4	| Symbols: PHYB, HY3, OOP1 | phytochrome B | chr2:8140079-8144151 FORWARD LENGTH=1172	AT2G18790.1	TAIR10_20101214.fasta	129,333.90	100.00%	25	41	65	0.33%	40.70%	FHEDEHGEVVAESK	99.70%	90.5	27.1	1.5	1,539.75
ELF4-HFC Col rep 4	| Symbols: PHYB, HY3, OOP1 | phytochrome B | chr2:8140079-8144151 FORWARD LENGTH=1172	AT2G18790.1	TAIR10_20101214.fasta	129,333.90	100.00%	25	41	65	0.33%	40.70%	FINIQGDYK	99.70%	49.3	28	102	2,146.99
ELF4-HFC Col rep 4	| Symbols: PHYB, HY3, OOP1 | phytochrome B | chr2:8140079-8144151 FORWARD LENGTH=1172	AT2G18790.1	TAIR10_20101214.fasta	129,333.90	100.00%	25	41	65	0.33%	40.70%	GGEEEPSSSHTPNNR	99.70%	57.6	25.3	23.3	1,025.54
ELF4-HFC Col rep 4	| Symbols: PHYB, HY3, OOP1 | phytochrome B | chr2:8140079-8144151 FORWARD LENGTH=1172	AT2G18790.1	TAIR10_20101214.fasta	129,333.90	100.00%	25	41	65	0.33%	40.70%	HHPEDKDDGQR	99.70%	45.1	25	41.5	1,197.70
ELF4-HFC Col rep 4	| Symbols: PHYB, HY3, OOP1 | phytochrome B | chr2:8140079-8144151 FORWARD LENGTH=1172	AT2G18790.1	TAIR10_20101214.fasta	129,333.90	100.00%	25	41	65	0.33%	40.70%	IAELTGLSVEEAMGK	99.70%	50.2	30.9	85.2	2,036.84
ELF4-HFC Col rep 4	| Symbols: PHYB, HY3, OOP1 | phytochrome B | chr2:8140079-8144151 FORWARD LENGTH=1172	AT2G18790.1	TAIR10_20101214.fasta	129,333.90	100.00%	25	41	65	0.33%	40.70%	IVGDMDLESIEDGSFVLK	99.70%	107	31.2	26.7	1,121.65
ELF4-HFC Col rep 4	| Symbols: PHYB, HY3, OOP1 | phytochrome B | chr2:8140079-8144151 FORWARD LENGTH=1172	AT2G18790.1	TAIR10_20101214.fasta	129,333.90	100.00%	25	41	65	0.33%	40.70%	LHAVFEQSGESGK	99.70%	72.8	29.2	57.6	1,600.81
ELF4-HFC Col rep 4	| Symbols: PHYB, HY3, OOP1 | phytochrome B | chr2:8140079-8144151 FORWARD LENGTH=1172	AT2G18790.1	TAIR10_20101214.fasta	129,333.90	100.00%	25	41	65	0.33%	40.70%	LIETATVPIFAVDAGGCINGWNAK	99.70%	90.7	31.4	38.6	1,981.07
ELF4-HFC Col rep 4	| Symbols: PHYB, HY3, OOP1 | phytochrome B | chr2:8140079-8144151 FORWARD LENGTH=1172	AT2G18790.1	TAIR10_20101214.fasta	129,333.90	100.00%	25	41	65	0.33%	40.70%	LLCDTVVESVR	99.70%	76.1	30.4	42.4	2,526.35
ELF4-HFC Col rep 4	| Symbols: PHYB, HY3, OOP1 | phytochrome B | chr2:8140079-8144151 FORWARD LENGTH=1172	AT2G18790.1	TAIR10_20101214.fasta	129,333.90	100.00%	25	41	65	0.33%	40.70%	MIVDCNATPVLVVQDDR	99.70%	93.3	30.7	34.6	1,616.87
ELF4-HFC Col rep 4	| Symbols: PHYB, HY3, OOP1 | phytochrome B | chr2:8140079-8144151 FORWARD LENGTH=1172	AT2G18790.1	TAIR10_20101214.fasta	129,333.90	100.00%	25	41	65	0.33%	40.70%	NTGKPFYAILHR	99.70%	61.1	29.3	73.8	1,435.78
ELF4-HFC Col rep 4	| Symbols: PHYB, HY3, OOP1 | phytochrome B | chr2:8140079-8144151 FORWARD LENGTH=1172	AT2G18790.1	TAIR10_20101214.fasta	129,333.90	100.00%	25	41	65	0.33%	40.70%	QLLETSVSCEK	99.70%	48.2	30.2	45.6	974.5093
ELF4-HFC Col rep 4	| Symbols: PHYB, HY3, OOP1 | phytochrome B | chr2:8140079-8144151 FORWARD LENGTH=1172	AT2G18790.1	TAIR10_20101214.fasta	129,333.90	100.00%	25	41	65	0.33%	40.70%	RDDLEPYIGLHYPATDIPQASR	99.70%	48.5	31.4	34	1,270.62
ELF4-HFC Col rep 4	| Symbols: PHYB, HY3, OOP1 | phytochrome B | chr2:8140079-8144151 FORWARD LENGTH=1172	AT2G18790.1	TAIR10_20101214.fasta	129,333.90	100.00%	25	41	65	0.33%	40.70%	RQDTECFTK	96.60%	26.4	26.1	61.6	1,164.60
ELF4-HFC Col rep 4	| Symbols: PHYB, HY3, OOP1 | phytochrome B | chr2:8140079-8144151 FORWARD LENGTH=1172	AT2G18790.1	TAIR10_20101214.fasta	129,333.90	100.00%	25	41	65	0.33%	40.70%	SFDYSQSLK	99.70%	45.9	26.4	97.2	1,410.80
ELF4-HFC Col rep 4	| Symbols: PHYB, HY3, OOP1 | phytochrome B | chr2:8140079-8144151 FORWARD LENGTH=1172	AT2G18790.1	TAIR10_20101214.fasta	129,333.90	100.00%	25	41	65	0.33%	40.70%	SIEVFGDQIR	99.70%	55.2	30.5	63.2	1,280.67
ELF4-HFC Col rep 4	| Symbols: PHYB, HY3, OOP1 | phytochrome B | chr2:8140079-8144151 FORWARD LENGTH=1172	AT2G18790.1	TAIR10_20101214.fasta	129,333.90	100.00%	25	41	65	0.33%	40.70%	SLFTSSSSILLER	99.70%	75.4	28.2	26.6	1,238.56
ELF4-HFC Col rep 4	| Symbols: PHYB, HY3, OOP1 | phytochrome B | chr2:8140079-8144151 FORWARD LENGTH=1172	AT2G18790.1	TAIR10_20101214.fasta	129,333.90	100.00%	25	41	65	0.33%	40.70%	SLVSDLIYK	99.70%	56.9	26.1	98	2,256.10
ELF4-HFC Col rep 4	| Symbols: PHYB, HY3, OOP1 | phytochrome B | chr2:8140079-8144151 FORWARD LENGTH=1172	AT2G18790.1	TAIR10_20101214.fasta	129,333.90	100.00%	25	41	65	0.33%	40.70%	SLVSDLIYKENEATVNK	99.70%	41.7	30.2	40.2	1,549.87
ELF4-HFC Col rep 4	| Symbols: PHYB, HY3, OOP1 | phytochrome B | chr2:8140079-8144151 FORWARD LENGTH=1172	AT2G18790.1	TAIR10_20101214.fasta	129,333.90	100.00%	25	41	65	0.33%	40.70%	SSFQAFLEVVK	99.70%	61.6	27.1	39.4	1,236.67
ELF4-HFC Col rep 4	| Symbols: PHYB, HY3, OOP1 | phytochrome B | chr2:8140079-8144151 FORWARD LENGTH=1172	AT2G18790.1	TAIR10_20101214.fasta	129,333.90	100.00%	25	41	65	0.33%	40.70%	TEDPALSIAGAVQSQK	99.70%	92.9	29.4	77	1,807.87
ELF4-HFC Col rep 4	| Symbols: PHYB, HY3, OOP1 | phytochrome B | chr2:8140079-8144151 FORWARD LENGTH=1172	AT2G18790.1	TAIR10_20101214.fasta	129,333.90	100.00%	25	41	65	0.33%	40.70%	TTTYGSSVPEQQITAYLSR	99.70%	59.4	30.9	83.8	1,612.72
ELF4-HFC Col rep 4	| Symbols: PHYB, HY3, OOP1 | phytochrome B | chr2:8140079-8144151 FORWARD LENGTH=1172	AT2G18790.1	TAIR10_20101214.fasta	129,333.90	100.00%	25	41	65	0.33%	40.70%	VIGAFCFLQIPSPELQQALAVQR	99.70%	84.1	28.4	33.8	1,097.56

ELF4-HFC Col rep 4	| Symbols: PHYB, HY3, OOP1 | phytochrome B | chr2:8140079-8144151 FORWARD LENGTH=1172	AT2G18790.1	TAIR10_20101214.fasta	129,333.90	100.00%	25	41	65	0.33%	40.70%	VSGVGGSGGGR	99.70%	45	28.4	45.1	1,597.68
ELF4-HFC Col rep 4	| Symbols: PHYB, HY3, OOP1 | phytochrome B | chr2:8140079-8144151 FORWARD LENGTH=1172	AT2G18790.1	TAIR10_20101214.fasta	129,333.90	100.00%	25	41	65	0.33%	40.70%	VVDGVVQPCR	99.70%	47.6	27.4	35.8	1,333.59
ELF4-HFC Col rep 4	| Symbols: PHYB, HY3, OOP1 | phytochrome B | chr2:8140079-8144151 FORWARD LENGTH=1172	AT2G18790.1	TAIR10_20101214.fasta	129,333.90	100.00%	25	41	65	0.33%	40.70%	YAPSQEWVEIHLSQLSK	99.70%	68.2	30.6	46.4	1,563.79
ELF4-HFC Col rep 4	| Symbols: PHYB, HY3, OOP1 | phytochrome B | chr2:8140079-8144151 FORWARD LENGTH=1172	AT2G18790.1	TAIR10_20101214.fasta	129,333.90	100.00%	25	41	65	0.33%	40.70%	YYPLGVAPSEVQIK	99.70%	67.2	29.5	97.2	1,982.96
ELF4-HFC Col rep 4	| Symbols: PHYC | phytochrome C | chr5:14008049-14011619 FORWARD LENGTH=1111	AT5G35840.1	TAIR10_20101214.fasta	123,724.60	100.00%	30	45	54	0.27%	36.20%	AAEVTGLAVEQAIGKPVSDLVEDDSVETVK	99.70%	98.9	30.4	62.3	1,388.68
ELF4-HFC Col rep 4	| Symbols: PHYC | phytochrome C | chr5:14008049-14011619 FORWARD LENGTH=1111	AT5G35840.1	TAIR10_20101214.fasta	123,724.60	100.00%	30	45	54	0.27%	36.20%	AISFLQDLLHSSGLSEDQK	99.70%	93.7	30.9	23.7	2,518.26
ELF4-HFC Col rep 4	| Symbols: PHYC | phytochrome C | chr5:14008049-14011619 FORWARD LENGTH=1111	AT5G35840.1	TAIR10_20101214.fasta	123,724.60	100.00%	30	45	54	0.27%	36.20%	AISFLQDLLHSSGLSEDQKR	99.70%	54.6	30.4	52.5	1,290.67
ELF4-HFC Col rep 4	| Symbols: PHYC | phytochrome C | chr5:14008049-14011619 FORWARD LENGTH=1111	AT5G35840.1	TAIR10_20101214.fasta	123,724.60	100.00%	30	45	54	0.27%	36.20%	AVDFGEISILNPITLHCR	99.70%	60.4	30	89.8	1,960.95
ELF4-HFC Col rep 4	| Symbols: PHYC | phytochrome C | chr5:14008049-14011619 FORWARD LENGTH=1111	AT5G35840.1	TAIR10_20101214.fasta	123,724.60	100.00%	30	45	54	0.27%	36.20%	CDGAALYYR	99.70%	47.8	25	55.5	1,416.77
ELF4-HFC Col rep 4	| Symbols: PHYC | phytochrome C | chr5:14008049-14011619 FORWARD LENGTH=1111	AT5G35840.1	TAIR10_20101214.fasta	123,724.60	100.00%	30	45	54	0.27%	36.20%	DGSFIEALLSANK	99.70%	100	30	42.5	1,293.64
ELF4-HFC Col rep 4	| Symbols: PHYC | phytochrome C | chr5:14008049-14011619 FORWARD LENGTH=1111	AT5G35840.1	TAIR10_20101214.fasta	123,724.60	100.00%	30	45	54	0.27%	36.20%	DGSFIEALLSANKR	99.70%	58.9	29.6	0	2,527.26
ELF4-HFC Col rep 4	| Symbols: PHYC | phytochrome C | chr5:14008049-14011619 FORWARD LENGTH=1111	AT5G35840.1	TAIR10_20101214.fasta	123,724.60	100.00%	30	45	54	0.27%	36.20%	DLIDWVLK	99.00%	47.1	29	10.8	1,184.54
ELF4-HFC Col rep 4	| Symbols: PHYC | phytochrome C | chr5:14008049-14011619 FORWARD LENGTH=1111	AT5G35840.1	TAIR10_20101214.fasta	123,724.60	100.00%	30	45	54	0.27%	36.20%	DMTNNVLGVCFIGQDVTGQK	99.70%	68	30.5	34.9	1,074.51
ELF4-HFC Col rep 4	| Symbols: PHYC | phytochrome C | chr5:14008049-14011619 FORWARD LENGTH=1111	AT5G35840.1	TAIR10_20101214.fasta	123,724.60	100.00%	30	45	54	0.27%	36.20%	DNLWSLGVTPTETQIR	99.70%	48.5	30.5	42.9	1,163.61
ELF4-HFC Col rep 4	| Symbols: PHYC | phytochrome C | chr5:14008049-14011619 FORWARD LENGTH=1111	AT5G35840.1	TAIR10_20101214.fasta	123,724.60	100.00%	30	45	54	0.27%	36.20%	EAESAVLLK	99.70%	61.6	30.5	65.2	1,439.77
ELF4-HFC Col rep 4	| Symbols: PHYC | phytochrome C | chr5:14008049-14011619 FORWARD LENGTH=1111	AT5G35840.1	TAIR10_20101214.fasta	123,724.60	100.00%	30	45	54	0.27%	36.20%	EALTIGTDVK	99.60%	37.5	30	43	1,037.59
ELF4-HFC Col rep 4	| Symbols: PHYC | phytochrome C | chr5:14008049-14011619 FORWARD LENGTH=1111	AT5G35840.1	TAIR10_20101214.fasta	123,724.60	100.00%	30	45	54	0.27%	36.20%	EGLGLHITQK	99.70%	38.7	25.9	29.3	1,923.01
ELF4-HFC Col rep 4	| Symbols: PHYC | phytochrome C | chr5:14008049-14011619 FORWARD LENGTH=1111	AT5G35840.1	TAIR10_20101214.fasta	123,724.60	100.00%	30	45	54	0.27%	36.20%	EVSELTGYDR	99.70%	39	27.5	46.8	1,254.67
ELF4-HFC Col rep 4	| Symbols: PHYC | phytochrome C | chr5:14008049-14011619 FORWARD LENGTH=1111	AT5G35840.1	TAIR10_20101214.fasta	123,724.60	100.00%	30	45	54	0.27%	36.20%	GSLQEEHSK	99.70%	47.2	25.6	78.9	1,614.83
ELF4-HFC Col rep 4	| Symbols: PHYC | phytochrome C | chr5:14008049-14011619 FORWARD LENGTH=1111	AT5G35840.1	TAIR10_20101214.fasta	123,724.60	100.00%	30	45	54	0.27%	36.20%	IEEGLVIDLEPVSPDEVPVTAAGALR	99.70%	86.6	28.7	48.5	2,102.04
ELF4-HFC Col rep 4	| Symbols: PHYC | phytochrome C | chr5:14008049-14011619 FORWARD LENGTH=1111	AT5G35840.1	TAIR10_20101214.fasta	123,724.60	100.00%	30	45	54	0.27%	36.20%	IGFNAVISGQK	99.70%	66.4	28.6	81	2,585.39
ELF4-HFC Col rep 4	| Symbols: PHYC | phytochrome C | chr5:14008049-14011619 FORWARD LENGTH=1111	AT5G35840.1	TAIR10_20101214.fasta	123,724.60	100.00%	30	45	54	0.27%	36.20%	IIHPAPGLPEDLVR	99.70%	60.6	26.1	24.9	889.4487
ELF4-HFC Col rep 4	| Symbols: PHYC | phytochrome C | chr5:14008049-14011619 FORWARD LENGTH=1111	AT5G35840.1	TAIR10_20101214.fasta	123,724.60	100.00%	30	45	54	0.27%	36.20%	ILLGEVFTTDDYGCCLK	99.70%	104	30.3	40.7	1,128.58
ELF4-HFC Col rep 4	| Symbols: PHYC | phytochrome C | chr5:14008049-14011619 FORWARD LENGTH=1111	AT5G35840.1	TAIR10_20101214.fasta	123,724.60	100.00%	30	45	54	0.27%	36.20%	LHGNFEESER	95.60%	24	25.8	58.5	2,015.02
ELF4-HFC Col rep 4	| Symbols: PHYC | phytochrome C | chr5:14008049-14011619 FORWARD LENGTH=1111	AT5G35840.1	TAIR10_20101214.fasta	123,724.60	100.00%	30	45	54	0.27%	36.20%	LIDTAAVPIFAVDASGVINGWNSK	99.70%	88.3	30.1	58	1,563.84
ELF4-HFC Col rep 4	| Symbols: PHYC | phytochrome C | chr5:14008049-14011619 FORWARD LENGTH=1111	AT5G35840.1	TAIR10_20101214.fasta	123,724.60	100.00%	30	45	54	0.27%	36.20%	LLFGFYHR	99.00%	33.5	27.6	96	3,069.58
ELF4-HFC Col rep 4	| Symbols: PHYC | phytochrome C | chr5:14008049-14011619 FORWARD LENGTH=1111	AT5G35840.1	TAIR10_20101214.fasta	123,724.60	100.00%	30	45	54	0.27%	36.20%	LQQILSETLLSSIR	99.70%	98.3	25.3	82.8	2,088.06
ELF4-HFC Col rep 4	| Symbols: PHYC | phytochrome C | chr5:14008049-14011619 FORWARD LENGTH=1111	AT5G35840.1	TAIR10_20101214.fasta	123,724.60	100.00%	30	45	54	0.27%	36.20%	NAPIGIVTQSPNIMDLVK	99.70%	63.4	28.7	48.5	2,244.16
ELF4-HFC Col rep 4	| Symbols: PHYC | phytochrome C | chr5:14008049-14011619 FORWARD LENGTH=1111	AT5G35840.1	TAIR10_20101214.fasta	123,724.60	100.00%	30	45	54	0.27%	36.20%	SLSQPISLSGSTLR	99.70%	86.6	29.1	50.5	2,055.07
ELF4-HFC Col rep 4	| Symbols: PHYC | phytochrome C | chr5:14008049-14011619 FORWARD LENGTH=1111	AT5G35840.1	TAIR10_20101214.fasta	123,724.60	100.00%	30	45	54	0.27%	36.20%	SSPVELVVNTCCSR	99.70%	52.8	29.1	38.5	1,088.48
ELF4-HFC Col rep 4	| Symbols: PHYC | phytochrome C | chr5:14008049-14011619 FORWARD LENGTH=1111	AT5G35840.1	TAIR10_20101214.fasta	123,724.60	100.00%	30	45	54	0.27%	36.20%	SSSKPFYAILHR	99.70%	53.2	29.5	83.3	1,364.71
ELF4-HFC Col rep 4	| Symbols: PHYC | phytochrome C | chr5:14008049-14011619 FORWARD LENGTH=1111	AT5G35840.1	TAIR10_20101214.fasta	123,724.60	100.00%	30	45	54	0.27%	36.20%	TVVDVPLVDNR	99.70%	52	26.9	43.8	1,520.81
ELF4-HFC Col rep 4	| Symbols: PHYC | phytochrome C | chr5:14008049-14011619 FORWARD LENGTH=1111	AT5G35840.1	TAIR10_20101214.fasta	123,724.60	100.00%	30	45	54	0.27%	36.20%	VSSQVLVDAK	99.70%	65.4	29.1	21.6	1,001.57
ELF4-HFC Col rep 4	| Symbols: PHYC | phytochrome C | chr5:14008049-14011619 FORWARD LENGTH=1111	AT5G35840.1	TAIR10_20101214.fasta	123,724.60	100.00%	30	45	54	0.27%	36.20%	YACEFLTQVFGVQINK	99.70%	77.9	30.5	61.1	2,212.04
ELF4-HFC Col rep 4	| Symbols: PHYD | phytochrome D | chr4:9195602-9199486 REVERSE LENGTH=1164	AT4G16250.1	TAIR10_20101214.fasta	129,270.30	99.40%	0	0	14	0.07%	10.80%	AIQQYTVDAR	99.70%	69.9	30	34.1	1,829.94
ELF4-HFC Col rep 4	| Symbols: PHYD | phytochrome D | chr4:9195602-9199486 REVERSE LENGTH=1164	AT4G16250.1	TAIR10_20101214.fasta	129,270.30	99.40%	0	0	14	0.07%	10.80%	FHEDEHGEVVAESK	99.70%	90.5	27.1	39.8	959.5408
ELF4-HFC Col rep 4	| Symbols: PHYD | phytochrome D | chr4:9195602-9199486 REVERSE LENGTH=1164	AT4G16250.1	TAIR10_20101214.fasta	129,270.30	99.40%	0	0	14	0.07%	10.80%	FINIQGDYK	99.70%	49.3	28	19.6	1,046.57
ELF4-HFC Col rep 4	| Symbols: PHYD | phytochrome D | chr4:9195602-9199486 REVERSE LENGTH=1164	AT4G16250.1	TAIR10_20101214.fasta	129,270.30	99.40%	0	0	14	0.07%	10.80%	HHPEDKDDGQR	99.70%	45.1	25	29	1,095.62
ELF4-HFC Col rep 4	| Symbols: PHYD | phytochrome D | chr4:9195602-9199486 REVERSE LENGTH=1164	AT4G16250.1	TAIR10_20101214.fasta	129,270.30	99.40%	0	0	14	0.07%	10.80%	LHAVFEQSGESGK	99.70%	72.8	29.2	32.2	1,168.55
ELF4-HFC Col rep 4	| Symbols: PHYD | phytochrome D | chr4:9195602-9199486 REVERSE LENGTH=1164	AT4G16250.1	TAIR10_20101214.fasta	129,270.30	99.40%	0	0	14	0.07%	10.80%	LLCDTVVESVR	99.70%	76.1	30.4	32.1	1,014.49
ELF4-HFC Col rep 4	| Symbols: PHYD | phytochrome D | chr4:9195602-9199486 REVERSE LENGTH=1164	AT4G16250.1	TAIR10_20101214.fasta	129,270.30	99.40%	0	0	14	0.07%	10.80%	QLLETSVSCEK	99.70%	48.2	30.2	80.6	2,689.43
ELF4-HFC Col rep 4	| Symbols: PHYD | phytochrome D | chr4:9195602-9199486 REVERSE LENGTH=1164	AT4G16250.1	TAIR10_20101214.fasta	129,270.30	99.40%	0	0	14	0.07%	10.80%	RNDLEPYIGLHYPATDIPQASR	99.70%	48.5	31.4	50.6	1,133.63
ELF4-HFC Col rep 4	| Symbols: PHYD | phytochrome D | chr4:9195602-9199486 REVERSE LENGTH=1164	AT4G16250.1	TAIR10_20101214.fasta	129,270.30	99.40%	0	0	14	0.07%	10.80%	SFDYSQSLK	99.70%	45.9	26.4	53.7	1,526.87
ELF4-HFC Col rep 4	| Symbols: PHYD | phytochrome D | chr4:9195602-9199486 REVERSE LENGTH=1164	AT4G16250.1	TAIR10_20101214.fasta	129,270.30	99.40%	0	0	14	0.07%	10.80%	TEDPALSIAGAVQSQK	99.70%	92.9	29.4	99.2	2,003.95
ELF4-HFC Col rep 4	| Symbols: PHYE | phytochrome E | chr4:10042312-10045948 REVERSE LENGTH=1112	AT4G18130.1	TAIR10_20101214.fasta	122,519.10	100.00%	19	22	29	0.15%	26.70%	AALESLLCK	99.70%	55.3	30.1	11.1	1,217.55
ELF4-HFC Col rep 4	| Symbols: PHYE | phytochrome E | chr4:10042312-10045948 REVERSE LENGTH=1112	AT4G18130.1	TAIR10_20101214.fasta	122,519.10	100.00%	19	22	29	0.15%	26.70%	AASFTEISLLNPVLVHSR	99.70%	56.1	27	85.5	2,458.30
ELF4-HFC Col rep 4	| Symbols: PHYE | phytochrome E | chr4:10042312-10045948 REVERSE LENGTH=1112	AT4G18130.1	TAIR10_20101214.fasta	122,519.10	100.00%	19	22	29	0.15%	26.70%	DGWVTPDGLGLK	99.70%	40.5	27.7	23.8	1,052.57
ELF4-HFC Col rep 4	| Symbols: PHYE | phytochrome E | chr4:10042312-10045948 REVERSE LENGTH=1112	AT4G18130.1	TAIR10_20101214.fasta	122,519.10	100.00%	19	22	29	0.15%	26.70%	DYTENIIGVCFVGQDITSEK	99.70%	71.3	30.9	92.8	1,600.93
ELF4-HFC Col rep 4	| Symbols: PHYE | phytochrome E | chr4:10042312-10045948 REVERSE LENGTH=1112	AT4G18130.1	TAIR10_20101214.fasta	122,519.10	100.00%	19	22	29	0.15%	26.70%	ESAQSLNELTYVR	99.70%	69.6	30.6	63.4	1,926.04
ELF4-HFC Col rep 4	| Symbols: PHYE | phytochrome E | chr4:10042312-10045948 REVERSE LENGTH=1112	AT4G18130.1	TAIR10_20101214.fasta	122,519.10	100.00%	19	22	29	0.15%	26.70%	ESFTSSRPVLSGNGVAR	99.70%	45.2	31.4	76.5	1,445.80
ELF4-HFC Col rep 4	| Symbols: PHYE | phytochrome E | chr4:10042312-10045948 REVERSE LENGTH=1112	AT4G18130.1	TAIR10_20101214.fasta	122,519.10	100.00%	19	22	29	0.15%	26.70%	FGQNNHPDYSSDVCVLVNSCTSR	99.70%	36.6	27.6	45.7	1,607.75
ELF4-HFC Col rep 4	| Symbols: PHYE | phytochrome E | chr4:10042312-10045948 REVERSE LENGTH=1112	AT4G18130.1	TAIR10_20101214.fasta	122,519.10	100.00%	19	22	29	0.15%	26.70%	GGLVQPFGCLIAVEEPSFR	99.70%	53.9	31.1	42.1	1,405.76
ELF4-HFC Col rep 4	| Symbols: PHYE | phytochrome E | chr4:10042312-10045948 REVERSE LENGTH=1112	AT4G18130.1	TAIR10_20101214.fasta	122,519.10	100.00%	19	22	29	0.15%	26.70%	LLESSEISASQR	99.70%	70.3	30.5	26.4	1,226.67
ELF4-HFC Col rep 4	| Symbols: PHYE | phytochrome E | chr4:10042312-10045948 REVERSE LENGTH=1112	AT4G18130.1	TAIR10_20101214.fasta	122,519.10	100.00%	19	22	29	0.15%	26.70%	LQLILADLLR	99.70%	51.3	25	51.8	1,045.59
ELF4-HFC Col rep 4	| Symbols: PHYE | phytochrome E | chr4:10042312-10045948 REVERSE LENGTH=1112	AT4G18130.1	TAIR10_20101214.fasta	122,519.10	100.00%	19	22	29	0.15%	26.70%	LQSLPGGDIGALCDTVVEDVQR	99.70%	118	31.8	64	1,916.96
ELF4-HFC Col rep 4	| Symbols: PHYE | phytochrome E | chr4:10042312-10045948 REVERSE LENGTH=1112	AT4G18130.1	TAIR10_20101214.fasta	122,519.10	100.00%	19	22	29	0.15%	26.70%	NIVNHAPFPNSWVGISISPGQELSR	99.70%	43	31	61.6	1,164.60
ELF4-HFC Col rep 4	| Symbols: PHYE | phytochrome E | chr4:10042312-10045948 REVERSE LENGTH=1112	AT4G18130.1	TAIR10_20101214.fasta	122,519.10	100.00%	19	22	29	0.15%	26.70%	QITTIIESTDLK	99.70%	55.9	28.6	83.8	1,612.72
ELF4-HFC Col rep 4	| Symbols: PHYE | phytochrome E | chr4:10042312-10045948 REVERSE LENGTH=1112	AT4G18130.1	TAIR10_20101214.fasta	122,519.10	100.00%	19	22	29	0.15%	26.70%	SGDPALTLAGAVQSQK	99.70%	54.6	29.4	33.8	1,097.56
ELF4-HFC Col rep 4	| Symbols: PHYE | phytochrome E | chr4:10042312-10045948 REVERSE LENGTH=1112	AT4G18130.1	TAIR10_20101214.fasta	122,519.10	100.00%	19	22	29	0.15%	26.70%	SLADEIVQEESR	99.70%	77.8	29.9	35.8	1,333.59
ELF4-HFC Col rep 4	| Symbols: PHYE | phytochrome E | chr4:10042312-10045948 REVERSE LENGTH=1112	AT4G18130.1	TAIR10_20101214.fasta	122,519.10	100.00%	19	22	29	0.15%	26.70%	SSFTAFLEVAK	99.70%	56	27.3	62.3	1,388.68
ELF4-HFC Col rep 4	| Symbols: PHYE | phytochrome E | chr4:10042312-10045948 REVERSE LENGTH=1112	AT4G18130.1	TAIR10_20101214.fasta	122,519.10	100.00%	19	22	29	0.15%	26.70%	SVISPPNHVPDEHITAYLSNIQR	99.70%	39.3	30.8	52.5	1,290.67
ELF4-HFC Col rep 4	| Symbols: PHYE | phytochrome E | chr4:10042312-10045948 REVERSE LENGTH=1112	AT4G18130.1	TAIR10_20101214.fasta	122,519.10	100.00%	19	22	29	0.15%	26.70%	TLFTPSSGASLSK	99.70%	42.6	28.9	42.5	1,293.64
ELF4-HFC Col rep 4	| Symbols: PHYE | phytochrome E | chr4:10042312-10045948 REVERSE LENGTH=1112	AT4G18130.1	TAIR10_20101214.fasta	122,519.10	100.00%	19	22	29	0.15%	26.70%	YIEASLTANK	99.70%	46.9	28.8	0	2,527.26
ELF4-HFC Col rep 4	| Symbols: SPA1 | SPA (suppressor of phyA-105) protein family | chr2:19022572-19026821 REVERSE LENGTH=1029	AT2G46340.1	TAIR10_20101214.fasta	114,689.10	100.00%	9	10	13	0.07%	16.70%	LSVEELTGNYR	99.70%	59.5	29.4	34.9	1,074.51
ELF4-HFC Col rep 4	| Symbols: SPA1 | SPA (suppressor of phyA-105) protein family | chr2:19022572-19026821 REVERSE LENGTH=1029	AT2G46340.1	TAIR10_20101214.fasta	114,689.10	100.00%	9	10	13	0.07%	16.70%	NDLESDVDEDLNR	99.70%	76.8	25.4	78.9	1,614.83
ELF4-HFC Col rep 4	| Symbols: SPA1 | SPA (suppressor of phyA-105) protein family | chr2:19022572-19026821 REVERSE LENGTH=1029	AT2G46340.1	TAIR10_20101214.fasta	114,689.10	100.00%	9	10	13	0.07%	16.70%	NQETPPEFVSDQDLGSK	99.70%	64.8	29.2	43.2	1,004.54
ELF4-HFC Col rep 4	| Symbols: SPA1 | SPA (suppressor of phyA-105) protein family | chr2:19022572-19026821 REVERSE LENGTH=1029	AT2G46340.1	TAIR10_20101214.fasta	114,689.10	100.00%	9	10	13	0.07%	16.70%	NYLASTDYDGVVQIWDAGTGQGFSQYTEHQK	96.60%	32.1	30.6	54.3	1,954.08
ELF4-HFC Col rep 4	| Symbols: SPA1 | SPA (suppressor of phyA-105) protein family | chr2:19022572-19026821 REVERSE LENGTH=1029	AT2G46340.1	TAIR10_20101214.fasta	114,689.10	100.00%	9	10	13	0.07%	16.70%	SGDLLNSASVVCSLSFDPDEEHIAAAGISK	99.70%	89.5	31.5	20.9	1,257.65
ELF4-HFC Col rep 4	| Symbols: SPA1 | SPA (suppressor of phyA-105) protein family | chr2:19022572-19026821 REVERSE LENGTH=1029	AT2G46340.1	TAIR10_20101214.fasta	114,689.10	100.00%	9	10	13	0.07%	16.70%	SSDQLEVFFEGLCK	99.70%	52.8	28.8	14.6	2,289.06
ELF4-HFC Col rep 4	| Symbols: SPA1 | SPA (suppressor of phyA-105) protein family | chr2:19022572-19026821 REVERSE LENGTH=1029	AT2G46340.1	TAIR10_20101214.fasta	114,689.10	100.00%	9	10	13	0.07%	16.70%	TNSSGLSPGACSLTYK	99.70%	40.5	29.1	61.8	1,509.75
ELF4-HFC Col rep 4	| Symbols: SPA1 | SPA (suppressor of phyA-105) protein family | chr2:19022572-19026821 REVERSE LENGTH=1029	AT2G46340.1	TAIR10_20101214.fasta	114,689.10	100.00%	9	10	13	0.07%	16.70%	TPVSSSNFSQLLLK	99.70%	88.7	28.1	35.5	1,764.89
ELF4-HFC Col rep 4	| Symbols: SPA1 | SPA (suppressor of phyA-105) protein family | chr2:19022572-19026821 REVERSE LENGTH=1029	AT2G46340.1	TAIR10_20101214.fasta	114,689.10	100.00%	9	10	13	0.07%	16.70%	VQSSPLDEHCTTSSALFVPTANTDR	99.40%	32.8	31.5	27.9	2,656.15
ELF4-HFC Col rep 4	| Symbols: TIC | time for coffee | chr3:7913181-7918879 FORWARD LENGTH=1550	AT3G22380.1,AT3G22380.2	TAIR10_20101214.fasta	164,452.80	100.00%	4	4	5	0.03%	4.06%	DSPEEEGPVELPEAAR	99.70%	40	29.4	46.2	2,076.06
ELF4-HFC Col rep 4	| Symbols: TIC | time for coffee | chr3:7913181-7918879 FORWARD LENGTH=1550	AT3G22380.1,AT3G22380.2	TAIR10_20101214.fasta	164,452.80	100.00%	4	4	5	0.03%	4.06%	QDLADASVSAVTSGSIVDSSSR	99.70%	75	31.3	59.7	1,319.68
ELF4-HFC Col rep 4	| Symbols: TIC | time for coffee | chr3:7913181-7918879 FORWARD LENGTH=1550	AT3G22380.1,AT3G22380.2	TAIR10_20101214.fasta	164,452.80	100.00%	4	4	5	0.03%	4.06%	SQQQQQLPK	95.10%	25.9	26.6	39.2	1,167.75
ELF4-HFC Col rep 4	| Symbols: TIC | time for coffee | chr3:7913181-7918879 FORWARD LENGTH=1550	AT3G22380.1,AT3G22380.2	TAIR10_20101214.fasta	164,452.80	100.00%	4	4	5	0.03%	4.06%	SSPSPAPVSPLVSTWK	99.70%	66.7	28.4	113	2,342.17
ELF4-HFC Col rep 4	| Symbols: TZP | zinc knuckle (CCHC-type) family protein | chr5:17527261-17530207 FORWARD LENGTH=831	AT5G43630.1	TAIR10_20101214.fasta	90,615.60	100.00%	21	26	29	0.15%	31.40%	AVFDAVQVLR	99.70%	63.5	28.4	29	2,719.40
ELF4-HFC Col rep 4	| Symbols: TZP | zinc knuckle (CCHC-type) family protein | chr5:17527261-17530207 FORWARD LENGTH=831	AT5G43630.1	TAIR10_20101214.fasta	90,615.60	100.00%	21	26	29	0.15%	31.40%	AWWQSAGK	99.00%	38.5	28.8	43.7	1,361.75
ELF4-HFC Col rep 4	| Symbols: TZP | zinc knuckle (CCHC-type) family protein | chr5:17527261-17530207 FORWARD LENGTH=831	AT5G43630.1	TAIR10_20101214.fasta	90,615.60	100.00%	21	26	29	0.15%	31.40%	CFQLSHWAATCPNAPLYGSGAEGR	99.70%	54	29.8	43	1,542.81
ELF4-HFC Col rep 4	| Symbols: TZP | zinc knuckle (CCHC-type) family protein | chr5:17527261-17530207 FORWARD LENGTH=831	AT5G43630.1	TAIR10_20101214.fasta	90,615.60	100.00%	21	26	29	0.15%	31.40%	DCLEVTDTELR	99.70%	56.7	28.4	57.6	1,375.67
ELF4-HFC Col rep 4	| Symbols: TZP | zinc knuckle (CCHC-type) family protein | chr5:17527261-17530207 FORWARD LENGTH=831	AT5G43630.1	TAIR10_20101214.fasta	90,615.60	100.00%	21	26	29	0.15%	31.40%	DLICFYCGK	99.70%	39.3	25	48.9	1,199.63
ELF4-HFC Col rep 4	| Symbols: TZP | zinc knuckle (CCHC-type) family protein | chr5:17527261-17530207 FORWARD LENGTH=831	AT5G43630.1	TAIR10_20101214.fasta	90,615.60	100.00%	21	26	29	0.15%	31.40%	DLLVNEQLR	99.70%	44	26.1	34.7	2,587.33
ELF4-HFC Col rep 4	| Symbols: TZP | zinc knuckle (CCHC-type) family protein | chr5:17527261-17530207 FORWARD LENGTH=831	AT5G43630.1	TAIR10_20101214.fasta	90,615.60	100.00%	21	26	29	0.15%	31.40%	DLVQNISVR	99.70%	47.8	28.8	25.3	1,295.68
ELF4-HFC Col rep 4	| Symbols: TZP | zinc knuckle (CCHC-type) family protein | chr5:17527261-17530207 FORWARD LENGTH=831	AT5G43630.1	TAIR10_20101214.fasta	90,615.60	100.00%	21	26	29	0.15%	31.40%	EANASASDAAK	97.40%	32.3	26.4	40.2	1,109.58
ELF4-HFC Col rep 4	| Symbols: TZP | zinc knuckle (CCHC-type) family protein | chr5:17527261-17530207 FORWARD LENGTH=831	AT5G43630.1	TAIR10_20101214.fasta	90,615.60	100.00%	21	26	29	0.15%	31.40%	GDDLSSSGNEIGPVAEPNISSGK	99.70%	96.8	30.6	47.8	1,393.73
ELF4-HFC Col rep 4	| Symbols: TZP | zinc knuckle (CCHC-type) family protein | chr5:17527261-17530207 FORWARD LENGTH=831	AT5G43630.1	TAIR10_20101214.fasta	90,615.60	100.00%	21	26	29	0.15%	31.40%	GDYLPEGEAGPSGSYR	99.70%	64.7	27.1	67.7	1,533.67
ELF4-HFC Col rep 4	| Symbols: TZP | zinc knuckle (CCHC-type) family protein | chr5:17527261-17530207 FORWARD LENGTH=831	AT5G43630.1	TAIR10_20101214.fasta	90,615.60	100.00%	21	26	29	0.15%	31.40%	GVTCLVESQFISNQDFLEEELK	99.70%	102	31.6	51.4	1,890.87
ELF4-HFC Col rep 4	| Symbols: TZP | zinc knuckle (CCHC-type) family protein | chr5:17527261-17530207 FORWARD LENGTH=831	AT5G43630.1	TAIR10_20101214.fasta	90,615.60	100.00%	21	26	29	0.15%	31.40%	IDGDTEGQSSR	99.70%	61.3	25.7	26.6	3,478.58
ELF4-HFC Col rep 4	| Symbols: TZP | zinc knuckle (CCHC-type) family protein | chr5:17527261-17530207 FORWARD LENGTH=831	AT5G43630.1	TAIR10_20101214.fasta	90,615.60	100.00%	21	26	29	0.15%	31.40%	KRPGFEEQLIFGSK	99.70%	68.2	28.5	82.9	3,089.47
ELF4-HFC Col rep 4	| Symbols: TZP | zinc knuckle (CCHC-type) family protein | chr5:17527261-17530207 FORWARD LENGTH=831	AT5G43630.1	TAIR10_20101214.fasta	90,615.60	100.00%	21	26	29	0.15%	31.40%	LPISGFTDVPR	99.70%	76.8	29.4	48.1	1,658.77
ELF4-HFC Col rep 4	| Symbols: TZP | zinc knuckle (CCHC-type) family protein | chr5:17527261-17530207 FORWARD LENGTH=831	AT5G43630.1	TAIR10_20101214.fasta	90,615.60	100.00%	21	26	29	0.15%	31.40%	NGREEASSLCIR	99.70%	38.4	28.9	29.1	1,642.77
ELF4-HFC Col rep 4	| Symbols: TZP | zinc knuckle (CCHC-type) family protein | chr5:17527261-17530207 FORWARD LENGTH=831	AT5G43630.1	TAIR10_20101214.fasta	90,615.60	100.00%	21	26	29	0.15%	31.40%	NTGFQSFFQSIYCPK	99.70%	98	29.5	75.6	1,520.83
ELF4-HFC Col rep 4	| Symbols: TZP | zinc knuckle (CCHC-type) family protein | chr5:17527261-17530207 FORWARD LENGTH=831	AT5G43630.1	TAIR10_20101214.fasta	90,615.60	100.00%	21	26	29	0.15%	31.40%	RPGFEEQLIFGSK	99.70%	52.4	29.8	27.8	2,733.28
ELF4-HFC Col rep 4	| Symbols: TZP | zinc knuckle (CCHC-type) family protein | chr5:17527261-17530207 FORWARD LENGTH=831	AT5G43630.1	TAIR10_20101214.fasta	90,615.60	100.00%	21	26	29	0.15%	31.40%	SVSGLEGFFLR	99.70%	57.6	27.8	29.2	1,724.80
ELF4-HFC Col rep 4	| Symbols: TZP | zinc knuckle (CCHC-type) family protein | chr5:17527261-17530207 FORWARD LENGTH=831	AT5G43630.1	TAIR10_20101214.fasta	90,615.60	100.00%	21	26	29	0.15%	31.40%	TLNQECLGSTSK	99.70%	62.3	29.5	69.9	2,152.04
ELF4-HFC Col rep 4	| Symbols: TZP | zinc knuckle (CCHC-type) family protein | chr5:17527261-17530207 FORWARD LENGTH=831	AT5G43630.1	TAIR10_20101214.fasta	90,615.60	100.00%	21	26	29	0.15%	31.40%	TSGYDGIPSAEELSR	99.70%	52.3	29.8	17.5	1,084.57
ELF4-HFC Col rep 4	| Symbols: TZP | zinc knuckle (CCHC-type) family protein | chr5:17527261-17530207 FORWARD LENGTH=831	AT5G43630.1	TAIR10_20101214.fasta	90,615.60	100.00%	21	26	29	0.15%	31.40%	VVFNQTSK	97.70%	29.4	27.8	53.5	1,639.87
ELF4-HFC elf3-1 rep 1	| Symbols: ELF4 | Protein of unknown function (DUF1313) | chr2:16734545-16734880 REVERSE LENGTH=111	AT2G40080.1	TAIR10_20101214.fasta	12,375.40	100.00%	7	27	227	1.11%	79.30%	NVAEEAEQGEDPAMWENLDR	99.70%	123	25.4	51.1	1,117.64
ELF4-HFC elf3-1 rep 1	| Symbols: ELF4 | Protein of unknown function (DUF1313) | chr2:16734545-16734880 REVERSE LENGTH=111	AT2G40080.1	TAIR10_20101214.fasta	12,375.40	100.00%	7	27	227	1.11%	79.30%	NVALIQELNGNISK	99.70%	121	27.5	22.4	933.4577
ELF4-HFC elf3-1 rep 1	| Symbols: ELF4 | Protein of unknown function (DUF1313) | chr2:16734545-16734880 REVERSE LENGTH=111	AT2G40080.1	TAIR10_20101214.fasta	12,375.40	100.00%	7	27	227	1.11%	79.30%	QVQSVLDR	99.00%	52.7	29.3	41.2	2,650.19
ELF4-HFC elf3-1 rep 1	| Symbols: ELF4 | Protein of unknown function (DUF1313) | chr2:16734545-16734880 REVERSE LENGTH=111	AT2G40080.1	TAIR10_20101214.fasta	12,375.40	100.00%	7	27	227	1.11%	79.30%	RNVAEEAEQGEDPAMWENLDR	99.70%	83	28	34.9	1,350.62
ELF4-HFC elf3-1 rep 1	| Symbols: ELF4 | Protein of unknown function (DUF1313) | chr2:16734545-16734880 REVERSE LENGTH=111	AT2G40080.1	TAIR10_20101214.fasta	12,375.40	100.00%	7	27	227	1.11%	79.30%	SLIQQVNDNHQSR	99.70%	75.7	30.1	30	1,175.52
ELF4-HFC elf3-1 rep 1	| Symbols: ELF4 | Protein of unknown function (DUF1313) | chr2:16734545-16734880 REVERSE LENGTH=111	AT2G40080.1	TAIR10_20101214.fasta	12,375.40	100.00%	7	27	227	1.11%	79.30%	VVNMYSDLNTSFSSGFHGGK	99.70%	100	28.4	20.9	1,099.61
ELF4-HFC elf3-1 rep 1	| Symbols: ELF4 | Protein of unknown function (DUF1313) | chr2:16734545-16734880 REVERSE LENGTH=111	AT2G40080.1	TAIR10_20101214.fasta	12,375.40	100.00%	7	27	227	1.11%	79.30%	VVNMYSDLNTSFSSGFHGGKNGHDGGGAAGTR	99.70%	77.2	28.4	31	1,043.58
ELF4-HFC elf3-1 rep 2	| Symbols: ELF4 | Protein of unknown function (DUF1313) | chr2:16734545-16734880 REVERSE LENGTH=111	AT2G40080.1	TAIR10_20101214.fasta	12,375.40	100.00%	8	26	203	1.02%	79.30%	NGHDGGGAAGTR	94.70%	21	26.2	23.8	1,034.47
ELF4-HFC elf3-1 rep 2	| Symbols: ELF4 | Protein of unknown function (DUF1313) | chr2:16734545-16734880 REVERSE LENGTH=111	AT2G40080.1	TAIR10_20101214.fasta	12,375.40	100.00%	8	26	203	1.02%	79.30%	NVAEEAEQGEDPAMWENLDR	99.70%	130	25.4	92.6	2,230.05
ELF4-HFC elf3-1 rep 2	| Symbols: ELF4 | Protein of unknown function (DUF1313) | chr2:16734545-16734880 REVERSE LENGTH=111	AT2G40080.1	TAIR10_20101214.fasta	12,375.40	100.00%	8	26	203	1.02%	79.30%	NVALIQELNGNISK	99.70%	118	27.5	56.5	1,654.73
ELF4-HFC elf3-1 rep 2	| Symbols: ELF4 | Protein of unknown function (DUF1313) | chr2:16734545-16734880 REVERSE LENGTH=111	AT2G40080.1	TAIR10_20101214.fasta	12,375.40	100.00%	8	26	203	1.02%	79.30%	QVQSVLDR	99.00%	56.8	29.3	94.8	2,585.24
ELF4-HFC elf3-1 rep 2	| Symbols: ELF4 | Protein of unknown function (DUF1313) | chr2:16734545-16734880 REVERSE LENGTH=111	AT2G40080.1	TAIR10_20101214.fasta	12,375.40	100.00%	8	26	203	1.02%	79.30%	RNVAEEAEQGEDPAMWENLDR	99.70%	84.3	28.2	53.9	1,164.51
ELF4-HFC elf3-1 rep 2	| Symbols: ELF4 | Protein of unknown function (DUF1313) | chr2:16734545-16734880 REVERSE LENGTH=111	AT2G40080.1	TAIR10_20101214.fasta	12,375.40	100.00%	8	26	203	1.02%	79.30%	SLIQQVNDNHQSR	99.70%	83.3	30.2	58.5	1,635.89
ELF4-HFC elf3-1 rep 2	| Symbols: ELF4 | Protein of unknown function (DUF1313) | chr2:16734545-16734880 REVERSE LENGTH=111	AT2G40080.1	TAIR10_20101214.fasta	12,375.40	100.00%	8	26	203	1.02%	79.30%	VVNMYSDLNTSFSSGFHGGK	99.70%	118	29.6	68	1,201.66
ELF4-HFC elf3-1 rep 2	| Symbols: ELF4 | Protein of unknown function (DUF1313) | chr2:16734545-16734880 REVERSE LENGTH=111	AT2G40080.1	TAIR10_20101214.fasta	12,375.40	100.00%	8	26	203	1.02%	79.30%	VVNMYSDLNTSFSSGFHGGKNGHDGGGAAGTR	99.70%	75.4	29.4	20.5	1,392.65
ELF4-HFC elf3-1 rep 3	| Symbols: ELF4 | Protein of unknown function (DUF1313) | chr2:16734545-16734880 REVERSE LENGTH=111	AT2G40080.1	TAIR10_20101214.fasta	12,375.40	100.00%	7	26	187	0.92%	79.30%	NVAEEAEQGEDPAMWENLDR	99.70%	120	25.2	83.6	1,823.84
ELF4-HFC elf3-1 rep 3	| Symbols: ELF4 | Protein of unknown function (DUF1313) | chr2:16734545-16734880 REVERSE LENGTH=111	AT2G40080.1	TAIR10_20101214.fasta	12,375.40	100.00%	7	26	187	0.92%	79.30%	NVALIQELNGNISK	99.70%	124	28.7	40.4	1,507.79
ELF4-HFC elf3-1 rep 3	| Symbols: ELF4 | Protein of unknown function (DUF1313) | chr2:16734545-16734880 REVERSE LENGTH=111	AT2G40080.1	TAIR10_20101214.fasta	12,375.40	100.00%	7	26	187	0.92%	79.30%	QVQSVLDR	99.00%	50.3	29.3	35.7	1,211.64
ELF4-HFC elf3-1 rep 3	| Symbols: ELF4 | Protein of unknown function (DUF1313) | chr2:16734545-16734880 REVERSE LENGTH=111	AT2G40080.1	TAIR10_20101214.fasta	12,375.40	100.00%	7	26	187	0.92%	79.30%	RNVAEEAEQGEDPAMWENLDR	99.70%	73.4	28.1	53.9	1,337.64
ELF4-HFC elf3-1 rep 3	| Symbols: ELF4 | Protein of unknown function (DUF1313) | chr2:16734545-16734880 REVERSE LENGTH=111	AT2G40080.1	TAIR10_20101214.fasta	12,375.40	100.00%	7	26	187	0.92%	79.30%	SLIQQVNDNHQSR	99.70%	70.5	30.2	31	1,581.74
ELF4-HFC elf3-1 rep 3	| Symbols: ELF4 | Protein of unknown function (DUF1313) | chr2:16734545-16734880 REVERSE LENGTH=111	AT2G40080.1	TAIR10_20101214.fasta	12,375.40	100.00%	7	26	187	0.92%	79.30%	VVNMYSDLNTSFSSGFHGGK	99.70%	97.1	28.8	21.8	922.4992
ELF4-HFC elf3-1 rep 3	| Symbols: ELF4 | Protein of unknown function (DUF1313) | chr2:16734545-16734880 REVERSE LENGTH=111	AT2G40080.1	TAIR10_20101214.fasta	12,375.40	100.00%	7	26	187	0.92%	79.30%	VVNMYSDLNTSFSSGFHGGKNGHDGGGAAGTR	99.70%	65.8	29.6	39.2	1,667.79
ELF4-HFC phyb9 rep 1	| Symbols: ELF3, PYK20 | hydroxyproline-rich glycoprotein family protein | chr2:11059459-11063178 FORWARD LENGTH=695	AT2G25930.1	TAIR10_20101214.fasta	77,204.80	100.00%	10	12	16	0.08%	23.60%	EEEDFAVPVYINSR	99.70%	57.9	30	22.8	1,149.59
ELF4-HFC phyb9 rep 1	| Symbols: ELF3, PYK20 | hydroxyproline-rich glycoprotein family protein | chr2:11059459-11063178 FORWARD LENGTH=695	AT2G25930.1	TAIR10_20101214.fasta	77,204.80	100.00%	10	12	16	0.08%	23.60%	FQEVNQTGSK	99.70%	38.3	29.4	84.4	1,915.08
ELF4-HFC phyb9 rep 1	| Symbols: ELF3, PYK20 | hydroxyproline-rich glycoprotein family protein | chr2:11059459-11063178 FORWARD LENGTH=695	AT2G25930.1	TAIR10_20101214.fasta	77,204.80	100.00%	10	12	16	0.08%	23.60%	IFQSIQEER	99.70%	39	30	75.7	1,618.77
ELF4-HFC phyb9 rep 1	| Symbols: ELF3, PYK20 | hydroxyproline-rich glycoprotein family protein | chr2:11059459-11063178 FORWARD LENGTH=695	AT2G25930.1	TAIR10_20101214.fasta	77,204.80	100.00%	10	12	16	0.08%	23.60%	LIAASPDLLLDEISFLGK	99.70%	108	25.7	69.8	1,695.79
ELF4-HFC phyb9 rep 1	| Symbols: ELF3, PYK20 | hydroxyproline-rich glycoprotein family protein | chr2:11059459-11063178 FORWARD LENGTH=695	AT2G25930.1	TAIR10_20101214.fasta	77,204.80	100.00%	10	12	16	0.08%	23.60%	NLSVQHLDSSAANQATEK	99.70%	97	31	69.5	3,316.54
ELF4-HFC phyb9 rep 1	| Symbols: ELF3, PYK20 | hydroxyproline-rich glycoprotein family protein | chr2:11059459-11063178 FORWARD LENGTH=695	AT2G25930.1	TAIR10_20101214.fasta	77,204.80	100.00%	10	12	16	0.08%	23.60%	QGSTGSSPSGPQGISGSK	99.70%	90.4	29.1	12.1	2,224.04
ELF4-HFC phyb9 rep 1	| Symbols: ELF3, PYK20 | hydroxyproline-rich glycoprotein family protein | chr2:11059459-11063178 FORWARD LENGTH=695	AT2G25930.1	TAIR10_20101214.fasta	77,204.80	100.00%	10	12	16	0.08%	23.60%	SDNEPAPQQQQQPTK	99.70%	59.3	29.7	12.9	1,358.76
ELF4-HFC phyb9 rep 1	| Symbols: ELF3, PYK20 | hydroxyproline-rich glycoprotein family protein | chr2:11059459-11063178 FORWARD LENGTH=695	AT2G25930.1	TAIR10_20101214.fasta	77,204.80	100.00%	10	12	16	0.08%	23.60%	SFRPFAAVDEDSNINNAPEQTMTTTTTTTR	99.70%	65.6	31	129	2,318.98
ELF4-HFC phyb9 rep 1	| Symbols: ELF3, PYK20 | hydroxyproline-rich glycoprotein family protein | chr2:11059459-11063178 FORWARD LENGTH=695	AT2G25930.1	TAIR10_20101214.fasta	77,204.80	100.00%	10	12	16	0.08%	23.60%	SNNTSTLVHPGPSSQPCGVER	99.60%	32.1	30.4	32.9	1,513.82
ELF4-HFC phyb9 rep 1	| Symbols: ELF3, PYK20 | hydroxyproline-rich glycoprotein family protein | chr2:11059459-11063178 FORWARD LENGTH=695	AT2G25930.1	TAIR10_20101214.fasta	77,204.80	100.00%	10	12	16	0.08%	23.60%	VFAVQLFELHR	99.70%	35.5	28.5	13.5	944.516
ELF4-HFC phyb9 rep 1	| Symbols: ELF4 | Protein of unknown function (DUF1313) | chr2:16734545-16734880 REVERSE LENGTH=111	AT2G40080.1	TAIR10_20101214.fasta	12,375.40	100.00%	5	20	90	0.44%	61.30%	NVAEEAEQGEDPAMWENLDR	99.70%	124	26.5	69.4	2,475.08
ELF4-HFC phyb9 rep 1	| Symbols: ELF4 | Protein of unknown function (DUF1313) | chr2:16734545-16734880 REVERSE LENGTH=111	AT2G40080.1	TAIR10_20101214.fasta	12,375.40	100.00%	5	20	90	0.44%	61.30%	NVALIQELNGNISK	99.70%	107	27.5	14.4	1,539.75
ELF4-HFC phyb9 rep 1	| Symbols: ELF4 | Protein of unknown function (DUF1313) | chr2:16734545-16734880 REVERSE LENGTH=111	AT2G40080.1	TAIR10_20101214.fasta	12,375.40	100.00%	5	20	90	0.44%	61.30%	RNVAEEAEQGEDPAMWENLDR	99.70%	81.9	28.8	113	2,146.99
ELF4-HFC phyb9 rep 1	| Symbols: ELF4 | Protein of unknown function (DUF1313) | chr2:16734545-16734880 REVERSE LENGTH=111	AT2G40080.1	TAIR10_20101214.fasta	12,375.40	100.00%	5	20	90	0.44%	61.30%	SLIQQVNDNHQSR	99.70%	77.5	30.1	6.16	1,197.70
ELF4-HFC phyb9 rep 1	| Symbols: ELF4 | Protein of unknown function (DUF1313) | chr2:16734545-16734880 REVERSE LENGTH=111	AT2G40080.1	TAIR10_20101214.fasta	12,375.40	100.00%	5	20	90	0.44%	61.30%	VVNMYSDLNTSFSSGFHGGK	99.70%	98.7	29.5	67.1	2,036.84
ELF4-HFC phyb9 rep 1	| Symbols: PCL1, LUX | Homeodomain-like superfamily protein | chr3:17183248-17184219 FORWARD LENGTH=323	AT3G46640.1,AT3G46640.2	TAIR10_20101214.fasta	34,648.50	100.00%	2	3	5	0.02%	15.20%	ENVASHLQK	99.70%	43.9	28.1	19.9	1,121.65
ELF4-HFC phyb9 rep 1	| Symbols: PCL1, LUX | Homeodomain-like superfamily protein | chr3:17183248-17184219 FORWARD LENGTH=323	AT3G46640.1,AT3G46640.2	TAIR10_20101214.fasta	34,648.50	100.00%	2	3	5	0.02%	15.20%	FVDVVAHLGIK	99.70%	28	25	49.3	1,516.78
ELF4-HFC phyb9 rep 1	| Symbols: PCL1, LUX | Homeodomain-like superfamily protein | chr3:17183248-17184219 FORWARD LENGTH=323	AT3G46640.1,AT3G46640.2	TAIR10_20101214.fasta	34,648.50	100.00%	2	3	5	0.02%	15.20%	GGSSGPNTSSSNNNVEEEDR	99.70%	72.6	25	10.9	1,675.94
ELF4-HFC phyb9 rep 1	| Symbols: PCL1, LUX | Homeodomain-like superfamily protein | chr3:17183248-17184219 FORWARD LENGTH=323	AT3G46640.1,AT3G46640.2	TAIR10_20101214.fasta	34,648.50	100.00%	2	3	5	0.02%	15.20%	LVWTPQLHK	98.10%	25.4	25.4	60.1	1,570.77
ELF4-HFC phyb9 rep 1	| Symbols: | C2 calcium/lipid-binding plant phosphoribosyltransferase family protein | chr5:2127200-2129584 REVERSE LENGTH=794	AT5G06850.1	TAIR10_20101214.fasta	91,004.50	100.00%	3	3	4	0.02%	6.42%	IYTHSYPLLVLQTK	99.70%	40.9	27.3	0	1,525.78
ELF4-HFC phyb9 rep 1	| Symbols: | C2 calcium/lipid-binding plant phosphoribosyltransferase family protein | chr5:2127200-2129584 REVERSE LENGTH=794	AT5G06850.1	TAIR10_20101214.fasta	91,004.50	100.00%	3	3	4	0.02%	6.42%	LISPLSVFEK	99.70%	52.2	26.3	96.1	2,571.14
ELF4-HFC phyb9 rep 1	| Symbols: | C2 calcium/lipid-binding plant phosphoribosyltransferase family protein | chr5:2127200-2129584 REVERSE LENGTH=794	AT5G06850.1	TAIR10_20101214.fasta	91,004.50	100.00%	3	3	4	0.02%	6.42%	VNVIEAQDVEPSDR	99.70%	53.4	29.7	44.8	1,667.79
ELF4-HFC phyb9 rep 1	| Symbols: | C2 calcium/lipid-binding plant phosphoribosyltransferase family protein | chr5:2127200-2129584 REVERSE LENGTH=794	AT5G06850.1	TAIR10_20101214.fasta	91,004.50	100.00%	3	3	4	0.02%	6.42%	VPPDSPLAPQWYR	99.70%	48.2	30	21	1,137.55
ELF4-HFC phyb9 rep 2	| Symbols: ELF3, PYK20 | hydroxyproline-rich glycoprotein family protein | chr2:11059459-11063178 FORWARD LENGTH=695	AT2G25930.1	TAIR10_20101214.fasta	77,204.80	100.00%	8	8	10	0.05%	19.60%	EEEDFAVPVYINSR	99.70%	48.5	30	22.8	1,149.59
ELF4-HFC phyb9 rep 2	| Symbols: ELF3, PYK20 | hydroxyproline-rich glycoprotein family protein | chr2:11059459-11063178 FORWARD LENGTH=695	AT2G25930.1	TAIR10_20101214.fasta	77,204.80	100.00%	8	8	10	0.05%	19.60%	IFQSIQEER	99.70%	42.8	30	89.6	1,915.08
ELF4-HFC phyb9 rep 2	| Symbols: ELF3, PYK20 | hydroxyproline-rich glycoprotein family protein | chr2:11059459-11063178 FORWARD LENGTH=695	AT2G25930.1	TAIR10_20101214.fasta	77,204.80	100.00%	8	8	10	0.05%	19.60%	LIAASPDLLLDEISFLGK	99.70%	98	25.3	92.6	1,912.94
ELF4-HFC phyb9 rep 2	| Symbols: ELF3, PYK20 | hydroxyproline-rich glycoprotein family protein | chr2:11059459-11063178 FORWARD LENGTH=695	AT2G25930.1	TAIR10_20101214.fasta	77,204.80	100.00%	8	8	10	0.05%	19.60%	QGSTGSSPSGPQGISGSK	99.70%	87.1	29.1	85.4	1,618.77
ELF4-HFC phyb9 rep 2	| Symbols: ELF3, PYK20 | hydroxyproline-rich glycoprotein family protein | chr2:11059459-11063178 FORWARD LENGTH=695	AT2G25930.1	TAIR10_20101214.fasta	77,204.80	100.00%	8	8	10	0.05%	19.60%	SDNEPAPQQQQQPTK	99.70%	81.9	29.4	51.4	1,695.79
ELF4-HFC phyb9 rep 2	| Symbols: ELF3, PYK20 | hydroxyproline-rich glycoprotein family protein | chr2:11059459-11063178 FORWARD LENGTH=695	AT2G25930.1	TAIR10_20101214.fasta	77,204.80	100.00%	8	8	10	0.05%	19.60%	SFRPFAAVDEDSNINNAPEQTMTTTTTTTR	99.70%	84.2	31	1.76	3,317.52
ELF4-HFC phyb9 rep 2	| Symbols: ELF3, PYK20 | hydroxyproline-rich glycoprotein family protein | chr2:11059459-11063178 FORWARD LENGTH=695	AT2G25930.1	TAIR10_20101214.fasta	77,204.80	100.00%	8	8	10	0.05%	19.60%	SNNTSTLVHPGPSSQPCGVER	95.10%	24.9	30.4	24.1	2,224.04
ELF4-HFC phyb9 rep 2	| Symbols: ELF3, PYK20 | hydroxyproline-rich glycoprotein family protein | chr2:11059459-11063178 FORWARD LENGTH=695	AT2G25930.1	TAIR10_20101214.fasta	77,204.80	100.00%	8	8	10	0.05%	19.60%	VFAVQLFELHR	99.70%	27.9	28.6	18.9	1,358.76
ELF4-HFC phyb9 rep 2	| Symbols: ELF4 | Protein of unknown function (DUF1313) | chr2:16734545-16734880 REVERSE LENGTH=111	AT2G40080.1	TAIR10_20101214.fasta	12,375.40	100.00%	6	23	88	0.44%	68.50%	NVAEEAEQGEDPAMWENLDR	99.70%	130	25.1	113	2,302.99
ELF4-HFC phyb9 rep 2	| Symbols: ELF4 | Protein of unknown function (DUF1313) | chr2:16734545-16734880 REVERSE LENGTH=111	AT2G40080.1	TAIR10_20101214.fasta	12,375.40	100.00%	6	23	88	0.44%	68.50%	NVALIQELNGNISK	99.70%	120	28.6	102	1,512.84
ELF4-HFC phyb9 rep 2	| Symbols: ELF4 | Protein of unknown function (DUF1313) | chr2:16734545-16734880 REVERSE LENGTH=111	AT2G40080.1	TAIR10_20101214.fasta	12,375.40	100.00%	6	23	88	0.44%	68.50%	QVQSVLDR	99.00%	50.9	29.3	8.03	944.516
ELF4-HFC phyb9 rep 2	| Symbols: ELF4 | Protein of unknown function (DUF1313) | chr2:16734545-16734880 REVERSE LENGTH=111	AT2G40080.1	TAIR10_20101214.fasta	12,375.40	100.00%	6	23	88	0.44%	68.50%	RNVAEEAEQGEDPAMWENLDR	99.70%	78.4	27.8	65.6	2,475.08
ELF4-HFC phyb9 rep 2	| Symbols: ELF4 | Protein of unknown function (DUF1313) | chr2:16734545-16734880 REVERSE LENGTH=111	AT2G40080.1	TAIR10_20101214.fasta	12,375.40	100.00%	6	23	88	0.44%	68.50%	SLIQQVNDNHQSR	99.70%	74.1	30	67.6	1,538.77

ELF4-HFC phyb9 rep 2	| Symbols: ELF4 | Protein of unknown function (DUF1313) | chr2:16734545-16734880 REVERSE LENGTH=111	AT2G40080.1	TAIR10_20101214.fasta	12,375.40	100.00%	6	23	88	0.44%	68.50%	VVNMYSDLNTSFSSGFHGGK	99.70%	113	29.7	89.7	2,146.99
ELF4-HFC phyb9 rep 2	| Symbols: PCL1, LUX | Homeodomain-like superfamily protein | chr3:17183248-17184219 FORWARD LENGTH=323	AT3G46640.1,AT3G46640.2	TAIR10_20101214.fasta	34,648.50	100.00%	2	2	3	0.02%	12.40%	FVDVVAHLGIK	99.50%	22.3	25	15.8	1,025.54
ELF4-HFC phyb9 rep 2	| Symbols: PCL1, LUX | Homeodomain-like superfamily protein | chr3:17183248-17184219 FORWARD LENGTH=323	AT3G46640.1,AT3G46640.2	TAIR10_20101214.fasta	34,648.50	100.00%	2	2	3	0.02%	12.40%	GGSSGPNTSSSNNNVEEEDR	99.70%	67.1	25	14.7	1,197.70
ELF4-HFC phyb9 rep 2	| Symbols: PCL1, LUX | Homeodomain-like superfamily protein | chr3:17183248-17184219 FORWARD LENGTH=323	AT3G46640.1,AT3G46640.2	TAIR10_20101214.fasta	34,648.50	100.00%	2	2	3	0.02%	12.40%	LVWTPQLHK	97.30%	24.4	25.4	94.4	2,036.84
ELF4-HFC phyb9 rep 2	| Symbols: | C2 calcium/lipid-binding plant phosphoribosyltransferase family protein | chr5:2127200-2129584 REVERSE LENGTH=794	AT5G06850.1	TAIR10_20101214.fasta	91,004.50	100.00%	4	4	6	0.03%	9.82%	IQMVVGDIATQGER	99.70%	58	30	15.5	1,121.65
ELF4-HFC phyb9 rep 2	| Symbols: | C2 calcium/lipid-binding plant phosphoribosyltransferase family protein | chr5:2127200-2129584 REVERSE LENGTH=794	AT5G06850.1	TAIR10_20101214.fasta	91,004.50	100.00%	4	4	6	0.03%	9.82%	IYTHSYPLLVLQTK	99.70%	31.1	27.2	44.6	1,516.78
ELF4-HFC phyb9 rep 2	| Symbols: | C2 calcium/lipid-binding plant phosphoribosyltransferase family protein | chr5:2127200-2129584 REVERSE LENGTH=794	AT5G06850.1	TAIR10_20101214.fasta	91,004.50	100.00%	4	4	6	0.03%	9.82%	VNVIEAQDVEPSDR	99.70%	67.2	29.8	18.7	1,675.94
ELF4-HFC phyb9 rep 2	| Symbols: | C2 calcium/lipid-binding plant phosphoribosyltransferase family protein | chr5:2127200-2129584 REVERSE LENGTH=794	AT5G06850.1	TAIR10_20101214.fasta	91,004.50	100.00%	4	4	6	0.03%	9.82%	VPPDSPLAPQWYR	99.60%	34	30.2	3.24	1,132.66
ELF4-HFC phyb9 rep 2	| Symbols: | C2 calcium/lipid-binding plant phosphoribosyltransferase family protein | chr5:2127200-2129584 REVERSE LENGTH=794	AT5G06850.1	TAIR10_20101214.fasta	91,004.50	100.00%	4	4	6	0.03%	9.82%	VSWAEAASPDELDEEFDTFPTSK	99.70%	96.1	29	55.7	1,570.77
ELF4-HFC phyb9 rep 3	| Symbols: ELF3, PYK20 | hydroxyproline-rich glycoprotein family protein | chr2:11059459-11063178 FORWARD LENGTH=695	AT2G25930.1	TAIR10_20101214.fasta	77,204.80	100.00%	10	11	12	0.06%	23.60%	EEEDFAVPVYINSR	99.70%	63.7	30.1	0	1,525.78
ELF4-HFC phyb9 rep 3	| Symbols: ELF3, PYK20 | hydroxyproline-rich glycoprotein family protein | chr2:11059459-11063178 FORWARD LENGTH=695	AT2G25930.1	TAIR10_20101214.fasta	77,204.80	100.00%	10	11	12	0.06%	23.60%	FQEVNQTGSK	99.70%	40.6	29.4	48	1,667.79
ELF4-HFC phyb9 rep 3	| Symbols: ELF3, PYK20 | hydroxyproline-rich glycoprotein family protein | chr2:11059459-11063178 FORWARD LENGTH=695	AT2G25930.1	TAIR10_20101214.fasta	77,204.80	100.00%	10	11	12	0.06%	23.60%	IFQSIQEER	99.70%	43.4	30.2	22.2	1,137.55
ELF4-HFC phyb9 rep 3	| Symbols: ELF3, PYK20 | hydroxyproline-rich glycoprotein family protein | chr2:11059459-11063178 FORWARD LENGTH=695	AT2G25930.1	TAIR10_20101214.fasta	77,204.80	100.00%	10	11	12	0.06%	23.60%	LIAASPDLLLDEISFLGK	99.70%	98.1	25.3	16.3	1,149.59
ELF4-HFC phyb9 rep 3	| Symbols: ELF3, PYK20 | hydroxyproline-rich glycoprotein family protein | chr2:11059459-11063178 FORWARD LENGTH=695	AT2G25930.1	TAIR10_20101214.fasta	77,204.80	100.00%	10	11	12	0.06%	23.60%	NLSVQHLDSSAANQATEK	99.70%	98	30.8	92	1,915.08
ELF4-HFC phyb9 rep 3	| Symbols: ELF3, PYK20 | hydroxyproline-rich glycoprotein family protein | chr2:11059459-11063178 FORWARD LENGTH=695	AT2G25930.1	TAIR10_20101214.fasta	77,204.80	100.00%	10	11	12	0.06%	23.60%	QGSTGSSPSGPQGISGSK	99.70%	94.5	29.1	93.8	1,912.94
ELF4-HFC phyb9 rep 3	| Symbols: ELF3, PYK20 | hydroxyproline-rich glycoprotein family protein | chr2:11059459-11063178 FORWARD LENGTH=695	AT2G25930.1	TAIR10_20101214.fasta	77,204.80	100.00%	10	11	12	0.06%	23.60%	SDNEPAPQQQQQPTK	99.70%	60.9	29.8	78.2	1,618.77
ELF4-HFC phyb9 rep 3	| Symbols: ELF3, PYK20 | hydroxyproline-rich glycoprotein family protein | chr2:11059459-11063178 FORWARD LENGTH=695	AT2G25930.1	TAIR10_20101214.fasta	77,204.80	100.00%	10	11	12	0.06%	23.60%	SFRPFAAVDEDSNINNAPEQTMTTTTTTTR	99.70%	68.8	31.5	48.7	1,695.79
ELF4-HFC phyb9 rep 3	| Symbols: ELF3, PYK20 | hydroxyproline-rich glycoprotein family protein | chr2:11059459-11063178 FORWARD LENGTH=695	AT2G25930.1	TAIR10_20101214.fasta	77,204.80	100.00%	10	11	12	0.06%	23.60%	SNNTSTLVHPGPSSQPCGVER	98.80%	28.7	30.6	7.38	3,317.52
ELF4-HFC phyb9 rep 3	| Symbols: ELF3, PYK20 | hydroxyproline-rich glycoprotein family protein | chr2:11059459-11063178 FORWARD LENGTH=695	AT2G25930.1	TAIR10_20101214.fasta	77,204.80	100.00%	10	11	12	0.06%	23.60%	VFAVQLFELHR	99.70%	31.2	28.4	23.8	2,224.04
ELF4-HFC phyb9 rep 3	| Symbols: ELF4 | Protein of unknown function (DUF1313) | chr2:16734545-16734880 REVERSE LENGTH=111	AT2G40080.1	TAIR10_20101214.fasta	12,375.40	100.00%	6	24	83	0.42%	68.50%	NVAEEAEQGEDPAMWENLDR	99.70%	116	26.8	21.6	1,358.76
ELF4-HFC phyb9 rep 3	| Symbols: ELF4 | Protein of unknown function (DUF1313) | chr2:16734545-16734880 REVERSE LENGTH=111	AT2G40080.1	TAIR10_20101214.fasta	12,375.40	100.00%	6	24	83	0.42%	68.50%	NVALIQELNGNISK	99.70%	112	27.4	123	2,302.99
ELF4-HFC phyb9 rep 3	| Symbols: ELF4 | Protein of unknown function (DUF1313) | chr2:16734545-16734880 REVERSE LENGTH=111	AT2G40080.1	TAIR10_20101214.fasta	12,375.40	100.00%	6	24	83	0.42%	68.50%	QVQSVLDR	99.00%	42.8	29.5	98.8	1,512.84
ELF4-HFC phyb9 rep 3	| Symbols: ELF4 | Protein of unknown function (DUF1313) | chr2:16734545-16734880 REVERSE LENGTH=111	AT2G40080.1	TAIR10_20101214.fasta	12,375.40	100.00%	6	24	83	0.42%	68.50%	RNVAEEAEQGEDPAMWENLDR	99.70%	71.7	28.1	77.2	2,459.09
ELF4-HFC phyb9 rep 3	| Symbols: ELF4 | Protein of unknown function (DUF1313) | chr2:16734545-16734880 REVERSE LENGTH=111	AT2G40080.1	TAIR10_20101214.fasta	12,375.40	100.00%	6	24	83	0.42%	68.50%	SLIQQVNDNHQSR	99.70%	74.2	30.4	0.56	1,539.75
ELF4-HFC phyb9 rep 3	| Symbols: ELF4 | Protein of unknown function (DUF1313) | chr2:16734545-16734880 REVERSE LENGTH=111	AT2G40080.1	TAIR10_20101214.fasta	12,375.40	100.00%	6	24	83	0.42%	68.50%	VVNMYSDLNTSFSSGFHGGK	99.70%	97.6	29.8	93.6	2,146.99
ELF4-HFC phyb9 rep 3	| Symbols: PCL1, LUX | Homeodomain-like superfamily protein | chr3:17183248-17184219 FORWARD LENGTH=323	AT3G46640.1,AT3G46640.2	TAIR10_20101214.fasta	34,648.50	100.00%	2	2	4	0.02%	15.20%	ENVASHLQK	95.90%	25.3	28	29.2	1,025.54
ELF4-HFC phyb9 rep 3	| Symbols: PCL1, LUX | Homeodomain-like superfamily protein | chr3:17183248-17184219 FORWARD LENGTH=323	AT3G46640.1,AT3G46640.2	TAIR10_20101214.fasta	34,648.50	100.00%	2	2	4	0.02%	15.20%	FVDVVAHLGIK	99.70%	28.2	25	12.1	1,197.70
ELF4-HFC phyb9 rep 3	| Symbols: PCL1, LUX | Homeodomain-like superfamily protein | chr3:17183248-17184219 FORWARD LENGTH=323	AT3G46640.1,AT3G46640.2	TAIR10_20101214.fasta	34,648.50	100.00%	2	2	4	0.02%	15.20%	GGSSGPNTSSSNNNVEEEDR	99.70%	99.5	25	67.3	2,036.84
ELF4-HFC phyb9 rep 3	| Symbols: PCL1, LUX | Homeodomain-like superfamily protein | chr3:17183248-17184219 FORWARD LENGTH=323	AT3G46640.1,AT3G46640.2	TAIR10_20101214.fasta	34,648.50	100.00%	2	2	4	0.02%	15.20%	LVWTPQLHK	95.70%	24.1	25.4	17.5	1,121.65
ELF4-HFC phyb9 rep 3	| Symbols: | C2 calcium/lipid-binding plant phosphoribosyltransferase family protein | chr5:2127200-2129584 REVERSE LENGTH=794	AT5G06850.1	TAIR10_20101214.fasta	91,004.50	100.00%	4	4	5	0.03%	8.19%	IQMVVGDIATQGER	99.70%	56.7	30.2	18.5	1,675.94
ELF4-HFC phyb9 rep 3	| Symbols: | C2 calcium/lipid-binding plant phosphoribosyltransferase family protein | chr5:2127200-2129584 REVERSE LENGTH=794	AT5G06850.1	TAIR10_20101214.fasta	91,004.50	100.00%	4	4	5	0.03%	8.19%	IYTHSYPLLVLQTK	99.70%	35.4	26.7	32.1	1,132.66
ELF4-HFC phyb9 rep 3	| Symbols: | C2 calcium/lipid-binding plant phosphoribosyltransferase family protein | chr5:2127200-2129584 REVERSE LENGTH=794	AT5G06850.1	TAIR10_20101214.fasta	91,004.50	100.00%	4	4	5	0.03%	8.19%	LISPLSVFEK	97.40%	24.6	26	45	1,570.77
ELF4-HFC phyb9 rep 3	| Symbols: | C2 calcium/lipid-binding plant phosphoribosyltransferase family protein | chr5:2127200-2129584 REVERSE LENGTH=794	AT5G06850.1	TAIR10_20101214.fasta	91,004.50	100.00%	4	4	5	0.03%	8.19%	VNVIEAQDVEPSDR	99.70%	64.4	29.8	0	1,525.78
ELF4-HFC phyb9 rep 3	| Symbols: | C2 calcium/lipid-binding plant phosphoribosyltransferase family protein | chr5:2127200-2129584 REVERSE LENGTH=794	AT5G06850.1	TAIR10_20101214.fasta	91,004.50	100.00%	4	4	5	0.03%	8.19%	VPPDSPLAPQWYR	99.70%	39.4	29.9	9.99	1,069.48
GFP-HFC Col rep 1	| Symbols: ELF4 | Protein of unknown function (DUF1313) | chr2:16734545-16734880 REVERSE LENGTH=111	AT2G40080.1	TAIR10_20101214.fasta	12,375.40	100.00%	2	6	7	0.04%	30.60%	NVALIQELNGNISK	99.70%	84.2	27.2	130	2,318.98
GFP-HFC Col rep 1	| Symbols: ELF4 | Protein of unknown function (DUF1313) | chr2:16734545-16734880 REVERSE LENGTH=111	AT2G40080.1	TAIR10_20101214.fasta	12,375.40	100.00%	2	6	7	0.04%	30.60%	VVNMYSDLNTSFSSGFHGGK	99.70%	81.3	30.1	104	1,512.84
GFP-HFC Col rep 1	| Symbols: PHYE | phytochrome E | chr4:10042312-10045948 REVERSE LENGTH=1112	AT4G18130.1	TAIR10_20101214.fasta	122,519.10	100.00%	2	4	6	0.03%	3.87%	SNTAQYSVDAALFADFAQSIYTGK	99.70%	101	31.9	17.2	944.516
GFP-HFC Col rep 1	| Symbols: PHYE | phytochrome E | chr4:10042312-10045948 REVERSE LENGTH=1112	AT4G18130.1	TAIR10_20101214.fasta	122,519.10	100.00%	2	4	6	0.03%	3.87%	VMVYQFHEDDHGEVVSEIR	99.70%	37.3	30.5	74.9	2,475.08
GFP-HFC Col rep 2	| Symbols: ELF4 | Protein of unknown function (DUF1313) | chr2:16734545-16734880 REVERSE LENGTH=111	AT2G40080.1	TAIR10_20101214.fasta	12,375.40	100.00%	3	8	23	0.11%	48.60%	NVAEEAEQGEDPAMWENLDR	99.70%	77.3	25.5	70.3	1,538.77
GFP-HFC Col rep 2	| Symbols: ELF4 | Protein of unknown function (DUF1313) | chr2:16734545-16734880 REVERSE LENGTH=111	AT2G40080.1	TAIR10_20101214.fasta	12,375.40	100.00%	3	8	23	0.11%	48.60%	NVALIQELNGNISK	99.70%	132	27.2	118	2,146.99
GFP-HFC Col rep 2	| Symbols: ELF4 | Protein of unknown function (DUF1313) | chr2:16734545-16734880 REVERSE LENGTH=111	AT2G40080.1	TAIR10_20101214.fasta	12,375.40	100.00%	3	8	23	0.11%	48.60%	VVNMYSDLNTSFSSGFHGGK	99.70%	99.4	29.4	37.3	3,198.43
GFP-HFC Col rep 2	| Symbols: | Ribosomal protein L6 family | chr4:6463201-6464458 REVERSE LENGTH=194	AT4G10450.1	TAIR10_20101214.fasta	21,974.50	100.00%	1	1	4	0.02%	18.00%	FVYAHFPINASIDGNNK	99.70%	39.5	31.3	117	2,318.98
GFP-HFC Col rep 2	| Symbols: | Ribosomal protein L6 family | chr4:6463201-6464458 REVERSE LENGTH=194	AT4G10450.1	TAIR10_20101214.fasta	21,974.50	100.00%	1	1	4	0.02%	18.00%	HLNLDFQLIK	95.30%	25.1	25.8	45	1,513.82
GFP-HFC Col rep 2	| Symbols: | Ribosomal protein L6 family | chr4:6463201-6464458 REVERSE LENGTH=194	AT4G10450.1	TAIR10_20101214.fasta	21,974.50	100.00%	1	1	4	0.02%	18.00%	VIEVEGPR	99.00%	45.8	25.9	11.1	944.516
GFP-HFC Col rep 3	| Symbols: ELF4 | Protein of unknown function (DUF1313) | chr2:16734545-16734880 REVERSE LENGTH=111	AT2G40080.1	TAIR10_20101214.fasta	12,375.40	100.00%	4	10	28	0.15%	49.50%	NVAEEAEQGEDPAMWENLDR	99.70%	89.1	26.6	65.3	2,475.08
GFP-HFC Col rep 3	| Symbols: ELF4 | Protein of unknown function (DUF1313) | chr2:16734545-16734880 REVERSE LENGTH=111	AT2G40080.1	TAIR10_20101214.fasta	12,375.40	100.00%	4	10	28	0.15%	49.50%	NVALIQELNGNISK	99.70%	107	27.2	67.1	1,538.77
GFP-HFC Col rep 3	| Symbols: ELF4 | Protein of unknown function (DUF1313) | chr2:16734545-16734880 REVERSE LENGTH=111	AT2G40080.1	TAIR10_20101214.fasta	12,375.40	100.00%	4	10	28	0.15%	49.50%	RNVAEEAEQGEDPAMWENLDR	99.10%	36.9	28.6	89.6	2,162.98
GFP-HFC Col rep 3	| Symbols: ELF4 | Protein of unknown function (DUF1313) | chr2:16734545-16734880 REVERSE LENGTH=111	AT2G40080.1	TAIR10_20101214.fasta	12,375.40	100.00%	4	10	28	0.15%	49.50%	VVNMYSDLNTSFSSGFHGGK	99.70%	100	29.7	23.9	3,198.43
GFP-HFC Col rep 3	| Symbols: | Ribosomal protein L6 family | chr4:6463201-6464458 REVERSE LENGTH=194	AT4G10450.1	TAIR10_20101214.fasta	21,974.50	100.00%	2	2	5	0.03%	27.30%	FLDGIYVSEK	99.70%	40.5	27.3	121	2,318.98
GFP-HFC Col rep 3	| Symbols: | Ribosomal protein L6 family | chr4:6463201-6464458 REVERSE LENGTH=194	AT4G10450.1	TAIR10_20101214.fasta	21,974.50	100.00%	2	2	5	0.03%	27.30%	FVYAHFPINASIDGNNK	99.70%	40.8	31.1	105	1,512.84
GFP-HFC Col rep 3	| Symbols: | Ribosomal protein L6 family | chr4:6463201-6464458 REVERSE LENGTH=194	AT4G10450.1	TAIR10_20101214.fasta	21,974.50	100.00%	2	2	5	0.03%	27.30%	KFLDGIYVSEK	99.60%	33.8	27.4	10.4	944.516
GFP-HFC Col rep 3	| Symbols: | Ribosomal protein L6 family | chr4:6463201-6464458 REVERSE LENGTH=194	AT4G10450.1	TAIR10_20101214.fasta	21,974.50	100.00%	2	2	5	0.03%	27.30%	VIEVEGPR	99.00%	31.2	25.9	73.6	2,475.08
GFP-HFC Col rep 3	| Symbols: | Ribosomal protein L6 family | chr4:6463201-6464458 REVERSE LENGTH=194	AT4G10450.1	TAIR10_20101214.fasta	21,974.50	100.00%	2	2	5	0.03%	27.30%	VKDEIILEGNDIELVSR	99.10%	27.2	28.6	0.98	1,539.75
GFP-HFC Col rep 4	| Symbols: | Granulin repeat cysteine protease family protein | chr3:6723024-6724768 FORWARD LENGTH=452	AT3G19390.1	TAIR10_20101214.fasta	49,308.20	100.00%	2	2	3	0.03%	7.08%	ALANQPISVAIEAGGR	99.70%	84.8	27.1	91.5	2,162.98
GFP-HFC Col rep 4	| Symbols: | Granulin repeat cysteine protease family protein | chr3:6723024-6724768 FORWARD LENGTH=452	AT3G19390.1	TAIR10_20101214.fasta	49,308.20	100.00%	2	2	3	0.03%	7.08%	VVTIDGYEDVPQNDEK	99.70%	48.3	30.1	10.9	3,199.41

Table S4. Peptide and Protein Identification for proteins that co-participated with ELF3::ELF3-HFC (all genotypes) and GFP-HFC controls as listed in Table 2.

Biological sample name	Protein name	Protein accession numbers	Database sources	Protein molecular weight (Da)	Protein identification probability	Exclusive unique peptide count	Exclusive unique spectrum count	Total spectrum count	Percentage of total spectra	Percentage sequence coverage	Peptide sequence	Best Peptide identification probability	Best Mascot Ion score	Best Mascot Identity score	Best Mascot Delta Ion score	Calculated +1H Peptide Mass (AMU)
ELF3-HFC Col rep1	| Symbols: | Homeodomain-like superfamily protein | chr5:24004047-24004943 FORWARD LENGTH=298	AT5G59570.1	TAIR10_20101214.fasta	32,366.70	99.90%	1	1	6	0.07%	17.40%	ASQITLSSLR	99.70%	61.4	28.1	45.3	1,075.61
ELF3-HFC Col rep1	| Symbols: | Homeodomain-like superfamily protein | chr5:24004047-24004943 FORWARD LENGTH=298	AT5G59570.1	TAIR10_20101214.fasta	32,366.70	99.90%	1	1	6	0.07%	17.40%	ENVASHLQK	99.70%	44.8	27.8	28.8	1,025.54
ELF3-HFC Col rep1	| Symbols: | Homeodomain-like superfamily protein | chr5:24004047-24004943 FORWARD LENGTH=298	AT5G59570.1	TAIR10_20101214.fasta	32,366.70	99.90%	1	1	6	0.07%	17.40%	FVDVVAHLGIK	99.70%	47	25	39.3	1,197.70
ELF3-HFC Col rep1	| Symbols: | Homeodomain-like superfamily protein | chr5:24004047-24004943 FORWARD LENGTH=298	AT5G59570.1	TAIR10_20101214.fasta	32,366.70	99.90%	1	1	6	0.07%	17.40%	LVWTPQLHK	99.70%	35.6	25.4	31.5	1,121.65
ELF3-HFC Col rep1	| Symbols: | Homeodomain-like superfamily protein | chr5:24004047-24004943 FORWARD LENGTH=298	AT5G59570.1	TAIR10_20101214.fasta	32,366.70	99.90%	1	1	6	0.07%	17.40%	TIMQLMNVEGLTR	99.70%	47.1	30.6	36.6	1,505.78
ELF3-HFC Col rep1	| Symbols: | C2 calcium/lipid-binding plant phosphoribosyltransferase family protein | chr5:2127200-2129584 REVERSE LENGTH=794	AT5G06850.1	TAIR10_20101214.fasta	91,004.50	100.00%	2	2	2	0.02%	3.40%	DKVQSSTVEVFVR	96.80%	23.4	29.3	13.6	1,493.80
ELF3-HFC Col rep1	| Symbols: | C2 calcium/lipid-binding plant phosphoribosyltransferase family protein | chr5:2127200-2129584 REVERSE LENGTH=794	AT5G06850.1	TAIR10_20101214.fasta	91,004.50	100.00%	2	2	2	0.02%	3.40%	VNVIEAQDVEPSDR	99.70%	53	29.7	43	1,570.77
ELF3-HFC Col rep1	| Symbols: | F-box family protein | chr2:7074940-7079181 FORWARD LENGTH=727	AT2G16365.4	TAIR10_20101214.fasta	82,716.20	100.00%	15	20	26	0.30%	27.40%	ALSGLNPYGLR	99.70%	54.2	29.9	40.5	1,160.64
ELF3-HFC Col rep1	| Symbols: | F-box family protein | chr2:7074940-7079181 FORWARD LENGTH=727	AT2G16365.4	TAIR10_20101214.fasta	82,716.20	100.00%	15	20	26	0.30%	27.40%	EASLGEKENNNLFLEILK	99.70%	58	30.5	46.6	2,061.09
ELF3-HFC Col rep1	| Symbols: | F-box family protein | chr2:7074940-7079181 FORWARD LENGTH=727	AT2G16365.4	TAIR10_20101214.fasta	82,716.20	100.00%	15	20	26	0.30%	27.40%	ENNNLFLEILK	99.70%	75.8	30.4	15.9	1,347.72
ELF3-HFC Col rep1	| Symbols: | F-box family protein | chr2:7074940-7079181 FORWARD LENGTH=727	AT2G16365.4	TAIR10_20101214.fasta	82,716.20	100.00%	15	20	26	0.30%	27.40%	ETTTTTTTKPSFHEDVGSSSR	99.70%	48.8	30.5	34.5	2,269.06
ELF3-HFC Col rep1	| Symbols: | F-box family protein | chr2:7074940-7079181 FORWARD LENGTH=727	AT2G16365.4	TAIR10_20101214.fasta	82,716.20	100.00%	15	20	26	0.30%	27.40%	IFGGYLDLFPNQDHSHNR	99.70%	71.8	30.9	0	2,131.00
ELF3-HFC Col rep1	| Symbols: | F-box family protein | chr2:7074940-7079181 FORWARD LENGTH=727	AT2G16365.4	TAIR10_20101214.fasta	82,716.20	100.00%	15	20	26	0.30%	27.40%	IYATVDSVNLPK	99.70%	93.6	29.5	73.4	1,319.72
ELF3-HFC Col rep1	| Symbols: | F-box family protein | chr2:7074940-7079181 FORWARD LENGTH=727	AT2G16365.4	TAIR10_20101214.fasta	82,716.20	100.00%	15	20	26	0.30%	27.40%	IYATVDSVNLPKEDSHQLLK	99.70%	37.6	29.9	23.2	2,270.20
ELF3-HFC Col rep1	| Symbols: | F-box family protein | chr2:7074940-7079181 FORWARD LENGTH=727	AT2G16365.4	TAIR10_20101214.fasta	82,716.20	100.00%	15	20	26	0.30%	27.40%	LDFPIQEK	99.00%	40.8	30.2	12.9	989.5304
ELF3-HFC Col rep1	| Symbols: | F-box family protein | chr2:7074940-7079181 FORWARD LENGTH=727	AT2G16365.4	TAIR10_20101214.fasta	82,716.20	100.00%	15	20	26	0.30%	27.40%	LQTIHLSGSISSSSTK	99.70%	45	30.4	31.4	1,645.88
ELF3-HFC Col rep1	| Symbols: | F-box family protein | chr2:7074940-7079181 FORWARD LENGTH=727	AT2G16365.4	TAIR10_20101214.fasta	82,716.20	100.00%	15	20	26	0.30%	27.40%	NSSVNPYESAWLGR	99.70%	87.7	30.3	72.7	1,579.75
ELF3-HFC Col rep1	| Symbols: | F-box family protein | chr2:7074940-7079181 FORWARD LENGTH=727	AT2G16365.4	TAIR10_20101214.fasta	82,716.20	100.00%	15	20	26	0.30%	27.40%	QGGDDITLLHPWIQR	99.50%	31.4	30.6	18.4	1,748.91
ELF3-HFC Col rep1	| Symbols: | F-box family protein | chr2:7074940-7079181 FORWARD LENGTH=727	AT2G16365.4	TAIR10_20101214.fasta	82,716.20	100.00%	15	20	26	0.30%	27.40%	SGINNLQPR	99.70%	34	25.7	21.9	998.5378
ELF3-HFC Col rep1	| Symbols: | F-box family protein | chr2:7074940-7079181 FORWARD LENGTH=727	AT2G16365.4	TAIR10_20101214.fasta	82,716.20	100.00%	15	20	26	0.30%	27.40%	TTQNLLELIRPVR	99.70%	63.1	25	50.1	1,552.92
ELF3-HFC Col rep1	| Symbols: | F-box family protein | chr2:7074940-7079181 FORWARD LENGTH=727	AT2G16365.4	TAIR10_20101214.fasta	82,716.20	100.00%	15	20	26	0.30%	27.40%	TTSTDQPTGQEASFEPESHKEFEK	99.10%	28.3	29	20.5	2,710.21
ELF3-HFC Col rep1	| Symbols: | F-box family protein | chr2:7074940-7079181 FORWARD LENGTH=727	AT2G16365.4	TAIR10_20101214.fasta	82,716.20	100.00%	15	20	26	0.30%	27.40%	VRLEPEVEASSR	99.70%	60.9	29.1	52.2	1,371.72
ELF3-HFC Col rep1	| Symbols: | Protein kinase family protein | chr2:10985118-10988652 REVERSE LENGTH=673	AT2G25760.1,AT2G25760.2	TAIR10_20101214.fasta	76,182.30	100.00%	3	4	5	0.06%	10.10%	GYVHGDVKPENFLLGPPGTPEEK	98.30%	25.4	31.3	17.7	2,480.25
ELF3-HFC Col rep1	| Symbols: | Protein kinase family protein | chr2:10985118-10988652 REVERSE LENGTH=673	AT2G25760.1,AT2G25760.2	TAIR10_20101214.fasta	76,182.30	100.00%	3	4	5	0.06%	10.10%	KLFLVDLGLASK	99.70%	66.8	25	62	1,303.80
ELF3-HFC Col rep1	| Symbols: | Protein kinase family protein | chr2:10985118-10988652 REVERSE LENGTH=673	AT2G25760.1,AT2G25760.2	TAIR10_20101214.fasta	76,182.30	100.00%	3	4	5	0.06%	10.10%	VSDSFPFK	95.50%	22.9	26.3	16.1	926.4618
ELF3-HFC Col rep1	| Symbols: | Protein kinase family protein | chr2:10985118-10988652 REVERSE LENGTH=673	AT2G25760.1,AT2G25760.2	TAIR10_20101214.fasta	76,182.30	100.00%	3	4	5	0.06%	10.10%	YVSLFDGIVGPNPDIRPINTEGAQK	99.70%	32.6	30	22.9	2,700.40
ELF3-HFC Col rep1	| Symbols: | Protein kinase family protein | chr3:1014412-1018244 REVERSE LENGTH=701	AT3G03940.1	TAIR10_20101214.fasta	77,995.40	99.90%	1	1	6	0.07%	9.27%	GRLPWQGYQGDNK	99.70%	56.5	30.1	44.5	1,518.75
ELF3-HFC Col rep1	| Symbols: | Protein kinase family protein | chr3:1014412-1018244 REVERSE LENGTH=701	AT3G03940.1	TAIR10_20101214.fasta	77,995.40	99.90%	1	1	6	0.07%	9.27%	LLINLEEDEQPR	99.70%	61	29.4	50.9	1,468.76
ELF3-HFC Col rep1	| Symbols: | Protein kinase family protein | chr3:1014412-1018244 REVERSE LENGTH=701	AT3G03940.1	TAIR10_20101214.fasta	77,995.40	99.90%	1	1	6	0.07%	9.27%	NSGYSEQVVELDFLYPSEGIHR	99.70%	72.6	31.5	55.2	2,539.21
ELF3-HFC Col rep1	| Symbols: | Protein kinase family protein | chr3:1014412-1018244 REVERSE LENGTH=701	AT3G03940.1	TAIR10_20101214.fasta	77,995.40	99.90%	1	1	6	0.07%	9.27%	VQVGNSPVYK	99.70%	45.7	30.4	34.2	1,090.59
ELF3-HFC Col rep1	| Symbols: | Protein kinase family protein | chr3:1014412-1018244 REVERSE LENGTH=701	AT3G03940.1	TAIR10_20101214.fasta	77,995.40	99.90%	1	1	6	0.07%	9.27%	VSDSFPFK	95.50%	22.9	26.3	16.1	926.4618
ELF3-HFC Col rep1	| Symbols: | Protein kinase family protein | chr3:4469434-4473234 FORWARD LENGTH=703	AT3G13670.1	TAIR10_20101214.fasta	78,640.00	100.00%	7	8	13	0.15%	21.60%	GGFGQVFVGR	99.70%	40.7	28.8	22	1,023.54
ELF3-HFC Col rep1	| Symbols: | Protein kinase family protein | chr3:4469434-4473234 FORWARD LENGTH=703	AT3G13670.1	TAIR10_20101214.fasta	78,640.00	100.00%	7	8	13	0.15%	21.60%	GRLPWQGYQGDNK	99.70%	56.5	30.1	44.5	1,518.75
ELF3-HFC Col rep1	| Symbols: | Protein kinase family protein | chr3:4469434-4473234 FORWARD LENGTH=703	AT3G13670.1	TAIR10_20101214.fasta	78,640.00	100.00%	7	8	13	0.15%	21.60%	ITSTAATTDQAALILSIPR	99.70%	38.6	27	30.5	1,944.07
ELF3-HFC Col rep1	| Symbols: | Protein kinase family protein | chr3:4469434-4473234 FORWARD LENGTH=703	AT3G13670.1	TAIR10_20101214.fasta	78,640.00	100.00%	7	8	13	0.15%	21.60%	KLFLVDLGLATK	99.70%	75.6	25	70.2	1,317.81
ELF3-HFC Col rep1	| Symbols: | Protein kinase family protein | chr3:4469434-4473234 FORWARD LENGTH=703	AT3G13670.1	TAIR10_20101214.fasta	78,640.00	100.00%	7	8	13	0.15%	21.60%	LFLVDLGLATK	99.70%	77.8	25	54.3	1,189.72
ELF3-HFC Col rep1	| Symbols: | Protein kinase family protein | chr3:4469434-4473234 FORWARD LENGTH=703	AT3G13670.1	TAIR10_20101214.fasta	78,640.00	100.00%	7	8	13	0.15%	21.60%	LGVPATQWISIYNAR	99.70%	62.7	29.7	56.4	1,688.91
ELF3-HFC Col rep1	| Symbols: | Protein kinase family protein | chr3:4469434-4473234 FORWARD LENGTH=703	AT3G13670.1	TAIR10_20101214.fasta	78,640.00	100.00%	7	8	13	0.15%	21.60%	LSIGEEEEDAPR	99.70%	84.7	27.9	80.4	1,344.63
ELF3-HFC Col rep1	| Symbols: | Protein kinase family protein | chr3:4469434-4473234 FORWARD LENGTH=703	AT3G13670.1	TAIR10_20101214.fasta	78,640.00	100.00%	7	8	13	0.15%	21.60%	LVSLFQDLLGENPAIRPINTEGAQK	99.70%	49	27.2	43.6	2,723.47
ELF3-HFC Col rep1	| Symbols: | Protein kinase family protein | chr3:4469434-4473234 FORWARD LENGTH=703	AT3G13670.1	TAIR10_20101214.fasta	78,640.00	100.00%	7	8	13	0.15%	21.60%	NSGYSEQVVELDFLYPSEGIHR	99.70%	72.6	31.5	55.2	2,539.21
ELF3-HFC Col rep1	| Symbols: | Protein kinase family protein | chr3:4469434-4473234 FORWARD LENGTH=703	AT3G13670.1	TAIR10_20101214.fasta	78,640.00	100.00%	7	8	13	0.15%	21.60%	RDDLESLAYTLIFLHR	99.70%	38.2	29.9	27.8	1,962.04
ELF3-HFC Col rep1	| Symbols: | Protein kinase family protein | chr3:4469434-4473234 FORWARD LENGTH=703	AT3G13670.1	TAIR10_20101214.fasta	78,640.00	100.00%	7	8	13	0.15%	21.60%	VSDSFPFK	95.50%	22.9	26.3	16.1	926.4618
ELF3-HFC Col rep1	| Symbols: | Protein kinase family protein | chr5:6010215-6013724 REVERSE LENGTH=691	AT5G18190.1	TAIR10_20101214.fasta	76,985.50	99.90%	1	1	7	0.08%	7.67%	GGFGQVFVGR	99.70%	40.7	28.8	22	1,023.54
ELF3-HFC Col rep1	| Symbols: | Protein kinase family protein | chr5:6010215-6013724 REVERSE LENGTH=691	AT5G18190.1	TAIR10_20101214.fasta	76,985.50	99.90%	1	1	7	0.08%	7.67%	GRLPWQGYQGDNK	99.70%	56.5	30.1	44.5	1,518.75
ELF3-HFC Col rep1	| Symbols: | Protein kinase family protein | chr5:6010215-6013724 REVERSE LENGTH=691	AT5G18190.1	TAIR10_20101214.fasta	76,985.50	99.90%	1	1	7	0.08%	7.67%	KLYLIDLGLASK	99.70%	47.6	25	44.5	1,333.81
ELF3-HFC Col rep1	| Symbols: | Protein kinase family protein | chr5:6010215-6013724 REVERSE LENGTH=691	AT5G18190.1	TAIR10_20101214.fasta	76,985.50	99.90%	1	1	7	0.08%	7.67%	VQVGNSPVYK	99.70%	45.7	30.4	34.2	1,090.59
ELF3-HFC Col rep1	| Symbols: | Protein kinase family protein | chr5:6010215-6013724 REVERSE LENGTH=691	AT5G18190.1	TAIR10_20101214.fasta	76,985.50	99.90%	1	1	7	0.08%	7.67%	VSDSFPFK	95.50%	22.9	26.3	16.1	926.4618
ELF3-HFC Col rep1	| Symbols: ATAN11, LWD1 | Transducin/WD40 repeat-like superfamily protein | chr1:4395114-4396154 REVERSE LENGTH=346	AT1G12910.1	TAIR10_20101214.fasta	39,089.30	100.00%	1	1	6	0.07%	12.70%	DKEHSTIIYESSEPDTPLVR	99.70%	53.8	31.8	45.4	2,316.14
ELF3-HFC Col rep1	| Symbols: ATAN11, LWD1 | Transducin/WD40 repeat-like superfamily protein | chr1:4395114-4396154 REVERSE LENGTH=346	AT1G12910.1	TAIR10_20101214.fasta	39,089.30	100.00%	1	1	6	0.07%	12.70%	FPALPVVELQR	99.70%	68.6	25	63.1	1,268.74
ELF3-HFC Col rep1	| Symbols: ATAN11, LWD1 | Transducin/WD40 repeat-like superfamily protein | chr1:4395114-4396154 REVERSE LENGTH=346	AT1G12910.1	TAIR10_20101214.fasta	39,089.30	100.00%	1	1	6	0.07%	12.70%	LAITSLLEQYPNR	99.70%	59.3	28.9	0	1,517.83
ELF3-HFC Col rep1	| Symbols: COP1, ATCOP1, DET340, FUS1, EMB168 | Transducin/WD40 repeat-like superfamily protein | chr2:13978000-13983282 FORWARD LENGTH=675	AT2G32950.1	TAIR10_20101214.fasta	76,188.00	100.00%	10	12	13	0.15%	17.90%	AQGSSHGLPK	99.70%	55.8	28	43.9	981.5113
ELF3-HFC Col rep1	| Symbols: COP1, ATCOP1, DET340, FUS1, EMB168 | Transducin/WD40 repeat-like superfamily protein | chr2:13978000-13983282 FORWARD LENGTH=675	AT2G32950.1	TAIR10_20101214.fasta	76,188.00	100.00%	10	12	13	0.15%	17.90%	EITRPVTSHR	98.20%	29.2	27.1	15.2	1,195.65
ELF3-HFC Col rep1	| Symbols: COP1, ATCOP1, DET340, FUS1, EMB168 | Transducin/WD40 repeat-like superfamily protein | chr2:13978000-13983282 FORWARD LENGTH=675	AT2G32950.1	TAIR10_20101214.fasta	76,188.00	100.00%	10	12	13	0.15%	17.90%	EVDNLLTLLAER	99.70%	73.6	28.2	57.8	1,385.76
ELF3-HFC Col rep1	| Symbols: COP1, ATCOP1, DET340, FUS1, EMB168 | Transducin/WD40 repeat-like superfamily protein | chr2:13978000-13983282 FORWARD LENGTH=675	AT2G32950.1	TAIR10_20101214.fasta	76,188.00	100.00%	10	12	13	0.15%	17.90%	GGNFVGNYQNK	99.70%	32	28.8	20.9	1,197.56
ELF3-HFC Col rep1	| Symbols: COP1, ATCOP1, DET340, FUS1, EMB168 | Transducin/WD40 repeat-like superfamily protein | chr2:13978000-13983282 FORWARD LENGTH=675	AT2G32950.1	TAIR10_20101214.fasta	76,188.00	100.00%	10	12	13	0.15%	17.90%	LWDVKDNLPVR	99.70%	35.8	26.1	34.9	1,354.75
ELF3-HFC Col rep1	| Symbols: COP1, ATCOP1, DET340, FUS1, EMB168 | Transducin/WD40 repeat-like superfamily protein | chr2:13978000-13983282 FORWARD LENGTH=675	AT2G32950.1	TAIR10_20101214.fasta	76,188.00	100.00%	10	12	13	0.15%	17.90%	NHIASSDYEGIVTVWDVTTR	99.60%	27.8	31.3	20.9	2,263.10
ELF3-HFC Col rep1	| Symbols: COP1, ATCOP1, DET340, FUS1, EMB168 | Transducin/WD40 repeat-like superfamily protein | chr2:13978000-13983282 FORWARD LENGTH=675	AT2G32950.1	TAIR10_20101214.fasta	76,188.00	100.00%	10	12	13	0.15%	17.90%	NQIGFNSNSLSIR	99.70%	86.9	30.6	66.9	1,449.74
ELF3-HFC Col rep1	| Symbols: COP1, ATCOP1, DET340, FUS1, EMB168 | Transducin/WD40 repeat-like superfamily protein | chr2:13978000-13983282 FORWARD LENGTH=675	AT2G32950.1	TAIR10_20101214.fasta	76,188.00	100.00%	10	12	13	0.15%	17.90%	QKVDELNEVQTDLQYIKEDINAVER	99.60%	29.3	31.5	22	2,989.51
ELF3-HFC Col rep1	| Symbols: COP1, ATCOP1, DET340, FUS1, EMB168 | Transducin/WD40 repeat-like superfamily protein | chr2:13978000-13983282 FORWARD LENGTH=675	AT2G32950.1	TAIR10_20101214.fasta	76,188.00	100.00%	10	12	13	0.15%	17.90%	TASPLDQFR	99.70%	36.4	30.6	21.3	1,034.53
ELF3-HFC Col rep1	| Symbols: COP1, ATCOP1, DET340, FUS1, EMB168 | Transducin/WD40 repeat-like superfamily protein | chr2:13978000-13983282 FORWARD LENGTH=675	AT2G32950.1	TAIR10_20101214.fasta	76,188.00	100.00%	10	12	13	0.15%	17.90%	VDELNEVQTDLQYIK	99.70%	82.6	31.2	68.5	1,806.91
ELF3-HFC Col rep1	| Symbols: ELF3, PYK20 | hydroxyproline-rich glycoprotein family protein | chr2:11059459-11063178 FORWARD LENGTH=695	AT2G25930.1	TAIR10_20101214.fasta	77,204.80	100.00%	23	34	66	0.76%	38.10%	EEEDFAVPVYINSR	99.70%	59.8	30.2	43.6	1,667.79
ELF3-HFC Col rep1	| Symbols: ELF3, PYK20 | hydroxyproline-rich glycoprotein family protein | chr2:11059459-11063178 FORWARD LENGTH=695	AT2G25930.1	TAIR10_20101214.fasta	77,204.80	100.00%	23	34	66	0.76%	38.10%	FQEVNQTGSK	99.70%	67.4	29.7	42.3	1,137.55
ELF3-HFC Col rep1	| Symbols: ELF3, PYK20 | hydroxyproline-rich glycoprotein family protein | chr2:11059459-11063178 FORWARD LENGTH=695	AT2G25930.1	TAIR10_20101214.fasta	77,204.80	100.00%	23	34	66	0.76%	38.10%	FVSQMSFMENVR	99.70%	71.3	27.3	59.2	1,506.67
ELF3-HFC Col rep1	| Symbols: ELF3, PYK20 | hydroxyproline-rich glycoprotein family protein | chr2:11059459-11063178 FORWARD LENGTH=695	AT2G25930.1	TAIR10_20101214.fasta	77,204.80	100.00%	23	34	66	0.76%	38.10%	HTPMVAPSSHHSIR	99.70%	59.2	29.2	40.6	1,572.77
ELF3-HFC Col rep1	| Symbols: ELF3, PYK20 | hydroxyproline-rich glycoprotein family protein | chr2:11059459-11063178 FORWARD LENGTH=695	AT2G25930.1	TAIR10_20101214.fasta	77,204.80	100.00%	23	34	66	0.76%	38.10%	IFQSIQEER	99.70%	62.1	30.1	27.6	1,149.59
ELF3-HFC Col rep1	| Symbols: ELF3, PYK20 | hydroxyproline-rich glycoprotein family protein | chr2:11059459-11063178 FORWARD LENGTH=695	AT2G25930.1	TAIR10_20101214.fasta	77,204.80	100.00%	23	34	66	0.76%	38.10%	ILEPMFPR	99.00%	59.6	30.3	25.8	1,018.54
ELF3-HFC Col rep1	| Symbols: ELF3, PYK20 | hydroxyproline-rich glycoprotein family protein | chr2:11059459-11063178 FORWARD LENGTH=695	AT2G25930.1	TAIR10_20101214.fasta	77,204.80	100.00%	23	34	66	0.76%	38.10%	KLLPSEFLVKPPLPHVVVK	99.70%	42.2	25	41.6	2,140.33
ELF3-HFC Col rep1	| Symbols: ELF3, PYK20 | hydroxyproline-rich glycoprotein family protein | chr2:11059459-11063178 FORWARD LENGTH=695	AT2G25930.1	TAIR10_20101214.fasta	77,204.80	100.00%	23	34	66	0.76%	38.10%	LASENAAR	99.00%	37.3	29.1	25.7	831.4318
ELF3-HFC Col rep1	| Symbols: ELF3, PYK20 | hydroxyproline-rich glycoprotein family protein | chr2:11059459-11063178 FORWARD LENGTH=695	AT2G25930.1	TAIR10_20101214.fasta	77,204.80	100.00%	23	34	66	0.76%	38.10%	LHVNDADK	99.00%	51.1	27.4	36.4	911.4582
ELF3-HFC Col rep1	| Symbols: ELF3, PYK20 | hydroxyproline-rich glycoprotein family protein | chr2:11059459-11063178 FORWARD LENGTH=695	AT2G25930.1	TAIR10_20101214.fasta	77,204.80	100.00%	23	34	66	0.76%	38.10%	LHVNDADKGGPR	99.70%	80.6	30.3	73.7	1,278.66
ELF3-HFC Col rep1	| Symbols: ELF3, PYK20 | hydroxyproline-rich glycoprotein family protein | chr2:11059459-11063178 FORWARD LENGTH=695	AT2G25930.1	TAIR10_20101214.fasta	77,204.80	100.00%	23	34	66	0.76%	38.10%	LIAASPDLLLDEISFLGK	99.70%	138	25.8	122	1,915.08
ELF3-HFC Col rep1	| Symbols: ELF3, PYK20 | hydroxyproline-rich glycoprotein family protein | chr2:11059459-11063178 FORWARD LENGTH=695	AT2G25930.1	TAIR10_20101214.fasta	77,204.80	100.00%	23	34	66	0.76%	38.10%	LLPSEFLVKPPLPHVVVK	99.70%	48.6	25	44.9	2,012.23
ELF3-HFC Col rep1	| Symbols: ELF3, PYK20 | hydroxyproline-rich glycoprotein family protein | chr2:11059459-11063178 FORWARD LENGTH=695	AT2G25930.1	TAIR10_20101214.fasta	77,204.80	100.00%	23	34	66	0.76%	38.10%	MALYEQLSIPSQR	99.70%	42.5	31.3	30.8	1,535.79
ELF3-HFC Col rep1	| Symbols: ELF3, PYK20 | hydroxyproline-rich glycoprotein family protein | chr2:11059459-11063178 FORWARD LENGTH=695	AT2G25930.1	TAIR10_20101214.fasta	77,204.80	100.00%	23	34	66	0.76%	38.10%	MESSAENVVGR	99.70%	68.2	28.3	60.7	1,178.55
ELF3-HFC Col rep1	| Symbols: ELF3, PYK20 | hydroxyproline-rich glycoprotein family protein | chr2:11059459-11063178 FORWARD LENGTH=695	AT2G25930.1	TAIR10_20101214.fasta	77,204.80	100.00%	23	34	66	0.76%	38.10%	MVREEEDFAVPVYINSR	98.60%	28	31.5	14.7	2,054.00
ELF3-HFC Col rep1	| Symbols: ELF3, PYK20 | hydroxyproline-rich glycoprotein family protein | chr2:11059459-11063178 FORWARD LENGTH=695	AT2G25930.1	TAIR10_20101214.fasta	77,204.80	100.00%	23	34	66	0.76%	38.10%	NLSVQHLDSSAANQATEK	99.70%	56.5	30.8	48.7	1,912.94
ELF3-HFC Col rep1	| Symbols: ELF3, PYK20 | hydroxyproline-rich glycoprotein family protein | chr2:11059459-11063178 FORWARD LENGTH=695	AT2G25930.1	TAIR10_20101214.fasta	77,204.80	100.00%	23	34	66	0.76%	38.10%	QGSTGSSPSGPQGISGSK	99.70%	93.5	29	79.1	1,618.77
ELF3-HFC Col rep1	| Symbols: ELF3, PYK20 | hydroxyproline-rich glycoprotein family protein | chr2:11059459-11063178 FORWARD LENGTH=695	AT2G25930.1	TAIR10_20101214.fasta	77,204.80	100.00%	23	34	66	0.76%	38.10%	QNVCLATCSKPEVR	99.70%	43	30.3	35.7	1,661.81
ELF3-HFC Col rep1	| Symbols: ELF3, PYK20 | hydroxyproline-rich glycoprotein family protein | chr2:11059459-11063178 FORWARD LENGTH=695	AT2G25930.1	TAIR10_20101214.fasta	77,204.80	100.00%	23	34	66	0.76%	38.10%	SDNEPAPQQQQQPTK	99.70%	28.1	29.7	23.5	1,695.79
ELF3-HFC Col rep1	| Symbols: ELF3, PYK20 | hydroxyproline-rich glycoprotein family protein | chr2:11059459-11063178 FORWARD LENGTH=695	AT2G25930.1	TAIR10_20101214.fasta	77,204.80	100.00%	23	34	66	0.76%	38.10%	SNNTSTLVHPGPSSQPCGVER	99.70%	42.4	30.1	1.27	2,225.03
ELF3-HFC Col rep1	| Symbols: ELF3, PYK20 | hydroxyproline-rich glycoprotein family protein | chr2:11059459-11063178 FORWARD LENGTH=695	AT2G25930.1	TAIR10_20101214.fasta	77,204.80	100.00%	23	34	66	0.76%	38.10%	TTVTQTTR	99.00%	41.8	28.4	32.5	907.4844
ELF3-HFC Col rep1	| Symbols: ELF3, PYK20 | hydroxyproline-rich glycoprotein family protein | chr2:11059459-11063178 FORWARD LENGTH=695	AT2G25930.1	TAIR10_20101214.fasta	77,204.80	100.00%	23	34	66	0.76%	38.10%	VFAVQLFELHR	99.70%	78.8	28.4	58.1	1,358.76
ELF3-HFC Col rep1	| Symbols: ELF3, PYK20 | hydroxyproline-rich glycoprotein family protein | chr2:11059459-11063178 FORWARD LENGTH=695	AT2G25930.1	TAIR10_20101214.fasta	77,204.80	100.00%	23	34	66	0.76%	38.10%	VNDYNASLR	99.70%	72.3	29.1	53.8	1,051.52
ELF3-HFC Col rep1	| Symbols: ELF4 | Protein of unknown function (DUF1313) | chr2:16734545-16734880 REVERSE LENGTH=111	AT2G40080.1	TAIR10_20101214.fasta	12,375.40	100.00%	4	8	22	0.26%	61.30%	NVALIQELNGNISK	99.70%	116	28.4	38.4	1,513.82
ELF3-HFC Col rep1	| Symbols: ELF4 | Protein of unknown function (DUF1313) | chr2:16734545-16734880 REVERSE LENGTH=111	AT2G40080.1	TAIR10_20101214.fasta	12,375.40	100.00%	4	8	22	0.26%	61.30%	RNVAEEAEQGEDPAMWENLDR	99.70%	66.4	28.3	1.43	2,476.07
ELF3-HFC Col rep1	| Symbols: ELF4 | Protein of unknown function (DUF1313) | chr2:16734545-16734880 REVERSE LENGTH=111	AT2G40080.1	TAIR10_20101214.fasta	12,375.40	100.00%	4	8	22	0.26%	61.30%	SLIQQVNDNHQSR	99.70%	97.4	30.3	79.4	1,538.77
ELF3-HFC Col rep1	| Symbols: ELF4 | Protein of unknown function (DUF1313) | chr2:16734545-16734880 REVERSE LENGTH=111	AT2G40080.1	TAIR10_20101214.fasta	12,375.40	100.00%	4	8	22	0.26%	61.30%	VVNMYSDLNTSFSSGFHGGK	99.70%	67.9	28.4	21	2,163.97
ELF3-HFC Col rep1	| Symbols: PCL1, LUX | Homeodomain-like superfamily protein | chr3:17183248-17184219 FORWARD LENGTH=323	AT3G46640.1,AT3G46640.2	TAIR10_20101214.fasta	34,648.50	100.00%	2	2	9	0.10%	20.40%	ASETTLSSLR	99.70%	74.5	28.9	55	1,064.56
ELF3-HFC Col rep1	| Symbols: PCL1, LUX | Homeodomain-like superfamily protein | chr3:17183248-17184219 FORWARD LENGTH=323	AT3G46640.1,AT3G46640.2	TAIR10_20101214.fasta	34,648.50	100.00%	2	2	9	0.10%	20.40%	ENVASHLQK	99.70%	44.8	27.8	28.8	1,025.54
ELF3-HFC Col rep1	| Symbols: PCL1, LUX | Homeodomain-like superfamily protein | chr3:17183248-17184219 FORWARD LENGTH=323	AT3G46640.1,AT3G46640.2	TAIR10_20101214.fasta	34,648.50	100.00%	2	2	9	0.10%	20.40%	FVDVVAHLGIK	99.70%	47	25	39.3	1,197.70
ELF3-HFC Col rep1	| Symbols: PCL1, LUX | Homeodomain-like superfamily protein | chr3:17183248-17184219 FORWARD LENGTH=323	AT3G46640.1,AT3G46640.2	TAIR10_20101214.fasta	34,648.50	100.00%	2	2	9	0.10%	20.40%	LVWTPQLHK	99.70%	35.6	25.4	31.5	1,121.65
ELF3-HFC Col rep1	| Symbols: PCL1, LUX | Homeodomain-like superfamily protein | chr3:17183248-17184219 FORWARD LENGTH=323	AT3G46640.1,AT3G46640.2	TAIR10_20101214.fasta	34,648.50	100.00%	2	2	9	0.10%	20.40%	MQGLTNEGPSASDK	99.70%	76.6	27.9	65.9	1,434.65
ELF3-HFC Col rep1	| Symbols: PCL1, LUX | Homeodomain-like superfamily protein | chr3:17183248-17184219 FORWARD LENGTH=323	AT3G46640.1,AT3G46640.2	TAIR10_20101214.fasta	34,648.50	100.00%	2	2	9	0.10%	20.40%	TIMQLMNVEGLTR	99.70%	47.1	30.6	36.6	1,505.78
ELF3-HFC Col rep1	| Symbols: PHYA, FHY2, FRE1, HY8 | phytochrome A | chr1:3095498-3099216 REVERSE LENGTH=1122	AT1G09570.1	TAIR10_20101214.fasta	124,503.20	100.00%	15	16	19	0.22%	19.40%	ADAGPISLVVNACASR	99.70%	46.2	29.5	39.6	1,600.81
ELF3-HFC Col rep1	| Symbols: PHYA, FHY2, FRE1, HY8 | phytochrome A | chr1:3095498-3099216 REVERSE LENGTH=1122	AT1G09570.1	TAIR10_20101214.fasta	124,503.20	100.00%	15	16	19	0.22%	19.40%	ALGFGDVSLLNPILVHCR	99.70%	55.6	28.1	52.5	1,981.07
ELF3-HFC Col rep1	| Symbols: PHYA, FHY2, FRE1, HY8 | phytochrome A | chr1:3095498-3099216 REVERSE LENGTH=1122	AT1G09570.1	TAIR10_20101214.fasta	124,503.20	100.00%	15	16	19	0.22%	19.40%	DAPLGIVSQSPNIMDLVK	99.70%	58.9	29.8	30.7	1,913.01
ELF3-HFC Col rep1	| Symbols: PHYA, FHY2, FRE1, HY8 | phytochrome A | chr1:3095498-3099216 REVERSE LENGTH=1122	AT1G09570.1	TAIR10_20101214.fasta	124,503.20	100.00%	15	16	19	0.22%	19.40%	IAELTGLSVDEAIGK	99.70%	77.9	29	64.7	1,515.83
ELF3-HFC Col rep1	| Symbols: PHYA, FHY2, FRE1, HY8 | phytochrome A | chr1:3095498-3099216 REVERSE LENGTH=1122	AT1G09570.1	TAIR10_20101214.fasta	124,503.20	100.00%	15	16	19	0.22%	19.40%	IIAQTTVDAK	99.70%	30.1	29	19	1,059.60
ELF3-HFC Col rep1	| Symbols: PHYA, FHY2, FRE1, HY8 | phytochrome A | chr1:3095498-3099216 REVERSE LENGTH=1122	AT1G09570.1	TAIR10_20101214.fasta	124,503.20	100.00%	15	16	19	0.22%	19.40%	LIETATVPILAVDSDGLVNGWNTK	99.70%	36.4	29	33.4	2,526.35
ELF3-HFC Col rep1	| Symbols: PHYA, FHY2, FRE1, HY8 | phytochrome A | chr1:3095498-3099216 REVERSE LENGTH=1122	AT1G09570.1	TAIR10_20101214.fasta	124,503.20	100.00%	15	16	19	0.22%	19.40%	LIQPFGCLLALDEK	99.70%	60.8	30.1	51.7	1,616.87
ELF3-HFC Col rep1	| Symbols: PHYA, FHY2, FRE1, HY8 | phytochrome A | chr1:3095498-3099216 REVERSE LENGTH=1122	AT1G09570.1	TAIR10_20101214.fasta	124,503.20	100.00%	15	16	19	0.22%	19.40%	MLLGEVFGTQK	99.60%	33	29	16.3	1,238.65
ELF3-HFC Col rep1	| Symbols: PHYA, FHY2, FRE1, HY8 | phytochrome A | chr1:3095498-3099216 REVERSE LENGTH=1122	AT1G09570.1	TAIR10_20101214.fasta	124,503.20	100.00%	15	16	19	0.22%	19.40%	NQEAFVNLGIVLNNAVTSQDPEK	99.70%	47.9	31.3	38.5	2,500.27
ELF3-HFC Col rep1	| Symbols: PHYA, FHY2, FRE1, HY8 | phytochrome A | chr1:3095498-3099216 REVERSE LENGTH=1122	AT1G09570.1	TAIR10_20101214.fasta	124,503.20	100.00%	15	16	19	0.22%	19.40%	SLFTAPSASALQK	99.70%	49.3	29.3	42.3	1,320.72
ELF3-HFC Col rep1	| Symbols: PHYA, FHY2, FRE1, HY8 | phytochrome A | chr1:3095498-3099216 REVERSE LENGTH=1122	AT1G09570.1	TAIR10_20101214.fasta	124,503.20	100.00%	15	16	19	0.22%	19.40%	SSFIITAELAAANK	99.70%	61.3	29.2	49.2	1,435.78
ELF3-HFC Col rep1	| Symbols: PHYA, FHY2, FRE1, HY8 | phytochrome A | chr1:3095498-3099216 REVERSE LENGTH=1122	AT1G09570.1	TAIR10_20101214.fasta	124,503.20	100.00%	15	16	19	0.22%	19.40%	SVHLANLEIR	99.70%	55.5	25.1	44.9	1,151.65
ELF3-HFC Col rep1	| Symbols: PHYA, FHY2, FRE1, HY8 | phytochrome A | chr1:3095498-3099216 REVERSE LENGTH=1122	AT1G09570.1	TAIR10_20101214.fasta	124,503.20	100.00%	15	16	19	0.22%	19.40%	TSAKPFYAIIHR	99.70%	41.7	28.5	36.6	1,403.78
ELF3-HFC Col rep1	| Symbols: PHYA, FHY2, FRE1, HY8 | phytochrome A | chr1:3095498-3099216 REVERSE LENGTH=1122	AT1G09570.1	TAIR10_20101214.fasta	124,503.20	100.00%	15	16	19	0.22%	19.40%	VSFAFFTR	99.00%	49.7	29.3	34	974.5093
ELF3-HFC Col rep1	| Symbols: PHYA, FHY2, FRE1, HY8 | phytochrome A | chr1:3095498-3099216 REVERSE LENGTH=1122	AT1G09570.1	TAIR10_20101214.fasta	124,503.20	100.00%	15	16	19	0.22%	19.40%	VTGPVVENQPPR	99.70%	58.2	29.5	44.2	1,292.70
ELF3-HFC Col rep1	| Symbols: PHYB, HY3, OOP1 | phytochrome B | chr2:8140079-8144151 FORWARD LENGTH=1172	AT2G18790.1	TAIR10_20101214.fasta	129,333.90	100.00%	39	55	124	1.44%	51.50%	AIQQYTVDAR	99.70%	60.2	29.9	49.2	1,164.60
ELF3-HFC Col rep1	| Symbols: PHYB, HY3, OOP1 | phytochrome B | chr2:8140079-8144151 FORWARD LENGTH=1172	AT2G18790.1	TAIR10_20101214.fasta	129,333.90	100.00%	39	55	124	1.44%	51.50%	AISQLQALPGGDIK	99.70%	100	26.1	87	1,410.80
ELF3-HFC Col rep1	| Symbols: PHYB, HY3, OOP1 | phytochrome B | chr2:8140079-8144151 FORWARD LENGTH=1172	AT2G18790.1	TAIR10_20101214.fasta	129,333.90	100.00%	39	55	124	1.44%	51.50%	AKELAYICQVIK	99.70%	58.6	27.3	44.7	1,435.80
ELF3-HFC Col rep1	| Symbols: PHYB, HY3, OOP1 | phytochrome B | chr2:8140079-8144151 FORWARD LENGTH=1172	AT2G18790.1	TAIR10_20101214.fasta	129,333.90	100.00%	39	55	124	1.44%	51.50%	ALRGDEEKNVEVK	99.70%	65	29.7	42.3	1,486.79
ELF3-HFC Col rep1	| Symbols: PHYB, HY3, OOP1 | phytochrome B | chr2:8140079-8144151 FORWARD LENGTH=1172	AT2G18790.1	TAIR10_20101214.fasta	129,333.90	100.00%	39	55	124	1.44%	51.50%	AVFVVVNACSSK	99.70%	65.7	29.7	57.3	1,280.67
ELF3-HFC Col rep1	| Symbols: PHYB, HY3, OOP1 | phytochrome B | chr2:8140079-8144151 FORWARD LENGTH=1172	AT2G18790.1	TAIR10_20101214.fasta	129,333.90	100.00%	39	55	124	1.44%	51.50%	DDLEPYIGLHYPATDIPQASR	99.70%	64.5	31.3	0	2,372.14
ELF3-HFC Col rep1	| Symbols: PHYB, HY3, OOP1 | phytochrome B | chr2:8140079-8144151 FORWARD LENGTH=1172	AT2G18790.1	TAIR10_20101214.fasta	129,333.90	100.00%	39	55	124	1.44%	51.50%	DIPEEIKSIEVFGDQIR	99.70%	78.9	30.4	70.2	1,988.03
ELF3-HFC Col rep1	| Symbols: PHYB, HY3, OOP1 | phytochrome B | chr2:8140079-8144151 FORWARD LENGTH=1172	AT2G18790.1	TAIR10_20101214.fasta	129,333.90	100.00%	39	55	124	1.44%	51.50%	DSPAGIVTQSPSIMDLVK	99.70%	82	30.7	73.3	1,873.96
ELF3-HFC Col rep1	| Symbols: PHYB, HY3, OOP1 | phytochrome B | chr2:8140079-8144151 FORWARD LENGTH=1172	AT2G18790.1	TAIR10_20101214.fasta	129,333.90	100.00%	39	55	124	1.44%	51.50%	DYLNNIVGVCFVGQDVTSQK	99.70%	36	31.4	29.3	2,256.10
ELF3-HFC Col rep1	| Symbols: PHYB, HY3, OOP1 | phytochrome B | chr2:8140079-8144151 FORWARD LENGTH=1172	AT2G18790.1	TAIR10_20101214.fasta	129,333.90	100.00%	39	55	124	1.44%	51.50%	EITLLNPVWIHSK	99.70%	85	26.7	66.7	1,549.87
ELF3-HFC Col rep1	| Symbols: PHYB, HY3, OOP1 | phytochrome B | chr2:8140079-8144151 FORWARD LENGTH=1172	AT2G18790.1	TAIR10_20101214.fasta	129,333.90	100.00%	39	55	124	1.44%	51.50%	ELAYICQVIK	96.70%	23.5	29	12.7	1,236.67
ELF3-HFC Col rep1	| Symbols: PHYB, HY3, OOP1 | phytochrome B | chr2:8140079-8144151 FORWARD LENGTH=1172	AT2G18790.1	TAIR10_20101214.fasta	129,333.90	100.00%	39	55	124	1.44%	51.50%	FANSLLEATDLNEDQK	99.70%	60.4	31	46	1,807.87
ELF3-HFC Col rep1	| Symbols: PHYB, HY3, OOP1 | phytochrome B | chr2:8140079-8144151 FORWARD LENGTH=1172	AT2G18790.1	TAIR10_20101214.fasta	129,333.90	100.00%	39	55	124	1.44%	51.50%	FHEDEHGEVVAESK	99.70%	118	27.5	107	1,612.72
ELF3-HFC Col rep1	| Symbols: PHYB, HY3, OOP1 | phytochrome B | chr2:8140079-8144151 FORWARD LENGTH=1172	AT2G18790.1	TAIR10_20101214.fasta	129,333.90	100.00%	39	55	124	1.44%	51.50%	FINIQGDYK	99.70%	56.8	28	41.2	1,097.56
ELF3-HFC Col rep1	| Symbols: PHYB, HY3, OOP1 | phytochrome B | chr2:8140079-8144151 FORWARD LENGTH=1172	AT2G18790.1	TAIR10_20101214.fasta	129,333.90	100.00%	39	55	124	1.44%	51.50%	FMIVLHNAIGGQDTDKFPFPFFDR	99.70%	38.3	31.8	29	2,828.39
ELF3-HFC Col rep1	| Symbols: PHYB, HY3, OOP1 | phytochrome B | chr2:8140079-8144151 FORWARD LENGTH=1172	AT2G18790.1	TAIR10_20101214.fasta	129,333.90	100.00%	39	55	124	1.44%	51.50%	FVQALLTANK	99.70%	38.6	26.1	19.1	1,104.64
ELF3-HFC Col rep1	| Symbols: PHYB, HY3, OOP1 | phytochrome B | chr2:8140079-8144151 FORWARD LENGTH=1172	AT2G18790.1	TAIR10_20101214.fasta	129,333.90	100.00%	39	55	124	1.44%	51.50%	FVQALLTANKR	99.70%	44.9	25	32.2	1,260.74
ELF3-HFC Col rep1	| Symbols: PHYB, HY3, OOP1 | phytochrome B | chr2:8140079-8144151 FORWARD LENGTH=1172	AT2G18790.1	TAIR10_20101214.fasta	129,333.90	100.00%	39	55	124	1.44%	51.50%	GDEEKNVEVK	99.70%	38	28.6	22.3	1,146.56
ELF3-HFC Col rep1	| Symbols: PHYB, HY3, OOP1 | phytochrome B | chr2:8140079-8144151 FORWARD LENGTH=1172	AT2G18790.1	TAIR10_20101214.fasta	129,333.90	100.00%	39	55	124	1.44%	51.50%	GGYIQPFGCMIAVDESSFR	99.70%	39.2	29	31.6	2,149.97
ELF3-HFC Col rep1	| Symbols: PHYB, HY3, OOP1 | phytochrome B | chr2:8140079-8144151 FORWARD LENGTH=1172	AT2G18790.1	TAIR10_20101214.fasta	129,333.90	100.00%	39	55	124	1.44%	51.50%	IAELTGLSVEEAMGK	99.70%	103	31.1	98	1,563.79
ELF3-HFC Col rep1	| Symbols: PHYB, HY3, OOP1 | phytochrome B | chr2:8140079-8144151 FORWARD LENGTH=1172	AT2G18790.1	TAIR10_20101214.fasta	129,333.90	100.00%	39	55	124	1.44%	51.50%	IDVGVVIDLEPAR	99.70%	71.2	27	60.4	1,395.78
ELF3-HFC Col rep1	| Symbols: PHYB, HY3, OOP1 | phytochrome B | chr2:8140079-8144151 FORWARD LENGTH=1172	AT2G18790.1	TAIR10_20101214.fasta	129,333.90	100.00%	39	55	124	1.44%	51.50%	IDVGVVIDLEPARTEDPALSIAGAVQSQK	99.70%	43.6	27.9	36.9	2,991.60
ELF3-HFC Col rep1	| Symbols: PHYB, HY3, OOP1 | phytochrome B | chr2:8140079-8144151 FORWARD LENGTH=1172	AT2G18790.1	TAIR10_20101214.fasta	129,333.90	100.00%	39	55	124	1.44%	51.50%	IIGYSENAR	99.70%	39.3	29.5	22	1,022.53
ELF3-HFC Col rep1	| Symbols: PHYB, HY3, OOP1 | phytochrome B | chr2:8140079-8144151 FORWARD LENGTH=1172	AT2G18790.1	TAIR10_20101214.fasta	129,333.90	100.00%	39	55	124	1.44%	51.50%	IVGDMDLESIEDGSFVLK	99.70%	78.6	31.1	69.5	1,982.96
ELF3-HFC Col rep1	| Symbols: PHYB, HY3, OOP1 | phytochrome B | chr2:8140079-8144151 FORWARD LENGTH=1172	AT2G18790.1	TAIR10_20101214.fasta	129,333.90	100.00%	39	55	124	1.44%	51.50%	IVGDMDLESIEDGSFVLKR	99.70%	43.4	31.4	35	2,139.06
ELF3-HFC Col rep1	| Symbols: PHYB, HY3, OOP1 | phytochrome B | chr2:8140079-8144151 FORWARD LENGTH=1172	AT2G18790.1	TAIR10_20101214.fasta	129,333.90	100.00%	39	55	124	1.44%	51.50%	LHAVFEQSGESGK	99.70%	32	29.4	29.2	1,388.68
ELF3-HFC Col rep1	| Symbols: PHYB, HY3, OOP1 | phytochrome B | chr2:8140079-8144151 FORWARD LENGTH=1172	AT2G18790.1	TAIR10_20101214.fasta	129,333.90	100.00%	39	55	124	1.44%	51.50%	LIETATVPIFAVDAGGCINGWNAK	99.70%	47.3	31.2	42.1	2,517.28
ELF3-HFC Col rep1	| Symbols: PHYB, HY3, OOP1 | phytochrome B | chr2:8140079-8144151 FORWARD LENGTH=1172	AT2G18790.1	TAIR10_20101214.fasta	129,333.90	100.00%	39	55	124	1.44%	51.50%	LKTFSPELQGK	99.70%	49.4	28.1	34.5	1,247.70
ELF3-HFC Col rep1	| Symbols: PHYB, HY3, OOP1 | phytochrome B | chr2:8140079-8144151 FORWARD LENGTH=1172	AT2G18790.1	TAIR10_20101214.fasta	129,333.90	100.00%	39	55	124	1.44%	51.50%	LLCDTVVESVR	99.70%	73	30.6	46.5	1,290.67
ELF3-HFC Col rep1	| Symbols: PHYB, HY3, OOP1 | phytochrome B | chr2:8140079-8144151 FORWARD LENGTH=1172	AT2G18790.1	TAIR10_20101214.fasta	129,333.90	100.00%	39	55	124	1.44%	51.50%	LMNGEVQYIR	99.70%	36.5	29.3	23.9	1,238.62
ELF3-HFC Col rep1	| Symbols: PHYB, HY3, OOP1 | phytochrome B | chr2:8140079-8144151 FORWARD LENGTH=1172	AT2G18790.1	TAIR10_20101214.fasta	129,333.90	100.00%	39	55	124	1.44%	51.50%	LTQSMCLVGSTLR	99.70%	68.8	29.9	61.5	1,481.75
ELF3-HFC Col rep1	| Symbols: PHYB, HY3, OOP1 | phytochrome B | chr2:8140079-8144151 FORWARD LENGTH=1172	AT2G18790.1	TAIR10_20101214.fasta	129,333.90	100.00%	39	55	124	1.44%	51.50%	MIVDCNATPVLVVQDDR	99.70%	28	30.7	24.8	1,960.95
ELF3-HFC Col rep1	| Symbols: PHYB, HY3, OOP1 | phytochrome B | chr2:8140079-8144151 FORWARD LENGTH=1172	AT2G18790.1	TAIR10_20101214.fasta	129,333.90	100.00%	39	55	124	1.44%	51.50%	NTGKPFYAILHR	99.70%	52.7	29.6	42.6	1,416.77
ELF3-HFC Col rep1	| Symbols: PHYB, HY3, OOP1 | phytochrome B | chr2:8140079-8144151 FORWARD LENGTH=1172	AT2G18790.1	TAIR10_20101214.fasta	129,333.90	100.00%	39	55	124	1.44%	51.50%	QLLETSVSCEK	99.70%	56.4	30.4	40.8	1,293.64
ELF3-HFC Col rep1	| Symbols: PHYB, HY3, OOP1 | phytochrome B | chr2:8140079-8144151 FORWARD LENGTH=1172	AT2G18790.1	TAIR10_20101214.fasta	129,333.90	100.00%	39	55	124	1.44%	51.50%	QMADGFAAIR	99.70%	58.8	28.5	41.8	1,095.53
ELF3-HFC Col rep1	| Symbols: PHYB, HY3, OOP1 | phytochrome B | chr2:8140079-8144151 FORWARD LENGTH=1172	AT2G18790.1	TAIR10_20101214.fasta	129,333.90	100.00%	39	55	124	1.44%	51.50%	RDDLEPYIGLHYPATDIPQASR	99.70%	84.4	31.5	0	2,527.26
ELF3-HFC Col rep1	| Symbols: PHYB, HY3, OOP1 | phytochrome B | chr2:8140079-8144151 FORWARD LENGTH=1172	AT2G18790.1	TAIR10_20101214.fasta	129,333.90	100.00%	39	55	124	1.44%	51.50%	RQDTECFTK	99.70%	39	26.1	13.9	1,184.54
ELF3-HFC Col rep1	| Symbols: PHYB, HY3, OOP1 | phytochrome B | chr2:8140079-8144151 FORWARD LENGTH=1172	AT2G18790.1	TAIR10_20101214.fasta	129,333.90	100.00%	39	55	124	1.44%	51.50%	SFDYSQSLK	99.70%	42.3	26.9	27	1,074.51
ELF3-HFC Col rep1	| Symbols: PHYB, HY3, OOP1 | phytochrome B | chr2:8140079-8144151 FORWARD LENGTH=1172	AT2G18790.1	TAIR10_20101214.fasta	129,333.90	100.00%	39	55	124	1.44%	51.50%	SIEVFGDQIR	99.70%	65	30.5	42.4	1,163.61
ELF3-HFC Col rep1	| Symbols: PHYB, HY3, OOP1 | phytochrome B | chr2:8140079-8144151 FORWARD LENGTH=1172	AT2G18790.1	TAIR10_20101214.fasta	129,333.90	100.00%	39	55	124	1.44%	51.50%	SLFTSSSSILLER	99.70%	94.3	28.2	87.2	1,439.77
ELF3-HFC Col rep1	| Symbols: PHYB, HY3, OOP1 | phytochrome B | chr2:8140079-8144151 FORWARD LENGTH=1172	AT2G18790.1	TAIR10_20101214.fasta	129,333.90	100.00%	39	55	124	1.44%	51.50%	SLVSDLIYK	99.70%	56.1	26.1	37.1	1,037.59
ELF3-HFC Col rep1	| Symbols: PHYB, HY3, OOP1 | phytochrome B | chr2:8140079-8144151 FORWARD LENGTH=1172	AT2G18790.1	TAIR10_20101214.fasta	129,333.90	100.00%	39	55	124	1.44%	51.50%	SLVSDLIYKENEATVNK	99.70%	78.5	30	64.8	1,923.01
ELF3-HFC Col rep1	| Symbols: PHYB, HY3, OOP1 | phytochrome B | chr2:8140079-8144151 FORWARD LENGTH=1172	AT2G18790.1	TAIR10_20101214.fasta	129,333.90	100.00%	39	55	124	1.44%	51.50%	SSFQAFLEVVK	99.70%	76.1	26.8	62.2	1,254.67
ELF3-HFC Col rep1	| Symbols: PHYB, HY3, OOP1 | phytochrome B | chr2:8140079-8144151 FORWARD LENGTH=1172	AT2G18790.1	TAIR10_20101214.fasta	129,333.90	100.00%	39	55	124	1.44%	51.50%	SYFLIILELPVPR	99.70%	56.1	25	56.1	1,559.92

ELF3-HFC Col rep1	| Symbols: PHYB, HY3, OOP1 | phytochrome B | chr2:8140079-8144151 FORWARD LENGTH=1172	AT2G18790.1	TAIR10_20101214.fasta	129,333.90	100.00%	39	55	124	1.44%	51.50%	TEDPALSIAGAVQSQK	99.70%	118	29.5	95.9	1,614.83
ELF3-HFC Col rep1	| Symbols: PHYB, HY3, OOP1 | phytochrome B | chr2:8140079-8144151 FORWARD LENGTH=1172	AT2G18790.1	TAIR10_20101214.fasta	129,333.90	100.00%	39	55	124	1.44%	51.50%	TFSPELQGK	99.40%	22.6	30	21.7	1,006.52
ELF3-HFC Col rep1	| Symbols: PHYB, HY3, OOP1 | phytochrome B | chr2:8140079-8144151 FORWARD LENGTH=1172	AT2G18790.1	TAIR10_20101214.fasta	129,333.90	100.00%	39	55	124	1.44%	51.50%	TQTLLCDMLLR	99.70%	37	30.3	23.6	1,379.70
ELF3-HFC Col rep1	| Symbols: PHYB, HY3, OOP1 | phytochrome B | chr2:8140079-8144151 FORWARD LENGTH=1172	AT2G18790.1	TAIR10_20101214.fasta	129,333.90	100.00%	39	55	124	1.44%	51.50%	TTTYGSSVPEQQITAYLSR	99.70%	36.1	31.1	28.5	2,102.04
ELF3-HFC Col rep1	| Symbols: PHYB, HY3, OOP1 | phytochrome B | chr2:8140079-8144151 FORWARD LENGTH=1172	AT2G18790.1	TAIR10_20101214.fasta	129,333.90	100.00%	39	55	124	1.44%	51.50%	VVDGVVQPCR	99.70%	46.5	27.3	42	1,128.58
ELF3-HFC Col rep1	| Symbols: PHYB, HY3, OOP1 | phytochrome B | chr2:8140079-8144151 FORWARD LENGTH=1172	AT2G18790.1	TAIR10_20101214.fasta	129,333.90	100.00%	39	55	124	1.44%	51.50%	WTSPEGLGLSVCR	99.70%	56.8	29.8	46.3	1,461.72
ELF3-HFC Col rep1	| Symbols: PHYB, HY3, OOP1 | phytochrome B | chr2:8140079-8144151 FORWARD LENGTH=1172	AT2G18790.1	TAIR10_20101214.fasta	129,333.90	100.00%	39	55	124	1.44%	51.50%	YAPSQEWVEIHLSQLSK	99.70%	50.9	30.6	36.2	2,015.02
ELF3-HFC Col rep1	| Symbols: PHYB, HY3, OOP1 | phytochrome B | chr2:8140079-8144151 FORWARD LENGTH=1172	AT2G18790.1	TAIR10_20101214.fasta	129,333.90	100.00%	39	55	124	1.44%	51.50%	YYPLGVAPSEVQIK	99.70%	62.2	29.5	53.5	1,563.84
ELF3-HFC Col rep1	| Symbols: PHYC | phytochrome C | chr5:14008049-14011619 FORWARD LENGTH=1111	AT5G35840.1	TAIR10_20101214.fasta	123,724.60	100.00%	37	49	70	0.81%	46.90%	AAEVTGLAVEQAIGKPVSDLVEDDSVETVK	99.70%	56.5	30.5	51.9	3,069.58
ELF3-HFC Col rep1	| Symbols: PHYC | phytochrome C | chr5:14008049-14011619 FORWARD LENGTH=1111	AT5G35840.1	TAIR10_20101214.fasta	123,724.60	100.00%	37	49	70	0.81%	46.90%	AISFLQDLLHSSGLSEDQK	99.70%	28.4	30.8	24.7	2,088.06
ELF3-HFC Col rep1	| Symbols: PHYC | phytochrome C | chr5:14008049-14011619 FORWARD LENGTH=1111	AT5G35840.1	TAIR10_20101214.fasta	123,724.60	100.00%	37	49	70	0.81%	46.90%	AISFLQDLLHSSGLSEDQKR	99.70%	69.9	30.4	62.8	2,244.16
ELF3-HFC Col rep1	| Symbols: PHYC | phytochrome C | chr5:14008049-14011619 FORWARD LENGTH=1111	AT5G35840.1	TAIR10_20101214.fasta	123,724.60	100.00%	37	49	70	0.81%	46.90%	AVDFGEISILNPITLHCR	99.70%	59.9	30	48.6	2,055.07
ELF3-HFC Col rep1	| Symbols: PHYC | phytochrome C | chr5:14008049-14011619 FORWARD LENGTH=1111	AT5G35840.1	TAIR10_20101214.fasta	123,724.60	100.00%	37	49	70	0.81%	46.90%	CDGAALYYR	99.70%	49	25	41.9	1,088.48
ELF3-HFC Col rep1	| Symbols: PHYC | phytochrome C | chr5:14008049-14011619 FORWARD LENGTH=1111	AT5G35840.1	TAIR10_20101214.fasta	123,724.60	100.00%	37	49	70	0.81%	46.90%	DGSFIEALLSANK	99.70%	101	30	88.8	1,364.71
ELF3-HFC Col rep1	| Symbols: PHYC | phytochrome C | chr5:14008049-14011619 FORWARD LENGTH=1111	AT5G35840.1	TAIR10_20101214.fasta	123,724.60	100.00%	37	49	70	0.81%	46.90%	DGSFIEALLSANKR	99.70%	81.7	29.4	58.1	1,520.81
ELF3-HFC Col rep1	| Symbols: PHYC | phytochrome C | chr5:14008049-14011619 FORWARD LENGTH=1111	AT5G35840.1	TAIR10_20101214.fasta	123,724.60	100.00%	37	49	70	0.81%	46.90%	DMTNNVLGVCFIGQDVTGQK	99.70%	30.9	30.6	21	2,212.04
ELF3-HFC Col rep1	| Symbols: PHYC | phytochrome C | chr5:14008049-14011619 FORWARD LENGTH=1111	AT5G35840.1	TAIR10_20101214.fasta	123,724.60	100.00%	37	49	70	0.81%	46.90%	DNLWSLGVTPTETQIR	99.70%	71.4	30.4	60.1	1,829.94
ELF3-HFC Col rep1	| Symbols: PHYC | phytochrome C | chr5:14008049-14011619 FORWARD LENGTH=1111	AT5G35840.1	TAIR10_20101214.fasta	123,724.60	100.00%	37	49	70	0.81%	46.90%	EAESAVLLK	98.30%	31.2	30.5	12	959.5408
ELF3-HFC Col rep1	| Symbols: PHYC | phytochrome C | chr5:14008049-14011619 FORWARD LENGTH=1111	AT5G35840.1	TAIR10_20101214.fasta	123,724.60	100.00%	37	49	70	0.81%	46.90%	EALTIGTDVK	99.70%	50	29.7	36	1,046.57
ELF3-HFC Col rep1	| Symbols: PHYC | phytochrome C | chr5:14008049-14011619 FORWARD LENGTH=1111	AT5G35840.1	TAIR10_20101214.fasta	123,724.60	100.00%	37	49	70	0.81%	46.90%	EGLGLHITQK	99.70%	38.2	25.9	28.5	1,095.62
ELF3-HFC Col rep1	| Symbols: PHYC | phytochrome C | chr5:14008049-14011619 FORWARD LENGTH=1111	AT5G35840.1	TAIR10_20101214.fasta	123,724.60	100.00%	37	49	70	0.81%	46.90%	EVSELTGYDR	99.70%	45.2	27.5	32.3	1,168.55
ELF3-HFC Col rep1	| Symbols: PHYC | phytochrome C | chr5:14008049-14011619 FORWARD LENGTH=1111	AT5G35840.1	TAIR10_20101214.fasta	123,724.60	100.00%	37	49	70	0.81%	46.90%	GMLIQPFGCLIVVDEK	99.70%	65.6	30.7	36.8	1,834.94
ELF3-HFC Col rep1	| Symbols: PHYC | phytochrome C | chr5:14008049-14011619 FORWARD LENGTH=1111	AT5G35840.1	TAIR10_20101214.fasta	123,724.60	100.00%	37	49	70	0.81%	46.90%	IEEGLVIDLEPVSPDEVPVTAAGALR	99.70%	64.3	28.7	54.7	2,689.43
ELF3-HFC Col rep1	| Symbols: PHYC | phytochrome C | chr5:14008049-14011619 FORWARD LENGTH=1111	AT5G35840.1	TAIR10_20101214.fasta	123,724.60	100.00%	37	49	70	0.81%	46.90%	IGFNAVISGQK	99.70%	81.6	28.5	59.1	1,133.63
ELF3-HFC Col rep1	| Symbols: PHYC | phytochrome C | chr5:14008049-14011619 FORWARD LENGTH=1111	AT5G35840.1	TAIR10_20101214.fasta	123,724.60	100.00%	37	49	70	0.81%	46.90%	IIHPAPGLPEDLVR	99.70%	88.1	25.9	76.3	1,526.87
ELF3-HFC Col rep1	| Symbols: PHYC | phytochrome C | chr5:14008049-14011619 FORWARD LENGTH=1111	AT5G35840.1	TAIR10_20101214.fasta	123,724.60	100.00%	37	49	70	0.81%	46.90%	ILLGEVFTTDDYGCCLK	99.70%	91.1	30.2	83.1	2,003.95
ELF3-HFC Col rep1	| Symbols: PHYC | phytochrome C | chr5:14008049-14011619 FORWARD LENGTH=1111	AT5G35840.1	TAIR10_20101214.fasta	123,724.60	100.00%	37	49	70	0.81%	46.90%	ILQTQSVLCDMLFR	99.70%	78.9	30.6	71.6	1,723.89
ELF3-HFC Col rep1	| Symbols: PHYC | phytochrome C | chr5:14008049-14011619 FORWARD LENGTH=1111	AT5G35840.1	TAIR10_20101214.fasta	123,724.60	100.00%	37	49	70	0.81%	46.90%	LHGNFEESER	99.70%	46.1	25.8	28.2	1,217.55
ELF3-HFC Col rep1	| Symbols: PHYC | phytochrome C | chr5:14008049-14011619 FORWARD LENGTH=1111	AT5G35840.1	TAIR10_20101214.fasta	123,724.60	100.00%	37	49	70	0.81%	46.90%	LIDTAAVPIFAVDASGVINGWNSK	99.70%	57.9	30.3	5.98	2,459.28
ELF3-HFC Col rep1	| Symbols: PHYC | phytochrome C | chr5:14008049-14011619 FORWARD LENGTH=1111	AT5G35840.1	TAIR10_20101214.fasta	123,724.60	100.00%	37	49	70	0.81%	46.90%	LLFGFYHR	99.00%	29	27.6	18.6	1,052.57
ELF3-HFC Col rep1	| Symbols: PHYC | phytochrome C | chr5:14008049-14011619 FORWARD LENGTH=1111	AT5G35840.1	TAIR10_20101214.fasta	123,724.60	100.00%	37	49	70	0.81%	46.90%	LQALPSGNMLLLCDALVK	99.50%	30.2	28.8	17.3	1,972.06
ELF3-HFC Col rep1	| Symbols: PHYC | phytochrome C | chr5:14008049-14011619 FORWARD LENGTH=1111	AT5G35840.1	TAIR10_20101214.fasta	123,724.60	100.00%	37	49	70	0.81%	46.90%	LQQILSETLLSSIR	99.70%	118	26	115	1,600.93
ELF3-HFC Col rep1	| Symbols: PHYC | phytochrome C | chr5:14008049-14011619 FORWARD LENGTH=1111	AT5G35840.1	TAIR10_20101214.fasta	123,724.60	100.00%	37	49	70	0.81%	46.90%	NAPIGIVTQSPNIMDLVK	99.70%	81.6	28.1	66.9	1,910.04
ELF3-HFC Col rep1	| Symbols: PHYC | phytochrome C | chr5:14008049-14011619 FORWARD LENGTH=1111	AT5G35840.1	TAIR10_20101214.fasta	123,724.60	100.00%	37	49	70	0.81%	46.90%	NMLALALEGSEER	99.70%	94.2	30.2	77.2	1,448.71
ELF3-HFC Col rep1	| Symbols: PHYC | phytochrome C | chr5:14008049-14011619 FORWARD LENGTH=1111	AT5G35840.1	TAIR10_20101214.fasta	123,724.60	100.00%	37	49	70	0.81%	46.90%	QVMELSIER	99.70%	44.6	30.3	29	1,120.57
ELF3-HFC Col rep1	| Symbols: PHYC | phytochrome C | chr5:14008049-14011619 FORWARD LENGTH=1111	AT5G35840.1	TAIR10_20101214.fasta	123,724.60	100.00%	37	49	70	0.81%	46.90%	SLFLSPGCSALEK	99.70%	45.2	30	26.8	1,408.71
ELF3-HFC Col rep1	| Symbols: PHYC | phytochrome C | chr5:14008049-14011619 FORWARD LENGTH=1111	AT5G35840.1	TAIR10_20101214.fasta	123,724.60	100.00%	37	49	70	0.81%	46.90%	SLSQPISLSGSTLR	99.70%	106	29.1	86.5	1,445.80
ELF3-HFC Col rep1	| Symbols: PHYC | phytochrome C | chr5:14008049-14011619 FORWARD LENGTH=1111	AT5G35840.1	TAIR10_20101214.fasta	123,724.60	100.00%	37	49	70	0.81%	46.90%	SSPVELVVNTCCSR	99.70%	59.7	29.4	50	1,607.75
ELF3-HFC Col rep1	| Symbols: PHYC | phytochrome C | chr5:14008049-14011619 FORWARD LENGTH=1111	AT5G35840.1	TAIR10_20101214.fasta	123,724.60	100.00%	37	49	70	0.81%	46.90%	SSSKPFYAILHR	99.70%	63.3	29.5	48.9	1,405.76
ELF3-HFC Col rep1	| Symbols: PHYC | phytochrome C | chr5:14008049-14011619 FORWARD LENGTH=1111	AT5G35840.1	TAIR10_20101214.fasta	123,724.60	100.00%	37	49	70	0.81%	46.90%	SVPWDDMEMDAINSLQLIIK	99.70%	33.7	31.5	21.3	2,350.13
ELF3-HFC Col rep1	| Symbols: PHYC | phytochrome C | chr5:14008049-14011619 FORWARD LENGTH=1111	AT5G35840.1	TAIR10_20101214.fasta	123,724.60	100.00%	37	49	70	0.81%	46.90%	TLTENYSR	99.00%	48.7	27.7	13.2	983.4792
ELF3-HFC Col rep1	| Symbols: PHYC | phytochrome C | chr5:14008049-14011619 FORWARD LENGTH=1111	AT5G35840.1	TAIR10_20101214.fasta	123,724.60	100.00%	37	49	70	0.81%	46.90%	TVVDVPLVDNR	99.70%	76.2	26.9	63.5	1,226.67
ELF3-HFC Col rep1	| Symbols: PHYC | phytochrome C | chr5:14008049-14011619 FORWARD LENGTH=1111	AT5G35840.1	TAIR10_20101214.fasta	123,724.60	100.00%	37	49	70	0.81%	46.90%	VIAFSENTQEMLGLIPHTVPSMEQR	99.70%	61.6	31.7	55.6	2,843.41
ELF3-HFC Col rep1	| Symbols: PHYC | phytochrome C | chr5:14008049-14011619 FORWARD LENGTH=1111	AT5G35840.1	TAIR10_20101214.fasta	123,724.60	100.00%	37	49	70	0.81%	46.90%	VSSQVLVDAK	99.70%	74.7	29.1	58.5	1,045.59
ELF3-HFC Col rep1	| Symbols: PHYC | phytochrome C | chr5:14008049-14011619 FORWARD LENGTH=1111	AT5G35840.1	TAIR10_20101214.fasta	123,724.60	100.00%	37	49	70	0.81%	46.90%	YACEFLTQVFGVQINK	99.70%	33.4	30.5	18.9	1,916.96
ELF3-HFC Col rep1	| Symbols: PHYD | phytochrome D | chr4:9195602-9199486 REVERSE LENGTH=1164	AT4G16250.1	TAIR10_20101214.fasta	129,270.30	100.00%	7	7	38	0.44%	20.40%	AIQQYTVDAR	99.70%	60.2	29.9	49.2	1,164.60
ELF3-HFC Col rep1	| Symbols: PHYD | phytochrome D | chr4:9195602-9199486 REVERSE LENGTH=1164	AT4G16250.1	TAIR10_20101214.fasta	129,270.30	100.00%	7	7	38	0.44%	20.40%	AISHLQSLPSGDIK	99.70%	44.4	28.2	34.4	1,465.80
ELF3-HFC Col rep1	| Symbols: PHYD | phytochrome D | chr4:9195602-9199486 REVERSE LENGTH=1164	AT4G16250.1	TAIR10_20101214.fasta	129,270.30	100.00%	7	7	38	0.44%	20.40%	ELAYIFQVIK	96.30%	22.9	25	14.6	1,223.70
ELF3-HFC Col rep1	| Symbols: PHYD | phytochrome D | chr4:9195602-9199486 REVERSE LENGTH=1164	AT4G16250.1	TAIR10_20101214.fasta	129,270.30	100.00%	7	7	38	0.44%	20.40%	FHEDEHGEVVAESK	99.70%	118	27.5	107	1,612.72
ELF3-HFC Col rep1	| Symbols: PHYD | phytochrome D | chr4:9195602-9199486 REVERSE LENGTH=1164	AT4G16250.1	TAIR10_20101214.fasta	129,270.30	100.00%	7	7	38	0.44%	20.40%	FINIQGDYK	99.70%	56.8	28	41.2	1,097.56
ELF3-HFC Col rep1	| Symbols: PHYD | phytochrome D | chr4:9195602-9199486 REVERSE LENGTH=1164	AT4G16250.1	TAIR10_20101214.fasta	129,270.30	100.00%	7	7	38	0.44%	20.40%	FMIVLHNAIGGQDTDKFPFPFFDR	99.70%	38.3	31.8	29	2,828.39
ELF3-HFC Col rep1	| Symbols: PHYD | phytochrome D | chr4:9195602-9199486 REVERSE LENGTH=1164	AT4G16250.1	TAIR10_20101214.fasta	129,270.30	100.00%	7	7	38	0.44%	20.40%	KGEFIQALLTLNKR	99.70%	31.7	25	29.3	1,630.96
ELF3-HFC Col rep1	| Symbols: PHYD | phytochrome D | chr4:9195602-9199486 REVERSE LENGTH=1164	AT4G16250.1	TAIR10_20101214.fasta	129,270.30	100.00%	7	7	38	0.44%	20.40%	LHAVFEQSGESGK	99.70%	32	29.4	29.2	1,388.68
ELF3-HFC Col rep1	| Symbols: PHYD | phytochrome D | chr4:9195602-9199486 REVERSE LENGTH=1164	AT4G16250.1	TAIR10_20101214.fasta	129,270.30	100.00%	7	7	38	0.44%	20.40%	LLCDTVVESVR	99.70%	73	30.6	46.5	1,290.67
ELF3-HFC Col rep1	| Symbols: PHYD | phytochrome D | chr4:9195602-9199486 REVERSE LENGTH=1164	AT4G16250.1	TAIR10_20101214.fasta	129,270.30	100.00%	7	7	38	0.44%	20.40%	LTQFICLVGSTLR	99.70%	41.4	27.5	31.4	1,507.83
ELF3-HFC Col rep1	| Symbols: PHYD | phytochrome D | chr4:9195602-9199486 REVERSE LENGTH=1164	AT4G16250.1	TAIR10_20101214.fasta	129,270.30	100.00%	7	7	38	0.44%	20.40%	NDLEPYIGLHYPATDIPQASR	99.70%	64.5	31.3	0	2,372.14
ELF3-HFC Col rep1	| Symbols: PHYD | phytochrome D | chr4:9195602-9199486 REVERSE LENGTH=1164	AT4G16250.1	TAIR10_20101214.fasta	129,270.30	100.00%	7	7	38	0.44%	20.40%	QLLETSVSCEK	99.70%	56.4	30.4	40.8	1,293.64
ELF3-HFC Col rep1	| Symbols: PHYD | phytochrome D | chr4:9195602-9199486 REVERSE LENGTH=1164	AT4G16250.1	TAIR10_20101214.fasta	129,270.30	100.00%	7	7	38	0.44%	20.40%	RNDLEPYIGLHYPATDIPQASR	99.70%	84.4	31.5	0	2,527.26
ELF3-HFC Col rep1	| Symbols: PHYD | phytochrome D | chr4:9195602-9199486 REVERSE LENGTH=1164	AT4G16250.1	TAIR10_20101214.fasta	129,270.30	100.00%	7	7	38	0.44%	20.40%	SEVLTIGTDLR	99.70%	74.1	29.5	58.7	1,203.66
ELF3-HFC Col rep1	| Symbols: PHYD | phytochrome D | chr4:9195602-9199486 REVERSE LENGTH=1164	AT4G16250.1	TAIR10_20101214.fasta	129,270.30	100.00%	7	7	38	0.44%	20.40%	SFDYSQSLK	99.70%	42.3	26.9	27	1,074.51
ELF3-HFC Col rep1	| Symbols: PHYD | phytochrome D | chr4:9195602-9199486 REVERSE LENGTH=1164	AT4G16250.1	TAIR10_20101214.fasta	129,270.30	100.00%	7	7	38	0.44%	20.40%	SSFQTFLEVVK	99.70%	52.4	27.7	37.8	1,284.68
ELF3-HFC Col rep1	| Symbols: PHYD | phytochrome D | chr4:9195602-9199486 REVERSE LENGTH=1164	AT4G16250.1	TAIR10_20101214.fasta	129,270.30	100.00%	7	7	38	0.44%	20.40%	TEDPALSIAGAVQSQK	99.70%	118	29.5	95.9	1,614.83
ELF3-HFC Col rep1	| Symbols: PHYD | phytochrome D | chr4:9195602-9199486 REVERSE LENGTH=1164	AT4G16250.1	TAIR10_20101214.fasta	129,270.30	100.00%	7	7	38	0.44%	20.40%	VDVGILIDLEPAR	98.70%	32.3	25.5	12	1,409.80
ELF3-HFC Col rep1	| Symbols: PHYD | phytochrome D | chr4:9195602-9199486 REVERSE LENGTH=1164	AT4G16250.1	TAIR10_20101214.fasta	129,270.30	100.00%	7	7	38	0.44%	20.40%	WTSPEGLGLSVCR	99.70%	56.8	29.8	46.3	1,461.72
ELF3-HFC Col rep1	| Symbols: PHYE | phytochrome E | chr4:10042312-10045948 REVERSE LENGTH=1112	AT4G18130.1	TAIR10_20101214.fasta	122,519.10	100.00%	25	27	36	0.42%	32.60%	AALESLLCK	99.70%	47.9	30.1	38.1	1,004.54
ELF3-HFC Col rep1	| Symbols: PHYE | phytochrome E | chr4:10042312-10045948 REVERSE LENGTH=1112	AT4G18130.1	TAIR10_20101214.fasta	122,519.10	100.00%	25	27	36	0.42%	32.60%	AASFTEISLLNPVLVHSR	99.70%	88	27	75.8	1,954.08
ELF3-HFC Col rep1	| Symbols: PHYE | phytochrome E | chr4:10042312-10045948 REVERSE LENGTH=1112	AT4G18130.1	TAIR10_20101214.fasta	122,519.10	100.00%	25	27	36	0.42%	32.60%	CFFQVDLQVK	99.40%	29.6	28.6	17.4	1,283.65
ELF3-HFC Col rep1	| Symbols: PHYE | phytochrome E | chr4:10042312-10045948 REVERSE LENGTH=1112	AT4G18130.1	TAIR10_20101214.fasta	122,519.10	100.00%	25	27	36	0.42%	32.60%	DGWVTPDGLGLK	98.90%	35.1	27.7	8.67	1,257.65
ELF3-HFC Col rep1	| Symbols: PHYE | phytochrome E | chr4:10042312-10045948 REVERSE LENGTH=1112	AT4G18130.1	TAIR10_20101214.fasta	122,519.10	100.00%	25	27	36	0.42%	32.60%	ESFTSSRPVLSGNGVAR	99.70%	28.8	31.4	22.2	1,764.89
ELF3-HFC Col rep1	| Symbols: PHYE | phytochrome E | chr4:10042312-10045948 REVERSE LENGTH=1112	AT4G18130.1	TAIR10_20101214.fasta	122,519.10	100.00%	25	27	36	0.42%	32.60%	GGLVQPFGCLIAVEEPSFR	99.70%	92	31.2	80.5	2,076.06
ELF3-HFC Col rep1	| Symbols: PHYE | phytochrome E | chr4:10042312-10045948 REVERSE LENGTH=1112	AT4G18130.1	TAIR10_20101214.fasta	122,519.10	100.00%	25	27	36	0.42%	32.60%	IDAGIVMDLEPAK	98.80%	30.4	29.6	13.8	1,387.71
ELF3-HFC Col rep1	| Symbols: PHYE | phytochrome E | chr4:10042312-10045948 REVERSE LENGTH=1112	AT4G18130.1	TAIR10_20101214.fasta	122,519.10	100.00%	25	27	36	0.42%	32.60%	ILGLSDNSSDFLGLLSLPSTSHSGEFDKVK	98.60%	32.9	30.6	8.6	3,163.62
ELF3-HFC Col rep1	| Symbols: PHYE | phytochrome E | chr4:10042312-10045948 REVERSE LENGTH=1112	AT4G18130.1	TAIR10_20101214.fasta	122,519.10	100.00%	25	27	36	0.42%	32.60%	LLESSEISASQR	99.70%	45.1	30.5	32.6	1,319.68
ELF3-HFC Col rep1	| Symbols: PHYE | phytochrome E | chr4:10042312-10045948 REVERSE LENGTH=1112	AT4G18130.1	TAIR10_20101214.fasta	122,519.10	100.00%	25	27	36	0.42%	32.60%	LQLILADLLR	99.70%	71.4	25	54.1	1,167.75
ELF3-HFC Col rep1	| Symbols: PHYE | phytochrome E | chr4:10042312-10045948 REVERSE LENGTH=1112	AT4G18130.1	TAIR10_20101214.fasta	122,519.10	100.00%	25	27	36	0.42%	32.60%	LQSLPGGDIGALCDTVVEDVQR	99.70%	57.2	31.8	49.2	2,342.17
ELF3-HFC Col rep1	| Symbols: PHYE | phytochrome E | chr4:10042312-10045948 REVERSE LENGTH=1112	AT4G18130.1	TAIR10_20101214.fasta	122,519.10	100.00%	25	27	36	0.42%	32.60%	NIVNHAPFPNSWVGISISPGQELSR	99.70%	73.5	31	57.9	2,719.40
ELF3-HFC Col rep1	| Symbols: PHYE | phytochrome E | chr4:10042312-10045948 REVERSE LENGTH=1112	AT4G18130.1	TAIR10_20101214.fasta	122,519.10	100.00%	25	27	36	0.42%	32.60%	QFLETSDACEK	95.70%	28.4	26	8.77	1,327.58
ELF3-HFC Col rep1	| Symbols: PHYE | phytochrome E | chr4:10042312-10045948 REVERSE LENGTH=1112	AT4G18130.1	TAIR10_20101214.fasta	122,519.10	100.00%	25	27	36	0.42%	32.60%	QITTIIESTDLK	99.70%	40.2	28.5	32.3	1,361.75
ELF3-HFC Col rep1	| Symbols: PHYE | phytochrome E | chr4:10042312-10045948 REVERSE LENGTH=1112	AT4G18130.1	TAIR10_20101214.fasta	122,519.10	100.00%	25	27	36	0.42%	32.60%	RPLCLVNSTLR	99.70%	39.7	28.6	29.4	1,328.75
ELF3-HFC Col rep1	| Symbols: PHYE | phytochrome E | chr4:10042312-10045948 REVERSE LENGTH=1112	AT4G18130.1	TAIR10_20101214.fasta	122,519.10	100.00%	25	27	36	0.42%	32.60%	RSDLEPYLGLHYPATDIPQAAR	99.70%	74.5	31.2	65.2	2,483.27
ELF3-HFC Col rep1	| Symbols: PHYE | phytochrome E | chr4:10042312-10045948 REVERSE LENGTH=1112	AT4G18130.1	TAIR10_20101214.fasta	122,519.10	100.00%	25	27	36	0.42%	32.60%	SDLEPYLGLHYPATDIPQAAR	99.70%	55.3	31.4	50.1	2,327.17
ELF3-HFC Col rep1	| Symbols: PHYE | phytochrome E | chr4:10042312-10045948 REVERSE LENGTH=1112	AT4G18130.1	TAIR10_20101214.fasta	122,519.10	100.00%	25	27	36	0.42%	32.60%	SGDPALTLAGAVQSQK	99.70%	33	29.5	20.2	1,542.81
ELF3-HFC Col rep1	| Symbols: PHYE | phytochrome E | chr4:10042312-10045948 REVERSE LENGTH=1112	AT4G18130.1	TAIR10_20101214.fasta	122,519.10	100.00%	25	27	36	0.42%	32.60%	SLADEIVQEESR	99.70%	84.2	30	67.2	1,375.67
ELF3-HFC Col rep1	| Symbols: PHYE | phytochrome E | chr4:10042312-10045948 REVERSE LENGTH=1112	AT4G18130.1	TAIR10_20101214.fasta	122,519.10	100.00%	25	27	36	0.42%	32.60%	SLPWEISEIDAIHSLR	99.70%	87.1	29.9	83.2	1,865.98
ELF3-HFC Col rep1	| Symbols: PHYE | phytochrome E | chr4:10042312-10045948 REVERSE LENGTH=1112	AT4G18130.1	TAIR10_20101214.fasta	122,519.10	100.00%	25	27	36	0.42%	32.60%	SSFTAFLEVAK	99.70%	63.2	27.3	55.2	1,199.63
ELF3-HFC Col rep1	| Symbols: PHYE | phytochrome E | chr4:10042312-10045948 REVERSE LENGTH=1112	AT4G18130.1	TAIR10_20101214.fasta	122,519.10	100.00%	25	27	36	0.42%	32.60%	TLFTPSSGASLSK	99.70%	46.7	28.8	29.1	1,295.68
ELF3-HFC Col rep1	| Symbols: PHYE | phytochrome E | chr4:10042312-10045948 REVERSE LENGTH=1112	AT4G18130.1	TAIR10_20101214.fasta	122,519.10	100.00%	25	27	36	0.42%	32.60%	TQTLLCDMLLR	99.70%	37	30.3	23.6	1,379.70
ELF3-HFC Col rep1	| Symbols: PHYE | phytochrome E | chr4:10042312-10045948 REVERSE LENGTH=1112	AT4G18130.1	TAIR10_20101214.fasta	122,519.10	100.00%	25	27	36	0.42%	32.60%	TTQKPFYAILHR	99.70%	45.9	29.1	40.6	1,474.82
ELF3-HFC Col rep1	| Symbols: PHYE | phytochrome E | chr4:10042312-10045948 REVERSE LENGTH=1112	AT4G18130.1	TAIR10_20101214.fasta	122,519.10	100.00%	25	27	36	0.42%	32.60%	VEVAEEIK	98.50%	33.1	30.6	4.02	916.4986
ELF3-HFC Col rep1	| Symbols: PHYE | phytochrome E | chr4:10042312-10045948 REVERSE LENGTH=1112	AT4G18130.1	TAIR10_20101214.fasta	122,519.10	100.00%	25	27	36	0.42%	32.60%	YIEASLTANK	99.70%	61.9	28.5	48	1,109.58
ELF3-HFC Col rep1	| Symbols: PIF7 | phytochrome-interacting factor7 | chr5:24638873-24640439 REVERSE LENGTH=366	AT5G61270.1,AT5G61270.2	TAIR10_20101214.fasta	40,825.60	100.00%	2	3	3	0.03%	4.10%	ADKVSILDDVIEHLK	99.70%	54.7	27.1	47.2	1,694.93
ELF3-HFC Col rep1	| Symbols: PIF7 | phytochrome-interacting factor7 | chr5:24638873-24640439 REVERSE LENGTH=366	AT5G61270.1,AT5G61270.2	TAIR10_20101214.fasta	40,825.60	100.00%	2	3	3	0.03%	4.10%	VSILDDVIEHLK	99.70%	79.3	26.7	64.5	1,380.77
ELF3-HFC Col rep1	| Symbols: SPA1 | SPA (suppressor of phyA-105) protein family | chr2:19022572-19026821 REVERSE LENGTH=1029	AT2G46340.1	TAIR10_20101214.fasta	114,689.10	100.00%	5	5	6	0.07%	6.90%	AWSVDFSPSDPTK	99.70%	54.5	29.2	47.2	1,436.67
ELF3-HFC Col rep1	| Symbols: SPA1 | SPA (suppressor of phyA-105) protein family | chr2:19022572-19026821 REVERSE LENGTH=1029	AT2G46340.1	TAIR10_20101214.fasta	114,689.10	100.00%	5	5	6	0.07%	6.90%	LLQDIQTLEDDIKEAER	99.70%	35.8	30.1	23.3	2,029.05
ELF3-HFC Col rep1	| Symbols: SPA1 | SPA (suppressor of phyA-105) protein family | chr2:19022572-19026821 REVERSE LENGTH=1029	AT2G46340.1	TAIR10_20101214.fasta	114,689.10	100.00%	5	5	6	0.07%	6.90%	RRPVVEESSSGGR	99.70%	71.2	30.1	42.6	1,415.73
ELF3-HFC Col rep1	| Symbols: SPA1 | SPA (suppressor of phyA-105) protein family | chr2:19022572-19026821 REVERSE LENGTH=1029	AT2G46340.1	TAIR10_20101214.fasta	114,689.10	100.00%	5	5	6	0.07%	6.90%	TPVSSSNFSQLLLK	99.70%	112	28.1	99.3	1,520.83
ELF3-HFC Col rep1	| Symbols: SPA1 | SPA (suppressor of phyA-105) protein family | chr2:19022572-19026821 REVERSE LENGTH=1029	AT2G46340.1	TAIR10_20101214.fasta	114,689.10	100.00%	5	5	6	0.07%	6.90%	VAEETVATNNIQLK	99.70%	81.2	29.7	70.7	1,529.82
ELF3-HFC Col rep1	| Symbols: SPA2 | SPA1-related 2 | chr4:6772163-6776675 FORWARD LENGTH=1036	AT4G11110.1	TAIR10_20101214.fasta	115,007.50	100.00%	4	4	4	0.05%	4.92%	FGSIDPISGK	99.70%	36.5	29.5	29.5	1,020.54
ELF3-HFC Col rep1	| Symbols: SPA2 | SPA1-related 2 | chr4:6772163-6776675 FORWARD LENGTH=1036	AT4G11110.1	TAIR10_20101214.fasta	115,007.50	100.00%	4	4	4	0.05%	4.92%	LGDTSSLSIPAKK	99.40%	26.2	28.2	19.3	1,316.74
ELF3-HFC Col rep1	| Symbols: SPA2 | SPA1-related 2 | chr4:6772163-6776675 FORWARD LENGTH=1036	AT4G11110.1	TAIR10_20101214.fasta	115,007.50	100.00%	4	4	4	0.05%	4.92%	NPVPVEALSHEGIK	99.70%	56.8	30.1	53.7	1,489.80
ELF3-HFC Col rep1	| Symbols: SPA2 | SPA1-related 2 | chr4:6772163-6776675 FORWARD LENGTH=1036	AT4G11110.1	TAIR10_20101214.fasta	115,007.50	100.00%	4	4	4	0.05%	4.92%	NSEQSFKPENIEVR	96.00%	23.5	30.6	14.7	1,676.82
ELF3-HFC Col rep1	| Symbols: SPA3 | SPA1-related 3 | chr3:5169327-5172480 REVERSE LENGTH=837	AT3G15354.1	TAIR10_20101214.fasta	93,386.90	100.00%	3	3	3	0.03%	4.90%	LQDTVSLLSSDIEQVVK	99.70%	34.5	28.8	24.8	1,874.01
ELF3-HFC Col rep1	| Symbols: SPA3 | SPA1-related 3 | chr3:5169327-5172480 REVERSE LENGTH=837	AT3G15354.1	TAIR10_20101214.fasta	93,386.90	100.00%	3	3	3	0.03%	4.90%	SGEDDSLGIDPFVR	99.70%	42.3	29.4	23.7	1,506.71
ELF3-HFC Col rep1	| Symbols: SPA3 | SPA1-related 3 | chr3:5169327-5172480 REVERSE LENGTH=837	AT3G15354.1	TAIR10_20101214.fasta	93,386.90	100.00%	3	3	3	0.03%	4.90%	SLEWGDVSLR	97.00%	31.6	30.4	0.63	1,161.59
ELF3-HFC Col rep1	| Symbols: TIC | time for coffee | chr3:7913181-7918879 FORWARD LENGTH=1550	AT3G22380.1,AT3G22380.2	TAIR10_20101214.fasta	164,452.80	100.00%	10	11	13	0.15%	8.52%	LDLDKSDHVGLVNK	94.90%	24.4	28.5	14.2	1,552.83
ELF3-HFC Col rep1	| Symbols: TIC | time for coffee | chr3:7913181-7918879 FORWARD LENGTH=1550	AT3G22380.1,AT3G22380.2	TAIR10_20101214.fasta	164,452.80	100.00%	10	11	13	0.15%	8.52%	NIQSHQQFTK	99.70%	41.4	28.8	24.4	1,230.62
ELF3-HFC Col rep1	| Symbols: TIC | time for coffee | chr3:7913181-7918879 FORWARD LENGTH=1550	AT3G22380.1,AT3G22380.2	TAIR10_20101214.fasta	164,452.80	100.00%	10	11	13	0.15%	8.52%	QEAAGNDLTEAAK	99.70%	56.3	28.1	45.2	1,317.63
ELF3-HFC Col rep1	| Symbols: TIC | time for coffee | chr3:7913181-7918879 FORWARD LENGTH=1550	AT3G22380.1,AT3G22380.2	TAIR10_20101214.fasta	164,452.80	100.00%	10	11	13	0.15%	8.52%	RPHESWASSTTGGGVFASGEQIHR	99.70%	36	31.5	27.1	2,555.20
ELF3-HFC Col rep1	| Symbols: TIC | time for coffee | chr3:7913181-7918879 FORWARD LENGTH=1550	AT3G22380.1,AT3G22380.2	TAIR10_20101214.fasta	164,452.80	100.00%	10	11	13	0.15%	8.52%	SQQQQQLPK	99.70%	45.8	26.5	33.3	1,084.57
ELF3-HFC Col rep1	| Symbols: TIC | time for coffee | chr3:7913181-7918879 FORWARD LENGTH=1550	AT3G22380.1,AT3G22380.2	TAIR10_20101214.fasta	164,452.80	100.00%	10	11	13	0.15%	8.52%	SQVPFSNQLK	99.70%	24.9	30.6	22.1	1,147.61
ELF3-HFC Col rep1	| Symbols: TIC | time for coffee | chr3:7913181-7918879 FORWARD LENGTH=1550	AT3G22380.1,AT3G22380.2	TAIR10_20101214.fasta	164,452.80	100.00%	10	11	13	0.15%	8.52%	SSGSGEGNSSVLDSIIPLTR	99.70%	93.9	31.3	89.6	1,975.99
ELF3-HFC Col rep1	| Symbols: TIC | time for coffee | chr3:7913181-7918879 FORWARD LENGTH=1550	AT3G22380.1,AT3G22380.2	TAIR10_20101214.fasta	164,452.80	100.00%	10	11	13	0.15%	8.52%	SSPSPAPVSPLVSTWK	99.70%	56.5	28.4	46.1	1,639.87
ELF3-HFC Col rep1	| Symbols: TIC | time for coffee | chr3:7913181-7918879 FORWARD LENGTH=1550	AT3G22380.1,AT3G22380.2	TAIR10_20101214.fasta	164,452.80	100.00%	10	11	13	0.15%	8.52%	VTEVETEAKPLLK	99.70%	73.6	28.1	65.2	1,456.83
ELF3-HFC Col rep1	| Symbols: TIC | time for coffee | chr3:7913181-7918879 FORWARD LENGTH=1550	AT3G22380.1,AT3G22380.2	TAIR10_20101214.fasta	164,452.80	100.00%	10	11	13	0.15%	8.52%	VTEVETEAKPLLKEDR	99.40%	29.2	29.6	22.2	1,857.00
ELF3-HFC Col rep1	| Symbols: TOC1, APRR1, PRR1, AtTOC1 | CCT motif -containing response regulator protein | chr5:24675540-24678176 FORWARD LENGTH=618	AT5G61380.1	TAIR10_20101214.fasta	69,195.40	100.00%	6	7	11	0.13%	16.20%	FQVVASEGINNTK	99.70%	103	30.5	14.8	1,407.71
ELF3-HFC Col rep1	| Symbols: TOC1, APRR1, PRR1, AtTOC1 | CCT motif -containing response regulator protein | chr5:24675540-24678176 FORWARD LENGTH=618	AT5G61380.1	TAIR10_20101214.fasta	69,195.40	100.00%	6	7	11	0.13%	16.20%	GNLSHQENEWSVATAPVHAR	99.70%	49.2	31.1	35.3	2,203.06
ELF3-HFC Col rep1	| Symbols: TOC1, APRR1, PRR1, AtTOC1 | CCT motif -containing response regulator protein | chr5:24675540-24678176 FORWARD LENGTH=618	AT5G61380.1	TAIR10_20101214.fasta	69,195.40	100.00%	6	7	11	0.13%	16.20%	GTEQYHSQGETLQNGASYPHSLER	99.70%	40.1	29.7	0	2,690.21
ELF3-HFC Col rep1	| Symbols: TOC1, APRR1, PRR1, AtTOC1 | CCT motif -containing response regulator protein | chr5:24675540-24678176 FORWARD LENGTH=618	AT5G61380.1	TAIR10_20101214.fasta	69,195.40	100.00%	6	7	11	0.13%	16.20%	IGESSAFFTYVK	99.70%	83.7	30.8	75.8	1,348.68
ELF3-HFC Col rep1	| Symbols: TOC1, APRR1, PRR1, AtTOC1 | CCT motif -containing response regulator protein | chr5:24675540-24678176 FORWARD LENGTH=618	AT5G61380.1	TAIR10_20101214.fasta	69,195.40	100.00%	6	7	11	0.13%	16.20%	NSNPAQFSSAPK	99.70%	67.6	29.1	56.5	1,247.60
ELF3-HFC Col rep1	| Symbols: TOC1, APRR1, PRR1, AtTOC1 | CCT motif -containing response regulator protein | chr5:24675540-24678176 FORWARD LENGTH=618	AT5G61380.1	TAIR10_20101214.fasta	69,195.40	100.00%	6	7	11	0.13%	16.20%	TNGQDPPLVDGNGSLHLHR	99.70%	70.5	30.8	10.4	2,028.97
ELF3-HFC Col rep1	| Symbols: TZP | zinc knuckle (CCHC-type) family protein | chr5:17527261-17530207 FORWARD LENGTH=831	AT5G43630.1	TAIR10_20101214.fasta	90,615.60	100.00%	23	25	35	0.41%	32.90%	AVFDAVQVLR	99.70%	84.9	28.7	59.7	1,117.64
ELF3-HFC Col rep1	| Symbols: TZP | zinc knuckle (CCHC-type) family protein | chr5:17527261-17530207 FORWARD LENGTH=831	AT5G43630.1	TAIR10_20101214.fasta	90,615.60	100.00%	23	25	35	0.41%	32.90%	DCLEVTDTELR	99.70%	81.2	28.4	54.8	1,350.62
ELF3-HFC Col rep1	| Symbols: TZP | zinc knuckle (CCHC-type) family protein | chr5:17527261-17530207 FORWARD LENGTH=831	AT5G43630.1	TAIR10_20101214.fasta	90,615.60	100.00%	23	25	35	0.41%	32.90%	DLICFYCGK	99.70%	44.1	25	36.6	1,175.52
ELF3-HFC Col rep1	| Symbols: TZP | zinc knuckle (CCHC-type) family protein | chr5:17527261-17530207 FORWARD LENGTH=831	AT5G43630.1	TAIR10_20101214.fasta	90,615.60	100.00%	23	25	35	0.41%	32.90%	DLLVNEQLR	99.70%	47.6	26.3	30.4	1,099.61
ELF3-HFC Col rep1	| Symbols: TZP | zinc knuckle (CCHC-type) family protein | chr5:17527261-17530207 FORWARD LENGTH=831	AT5G43630.1	TAIR10_20101214.fasta	90,615.60	100.00%	23	25	35	0.41%	32.90%	DLVQNISVR	99.70%	59	29	44.1	1,043.58
ELF3-HFC Col rep1	| Symbols: TZP | zinc knuckle (CCHC-type) family protein | chr5:17527261-17530207 FORWARD LENGTH=831	AT5G43630.1	TAIR10_20101214.fasta	90,615.60	100.00%	23	25	35	0.41%	32.90%	FAAVDAITELVWSPSNGLSLR	99.60%	32.9	29.9	18.5	2,246.18
ELF3-HFC Col rep1	| Symbols: TZP | zinc knuckle (CCHC-type) family protein | chr5:17527261-17530207 FORWARD LENGTH=831	AT5G43630.1	TAIR10_20101214.fasta	90,615.60	100.00%	23	25	35	0.41%	32.90%	GDDLSSSGNEIGPVAEPNISSGK	99.70%	51.1	30.4	43.1	2,230.05
ELF3-HFC Col rep1	| Symbols: TZP | zinc knuckle (CCHC-type) family protein | chr5:17527261-17530207 FORWARD LENGTH=831	AT5G43630.1	TAIR10_20101214.fasta	90,615.60	100.00%	23	25	35	0.41%	32.90%	GDYLPEGEAGPSGSYR	99.70%	65.1	27.4	56.7	1,654.73
ELF3-HFC Col rep1	| Symbols: TZP | zinc knuckle (CCHC-type) family protein | chr5:17527261-17530207 FORWARD LENGTH=831	AT5G43630.1	TAIR10_20101214.fasta	90,615.60	100.00%	23	25	35	0.41%	32.90%	GVTCLVESQFISNQDFLEEELK	99.70%	73.8	31.6	62.8	2,585.24
ELF3-HFC Col rep1	| Symbols: TZP | zinc knuckle (CCHC-type) family protein | chr5:17527261-17530207 FORWARD LENGTH=831	AT5G43630.1	TAIR10_20101214.fasta	90,615.60	100.00%	23	25	35	0.41%	32.90%	IESSEAMASLFAR	99.70%	89.1	29	73	1,427.68
ELF3-HFC Col rep1	| Symbols: TZP | zinc knuckle (CCHC-type) family protein | chr5:17527261-17530207 FORWARD LENGTH=831	AT5G43630.1	TAIR10_20101214.fasta	90,615.60	100.00%	23	25	35	0.41%	32.90%	KRPGFEEQLIFGSK	99.70%	46.7	28.3	37.3	1,635.89
ELF3-HFC Col rep1	| Symbols: TZP | zinc knuckle (CCHC-type) family protein | chr5:17527261-17530207 FORWARD LENGTH=831	AT5G43630.1	TAIR10_20101214.fasta	90,615.60	100.00%	23	25	35	0.41%	32.90%	KSVSGLEGFFLR	99.70%	39.9	26.7	20.2	1,339.74
ELF3-HFC Col rep1	| Symbols: TZP | zinc knuckle (CCHC-type) family protein | chr5:17527261-17530207 FORWARD LENGTH=831	AT5G43630.1	TAIR10_20101214.fasta	90,615.60	100.00%	23	25	35	0.41%	32.90%	LGKWEEGLGGTGYYVAR	99.70%	37.8	30.3	28	1,855.93
ELF3-HFC Col rep1	| Symbols: TZP | zinc knuckle (CCHC-type) family protein | chr5:17527261-17530207 FORWARD LENGTH=831	AT5G43630.1	TAIR10_20101214.fasta	90,615.60	100.00%	23	25	35	0.41%	32.90%	LPISGFTDVPR	99.70%	92.8	29.4	79.6	1,201.66
ELF3-HFC Col rep1	| Symbols: TZP | zinc knuckle (CCHC-type) family protein | chr5:17527261-17530207 FORWARD LENGTH=831	AT5G43630.1	TAIR10_20101214.fasta	90,615.60	100.00%	23	25	35	0.41%	32.90%	NALASTSGMK	97.60%	23.8	27.8	15.1	995.4827
ELF3-HFC Col rep1	| Symbols: TZP | zinc knuckle (CCHC-type) family protein | chr5:17527261-17530207 FORWARD LENGTH=831	AT5G43630.1	TAIR10_20101214.fasta	90,615.60	100.00%	23	25	35	0.41%	32.90%	NALASTSGMKLPISGFTDVPR	99.70%	44	31.1	33.7	2,178.12
ELF3-HFC Col rep1	| Symbols: TZP | zinc knuckle (CCHC-type) family protein | chr5:17527261-17530207 FORWARD LENGTH=831	AT5G43630.1	TAIR10_20101214.fasta	90,615.60	100.00%	23	25	35	0.41%	32.90%	NGREEASSLCIR	99.30%	30.3	28.8	16.7	1,392.65
ELF3-HFC Col rep1	| Symbols: TZP | zinc knuckle (CCHC-type) family protein | chr5:17527261-17530207 FORWARD LENGTH=831	AT5G43630.1	TAIR10_20101214.fasta	90,615.60	100.00%	23	25	35	0.41%	32.90%	NTGFQSFFQSIYCPK	99.70%	86	29.4	67.4	1,823.84
ELF3-HFC Col rep1	| Symbols: TZP | zinc knuckle (CCHC-type) family protein | chr5:17527261-17530207 FORWARD LENGTH=831	AT5G43630.1	TAIR10_20101214.fasta	90,615.60	100.00%	23	25	35	0.41%	32.90%	NVIRPSISSVDGPDKPDTVLPIVSSMR	99.70%	31.3	29.2	20.1	2,895.52
ELF3-HFC Col rep1	| Symbols: TZP | zinc knuckle (CCHC-type) family protein | chr5:17527261-17530207 FORWARD LENGTH=831	AT5G43630.1	TAIR10_20101214.fasta	90,615.60	100.00%	23	25	35	0.41%	32.90%	RPGFEEQLIFGSK	99.70%	41.6	30.1	29.2	1,507.79
ELF3-HFC Col rep1	| Symbols: TZP | zinc knuckle (CCHC-type) family protein | chr5:17527261-17530207 FORWARD LENGTH=831	AT5G43630.1	TAIR10_20101214.fasta	90,615.60	100.00%	23	25	35	0.41%	32.90%	SVSGLEGFFLR	99.70%	56.3	27.8	40.2	1,211.64
ELF3-HFC Col rep1	| Symbols: TZP | zinc knuckle (CCHC-type) family protein | chr5:17527261-17530207 FORWARD LENGTH=831	AT5G43630.1	TAIR10_20101214.fasta	90,615.60	100.00%	23	25	35	0.41%	32.90%	TLNQECLGSTSK	99.70%	50.5	29.5	48.9	1,337.64
ELF3-HFC Col rep1	| Symbols: TZP | zinc knuckle (CCHC-type) family protein | chr5:17527261-17530207 FORWARD LENGTH=831	AT5G43630.1	TAIR10_20101214.fasta	90,615.60	100.00%	23	25	35	0.41%	32.90%	WEEGLGGTGYYVAR	99.70%	71.7	29.2	63	1,557.73
ELF3-HFC Col rep2	| Symbols: | BED zinc finger ;hAT family dimerisation domain | chr3:14321838-14323928 FORWARD LENGTH=696	AT3G42170.1	TAIR10_20101214.fasta	78,815.20	100.00%	2	2	2	0.01%	4.17%	SELDQYLDETLLPR	99.40%	39	30.9	33.3	1,691.85
ELF3-HFC Col rep2	| Symbols: | BED zinc finger ;hAT family dimerisation domain | chr3:14321838-14323928 FORWARD LENGTH=696	AT3G42170.1	TAIR10_20101214.fasta	78,815.20	100.00%	2	2	2	0.01%	4.17%	SQNNASPYVAFNQDK	99.60%	36.8	28.8	36.4	1,682.78
ELF3-HFC Col rep2	| Symbols: | Homeodomain-like superfamily protein | chr5:24004047-24004943 FORWARD LENGTH=298	AT5G59570.1	TAIR10_20101214.fasta	32,366.70	99.90%	1	1	6	0.03%	17.40%	ASQITLSSLR	99.70%	63.8	29	42.7	1,075.61
ELF3-HFC Col rep2	| Symbols: | Homeodomain-like superfamily protein | chr5:24004047-24004943 FORWARD LENGTH=298	AT5G59570.1	TAIR10_20101214.fasta	32,366.70	99.90%	1	1	6	0.03%	17.40%	ENVASHLQK	99.70%	37.5	27.7	23.6	1,025.54
ELF3-HFC Col rep2	| Symbols: | Homeodomain-like superfamily protein | chr5:24004047-24004943 FORWARD LENGTH=298	AT5G59570.1	TAIR10_20101214.fasta	32,366.70	99.90%	1	1	6	0.03%	17.40%	FVDVVAHLGIK	99.70%	59.9	25	47.7	1,197.70
ELF3-HFC Col rep2	| Symbols: | Homeodomain-like superfamily protein | chr5:24004047-24004943 FORWARD LENGTH=298	AT5G59570.1	TAIR10_20101214.fasta	32,366.70	99.90%	1	1	6	0.03%	17.40%	LVWTPQLHK	99.70%	35.1	25.5	26.7	1,121.65
ELF3-HFC Col rep2	| Symbols: | Homeodomain-like superfamily protein | chr5:24004047-24004943 FORWARD LENGTH=298	AT5G59570.1	TAIR10_20101214.fasta	32,366.70	99.90%	1	1	6	0.03%	17.40%	TIMQLMNVEGLTR	99.70%	87.6	30.8	73.8	1,505.78
ELF3-HFC Col rep2	| Symbols: | C2 calcium/lipid-binding plant phosphoribosyltransferase family protein | chr5:2127200-2129584 REVERSE LENGTH=794	AT5G06850.1	TAIR10_20101214.fasta	91,004.50	100.00%	3	3	3	0.02%	5.92%	LISPLSVFEK	99.70%	44.2	25.9	24.5	1,132.66
ELF3-HFC Col rep2	| Symbols: | C2 calcium/lipid-binding plant phosphoribosyltransferase family protein | chr5:2127200-2129584 REVERSE LENGTH=794	AT5G06850.1	TAIR10_20101214.fasta	91,004.50	100.00%	3	3	3	0.02%	5.92%	VNVIEAQDVEPSDR	99.70%	49.8	29.7	44.7	1,570.77
ELF3-HFC Col rep2	| Symbols: | C2 calcium/lipid-binding plant phosphoribosyltransferase family protein | chr5:2127200-2129584 REVERSE LENGTH=794	AT5G06850.1	TAIR10_20101214.fasta	91,004.50	100.00%	3	3	3	0.02%	5.92%	VSWAEAASPDELDEEFDTFPTSK	99.70%	35.2	28.9	35.2	2,571.14
ELF3-HFC Col rep2	| Symbols: | F-box family protein | chr2:7074940-7079181 FORWARD LENGTH=727	AT2G16365.4	TAIR10_20101214.fasta	82,716.20	100.00%	18	22	25	0.15%	30.00%	ALSGLNPYGLR	99.70%	63.9	30.1	47.7	1,160.64
ELF3-HFC Col rep2	| Symbols: | F-box family protein | chr2:7074940-7079181 FORWARD LENGTH=727	AT2G16365.4	TAIR10_20101214.fasta	82,716.20	100.00%	18	22	25	0.15%	30.00%	EDSHQLLK	98.90%	28	26.6	11.1	969.5001

ELF3-HFC Col rep2	| Symbols: | F-box family protein | chr2:7074940-7079181 FORWARD LENGTH=727	AT2G16365.4	TAIR10_20101214.fasta	82,716.20	100.00%	18	22	25	0.15%	30.00%	ENNNLFLEILK	99.70%	73.2	30.2	57.1	1,346.73
ELF3-HFC Col rep2	| Symbols: | F-box family protein | chr2:7074940-7079181 FORWARD LENGTH=727	AT2G16365.4	TAIR10_20101214.fasta	82,716.20	100.00%	18	22	25	0.15%	30.00%	ETTTTTTTKPSFHEDVGSSSR	99.70%	77.9	30.4	70.1	2,269.06
ELF3-HFC Col rep2	| Symbols: | F-box family protein | chr2:7074940-7079181 FORWARD LENGTH=727	AT2G16365.4	TAIR10_20101214.fasta	82,716.20	100.00%	18	22	25	0.15%	30.00%	IFGGYLDLFPNQDHSHNR	99.70%	45.3	30.5	34.3	2,130.02
ELF3-HFC Col rep2	| Symbols: | F-box family protein | chr2:7074940-7079181 FORWARD LENGTH=727	AT2G16365.4	TAIR10_20101214.fasta	82,716.20	100.00%	18	22	25	0.15%	30.00%	IYATVDSVNLPK	99.70%	55.6	29.5	41.3	1,319.72
ELF3-HFC Col rep2	| Symbols: | F-box family protein | chr2:7074940-7079181 FORWARD LENGTH=727	AT2G16365.4	TAIR10_20101214.fasta	82,716.20	100.00%	18	22	25	0.15%	30.00%	LDFPIQEK	99.00%	39.9	30.2	12.2	989.5304
ELF3-HFC Col rep2	| Symbols: | F-box family protein | chr2:7074940-7079181 FORWARD LENGTH=727	AT2G16365.4	TAIR10_20101214.fasta	82,716.20	100.00%	18	22	25	0.15%	30.00%	LEPEVEASSR	98.80%	29	27.7	20.2	1,116.55
ELF3-HFC Col rep2	| Symbols: | F-box family protein | chr2:7074940-7079181 FORWARD LENGTH=727	AT2G16365.4	TAIR10_20101214.fasta	82,716.20	100.00%	18	22	25	0.15%	30.00%	LQSLESSKDTQEDGPR	99.70%	81.1	29.9	68.7	1,789.86
ELF3-HFC Col rep2	| Symbols: | F-box family protein | chr2:7074940-7079181 FORWARD LENGTH=727	AT2G16365.4	TAIR10_20101214.fasta	82,716.20	100.00%	18	22	25	0.15%	30.00%	LQTIHLSGSISSSSTK	99.70%	47.8	30.5	43.9	1,645.88
ELF3-HFC Col rep2	| Symbols: | F-box family protein | chr2:7074940-7079181 FORWARD LENGTH=727	AT2G16365.4	TAIR10_20101214.fasta	82,716.20	100.00%	18	22	25	0.15%	30.00%	NSSVNPYESAWLGR	99.70%	95.1	30	82.7	1,579.75
ELF3-HFC Col rep2	| Symbols: | F-box family protein | chr2:7074940-7079181 FORWARD LENGTH=727	AT2G16365.4	TAIR10_20101214.fasta	82,716.20	100.00%	18	22	25	0.15%	30.00%	QGGDDITLLHPWIQR	99.40%	36.9	30.7	27.8	1,748.91
ELF3-HFC Col rep2	| Symbols: | F-box family protein | chr2:7074940-7079181 FORWARD LENGTH=727	AT2G16365.4	TAIR10_20101214.fasta	82,716.20	100.00%	18	22	25	0.15%	30.00%	SGINNLQPR	99.70%	37.5	25.4	22.7	998.5378
ELF3-HFC Col rep2	| Symbols: | F-box family protein | chr2:7074940-7079181 FORWARD LENGTH=727	AT2G16365.4	TAIR10_20101214.fasta	82,716.20	100.00%	18	22	25	0.15%	30.00%	TTQNLLELIRPVR	99.70%	54.7	25	48.9	1,552.92
ELF3-HFC Col rep2	| Symbols: | F-box family protein | chr2:7074940-7079181 FORWARD LENGTH=727	AT2G16365.4	TAIR10_20101214.fasta	82,716.20	100.00%	18	22	25	0.15%	30.00%	TTSTDQPTGQEASFEPESHKEFEK	99.70%	57	29	48	2,710.21
ELF3-HFC Col rep2	| Symbols: | F-box family protein | chr2:7074940-7079181 FORWARD LENGTH=727	AT2G16365.4	TAIR10_20101214.fasta	82,716.20	100.00%	18	22	25	0.15%	30.00%	TTSTDQPTGQEASFEPESHKEFEKK	98.80%	30.4	30.9	18.8	2,838.31
ELF3-HFC Col rep2	| Symbols: | F-box family protein | chr2:7074940-7079181 FORWARD LENGTH=727	AT2G16365.4	TAIR10_20101214.fasta	82,716.20	100.00%	18	22	25	0.15%	30.00%	VRLEPEVEASSR	99.70%	58.7	29.1	52.9	1,371.72
ELF3-HFC Col rep2	| Symbols: | F-box family protein | chr2:7074940-7079181 FORWARD LENGTH=727	AT2G16365.4	TAIR10_20101214.fasta	82,716.20	100.00%	18	22	25	0.15%	30.00%	WTQSGSEVK	95.20%	28.2	29.4	15.8	1,021.49
ELF3-HFC Col rep2	| Symbols: | Protein kinase family protein | chr2:10985118-10988652 REVERSE LENGTH=673	AT2G25760.1,AT2G25760.2	TAIR10_20101214.fasta	76,182.30	99.90%	1	1	3	0.02%	6.39%	LTDETQETLR	99.70%	44.5	30.2	0	1,205.60
ELF3-HFC Col rep2	| Symbols: | Protein kinase family protein | chr2:10985118-10988652 REVERSE LENGTH=673	AT2G25760.1,AT2G25760.2	TAIR10_20101214.fasta	76,182.30	99.90%	1	1	3	0.02%	6.39%	VSDSFPFK	98.90%	28.3	26.8	18.5	926.4618
ELF3-HFC Col rep2	| Symbols: | Protein kinase family protein | chr2:10985118-10988652 REVERSE LENGTH=673	AT2G25760.1,AT2G25760.2	TAIR10_20101214.fasta	76,182.30	99.90%	1	1	3	0.02%	6.39%	YVSLFDGIVGPNPDIRPINTEGAQK	99.70%	31.8	30.2	20.8	2,700.40
ELF3-HFC Col rep2	| Symbols: | Protein kinase family protein | chr3:1014412-1018244 REVERSE LENGTH=701	AT3G03940.1	TAIR10_20101214.fasta	77,995.40	100.00%	1	1	6	0.03%	10.60%	GFVHGDVKPENFLLGQPGTADEK	99.70%	47.4	31.4	44	2,455.23
ELF3-HFC Col rep2	| Symbols: | Protein kinase family protein | chr3:1014412-1018244 REVERSE LENGTH=701	AT3G03940.1	TAIR10_20101214.fasta	77,995.40	100.00%	1	1	6	0.03%	10.60%	IGADAIEVALK	99.70%	77.2	27.5	63	1,099.64
ELF3-HFC Col rep2	| Symbols: | Protein kinase family protein | chr3:1014412-1018244 REVERSE LENGTH=701	AT3G03940.1	TAIR10_20101214.fasta	77,995.40	100.00%	1	1	6	0.03%	10.60%	ITDETQETLR	99.70%	44.5	30.2	0	1,205.60
ELF3-HFC Col rep2	| Symbols: | Protein kinase family protein | chr3:1014412-1018244 REVERSE LENGTH=701	AT3G03940.1	TAIR10_20101214.fasta	77,995.40	100.00%	1	1	6	0.03%	10.60%	LLINLEEDEQPR	99.70%	59.9	29.1	50.9	1,468.76
ELF3-HFC Col rep2	| Symbols: | Protein kinase family protein | chr3:1014412-1018244 REVERSE LENGTH=701	AT3G03940.1	TAIR10_20101214.fasta	77,995.40	100.00%	1	1	6	0.03%	10.60%	VQVGNSPVYK	99.70%	38	30.4	29	1,090.59
ELF3-HFC Col rep2	| Symbols: | Protein kinase family protein | chr3:1014412-1018244 REVERSE LENGTH=701	AT3G03940.1	TAIR10_20101214.fasta	77,995.40	100.00%	1	1	6	0.03%	10.60%	VSDSFPFK	98.90%	28.3	26.8	18.5	926.4618
ELF3-HFC Col rep2	| Symbols: | Protein kinase family protein | chr3:4469434-4473234 FORWARD LENGTH=703	AT3G13670.1	TAIR10_20101214.fasta	78,640.00	100.00%	1	1	2	0.01%	3.27%	LGVPATQWISIYNAR	99.60%	41.4	29.5	41.2	1,688.91
ELF3-HFC Col rep2	| Symbols: | Protein kinase family protein | chr3:4469434-4473234 FORWARD LENGTH=703	AT3G13670.1	TAIR10_20101214.fasta	78,640.00	100.00%	1	1	2	0.01%	3.27%	VSDSFPFK	98.90%	28.3	26.8	18.5	926.4618
ELF3-HFC Col rep2	| Symbols: | Protein kinase family protein | chr5:6010215-6013724 REVERSE LENGTH=691	AT5G18190.1	TAIR10_20101214.fasta	76,985.50	99.90%	1	1	5	0.03%	9.84%	GFVHGDVKPENFLLGQPGTADEK	99.70%	47.4	31.4	44	2,455.23
ELF3-HFC Col rep2	| Symbols: | Protein kinase family protein | chr5:6010215-6013724 REVERSE LENGTH=691	AT5G18190.1	TAIR10_20101214.fasta	76,985.50	99.90%	1	1	5	0.03%	9.84%	IGADAIEVALK	99.70%	77.2	27.5	63	1,099.64
ELF3-HFC Col rep2	| Symbols: | Protein kinase family protein | chr5:6010215-6013724 REVERSE LENGTH=691	AT5G18190.1	TAIR10_20101214.fasta	76,985.50	99.90%	1	1	5	0.03%	9.84%	VQVGNSPVYK	99.70%	38	30.4	29	1,090.59
ELF3-HFC Col rep2	| Symbols: | Protein kinase family protein | chr5:6010215-6013724 REVERSE LENGTH=691	AT5G18190.1	TAIR10_20101214.fasta	76,985.50	99.90%	1	1	5	0.03%	9.84%	VSDSFPFK	98.90%	28.3	26.8	18.5	926.4618
ELF3-HFC Col rep2	| Symbols: | Protein kinase family protein | chr5:6010215-6013724 REVERSE LENGTH=691	AT5G18190.1	TAIR10_20101214.fasta	76,985.50	99.90%	1	1	5	0.03%	9.84%	VVGVEEDSSTAPVPER	98.90%	37.7	30.1	32.4	1,670.82
ELF3-HFC Col rep2	| Symbols: ATAN11, LWD1 | Transducin/WD40 repeat-like superfamily protein | chr1:4395114-4396154 REVERSE LENGTH=346	AT1G12910.1	TAIR10_20101214.fasta	39,089.30	100.00%	2	2	2	0.01%	10.70%	DKEHSTIIYESSEPDTPLVR	98.10%	32.2	31.8	25.2	2,316.14
ELF3-HFC Col rep2	| Symbols: ATAN11, LWD1 | Transducin/WD40 repeat-like superfamily protein | chr1:4395114-4396154 REVERSE LENGTH=346	AT1G12910.1	TAIR10_20101214.fasta	39,089.30	100.00%	2	2	2	0.01%	10.70%	IGTSSTDTTCTIWDIER	99.70%	85.2	29.8	83.1	1,955.90
ELF3-HFC Col rep2	| Symbols: COP1, ATCOP1, DET340, FUS1, EMB168 | Transducin/WD40 repeat-like superfamily protein | chr2:13978000-13983282 FORWARD LENGTH=675	AT2G32950.1	TAIR10_20101214.fasta	76,188.00	100.00%	8	9	9	0.05%	18.50%	AWSVDFSR	99.00%	31.3	27.7	18	967.4631
ELF3-HFC Col rep2	| Symbols: COP1, ATCOP1, DET340, FUS1, EMB168 | Transducin/WD40 repeat-like superfamily protein | chr2:13978000-13983282 FORWARD LENGTH=675	AT2G32950.1	TAIR10_20101214.fasta	76,188.00	100.00%	8	9	9	0.05%	18.50%	EGYSNGLADFQSVLTTFTR	99.70%	85.5	30.7	24.9	2,107.00
ELF3-HFC Col rep2	| Symbols: COP1, ATCOP1, DET340, FUS1, EMB168 | Transducin/WD40 repeat-like superfamily protein | chr2:13978000-13983282 FORWARD LENGTH=675	AT2G32950.1	TAIR10_20101214.fasta	76,188.00	100.00%	8	9	9	0.05%	18.50%	EVDNLLTLLAER	99.70%	44	28.1	30.6	1,385.76
ELF3-HFC Col rep2	| Symbols: COP1, ATCOP1, DET340, FUS1, EMB168 | Transducin/WD40 repeat-like superfamily protein | chr2:13978000-13983282 FORWARD LENGTH=675	AT2G32950.1	TAIR10_20101214.fasta	76,188.00	100.00%	8	9	9	0.05%	18.50%	FLSNNELASASTDSTLR	99.70%	111	30.6	101	1,825.89
ELF3-HFC Col rep2	| Symbols: COP1, ATCOP1, DET340, FUS1, EMB168 | Transducin/WD40 repeat-like superfamily protein | chr2:13978000-13983282 FORWARD LENGTH=675	AT2G32950.1	TAIR10_20101214.fasta	76,188.00	100.00%	8	9	9	0.05%	18.50%	GGNFVGNYQNK	99.70%	34.6	28.8	25.4	1,197.56
ELF3-HFC Col rep2	| Symbols: COP1, ATCOP1, DET340, FUS1, EMB168 | Transducin/WD40 repeat-like superfamily protein | chr2:13978000-13983282 FORWARD LENGTH=675	AT2G32950.1	TAIR10_20101214.fasta	76,188.00	100.00%	8	9	9	0.05%	18.50%	NHIASSDYEGIVTVWDVTTR	99.70%	45.9	31.1	35.2	2,263.10
ELF3-HFC Col rep2	| Symbols: COP1, ATCOP1, DET340, FUS1, EMB168 | Transducin/WD40 repeat-like superfamily protein | chr2:13978000-13983282 FORWARD LENGTH=675	AT2G32950.1	TAIR10_20101214.fasta	76,188.00	100.00%	8	9	9	0.05%	18.50%	SDCPCCSQHLTNNQLYPNFLLDK	99.70%	46.6	28.2	5.46	2,825.23
ELF3-HFC Col rep2	| Symbols: COP1, ATCOP1, DET340, FUS1, EMB168 | Transducin/WD40 repeat-like superfamily protein | chr2:13978000-13983282 FORWARD LENGTH=675	AT2G32950.1	TAIR10_20101214.fasta	76,188.00	100.00%	8	9	9	0.05%	18.50%	VDELNEVQTDLQYIK	99.70%	69.9	31.2	56.5	1,806.91
ELF3-HFC Col rep2	| Symbols: ELF3, PYK20 | hydroxyproline-rich glycoprotein family protein | chr2:11059459-11063178 FORWARD LENGTH=695	AT2G25930.1	TAIR10_20101214.fasta	77,204.80	100.00%	22	37	42	0.24%	41.90%	EEEDFAVPVYINSR	99.70%	68.4	30	55.6	1,667.79
ELF3-HFC Col rep2	| Symbols: ELF3, PYK20 | hydroxyproline-rich glycoprotein family protein | chr2:11059459-11063178 FORWARD LENGTH=695	AT2G25930.1	TAIR10_20101214.fasta	77,204.80	100.00%	22	37	42	0.24%	41.90%	FQEVNQTGSK	99.70%	60.1	29.3	41.9	1,137.55
ELF3-HFC Col rep2	| Symbols: ELF3, PYK20 | hydroxyproline-rich glycoprotein family protein | chr2:11059459-11063178 FORWARD LENGTH=695	AT2G25930.1	TAIR10_20101214.fasta	77,204.80	100.00%	22	37	42	0.24%	41.90%	IFQSIQEER	99.70%	52.3	30.1	27	1,149.59
ELF3-HFC Col rep2	| Symbols: ELF3, PYK20 | hydroxyproline-rich glycoprotein family protein | chr2:11059459-11063178 FORWARD LENGTH=695	AT2G25930.1	TAIR10_20101214.fasta	77,204.80	100.00%	22	37	42	0.24%	41.90%	KAIANQQR	99.00%	42.8	25.8	13.8	928.5323
ELF3-HFC Col rep2	| Symbols: ELF3, PYK20 | hydroxyproline-rich glycoprotein family protein | chr2:11059459-11063178 FORWARD LENGTH=695	AT2G25930.1	TAIR10_20101214.fasta	77,204.80	100.00%	22	37	42	0.24%	41.90%	KLLPSEFLVKPPLPHVVVK	98.90%	24.9	25	24.9	2,140.33
ELF3-HFC Col rep2	| Symbols: ELF3, PYK20 | hydroxyproline-rich glycoprotein family protein | chr2:11059459-11063178 FORWARD LENGTH=695	AT2G25930.1	TAIR10_20101214.fasta	77,204.80	100.00%	22	37	42	0.24%	41.90%	LASENAAR	99.00%	50.5	29.1	33.9	831.4318
ELF3-HFC Col rep2	| Symbols: ELF3, PYK20 | hydroxyproline-rich glycoprotein family protein | chr2:11059459-11063178 FORWARD LENGTH=695	AT2G25930.1	TAIR10_20101214.fasta	77,204.80	100.00%	22	37	42	0.24%	41.90%	LHVNDADK	99.00%	39.8	27.4	26.2	911.4582
ELF3-HFC Col rep2	| Symbols: ELF3, PYK20 | hydroxyproline-rich glycoprotein family protein | chr2:11059459-11063178 FORWARD LENGTH=695	AT2G25930.1	TAIR10_20101214.fasta	77,204.80	100.00%	22	37	42	0.24%	41.90%	LHVNDADKGGPR	99.70%	46.3	30.3	46.3	1,278.66
ELF3-HFC Col rep2	| Symbols: ELF3, PYK20 | hydroxyproline-rich glycoprotein family protein | chr2:11059459-11063178 FORWARD LENGTH=695	AT2G25930.1	TAIR10_20101214.fasta	77,204.80	100.00%	22	37	42	0.24%	41.90%	LIAASPDLLLDEISFLGK	99.70%	102	25.9	91.3	1,915.08
ELF3-HFC Col rep2	| Symbols: ELF3, PYK20 | hydroxyproline-rich glycoprotein family protein | chr2:11059459-11063178 FORWARD LENGTH=695	AT2G25930.1	TAIR10_20101214.fasta	77,204.80	100.00%	22	37	42	0.24%	41.90%	LKDTDNGAESHLATENHSQEGHGSPEDIDNDR	99.70%	44.3	27.7	38.5	3,488.52
ELF3-HFC Col rep2	| Symbols: ELF3, PYK20 | hydroxyproline-rich glycoprotein family protein | chr2:11059459-11063178 FORWARD LENGTH=695	AT2G25930.1	TAIR10_20101214.fasta	77,204.80	100.00%	22	37	42	0.24%	41.90%	LKDTDNGAESHLATENHSQEGHGSPEDIDNDREYSK	99.00%	36.6	28.2	26	3,995.75
ELF3-HFC Col rep2	| Symbols: ELF3, PYK20 | hydroxyproline-rich glycoprotein family protein | chr2:11059459-11063178 FORWARD LENGTH=695	AT2G25930.1	TAIR10_20101214.fasta	77,204.80	100.00%	22	37	42	0.24%	41.90%	LLPSEFLVKPPLPHVVVK	99.70%	31.4	25	29.4	2,012.23
ELF3-HFC Col rep2	| Symbols: ELF3, PYK20 | hydroxyproline-rich glycoprotein family protein | chr2:11059459-11063178 FORWARD LENGTH=695	AT2G25930.1	TAIR10_20101214.fasta	77,204.80	100.00%	22	37	42	0.24%	41.90%	MALYEQLSIPSQR	99.70%	79.4	31.2	69.4	1,535.79
ELF3-HFC Col rep2	| Symbols: ELF3, PYK20 | hydroxyproline-rich glycoprotein family protein | chr2:11059459-11063178 FORWARD LENGTH=695	AT2G25930.1	TAIR10_20101214.fasta	77,204.80	100.00%	22	37	42	0.24%	41.90%	NLSVQHLDSSAANQATEK	99.70%	106	31	99.2	1,912.94
ELF3-HFC Col rep2	| Symbols: ELF3, PYK20 | hydroxyproline-rich glycoprotein family protein | chr2:11059459-11063178 FORWARD LENGTH=695	AT2G25930.1	TAIR10_20101214.fasta	77,204.80	100.00%	22	37	42	0.24%	41.90%	QGSTGSSPSGPQGISGSK	99.70%	95.3	29	89.3	1,618.77
ELF3-HFC Col rep2	| Symbols: ELF3, PYK20 | hydroxyproline-rich glycoprotein family protein | chr2:11059459-11063178 FORWARD LENGTH=695	AT2G25930.1	TAIR10_20101214.fasta	77,204.80	100.00%	22	37	42	0.24%	41.90%	QNVCLATCSKPEVR	99.70%	64.9	30.2	54.6	1,661.81
ELF3-HFC Col rep2	| Symbols: ELF3, PYK20 | hydroxyproline-rich glycoprotein family protein | chr2:11059459-11063178 FORWARD LENGTH=695	AT2G25930.1	TAIR10_20101214.fasta	77,204.80	100.00%	22	37	42	0.24%	41.90%	SDNEPAPQQQQQPTK	99.70%	61.9	29.8	47.2	1,695.79
ELF3-HFC Col rep2	| Symbols: ELF3, PYK20 | hydroxyproline-rich glycoprotein family protein | chr2:11059459-11063178 FORWARD LENGTH=695	AT2G25930.1	TAIR10_20101214.fasta	77,204.80	100.00%	22	37	42	0.24%	41.90%	SFRPFAAVDEDSNINNAPEQTMTTTTTTTR	99.70%	88.4	30.9	83.2	3,332.53
ELF3-HFC Col rep2	| Symbols: ELF3, PYK20 | hydroxyproline-rich glycoprotein family protein | chr2:11059459-11063178 FORWARD LENGTH=695	AT2G25930.1	TAIR10_20101214.fasta	77,204.80	100.00%	22	37	42	0.24%	41.90%	SNNTSTLVHPGPSSQPCGVER	99.40%	32.3	30.5	25.8	2,224.04
ELF3-HFC Col rep2	| Symbols: ELF3, PYK20 | hydroxyproline-rich glycoprotein family protein | chr2:11059459-11063178 FORWARD LENGTH=695	AT2G25930.1	TAIR10_20101214.fasta	77,204.80	100.00%	22	37	42	0.24%	41.90%	TTVTQTTR	99.00%	35.2	28.4	24.9	907.4844
ELF3-HFC Col rep2	| Symbols: ELF3, PYK20 | hydroxyproline-rich glycoprotein family protein | chr2:11059459-11063178 FORWARD LENGTH=695	AT2G25930.1	TAIR10_20101214.fasta	77,204.80	100.00%	22	37	42	0.24%	41.90%	VFAVQLFELHR	99.70%	46.7	28.5	34	1,358.76
ELF3-HFC Col rep2	| Symbols: ELF3, PYK20 | hydroxyproline-rich glycoprotein family protein | chr2:11059459-11063178 FORWARD LENGTH=695	AT2G25930.1	TAIR10_20101214.fasta	77,204.80	100.00%	22	37	42	0.24%	41.90%	VNDYNASLR	99.70%	70.2	28.9	55	1,051.52
ELF3-HFC Col rep2	| Symbols: ELF4 | Protein of unknown function (DUF1313) | chr2:16734545-16734880 REVERSE LENGTH=111	AT2G40080.1	TAIR10_20101214.fasta	12,375.40	100.00%	4	10	13	0.08%	60.40%	NVAEEAEQGEDPAMWENLDR	99.70%	69.7	25.4	69.7	2,318.98
ELF3-HFC Col rep2	| Symbols: ELF4 | Protein of unknown function (DUF1313) | chr2:16734545-16734880 REVERSE LENGTH=111	AT2G40080.1	TAIR10_20101214.fasta	12,375.40	100.00%	4	10	13	0.08%	60.40%	NVALIQELNGNISK	99.70%	118	27.5	104	1,512.84
ELF3-HFC Col rep2	| Symbols: ELF4 | Protein of unknown function (DUF1313) | chr2:16734545-16734880 REVERSE LENGTH=111	AT2G40080.1	TAIR10_20101214.fasta	12,375.40	100.00%	4	10	13	0.08%	60.40%	SLIQQVNDNHQSR	99.70%	66.8	30.2	16.2	1,539.75
ELF3-HFC Col rep2	| Symbols: ELF4 | Protein of unknown function (DUF1313) | chr2:16734545-16734880 REVERSE LENGTH=111	AT2G40080.1	TAIR10_20101214.fasta	12,375.40	100.00%	4	10	13	0.08%	60.40%	VVNMYSDLNTSFSSGFHGGK	99.70%	61.1	29	49.1	2,162.98
ELF3-HFC Col rep2	| Symbols: PCL1, LUX | Homeodomain-like superfamily protein | chr3:17183248-17184219 FORWARD LENGTH=323	AT3G46640.1,AT3G46640.2	TAIR10_20101214.fasta	34,648.50	100.00%	3	4	10	0.06%	28.20%	ENVASHLQK	99.70%	37.5	27.7	23.6	1,025.54
ELF3-HFC Col rep2	| Symbols: PCL1, LUX | Homeodomain-like superfamily protein | chr3:17183248-17184219 FORWARD LENGTH=323	AT3G46640.1,AT3G46640.2	TAIR10_20101214.fasta	34,648.50	100.00%	3	4	10	0.06%	28.20%	FGSMASYPSVGGGSANEN	99.70%	34.3	25	26.3	1,747.72
ELF3-HFC Col rep2	| Symbols: PCL1, LUX | Homeodomain-like superfamily protein | chr3:17183248-17184219 FORWARD LENGTH=323	AT3G46640.1,AT3G46640.2	TAIR10_20101214.fasta	34,648.50	100.00%	3	4	10	0.06%	28.20%	FVDVVAHLGIK	99.70%	59.9	25	47.7	1,197.70
ELF3-HFC Col rep2	| Symbols: PCL1, LUX | Homeodomain-like superfamily protein | chr3:17183248-17184219 FORWARD LENGTH=323	AT3G46640.1,AT3G46640.2	TAIR10_20101214.fasta	34,648.50	100.00%	3	4	10	0.06%	28.20%	GGSSGPNTSSSNNNVEEEDR	99.70%	92	25	87.3	2,036.84
ELF3-HFC Col rep2	| Symbols: PCL1, LUX | Homeodomain-like superfamily protein | chr3:17183248-17184219 FORWARD LENGTH=323	AT3G46640.1,AT3G46640.2	TAIR10_20101214.fasta	34,648.50	100.00%	3	4	10	0.06%	28.20%	GGSSGPNTSSSNNNVEEEDRVGSSSPGSDSK	99.70%	82.7	25.9	1.36	3,027.25
ELF3-HFC Col rep2	| Symbols: PCL1, LUX | Homeodomain-like superfamily protein | chr3:17183248-17184219 FORWARD LENGTH=323	AT3G46640.1,AT3G46640.2	TAIR10_20101214.fasta	34,648.50	100.00%	3	4	10	0.06%	28.20%	LVWTPQLHK	99.70%	35.1	25.5	26.7	1,121.65
ELF3-HFC Col rep2	| Symbols: PCL1, LUX | Homeodomain-like superfamily protein | chr3:17183248-17184219 FORWARD LENGTH=323	AT3G46640.1,AT3G46640.2	TAIR10_20101214.fasta	34,648.50	100.00%	3	4	10	0.06%	28.20%	TIMQLMNVEGLTR	99.70%	87.6	30.8	73.8	1,505.78
ELF3-HFC Col rep2	| Symbols: PHYA, FHY2, FRE1, HY8 | phytochrome A | chr1:3095498-3099216 REVERSE LENGTH=1122	AT1G09570.1	TAIR10_20101214.fasta	124,503.20	100.00%	7	7	8	0.05%	11.50%	ALGFGDVSLLNPILVHCR	99.70%	40.8	28.3	34.8	1,981.07
ELF3-HFC Col rep2	| Symbols: PHYA, FHY2, FRE1, HY8 | phytochrome A | chr1:3095498-3099216 REVERSE LENGTH=1122	AT1G09570.1	TAIR10_20101214.fasta	124,503.20	100.00%	7	7	8	0.05%	11.50%	FHEDDHGEVVSEVTKPGLEPYLGLHYPATDIPQAAR	99.70%	46	31.6	29.2	3,987.95
ELF3-HFC Col rep2	| Symbols: PHYA, FHY2, FRE1, HY8 | phytochrome A | chr1:3095498-3099216 REVERSE LENGTH=1122	AT1G09570.1	TAIR10_20101214.fasta	124,503.20	100.00%	7	7	8	0.05%	11.50%	IAELTGLSVDEAIGK	99.70%	84	29.5	73.6	1,515.83
ELF3-HFC Col rep2	| Symbols: PHYA, FHY2, FRE1, HY8 | phytochrome A | chr1:3095498-3099216 REVERSE LENGTH=1122	AT1G09570.1	TAIR10_20101214.fasta	124,503.20	100.00%	7	7	8	0.05%	11.50%	IIAQTTVDAK	99.70%	53	29	35.8	1,059.60
ELF3-HFC Col rep2	| Symbols: PHYA, FHY2, FRE1, HY8 | phytochrome A | chr1:3095498-3099216 REVERSE LENGTH=1122	AT1G09570.1	TAIR10_20101214.fasta	124,503.20	100.00%	7	7	8	0.05%	11.50%	LKNQEAFVNLGIVLNNAVTSQDPEK	99.70%	41.2	29.8	37	2,741.45
ELF3-HFC Col rep2	| Symbols: PHYA, FHY2, FRE1, HY8 | phytochrome A | chr1:3095498-3099216 REVERSE LENGTH=1122	AT1G09570.1	TAIR10_20101214.fasta	124,503.20	100.00%	7	7	8	0.05%	11.50%	SLFTAPSASALQK	99.70%	70.3	29.3	60.5	1,320.72
ELF3-HFC Col rep2	| Symbols: PHYA, FHY2, FRE1, HY8 | phytochrome A | chr1:3095498-3099216 REVERSE LENGTH=1122	AT1G09570.1	TAIR10_20101214.fasta	124,503.20	100.00%	7	7	8	0.05%	11.50%	VTGPVVENQPPR	99.70%	53.2	29.7	46.2	1,292.70
ELF3-HFC Col rep2	| Symbols: PHYB, HY3, OOP1 | phytochrome B | chr2:8140079-8144151 FORWARD LENGTH=1172	AT2G18790.1	TAIR10_20101214.fasta	129,333.90	100.00%	35	49	74	0.43%	50.90%	AIQQYTVDAR	99.70%	67.8	29.9	56.1	1,164.60
ELF3-HFC Col rep2	| Symbols: PHYB, HY3, OOP1 | phytochrome B | chr2:8140079-8144151 FORWARD LENGTH=1172	AT2G18790.1	TAIR10_20101214.fasta	129,333.90	100.00%	35	49	74	0.43%	50.90%	AISQLQALPGGDIK	99.70%	108	26.1	93.6	1,410.80
ELF3-HFC Col rep2	| Symbols: PHYB, HY3, OOP1 | phytochrome B | chr2:8140079-8144151 FORWARD LENGTH=1172	AT2G18790.1	TAIR10_20101214.fasta	129,333.90	100.00%	35	49	74	0.43%	50.90%	ALRGDEEKNVEVK	99.70%	46.9	29.8	30.5	1,486.79
ELF3-HFC Col rep2	| Symbols: PHYB, HY3, OOP1 | phytochrome B | chr2:8140079-8144151 FORWARD LENGTH=1172	AT2G18790.1	TAIR10_20101214.fasta	129,333.90	100.00%	35	49	74	0.43%	50.90%	AVFVVVNACSSK	99.70%	56.2	29.6	47.1	1,280.67
ELF3-HFC Col rep2	| Symbols: PHYB, HY3, OOP1 | phytochrome B | chr2:8140079-8144151 FORWARD LENGTH=1172	AT2G18790.1	TAIR10_20101214.fasta	129,333.90	100.00%	35	49	74	0.43%	50.90%	CDGAAFLYHGK	99.70%	55.3	27.1	31.2	1,238.56
ELF3-HFC Col rep2	| Symbols: PHYB, HY3, OOP1 | phytochrome B | chr2:8140079-8144151 FORWARD LENGTH=1172	AT2G18790.1	TAIR10_20101214.fasta	129,333.90	100.00%	35	49	74	0.43%	50.90%	DDLEPYIGLHYPATDIPQASR	99.70%	44.7	31.3	0	2,371.16
ELF3-HFC Col rep2	| Symbols: PHYB, HY3, OOP1 | phytochrome B | chr2:8140079-8144151 FORWARD LENGTH=1172	AT2G18790.1	TAIR10_20101214.fasta	129,333.90	100.00%	35	49	74	0.43%	50.90%	DMAGEQGIDELGAVAR	99.70%	118	28.6	106	1,647.76
ELF3-HFC Col rep2	| Symbols: PHYB, HY3, OOP1 | phytochrome B | chr2:8140079-8144151 FORWARD LENGTH=1172	AT2G18790.1	TAIR10_20101214.fasta	129,333.90	100.00%	35	49	74	0.43%	50.90%	DSPAGIVTQSPSIMDLVK	99.70%	84.1	30.4	80.6	1,857.96
ELF3-HFC Col rep2	| Symbols: PHYB, HY3, OOP1 | phytochrome B | chr2:8140079-8144151 FORWARD LENGTH=1172	AT2G18790.1	TAIR10_20101214.fasta	129,333.90	100.00%	35	49	74	0.43%	50.90%	DYLNNIVGVCFVGQDVTSQK	99.70%	78.9	31.5	75.1	2,256.10
ELF3-HFC Col rep2	| Symbols: PHYB, HY3, OOP1 | phytochrome B | chr2:8140079-8144151 FORWARD LENGTH=1172	AT2G18790.1	TAIR10_20101214.fasta	129,333.90	100.00%	35	49	74	0.43%	50.90%	EITLLNPVWIHSK	99.70%	67.1	26.7	51	1,549.87
ELF3-HFC Col rep2	| Symbols: PHYB, HY3, OOP1 | phytochrome B | chr2:8140079-8144151 FORWARD LENGTH=1172	AT2G18790.1	TAIR10_20101214.fasta	129,333.90	100.00%	35	49	74	0.43%	50.90%	ELAYICQVIK	99.70%	57.2	28.6	40.9	1,236.67
ELF3-HFC Col rep2	| Symbols: PHYB, HY3, OOP1 | phytochrome B | chr2:8140079-8144151 FORWARD LENGTH=1172	AT2G18790.1	TAIR10_20101214.fasta	129,333.90	100.00%	35	49	74	0.43%	50.90%	FANSLLEATDLNEDQK	99.70%	118	30.5	72	1,808.86
ELF3-HFC Col rep2	| Symbols: PHYB, HY3, OOP1 | phytochrome B | chr2:8140079-8144151 FORWARD LENGTH=1172	AT2G18790.1	TAIR10_20101214.fasta	129,333.90	100.00%	35	49	74	0.43%	50.90%	FHEDEHGEVVAESK	99.70%	101	27.2	96.4	1,612.72
ELF3-HFC Col rep2	| Symbols: PHYB, HY3, OOP1 | phytochrome B | chr2:8140079-8144151 FORWARD LENGTH=1172	AT2G18790.1	TAIR10_20101214.fasta	129,333.90	100.00%	35	49	74	0.43%	50.90%	FINIQGDYK	99.70%	50.5	27.9	35.1	1,097.56
ELF3-HFC Col rep2	| Symbols: PHYB, HY3, OOP1 | phytochrome B | chr2:8140079-8144151 FORWARD LENGTH=1172	AT2G18790.1	TAIR10_20101214.fasta	129,333.90	100.00%	35	49	74	0.43%	50.90%	FMIVLHNAIGGQDTDKFPFPFFDR	98.20%	41.2	31.8	30	2,828.39
ELF3-HFC Col rep2	| Symbols: PHYB, HY3, OOP1 | phytochrome B | chr2:8140079-8144151 FORWARD LENGTH=1172	AT2G18790.1	TAIR10_20101214.fasta	129,333.90	100.00%	35	49	74	0.43%	50.90%	FVQALLTANK	99.70%	61.1	26.3	36.9	1,104.64
ELF3-HFC Col rep2	| Symbols: PHYB, HY3, OOP1 | phytochrome B | chr2:8140079-8144151 FORWARD LENGTH=1172	AT2G18790.1	TAIR10_20101214.fasta	129,333.90	100.00%	35	49	74	0.43%	50.90%	FVQALLTANKR	99.70%	45.8	25	28.3	1,260.74
ELF3-HFC Col rep2	| Symbols: PHYB, HY3, OOP1 | phytochrome B | chr2:8140079-8144151 FORWARD LENGTH=1172	AT2G18790.1	TAIR10_20101214.fasta	129,333.90	100.00%	35	49	74	0.43%	50.90%	IDVGVVIDLEPAR	99.70%	48.5	26.8	36.8	1,395.78
ELF3-HFC Col rep2	| Symbols: PHYB, HY3, OOP1 | phytochrome B | chr2:8140079-8144151 FORWARD LENGTH=1172	AT2G18790.1	TAIR10_20101214.fasta	129,333.90	100.00%	35	49	74	0.43%	50.90%	IIGYSENAR	96.10%	29.9	29.5	16.4	1,022.53
ELF3-HFC Col rep2	| Symbols: PHYB, HY3, OOP1 | phytochrome B | chr2:8140079-8144151 FORWARD LENGTH=1172	AT2G18790.1	TAIR10_20101214.fasta	129,333.90	100.00%	35	49	74	0.43%	50.90%	IQQLLAEFLLSIIR	99.70%	122	25	122	1,657.00
ELF3-HFC Col rep2	| Symbols: PHYB, HY3, OOP1 | phytochrome B | chr2:8140079-8144151 FORWARD LENGTH=1172	AT2G18790.1	TAIR10_20101214.fasta	129,333.90	100.00%	35	49	74	0.43%	50.90%	IVGDMDLESIEDGSFVLKR	95.00%	26.4	31.4	17.6	2,139.06
ELF3-HFC Col rep2	| Symbols: PHYB, HY3, OOP1 | phytochrome B | chr2:8140079-8144151 FORWARD LENGTH=1172	AT2G18790.1	TAIR10_20101214.fasta	129,333.90	100.00%	35	49	74	0.43%	50.90%	LHAVFEQSGESGK	99.70%	53.4	29.2	48.2	1,388.68
ELF3-HFC Col rep2	| Symbols: PHYB, HY3, OOP1 | phytochrome B | chr2:8140079-8144151 FORWARD LENGTH=1172	AT2G18790.1	TAIR10_20101214.fasta	129,333.90	100.00%	35	49	74	0.43%	50.90%	LIETATVPIFAVDAGGCINGWNAK	99.70%	61.1	31.2	57.2	2,517.28
ELF3-HFC Col rep2	| Symbols: PHYB, HY3, OOP1 | phytochrome B | chr2:8140079-8144151 FORWARD LENGTH=1172	AT2G18790.1	TAIR10_20101214.fasta	129,333.90	100.00%	35	49	74	0.43%	50.90%	LLCDTVVESVR	99.70%	59.5	30.6	39.6	1,290.67
ELF3-HFC Col rep2	| Symbols: PHYB, HY3, OOP1 | phytochrome B | chr2:8140079-8144151 FORWARD LENGTH=1172	AT2G18790.1	TAIR10_20101214.fasta	129,333.90	100.00%	35	49	74	0.43%	50.90%	LTQSMCLVGSTLR	99.70%	50	29.8	42.4	1,481.75
ELF3-HFC Col rep2	| Symbols: PHYB, HY3, OOP1 | phytochrome B | chr2:8140079-8144151 FORWARD LENGTH=1172	AT2G18790.1	TAIR10_20101214.fasta	129,333.90	100.00%	35	49	74	0.43%	50.90%	MIVDCNATPVLVVQDDR	99.70%	69.7	30.4	60.4	1,944.95
ELF3-HFC Col rep2	| Symbols: PHYB, HY3, OOP1 | phytochrome B | chr2:8140079-8144151 FORWARD LENGTH=1172	AT2G18790.1	TAIR10_20101214.fasta	129,333.90	100.00%	35	49	74	0.43%	50.90%	NTGKPFYAILHR	99.70%	70.1	29.6	61.1	1,416.77
ELF3-HFC Col rep2	| Symbols: PHYB, HY3, OOP1 | phytochrome B | chr2:8140079-8144151 FORWARD LENGTH=1172	AT2G18790.1	TAIR10_20101214.fasta	129,333.90	100.00%	35	49	74	0.43%	50.90%	QDTECFTK	99.00%	37.9	25	33	1,028.44
ELF3-HFC Col rep2	| Symbols: PHYB, HY3, OOP1 | phytochrome B | chr2:8140079-8144151 FORWARD LENGTH=1172	AT2G18790.1	TAIR10_20101214.fasta	129,333.90	100.00%	35	49	74	0.43%	50.90%	QLLETSVSCEK	99.70%	61.9	30.4	43.2	1,293.64
ELF3-HFC Col rep2	| Symbols: PHYB, HY3, OOP1 | phytochrome B | chr2:8140079-8144151 FORWARD LENGTH=1172	AT2G18790.1	TAIR10_20101214.fasta	129,333.90	100.00%	35	49	74	0.43%	50.90%	RDDLEPYIGLHYPATDIPQASR	99.70%	61.8	31.4	0	2,527.26
ELF3-HFC Col rep2	| Symbols: PHYB, HY3, OOP1 | phytochrome B | chr2:8140079-8144151 FORWARD LENGTH=1172	AT2G18790.1	TAIR10_20101214.fasta	129,333.90	100.00%	35	49	74	0.43%	50.90%	RQDTECFTK	99.70%	33.6	26.1	12.1	1,184.54
ELF3-HFC Col rep2	| Symbols: PHYB, HY3, OOP1 | phytochrome B | chr2:8140079-8144151 FORWARD LENGTH=1172	AT2G18790.1	TAIR10_20101214.fasta	129,333.90	100.00%	35	49	74	0.43%	50.90%	SFDYSQSLK	99.70%	51.1	26.6	38.6	1,074.51
ELF3-HFC Col rep2	| Symbols: PHYB, HY3, OOP1 | phytochrome B | chr2:8140079-8144151 FORWARD LENGTH=1172	AT2G18790.1	TAIR10_20101214.fasta	129,333.90	100.00%	35	49	74	0.43%	50.90%	SIEVFGDQIR	99.70%	58.7	30.5	40.2	1,163.61
ELF3-HFC Col rep2	| Symbols: PHYB, HY3, OOP1 | phytochrome B | chr2:8140079-8144151 FORWARD LENGTH=1172	AT2G18790.1	TAIR10_20101214.fasta	129,333.90	100.00%	35	49	74	0.43%	50.90%	SLFTSSSSILLER	99.70%	79.7	28.2	73.3	1,439.77
ELF3-HFC Col rep2	| Symbols: PHYB, HY3, OOP1 | phytochrome B | chr2:8140079-8144151 FORWARD LENGTH=1172	AT2G18790.1	TAIR10_20101214.fasta	129,333.90	100.00%	35	49	74	0.43%	50.90%	SLVSDLIYK	99.70%	53.7	26.3	42.7	1,037.59
ELF3-HFC Col rep2	| Symbols: PHYB, HY3, OOP1 | phytochrome B | chr2:8140079-8144151 FORWARD LENGTH=1172	AT2G18790.1	TAIR10_20101214.fasta	129,333.90	100.00%	35	49	74	0.43%	50.90%	SLVSDLIYKENEATVNK	99.70%	87.9	30.2	64.9	1,923.01
ELF3-HFC Col rep2	| Symbols: PHYB, HY3, OOP1 | phytochrome B | chr2:8140079-8144151 FORWARD LENGTH=1172	AT2G18790.1	TAIR10_20101214.fasta	129,333.90	100.00%	35	49	74	0.43%	50.90%	SQPWETAEMDAIHSLQLILR	97.10%	29.9	30.9	16.9	2,338.19
ELF3-HFC Col rep2	| Symbols: PHYB, HY3, OOP1 | phytochrome B | chr2:8140079-8144151 FORWARD LENGTH=1172	AT2G18790.1	TAIR10_20101214.fasta	129,333.90	100.00%	35	49	74	0.43%	50.90%	SSFQAFLEVVK	99.70%	63.3	27.3	50.1	1,254.67
ELF3-HFC Col rep2	| Symbols: PHYB, HY3, OOP1 | phytochrome B | chr2:8140079-8144151 FORWARD LENGTH=1172	AT2G18790.1	TAIR10_20101214.fasta	129,333.90	100.00%	35	49	74	0.43%	50.90%	TEDPALSIAGAVQSQK	99.70%	100	29.5	80.2	1,614.83
ELF3-HFC Col rep2	| Symbols: PHYB, HY3, OOP1 | phytochrome B | chr2:8140079-8144151 FORWARD LENGTH=1172	AT2G18790.1	TAIR10_20101214.fasta	129,333.90	100.00%	35	49	74	0.43%	50.90%	TFSPELQGK	99.70%	40.5	30	31.9	1,006.52
ELF3-HFC Col rep2	| Symbols: PHYB, HY3, OOP1 | phytochrome B | chr2:8140079-8144151 FORWARD LENGTH=1172	AT2G18790.1	TAIR10_20101214.fasta	129,333.90	100.00%	35	49	74	0.43%	50.90%	TTTYGSSVPEQQITAYLSR	99.70%	82	31	80	2,102.04
ELF3-HFC Col rep2	| Symbols: PHYB, HY3, OOP1 | phytochrome B | chr2:8140079-8144151 FORWARD LENGTH=1172	AT2G18790.1	TAIR10_20101214.fasta	129,333.90	100.00%	35	49	74	0.43%	50.90%	VIGAFCFLQIPSPELQQALAVQR	99.70%	52.3	29.1	2.45	2,586.38
ELF3-HFC Col rep2	| Symbols: PHYB, HY3, OOP1 | phytochrome B | chr2:8140079-8144151 FORWARD LENGTH=1172	AT2G18790.1	TAIR10_20101214.fasta	129,333.90	100.00%	35	49	74	0.43%	50.90%	VSGVGGSGGGR	99.70%	49.6	28.6	29.8	889.4487
ELF3-HFC Col rep2	| Symbols: PHYB, HY3, OOP1 | phytochrome B | chr2:8140079-8144151 FORWARD LENGTH=1172	AT2G18790.1	TAIR10_20101214.fasta	129,333.90	100.00%	35	49	74	0.43%	50.90%	VVDGVVQPCR	99.70%	45.5	27.4	39.6	1,128.58
ELF3-HFC Col rep2	| Symbols: PHYB, HY3, OOP1 | phytochrome B | chr2:8140079-8144151 FORWARD LENGTH=1172	AT2G18790.1	TAIR10_20101214.fasta	129,333.90	100.00%	35	49	74	0.43%	50.90%	YAPSQEWVEIHLSQLSK	99.70%	63.2	30.6	45.3	2,015.02
ELF3-HFC Col rep2	| Symbols: PHYB, HY3, OOP1 | phytochrome B | chr2:8140079-8144151 FORWARD LENGTH=1172	AT2G18790.1	TAIR10_20101214.fasta	129,333.90	100.00%	35	49	74	0.43%	50.90%	YYPLGVAPSEVQIK	99.70%	83.7	29.6	73.8	1,563.84
ELF3-HFC Col rep2	| Symbols: PHYC | phytochrome C | chr5:14008049-14011619 FORWARD LENGTH=1111	AT5G35840.1	TAIR10_20101214.fasta	123,724.60	100.00%	35	50	54	0.31%	43.80%	AAEVTGLAVEQAIGKPVSDLVEDDSVETVK	99.70%	92.8	30.6	91.7	3,069.58
ELF3-HFC Col rep2	| Symbols: PHYC | phytochrome C | chr5:14008049-14011619 FORWARD LENGTH=1111	AT5G35840.1	TAIR10_20101214.fasta	123,724.60	100.00%	35	50	54	0.31%	43.80%	AISFLQDLLHSSGLSEDQK	99.70%	78.7	30.8	69.4	2,088.06
ELF3-HFC Col rep2	| Symbols: PHYC | phytochrome C | chr5:14008049-14011619 FORWARD LENGTH=1111	AT5G35840.1	TAIR10_20101214.fasta	123,724.60	100.00%	35	50	54	0.31%	43.80%	AISFLQDLLHSSGLSEDQKR	99.70%	64	30.4	54.1	2,244.16
ELF3-HFC Col rep2	| Symbols: PHYC | phytochrome C | chr5:14008049-14011619 FORWARD LENGTH=1111	AT5G35840.1	TAIR10_20101214.fasta	123,724.60	100.00%	35	50	54	0.31%	43.80%	AVDFGEISILNPITLHCR	99.70%	110	30	104	2,055.07
ELF3-HFC Col rep2	| Symbols: PHYC | phytochrome C | chr5:14008049-14011619 FORWARD LENGTH=1111	AT5G35840.1	TAIR10_20101214.fasta	123,724.60	100.00%	35	50	54	0.31%	43.80%	DGSFIEALLSANK	99.70%	100	29.7	79.9	1,364.71
ELF3-HFC Col rep2	| Symbols: PHYC | phytochrome C | chr5:14008049-14011619 FORWARD LENGTH=1111	AT5G35840.1	TAIR10_20101214.fasta	123,724.60	100.00%	35	50	54	0.31%	43.80%	DGSFIEALLSANKR	99.70%	76.3	29.9	60.2	1,520.81
ELF3-HFC Col rep2	| Symbols: PHYC | phytochrome C | chr5:14008049-14011619 FORWARD LENGTH=1111	AT5G35840.1	TAIR10_20101214.fasta	123,724.60	100.00%	35	50	54	0.31%	43.80%	DLIDWVLK	98.60%	39.8	28.4	13.3	1,001.57
ELF3-HFC Col rep2	| Symbols: PHYC | phytochrome C | chr5:14008049-14011619 FORWARD LENGTH=1111	AT5G35840.1	TAIR10_20101214.fasta	123,724.60	100.00%	35	50	54	0.31%	43.80%	DMTNNVLGVCFIGQDVTGQK	99.20%	30.7	30.6	21.5	2,212.04
ELF3-HFC Col rep2	| Symbols: PHYC | phytochrome C | chr5:14008049-14011619 FORWARD LENGTH=1111	AT5G35840.1	TAIR10_20101214.fasta	123,724.60	100.00%	35	50	54	0.31%	43.80%	DNLWSLGVTPTETQIR	99.30%	33.4	30.6	28.5	1,829.94
ELF3-HFC Col rep2	| Symbols: PHYC | phytochrome C | chr5:14008049-14011619 FORWARD LENGTH=1111	AT5G35840.1	TAIR10_20101214.fasta	123,724.60	100.00%	35	50	54	0.31%	43.80%	EAESAVLLK	99.70%	52	29.4	30.9	959.5408
ELF3-HFC Col rep2	| Symbols: PHYC | phytochrome C | chr5:14008049-14011619 FORWARD LENGTH=1111	AT5G35840.1	TAIR10_20101214.fasta	123,724.60	100.00%	35	50	54	0.31%	43.80%	EALTIGTDVK	99.70%	38.9	29.8	28	1,046.57
ELF3-HFC Col rep2	| Symbols: PHYC | phytochrome C | chr5:14008049-14011619 FORWARD LENGTH=1111	AT5G35840.1	TAIR10_20101214.fasta	123,724.60	100.00%	35	50	54	0.31%	43.80%	EDMEPYLGLHYSATDIPQASR	99.70%	39.8	30.7	33.8	2,393.11
ELF3-HFC Col rep2	| Symbols: PHYC | phytochrome C | chr5:14008049-14011619 FORWARD LENGTH=1111	AT5G35840.1	TAIR10_20101214.fasta	123,724.60	100.00%	35	50	54	0.31%	43.80%	EGLGLHITQK	99.70%	45	25.9	34.3	1,095.62
ELF3-HFC Col rep2	| Symbols: PHYC | phytochrome C | chr5:14008049-14011619 FORWARD LENGTH=1111	AT5G35840.1	TAIR10_20101214.fasta	123,724.60	100.00%	35	50	54	0.31%	43.80%	EVSELTGYDR	99.70%	54.3	27.5	38.1	1,168.55
ELF3-HFC Col rep2	| Symbols: PHYC | phytochrome C | chr5:14008049-14011619 FORWARD LENGTH=1111	AT5G35840.1	TAIR10_20101214.fasta	123,724.60	100.00%	35	50	54	0.31%	43.80%	FHEDGHGEVIAECCR	99.70%	73.1	25	68.2	1,815.75
ELF3-HFC Col rep2	| Symbols: PHYC | phytochrome C | chr5:14008049-14011619 FORWARD LENGTH=1111	AT5G35840.1	TAIR10_20101214.fasta	123,724.60	100.00%	35	50	54	0.31%	43.80%	GMLIQPFGCLIVVDEK	99.70%	43.8	30.7	30	1,834.94
ELF3-HFC Col rep2	| Symbols: PHYC | phytochrome C | chr5:14008049-14011619 FORWARD LENGTH=1111	AT5G35840.1	TAIR10_20101214.fasta	123,724.60	100.00%	35	50	54	0.31%	43.80%	IEEGLVIDLEPVSPDEVPVTAAGALR	99.70%	52.9	28.4	50.1	2,689.43
ELF3-HFC Col rep2	| Symbols: PHYC | phytochrome C | chr5:14008049-14011619 FORWARD LENGTH=1111	AT5G35840.1	TAIR10_20101214.fasta	123,724.60	100.00%	35	50	54	0.31%	43.80%	IGFNAVISGQK	99.70%	69.8	28.6	51.1	1,133.63
ELF3-HFC Col rep2	| Symbols: PHYC | phytochrome C | chr5:14008049-14011619 FORWARD LENGTH=1111	AT5G35840.1	TAIR10_20101214.fasta	123,724.60	100.00%	35	50	54	0.31%	43.80%	IIHPAPGLPEDLVR	99.70%	62.4	26.1	54.4	1,526.87
ELF3-HFC Col rep2	| Symbols: PHYC | phytochrome C | chr5:14008049-14011619 FORWARD LENGTH=1111	AT5G35840.1	TAIR10_20101214.fasta	123,724.60	100.00%	35	50	54	0.31%	43.80%	ILLGEVFTTDDYGCCLK	99.70%	75.8	30.1	74.5	2,003.95
ELF3-HFC Col rep2	| Symbols: PHYC | phytochrome C | chr5:14008049-14011619 FORWARD LENGTH=1111	AT5G35840.1	TAIR10_20101214.fasta	123,724.60	100.00%	35	50	54	0.31%	43.80%	ILLGEVFTTDDYGCCLKDHDTLTK	99.40%	33.3	31.3	29.7	2,814.33
ELF3-HFC Col rep2	| Symbols: PHYC | phytochrome C | chr5:14008049-14011619 FORWARD LENGTH=1111	AT5G35840.1	TAIR10_20101214.fasta	123,724.60	100.00%	35	50	54	0.31%	43.80%	LHGNFEESER	99.70%	46.6	25.8	31	1,217.55
ELF3-HFC Col rep2	| Symbols: PHYC | phytochrome C | chr5:14008049-14011619 FORWARD LENGTH=1111	AT5G35840.1	TAIR10_20101214.fasta	123,724.60	100.00%	35	50	54	0.31%	43.80%	LIDTAAVPIFAVDASGVINGWNSK	99.70%	69.3	29.9	67.3	2,458.30
ELF3-HFC Col rep2	| Symbols: PHYC | phytochrome C | chr5:14008049-14011619 FORWARD LENGTH=1111	AT5G35840.1	TAIR10_20101214.fasta	123,724.60	100.00%	35	50	54	0.31%	43.80%	LLFGFYHR	99.00%	46	27.3	34.5	1,052.57
ELF3-HFC Col rep2	| Symbols: PHYC | phytochrome C | chr5:14008049-14011619 FORWARD LENGTH=1111	AT5G35840.1	TAIR10_20101214.fasta	123,724.60	100.00%	35	50	54	0.31%	43.80%	LQQILSETLLSSIR	99.70%	123	26	116	1,600.93
ELF3-HFC Col rep2	| Symbols: PHYC | phytochrome C | chr5:14008049-14011619 FORWARD LENGTH=1111	AT5G35840.1	TAIR10_20101214.fasta	123,724.60	100.00%	35	50	54	0.31%	43.80%	NAPIGIVTQSPNIMDLVK	99.70%	56.9	29	53.8	1,926.04
ELF3-HFC Col rep2	| Symbols: PHYC | phytochrome C | chr5:14008049-14011619 FORWARD LENGTH=1111	AT5G35840.1	TAIR10_20101214.fasta	123,724.60	100.00%	35	50	54	0.31%	43.80%	NMLALALEGSEER	99.70%	66.8	30.1	59.9	1,432.71

ELF3-HFC Col rep2	| Symbols: PHYC | phytochrome C | chr5:14008049-14011619 FORWARD LENGTH=1111	AT5G35840.1	TAIR10_20101214.fasta	123,724.60	100.00%	35	50	54	0.31%	43.80%	SLFLSPGCSALEK	99.70%	54.2	30.2	35.8	1,408.71
ELF3-HFC Col rep2	| Symbols: PHYC | phytochrome C | chr5:14008049-14011619 FORWARD LENGTH=1111	AT5G35840.1	TAIR10_20101214.fasta	123,724.60	100.00%	35	50	54	0.31%	43.80%	SLSQPISLSGSTLR	99.70%	111	29.1	91.9	1,445.80
ELF3-HFC Col rep2	| Symbols: PHYC | phytochrome C | chr5:14008049-14011619 FORWARD LENGTH=1111	AT5G35840.1	TAIR10_20101214.fasta	123,724.60	100.00%	35	50	54	0.31%	43.80%	SSPVELVVNTCCSR	99.70%	78.5	29.4	70.7	1,607.75
ELF3-HFC Col rep2	| Symbols: PHYC | phytochrome C | chr5:14008049-14011619 FORWARD LENGTH=1111	AT5G35840.1	TAIR10_20101214.fasta	123,724.60	100.00%	35	50	54	0.31%	43.80%	SSSKPFYAILHR	99.70%	53.2	29.5	44.5	1,405.76
ELF3-HFC Col rep2	| Symbols: PHYC | phytochrome C | chr5:14008049-14011619 FORWARD LENGTH=1111	AT5G35840.1	TAIR10_20101214.fasta	123,724.60	100.00%	35	50	54	0.31%	43.80%	SVPWDDMEMDAINSLQLIIK	99.70%	72.9	31.6	61.7	2,318.14
ELF3-HFC Col rep2	| Symbols: PHYC | phytochrome C | chr5:14008049-14011619 FORWARD LENGTH=1111	AT5G35840.1	TAIR10_20101214.fasta	123,724.60	100.00%	35	50	54	0.31%	43.80%	TLTENYSR	99.00%	47	27.8	22.4	983.4792
ELF3-HFC Col rep2	| Symbols: PHYC | phytochrome C | chr5:14008049-14011619 FORWARD LENGTH=1111	AT5G35840.1	TAIR10_20101214.fasta	123,724.60	100.00%	35	50	54	0.31%	43.80%	TVVDVPLVDNR	99.70%	44	26.4	36.3	1,226.67
ELF3-HFC Col rep2	| Symbols: PHYC | phytochrome C | chr5:14008049-14011619 FORWARD LENGTH=1111	AT5G35840.1	TAIR10_20101214.fasta	123,724.60	100.00%	35	50	54	0.31%	43.80%	YACEFLTQVFGVQINK	99.70%	90.5	30.6	79.2	1,916.96
ELF3-HFC Col rep2	| Symbols: PHYD | phytochrome D | chr4:9195602-9199486 REVERSE LENGTH=1164	AT4G16250.1	TAIR10_20101214.fasta	129,270.30	100.00%	1	1	18	0.10%	12.90%	AIQQYTVDAR	99.70%	67.8	29.9	56.1	1,164.60
ELF3-HFC Col rep2	| Symbols: PHYD | phytochrome D | chr4:9195602-9199486 REVERSE LENGTH=1164	AT4G16250.1	TAIR10_20101214.fasta	129,270.30	100.00%	1	1	18	0.10%	12.90%	FHEDEHGEVVAESK	99.70%	101	27.2	96.4	1,612.72
ELF3-HFC Col rep2	| Symbols: PHYD | phytochrome D | chr4:9195602-9199486 REVERSE LENGTH=1164	AT4G16250.1	TAIR10_20101214.fasta	129,270.30	100.00%	1	1	18	0.10%	12.90%	FINIQGDYK	99.70%	50.5	27.9	35.1	1,097.56
ELF3-HFC Col rep2	| Symbols: PHYD | phytochrome D | chr4:9195602-9199486 REVERSE LENGTH=1164	AT4G16250.1	TAIR10_20101214.fasta	129,270.30	100.00%	1	1	18	0.10%	12.90%	FMIVLHNAIGGQDTDKFPFPFFDR	98.20%	41.2	31.8	30	2,828.39
ELF3-HFC Col rep2	| Symbols: PHYD | phytochrome D | chr4:9195602-9199486 REVERSE LENGTH=1164	AT4G16250.1	TAIR10_20101214.fasta	129,270.30	100.00%	1	1	18	0.10%	12.90%	LHAVFEQSGESGK	99.70%	53.4	29.2	48.2	1,388.68
ELF3-HFC Col rep2	| Symbols: PHYD | phytochrome D | chr4:9195602-9199486 REVERSE LENGTH=1164	AT4G16250.1	TAIR10_20101214.fasta	129,270.30	100.00%	1	1	18	0.10%	12.90%	LLCDTVVESVR	99.70%	59.5	30.6	39.6	1,290.67
ELF3-HFC Col rep2	| Symbols: PHYD | phytochrome D | chr4:9195602-9199486 REVERSE LENGTH=1164	AT4G16250.1	TAIR10_20101214.fasta	129,270.30	100.00%	1	1	18	0.10%	12.90%	NDLEPYIGLHYPATDIPQASR	99.70%	44.7	31.3	0	2,371.16
ELF3-HFC Col rep2	| Symbols: PHYD | phytochrome D | chr4:9195602-9199486 REVERSE LENGTH=1164	AT4G16250.1	TAIR10_20101214.fasta	129,270.30	100.00%	1	1	18	0.10%	12.90%	QLLETSVSCEK	99.70%	61.9	30.4	43.2	1,293.64
ELF3-HFC Col rep2	| Symbols: PHYD | phytochrome D | chr4:9195602-9199486 REVERSE LENGTH=1164	AT4G16250.1	TAIR10_20101214.fasta	129,270.30	100.00%	1	1	18	0.10%	12.90%	RNDLEPYIGLHYPATDIPQASR	99.70%	61.8	31.4	0	2,527.26
ELF3-HFC Col rep2	| Symbols: PHYD | phytochrome D | chr4:9195602-9199486 REVERSE LENGTH=1164	AT4G16250.1	TAIR10_20101214.fasta	129,270.30	100.00%	1	1	18	0.10%	12.90%	SFDYSQSLK	99.70%	51.1	26.6	38.6	1,074.51
ELF3-HFC Col rep2	| Symbols: PHYD | phytochrome D | chr4:9195602-9199486 REVERSE LENGTH=1164	AT4G16250.1	TAIR10_20101214.fasta	129,270.30	100.00%	1	1	18	0.10%	12.90%	SSFQTFLEVVK	99.70%	44.9	27.8	25.7	1,284.68
ELF3-HFC Col rep2	| Symbols: PHYD | phytochrome D | chr4:9195602-9199486 REVERSE LENGTH=1164	AT4G16250.1	TAIR10_20101214.fasta	129,270.30	100.00%	1	1	18	0.10%	12.90%	TEDPALSIAGAVQSQK	99.70%	100	29.5	80.2	1,614.83
ELF3-HFC Col rep2	| Symbols: PHYE | phytochrome E | chr4:10042312-10045948 REVERSE LENGTH=1112	AT4G18130.1	TAIR10_20101214.fasta	122,519.10	100.00%	18	19	20	0.12%	22.80%	AALESLLCK	99.70%	55.9	30.1	45.4	1,004.54
ELF3-HFC Col rep2	| Symbols: PHYE | phytochrome E | chr4:10042312-10045948 REVERSE LENGTH=1112	AT4G18130.1	TAIR10_20101214.fasta	122,519.10	100.00%	18	19	20	0.12%	22.80%	AASFTEISLLNPVLVHSR	99.70%	57.8	27.1	48.6	1,954.08
ELF3-HFC Col rep2	| Symbols: PHYE | phytochrome E | chr4:10042312-10045948 REVERSE LENGTH=1112	AT4G18130.1	TAIR10_20101214.fasta	122,519.10	100.00%	18	19	20	0.12%	22.80%	DGWVTPDGLGLK	99.60%	30	27.7	11.9	1,257.65
ELF3-HFC Col rep2	| Symbols: PHYE | phytochrome E | chr4:10042312-10045948 REVERSE LENGTH=1112	AT4G18130.1	TAIR10_20101214.fasta	122,519.10	100.00%	18	19	20	0.12%	22.80%	ESAQSLNELTYVR	99.70%	80.4	30.5	67.6	1,509.75
ELF3-HFC Col rep2	| Symbols: PHYE | phytochrome E | chr4:10042312-10045948 REVERSE LENGTH=1112	AT4G18130.1	TAIR10_20101214.fasta	122,519.10	100.00%	18	19	20	0.12%	22.80%	ESFTSSRPVLSGNGVAR	99.70%	45.7	31.5	35.9	1,764.89
ELF3-HFC Col rep2	| Symbols: PHYE | phytochrome E | chr4:10042312-10045948 REVERSE LENGTH=1112	AT4G18130.1	TAIR10_20101214.fasta	122,519.10	100.00%	18	19	20	0.12%	22.80%	ILGLSDNSSDFLGLLSLPSTSHSGEFDK	99.70%	85.5	31.9	75	2,936.45
ELF3-HFC Col rep2	| Symbols: PHYE | phytochrome E | chr4:10042312-10045948 REVERSE LENGTH=1112	AT4G18130.1	TAIR10_20101214.fasta	122,519.10	100.00%	18	19	20	0.12%	22.80%	LLESSEISASQR	99.70%	79.2	30.7	61.5	1,319.68
ELF3-HFC Col rep2	| Symbols: PHYE | phytochrome E | chr4:10042312-10045948 REVERSE LENGTH=1112	AT4G18130.1	TAIR10_20101214.fasta	122,519.10	100.00%	18	19	20	0.12%	22.80%	LQLILADLLR	99.70%	50	25	35.5	1,167.75
ELF3-HFC Col rep2	| Symbols: PHYE | phytochrome E | chr4:10042312-10045948 REVERSE LENGTH=1112	AT4G18130.1	TAIR10_20101214.fasta	122,519.10	100.00%	18	19	20	0.12%	22.80%	NIVNHAPFPNSWVGISISPGQELSR	99.70%	52	31.1	40.6	2,719.40
ELF3-HFC Col rep2	| Symbols: PHYE | phytochrome E | chr4:10042312-10045948 REVERSE LENGTH=1112	AT4G18130.1	TAIR10_20101214.fasta	122,519.10	100.00%	18	19	20	0.12%	22.80%	QFLETSDACEK	99.70%	61.8	25.7	29.8	1,327.58
ELF3-HFC Col rep2	| Symbols: PHYE | phytochrome E | chr4:10042312-10045948 REVERSE LENGTH=1112	AT4G18130.1	TAIR10_20101214.fasta	122,519.10	100.00%	18	19	20	0.12%	22.80%	RPLCLVNSTLR	99.40%	30	28.5	19.6	1,328.75
ELF3-HFC Col rep2	| Symbols: PHYE | phytochrome E | chr4:10042312-10045948 REVERSE LENGTH=1112	AT4G18130.1	TAIR10_20101214.fasta	122,519.10	100.00%	18	19	20	0.12%	22.80%	SDLEPYLGLHYPATDIPQAAR	99.70%	47.2	31.4	43.7	2,327.17
ELF3-HFC Col rep2	| Symbols: PHYE | phytochrome E | chr4:10042312-10045948 REVERSE LENGTH=1112	AT4G18130.1	TAIR10_20101214.fasta	122,519.10	100.00%	18	19	20	0.12%	22.80%	SLADEIVQEESR	99.70%	82.7	29.3	73.9	1,375.67
ELF3-HFC Col rep2	| Symbols: PHYE | phytochrome E | chr4:10042312-10045948 REVERSE LENGTH=1112	AT4G18130.1	TAIR10_20101214.fasta	122,519.10	100.00%	18	19	20	0.12%	22.80%	SSFTAFLEVAK	99.70%	87	27.3	79.5	1,199.63
ELF3-HFC Col rep2	| Symbols: PHYE | phytochrome E | chr4:10042312-10045948 REVERSE LENGTH=1112	AT4G18130.1	TAIR10_20101214.fasta	122,519.10	100.00%	18	19	20	0.12%	22.80%	TLFTPSSGASLSK	99.70%	39.5	29.1	21.9	1,295.68
ELF3-HFC Col rep2	| Symbols: PHYE | phytochrome E | chr4:10042312-10045948 REVERSE LENGTH=1112	AT4G18130.1	TAIR10_20101214.fasta	122,519.10	100.00%	18	19	20	0.12%	22.80%	TTQKPFYAILHR	99.70%	37.3	29.4	29	1,474.82
ELF3-HFC Col rep2	| Symbols: PHYE | phytochrome E | chr4:10042312-10045948 REVERSE LENGTH=1112	AT4G18130.1	TAIR10_20101214.fasta	122,519.10	100.00%	18	19	20	0.12%	22.80%	VVQSEELK	95.60%	28.2	31.2	16.5	931.5095
ELF3-HFC Col rep2	| Symbols: PHYE | phytochrome E | chr4:10042312-10045948 REVERSE LENGTH=1112	AT4G18130.1	TAIR10_20101214.fasta	122,519.10	100.00%	18	19	20	0.12%	22.80%	YIEASLTANK	99.70%	57.6	28.8	40.9	1,109.58
ELF3-HFC Col rep2	| Symbols: SPA1 | SPA (suppressor of phyA-105) protein family | chr2:19022572-19026821 REVERSE LENGTH=1029	AT2G46340.1	TAIR10_20101214.fasta	114,689.10	100.00%	7	7	7	0.04%	11.10%	LLQDIQTLEDDIKEAER	97.20%	30.9	30.1	19.4	2,029.05
ELF3-HFC Col rep2	| Symbols: SPA1 | SPA (suppressor of phyA-105) protein family | chr2:19022572-19026821 REVERSE LENGTH=1029	AT2G46340.1	TAIR10_20101214.fasta	114,689.10	100.00%	7	7	7	0.04%	11.10%	NDLESDVDEDLNR	99.70%	77.2	25.5	65.1	1,533.67
ELF3-HFC Col rep2	| Symbols: SPA1 | SPA (suppressor of phyA-105) protein family | chr2:19022572-19026821 REVERSE LENGTH=1029	AT2G46340.1	TAIR10_20101214.fasta	114,689.10	100.00%	7	7	7	0.04%	11.10%	NQETPPEFVSDQDLGSK	99.70%	65.4	29.1	58.7	1,890.87
ELF3-HFC Col rep2	| Symbols: SPA1 | SPA (suppressor of phyA-105) protein family | chr2:19022572-19026821 REVERSE LENGTH=1029	AT2G46340.1	TAIR10_20101214.fasta	114,689.10	100.00%	7	7	7	0.04%	11.10%	SGDLLNSASVVCSLSFDPDEEHIAAAGISK	99.70%	82.4	31.4	73	3,089.47
ELF3-HFC Col rep2	| Symbols: SPA1 | SPA (suppressor of phyA-105) protein family | chr2:19022572-19026821 REVERSE LENGTH=1029	AT2G46340.1	TAIR10_20101214.fasta	114,689.10	100.00%	7	7	7	0.04%	11.10%	TPVSSSNFSQLLLK	99.70%	95.2	28.6	85.3	1,520.83
ELF3-HFC Col rep2	| Symbols: SPA1 | SPA (suppressor of phyA-105) protein family | chr2:19022572-19026821 REVERSE LENGTH=1029	AT2G46340.1	TAIR10_20101214.fasta	114,689.10	100.00%	7	7	7	0.04%	11.10%	VAEETVATNNIQLK	99.70%	78.1	29.9	69.9	1,529.82
ELF3-HFC Col rep2	| Symbols: SPA1 | SPA (suppressor of phyA-105) protein family | chr2:19022572-19026821 REVERSE LENGTH=1029	AT2G46340.1	TAIR10_20101214.fasta	114,689.10	100.00%	7	7	7	0.04%	11.10%	YSSNVSLVR	99.70%	64.7	27.9	51.1	1,024.54
ELF3-HFC Col rep2	| Symbols: TIC | time for coffee | chr3:7913181-7918879 FORWARD LENGTH=1550	AT3G22380.1,AT3G22380.2	TAIR10_20101214.fasta	164,452.80	100.00%	4	4	4	0.02%	4.19%	NIQSHQQFTK	99.20%	29.1	28.8	19.7	1,230.62
ELF3-HFC Col rep2	| Symbols: TIC | time for coffee | chr3:7913181-7918879 FORWARD LENGTH=1550	AT3G22380.1,AT3G22380.2	TAIR10_20101214.fasta	164,452.80	100.00%	4	4	4	0.02%	4.19%	QDLADASVSAVTSGSIVDSSSR	99.70%	108	31.2	105	2,152.04
ELF3-HFC Col rep2	| Symbols: TIC | time for coffee | chr3:7913181-7918879 FORWARD LENGTH=1550	AT3G22380.1,AT3G22380.2	TAIR10_20101214.fasta	164,452.80	100.00%	4	4	4	0.02%	4.19%	QEAAGNDLTEAAK	99.70%	60.2	28.1	51.1	1,317.63
ELF3-HFC Col rep2	| Symbols: TIC | time for coffee | chr3:7913181-7918879 FORWARD LENGTH=1550	AT3G22380.1,AT3G22380.2	TAIR10_20101214.fasta	164,452.80	100.00%	4	4	4	0.02%	4.19%	SSGSGEGNSSVLDSIIPLTR	99.70%	98.6	31.3	91	1,975.99
ELF3-HFC Col rep2	| Symbols: TOC1, APRR1, PRR1, AtTOC1 | CCT motif -containing response regulator protein | chr5:24675540-24678176 FORWARD LENGTH=618	AT5G61380.1	TAIR10_20101214.fasta	69,195.40	100.00%	2	2	2	0.01%	5.34%	FQVVASEGINNTK	99.70%	82.8	30.2	64.8	1,406.73
ELF3-HFC Col rep2	| Symbols: TOC1, APRR1, PRR1, AtTOC1 | CCT motif -containing response regulator protein | chr5:24675540-24678176 FORWARD LENGTH=618	AT5G61380.1	TAIR10_20101214.fasta	69,195.40	100.00%	2	2	2	0.01%	5.34%	GNLSHQENEWSVATAPVHAR	99.70%	38.3	31	33.6	2,203.06
ELF3-HFC Col rep2	| Symbols: TZP | zinc knuckle (CCHC-type) family protein | chr5:17527261-17530207 FORWARD LENGTH=831	AT5G43630.1	TAIR10_20101214.fasta	90,615.60	100.00%	24	30	34	0.20%	37.80%	AVFDAVQVLR	99.70%	76.8	28.4	52.1	1,117.64
ELF3-HFC Col rep2	| Symbols: TZP | zinc knuckle (CCHC-type) family protein | chr5:17527261-17530207 FORWARD LENGTH=831	AT5G43630.1	TAIR10_20101214.fasta	90,615.60	100.00%	24	30	34	0.20%	37.80%	AWWQSAGK	99.00%	40	28.8	18.8	933.4577
ELF3-HFC Col rep2	| Symbols: TZP | zinc knuckle (CCHC-type) family protein | chr5:17527261-17530207 FORWARD LENGTH=831	AT5G43630.1	TAIR10_20101214.fasta	90,615.60	100.00%	24	30	34	0.20%	37.80%	DCLEVTDTELR	99.70%	74.4	28.2	43.3	1,350.62
ELF3-HFC Col rep2	| Symbols: TZP | zinc knuckle (CCHC-type) family protein | chr5:17527261-17530207 FORWARD LENGTH=831	AT5G43630.1	TAIR10_20101214.fasta	90,615.60	100.00%	24	30	34	0.20%	37.80%	DLLVNEQLR	99.70%	37.4	26.1	14.2	1,099.61
ELF3-HFC Col rep2	| Symbols: TZP | zinc knuckle (CCHC-type) family protein | chr5:17527261-17530207 FORWARD LENGTH=831	AT5G43630.1	TAIR10_20101214.fasta	90,615.60	100.00%	24	30	34	0.20%	37.80%	DLVQNISVR	99.70%	53.7	28.8	36.5	1,043.58
ELF3-HFC Col rep2	| Symbols: TZP | zinc knuckle (CCHC-type) family protein | chr5:17527261-17530207 FORWARD LENGTH=831	AT5G43630.1	TAIR10_20101214.fasta	90,615.60	100.00%	24	30	34	0.20%	37.80%	EANASASDAAK	97.60%	28.6	26.5	23.4	1,034.47
ELF3-HFC Col rep2	| Symbols: TZP | zinc knuckle (CCHC-type) family protein | chr5:17527261-17530207 FORWARD LENGTH=831	AT5G43630.1	TAIR10_20101214.fasta	90,615.60	100.00%	24	30	34	0.20%	37.80%	FAAVDAITELVWSPSNGLSLR	99.70%	35.9	30.2	30.2	2,246.18
ELF3-HFC Col rep2	| Symbols: TZP | zinc knuckle (CCHC-type) family protein | chr5:17527261-17530207 FORWARD LENGTH=831	AT5G43630.1	TAIR10_20101214.fasta	90,615.60	100.00%	24	30	34	0.20%	37.80%	GDDLSSSGNEIGPVAEPNISSGK	99.70%	95.8	31.2	83.6	2,230.05
ELF3-HFC Col rep2	| Symbols: TZP | zinc knuckle (CCHC-type) family protein | chr5:17527261-17530207 FORWARD LENGTH=831	AT5G43630.1	TAIR10_20101214.fasta	90,615.60	100.00%	24	30	34	0.20%	37.80%	GDYLPEGEAGPSGSYR	99.70%	57.8	27.1	49.1	1,654.73
ELF3-HFC Col rep2	| Symbols: TZP | zinc knuckle (CCHC-type) family protein | chr5:17527261-17530207 FORWARD LENGTH=831	AT5G43630.1	TAIR10_20101214.fasta	90,615.60	100.00%	24	30	34	0.20%	37.80%	GNEEDNSPFVALTTTSNANGHGQVNAIVDQQQLSPCCVK	99.70%	95.2	30.8	2.01	4,201.90
ELF3-HFC Col rep2	| Symbols: TZP | zinc knuckle (CCHC-type) family protein | chr5:17527261-17530207 FORWARD LENGTH=831	AT5G43630.1	TAIR10_20101214.fasta	90,615.60	100.00%	24	30	34	0.20%	37.80%	GVTCLVESQFISNQDFLEEELK	99.70%	104	31.5	95.3	2,585.24
ELF3-HFC Col rep2	| Symbols: TZP | zinc knuckle (CCHC-type) family protein | chr5:17527261-17530207 FORWARD LENGTH=831	AT5G43630.1	TAIR10_20101214.fasta	90,615.60	100.00%	24	30	34	0.20%	37.80%	IDGDTEGQSSR	99.70%	76.9	25.7	63.1	1,164.51
ELF3-HFC Col rep2	| Symbols: TZP | zinc knuckle (CCHC-type) family protein | chr5:17527261-17530207 FORWARD LENGTH=831	AT5G43630.1	TAIR10_20101214.fasta	90,615.60	100.00%	24	30	34	0.20%	37.80%	IESSEAMASLFAR	99.70%	77.8	29.3	65.3	1,411.69
ELF3-HFC Col rep2	| Symbols: TZP | zinc knuckle (CCHC-type) family protein | chr5:17527261-17530207 FORWARD LENGTH=831	AT5G43630.1	TAIR10_20101214.fasta	90,615.60	100.00%	24	30	34	0.20%	37.80%	KRPGFEEQLIFGSK	99.70%	62.4	28.5	56.2	1,635.89
ELF3-HFC Col rep2	| Symbols: TZP | zinc knuckle (CCHC-type) family protein | chr5:17527261-17530207 FORWARD LENGTH=831	AT5G43630.1	TAIR10_20101214.fasta	90,615.60	100.00%	24	30	34	0.20%	37.80%	KSVSGLEGFFLR	99.70%	33.1	26.9	13.6	1,339.74
ELF3-HFC Col rep2	| Symbols: TZP | zinc knuckle (CCHC-type) family protein | chr5:17527261-17530207 FORWARD LENGTH=831	AT5G43630.1	TAIR10_20101214.fasta	90,615.60	100.00%	24	30	34	0.20%	37.80%	LPISGFTDVPR	99.70%	67.1	29.5	58.8	1,201.66
ELF3-HFC Col rep2	| Symbols: TZP | zinc knuckle (CCHC-type) family protein | chr5:17527261-17530207 FORWARD LENGTH=831	AT5G43630.1	TAIR10_20101214.fasta	90,615.60	100.00%	24	30	34	0.20%	37.80%	RPGFEEQLIFGSK	99.70%	51.9	29.9	33.1	1,507.79
ELF3-HFC Col rep2	| Symbols: TZP | zinc knuckle (CCHC-type) family protein | chr5:17527261-17530207 FORWARD LENGTH=831	AT5G43630.1	TAIR10_20101214.fasta	90,615.60	100.00%	24	30	34	0.20%	37.80%	SIMPSGSLAENAEEEQR	99.70%	45.3	27.7	45.3	1,863.84
ELF3-HFC Col rep2	| Symbols: TZP | zinc knuckle (CCHC-type) family protein | chr5:17527261-17530207 FORWARD LENGTH=831	AT5G43630.1	TAIR10_20101214.fasta	90,615.60	100.00%	24	30	34	0.20%	37.80%	SVSGLEGFFLR	99.70%	65.9	27.9	51.5	1,211.64
ELF3-HFC Col rep2	| Symbols: TZP | zinc knuckle (CCHC-type) family protein | chr5:17527261-17530207 FORWARD LENGTH=831	AT5G43630.1	TAIR10_20101214.fasta	90,615.60	100.00%	24	30	34	0.20%	37.80%	TLNQECLGSTSK	99.60%	52.1	29.3	42.8	1,337.64
ELF3-HFC Col rep2	| Symbols: TZP | zinc knuckle (CCHC-type) family protein | chr5:17527261-17530207 FORWARD LENGTH=831	AT5G43630.1	TAIR10_20101214.fasta	90,615.60	100.00%	24	30	34	0.20%	37.80%	TSGYDGIPSAEELSR	99.70%	59.2	29.8	27.3	1,581.74
ELF3-HFC Col rep2	| Symbols: TZP | zinc knuckle (CCHC-type) family protein | chr5:17527261-17530207 FORWARD LENGTH=831	AT5G43630.1	TAIR10_20101214.fasta	90,615.60	100.00%	24	30	34	0.20%	37.80%	VSPCLTNR	99.00%	35.9	28.9	15	946.4775
ELF3-HFC Col rep2	| Symbols: TZP | zinc knuckle (CCHC-type) family protein | chr5:17527261-17530207 FORWARD LENGTH=831	AT5G43630.1	TAIR10_20101214.fasta	90,615.60	100.00%	24	30	34	0.20%	37.80%	VVFNQTSK	99.00%	35.1	28.6	18.2	922.4992
ELF3-HFC Col rep2	| Symbols: TZP | zinc knuckle (CCHC-type) family protein | chr5:17527261-17530207 FORWARD LENGTH=831	AT5G43630.1	TAIR10_20101214.fasta	90,615.60	100.00%	24	30	34	0.20%	37.80%	WEEGLGGTGYYVAR	99.70%	95.7	29.1	87.6	1,557.73
ELF3-HFC elf4-2 rep 1	| Symbols: | BED zinc finger ;hAT family dimerisation domain | chr3:14321838-14323928 FORWARD LENGTH=696	AT3G42170.1	TAIR10_20101214.fasta	78,815.20	100.00%	6	6	6	0.04%	12.20%	AIAGEDPFVTGIAK	99.70%	68.3	30.2	55.4	1,388.74
ELF3-HFC elf4-2 rep 1	| Symbols: | BED zinc finger ;hAT family dimerisation domain | chr3:14321838-14323928 FORWARD LENGTH=696	AT3G42170.1	TAIR10_20101214.fasta	78,815.20	100.00%	6	6	6	0.04%	12.20%	EVFSCLDTADPDYK	99.70%	32.9	25.9	27.7	1,659.72
ELF3-HFC elf4-2 rep 1	| Symbols: | BED zinc finger ;hAT family dimerisation domain | chr3:14321838-14323928 FORWARD LENGTH=696	AT3G42170.1	TAIR10_20101214.fasta	78,815.20	100.00%	6	6	6	0.04%	12.20%	NPGILDGQLVIGNCVAR	99.70%	64.9	29.6	57.4	1,795.95
ELF3-HFC elf4-2 rep 1	| Symbols: | BED zinc finger ;hAT family dimerisation domain | chr3:14321838-14323928 FORWARD LENGTH=696	AT3G42170.1	TAIR10_20101214.fasta	78,815.20	100.00%	6	6	6	0.04%	12.20%	SELDQYLDETLLPR	99.70%	47.8	31	34.9	1,691.85
ELF3-HFC elf4-2 rep 1	| Symbols: | BED zinc finger ;hAT family dimerisation domain | chr3:14321838-14323928 FORWARD LENGTH=696	AT3G42170.1	TAIR10_20101214.fasta	78,815.20	100.00%	6	6	6	0.04%	12.20%	SQNNASPYVAFNQDK	99.70%	76.5	29	74.3	1,682.78
ELF3-HFC elf4-2 rep 1	| Symbols: | BED zinc finger ;hAT family dimerisation domain | chr3:14321838-14323928 FORWARD LENGTH=696	AT3G42170.1	TAIR10_20101214.fasta	78,815.20	100.00%	6	6	6	0.04%	12.20%	VQEFDVLDWWK	99.70%	54.2	30.5	33.4	1,464.72
ELF3-HFC elf4-2 rep 1	| Symbols: | C2 calcium/lipid-binding plant phosphoribosyltransferase family protein | chr5:2127200-2129584 REVERSE LENGTH=794	AT5G06850.1	TAIR10_20101214.fasta	91,004.50	100.00%	7	8	8	0.05%	11.10%	FGFGALEGDKR	99.70%	39.1	28.7	20.9	1,196.61
ELF3-HFC elf4-2 rep 1	| Symbols: | C2 calcium/lipid-binding plant phosphoribosyltransferase family protein | chr5:2127200-2129584 REVERSE LENGTH=794	AT5G06850.1	TAIR10_20101214.fasta	91,004.50	100.00%	7	8	8	0.05%	11.10%	LISPLSVFEK	99.70%	39.2	26.3	17.7	1,132.66
ELF3-HFC elf4-2 rep 1	| Symbols: | C2 calcium/lipid-binding plant phosphoribosyltransferase family protein | chr5:2127200-2129584 REVERSE LENGTH=794	AT5G06850.1	TAIR10_20101214.fasta	91,004.50	100.00%	7	8	8	0.05%	11.10%	LSTLEADR	95.40%	27.6	30.3	9.36	904.4735
ELF3-HFC elf4-2 rep 1	| Symbols: | C2 calcium/lipid-binding plant phosphoribosyltransferase family protein | chr5:2127200-2129584 REVERSE LENGTH=794	AT5G06850.1	TAIR10_20101214.fasta	91,004.50	100.00%	7	8	8	0.05%	11.10%	TNPEWNQVFAFSK	99.70%	44.7	29.9	41.9	1,567.75
ELF3-HFC elf4-2 rep 1	| Symbols: | C2 calcium/lipid-binding plant phosphoribosyltransferase family protein | chr5:2127200-2129584 REVERSE LENGTH=794	AT5G06850.1	TAIR10_20101214.fasta	91,004.50	100.00%	7	8	8	0.05%	11.10%	VNVIEAQDVEPSDR	99.70%	79.2	29.6	72.7	1,570.77
ELF3-HFC elf4-2 rep 1	| Symbols: | C2 calcium/lipid-binding plant phosphoribosyltransferase family protein | chr5:2127200-2129584 REVERSE LENGTH=794	AT5G06850.1	TAIR10_20101214.fasta	91,004.50	100.00%	7	8	8	0.05%	11.10%	VQVGNQILK	99.50%	30.2	25	17.3	998.5994
ELF3-HFC elf4-2 rep 1	| Symbols: | C2 calcium/lipid-binding plant phosphoribosyltransferase family protein | chr5:2127200-2129584 REVERSE LENGTH=794	AT5G06850.1	TAIR10_20101214.fasta	91,004.50	100.00%	7	8	8	0.05%	11.10%	VSWAEAASPDELDEEFDTFPTSK	99.70%	66.2	29	66.2	2,571.14
ELF3-HFC elf4-2 rep 1	| Symbols: | F-box family protein | chr2:7074940-7079181 FORWARD LENGTH=727	AT2G16365.4	TAIR10_20101214.fasta	82,716.20	100.00%	13	13	13	0.08%	24.60%	ALSGLNPYGLR	99.70%	60.9	30.1	42.9	1,160.64
ELF3-HFC elf4-2 rep 1	| Symbols: | F-box family protein | chr2:7074940-7079181 FORWARD LENGTH=727	AT2G16365.4	TAIR10_20101214.fasta	82,716.20	100.00%	13	13	13	0.08%	24.60%	ENNNLFLEILK	99.70%	72.5	29.7	52.7	1,346.73
ELF3-HFC elf4-2 rep 1	| Symbols: | F-box family protein | chr2:7074940-7079181 FORWARD LENGTH=727	AT2G16365.4	TAIR10_20101214.fasta	82,716.20	100.00%	13	13	13	0.08%	24.60%	IFGGYLDLFPNQDHSHNR	99.70%	47.2	30.6	35	2,130.02
ELF3-HFC elf4-2 rep 1	| Symbols: | F-box family protein | chr2:7074940-7079181 FORWARD LENGTH=727	AT2G16365.4	TAIR10_20101214.fasta	82,716.20	100.00%	13	13	13	0.08%	24.60%	IYATVDSVNLPK	99.70%	46.2	28.9	32	1,319.72
ELF3-HFC elf4-2 rep 1	| Symbols: | F-box family protein | chr2:7074940-7079181 FORWARD LENGTH=727	AT2G16365.4	TAIR10_20101214.fasta	82,716.20	100.00%	13	13	13	0.08%	24.60%	LDFPIQEK	99.00%	37.6	29.7	8.59	989.5304
ELF3-HFC elf4-2 rep 1	| Symbols: | F-box family protein | chr2:7074940-7079181 FORWARD LENGTH=727	AT2G16365.4	TAIR10_20101214.fasta	82,716.20	100.00%	13	13	13	0.08%	24.60%	LQSLESSKDTQEDGPR	96.80%	24.8	30	18	1,789.86
ELF3-HFC elf4-2 rep 1	| Symbols: | F-box family protein | chr2:7074940-7079181 FORWARD LENGTH=727	AT2G16365.4	TAIR10_20101214.fasta	82,716.20	100.00%	13	13	13	0.08%	24.60%	LQTIHLSGSISSSSTK	99.70%	40.8	30.4	36.2	1,645.88
ELF3-HFC elf4-2 rep 1	| Symbols: | F-box family protein | chr2:7074940-7079181 FORWARD LENGTH=727	AT2G16365.4	TAIR10_20101214.fasta	82,716.20	100.00%	13	13	13	0.08%	24.60%	NSSVNPYESAWLGR	99.70%	79.1	30	74.1	1,579.75
ELF3-HFC elf4-2 rep 1	| Symbols: | F-box family protein | chr2:7074940-7079181 FORWARD LENGTH=727	AT2G16365.4	TAIR10_20101214.fasta	82,716.20	100.00%	13	13	13	0.08%	24.60%	QGGDDITLLHPWIQR	99.40%	28.7	30.7	17.5	1,748.91
ELF3-HFC elf4-2 rep 1	| Symbols: | F-box family protein | chr2:7074940-7079181 FORWARD LENGTH=727	AT2G16365.4	TAIR10_20101214.fasta	82,716.20	100.00%	13	13	13	0.08%	24.60%	SGINNLQPR	99.50%	26.5	25.4	12.2	998.5378
ELF3-HFC elf4-2 rep 1	| Symbols: | F-box family protein | chr2:7074940-7079181 FORWARD LENGTH=727	AT2G16365.4	TAIR10_20101214.fasta	82,716.20	100.00%	13	13	13	0.08%	24.60%	TTQNLLEIRPVR	99.70%	50.3	25	39.9	1,552.92
ELF3-HFC elf4-2 rep 1	| Symbols: | F-box family protein | chr2:7074940-7079181 FORWARD LENGTH=727	AT2G16365.4	TAIR10_20101214.fasta	82,716.20	100.00%	13	13	13	0.08%	24.60%	TTSTDQPTGQEASFEPESHKEFEK	99.70%	35.6	29.1	30	2,710.21
ELF3-HFC elf4-2 rep 1	| Symbols: | F-box family protein | chr2:7074940-7079181 FORWARD LENGTH=727	AT2G16365.4	TAIR10_20101214.fasta	82,716.20	100.00%	13	13	13	0.08%	24.60%	VRLEPEVEASSR	99.70%	56.4	29.1	50	1,371.72
ELF3-HFC elf4-2 rep 1	| Symbols: ATAN11, LWD1 | Transducin/WD40 repeat-like superfamily protein | chr1:4395114-4396154 REVERSE LENGTH=346	AT1G12910.1	TAIR10_20101214.fasta	39,089.30	100.00%	2	2	2	0.01%	10.70%	DKEHSTIIYESSEPDTPLVR	99.70%	43.8	31.7	31.6	2,316.14
ELF3-HFC elf4-2 rep 1	| Symbols: ATAN11, LWD1 | Transducin/WD40 repeat-like superfamily protein | chr1:4395114-4396154 REVERSE LENGTH=346	AT1G12910.1	TAIR10_20101214.fasta	39,089.30	100.00%	2	2	2	0.01%	10.70%	IGTSSTDTTCTIWDIER	99.70%	45.6	29.7	43	1,955.90
ELF3-HFC elf4-2 rep 1	| Symbols: ATARCA, RACK1A_AT, RACK1A | Transducin/WD40 repeat-like superfamily protein | chr1:6222325-6223901 FORWARD LENGTH=327	AT1G18080.1	TAIR10_20101214.fasta	35,748.00	100.00%	2	2	3	0.02%	11.30%	FSPNTLQPTIVSASWDK	99.70%	55.4	31.2	42.3	1,890.96
ELF3-HFC elf4-2 rep 1	| Symbols: ATARCA, RACK1A_AT, RACK1A | Transducin/WD40 repeat-like superfamily protein | chr1:6222325-6223901 FORWARD LENGTH=327	AT1G18080.1	TAIR10_20101214.fasta	35,748.00	100.00%	2	2	3	0.02%	11.30%	LWDLAAGVSTR	99.70%	59	29.9	48.8	1,188.64
ELF3-HFC elf4-2 rep 1	| Symbols: ATARCA, RACK1A_AT, RACK1A | Transducin/WD40 repeat-like superfamily protein | chr1:6222325-6223901 FORWARD LENGTH=327	AT1G18080.1	TAIR10_20101214.fasta	35,748.00	100.00%	2	2	3	0.02%	11.30%	LWNTLGECK	99.70%	35.8	29.3	23.2	1,120.55
ELF3-HFC elf4-2 rep 1	| Symbols: COP1, ATCOP1, DET340, FUS1, EMB168 | Transducin/WD40 repeat-like superfamily protein | chr2:13978000-13983282 FORWARD LENGTH=675	AT2G32950.1	TAIR10_20101214.fasta	76,188.00	100.00%	5	5	5	0.03%	11.60%	FLSNNELASASTDSTLR	99.70%	114	30.5	101	1,825.89
ELF3-HFC elf4-2 rep 1	| Symbols: COP1, ATCOP1, DET340, FUS1, EMB168 | Transducin/WD40 repeat-like superfamily protein | chr2:13978000-13983282 FORWARD LENGTH=675	AT2G32950.1	TAIR10_20101214.fasta	76,188.00	100.00%	5	5	5	0.03%	11.60%	GGNFVGNYQNK	99.70%	34	28.8	19.6	1,197.56
ELF3-HFC elf4-2 rep 1	| Symbols: COP1, ATCOP1, DET340, FUS1, EMB168 | Transducin/WD40 repeat-like superfamily protein | chr2:13978000-13983282 FORWARD LENGTH=675	AT2G32950.1	TAIR10_20101214.fasta	76,188.00	100.00%	5	5	5	0.03%	11.60%	IHAQFNDLQECYLQK	99.70%	50	30.6	46.5	1,906.91
ELF3-HFC elf4-2 rep 1	| Symbols: COP1, ATCOP1, DET340, FUS1, EMB168 | Transducin/WD40 repeat-like superfamily protein | chr2:13978000-13983282 FORWARD LENGTH=675	AT2G32950.1	TAIR10_20101214.fasta	76,188.00	100.00%	5	5	5	0.03%	11.60%	NHIASSDYEGIVTVWDVTTR	99.70%	47.4	31.1	41.3	2,263.10
ELF3-HFC elf4-2 rep 1	| Symbols: COP1, ATCOP1, DET340, FUS1, EMB168 | Transducin/WD40 repeat-like superfamily protein | chr2:13978000-13983282 FORWARD LENGTH=675	AT2G32950.1	TAIR10_20101214.fasta	76,188.00	100.00%	5	5	5	0.03%	11.60%	VDELNEVQTDLQYIK	99.70%	57.3	31.2	51.3	1,806.91
ELF3-HFC elf4-2 rep 1	| Symbols: CRB, CSP41B, HIP1.3 | chloroplast RNA binding | chr1:3015473-3018035 FORWARD LENGTH=378	AT1G09340.1	TAIR10_20101214.fasta	42,621.40	100.00%	6	6	6	0.04%	25.70%	AGRPIPVPNSGIQISQLGHVK	99.70%	56.1	25	4.5	2,169.21
ELF3-HFC elf4-2 rep 1	| Symbols: CRB, CSP41B, HIP1.3 | chloroplast RNA binding | chr1:3015473-3018035 FORWARD LENGTH=378	AT1G09340.1	TAIR10_20101214.fasta	42,621.40	100.00%	6	6	6	0.04%	25.70%	DLATAFLNVLGNEK	99.70%	91.7	30.2	80.9	1,504.80
ELF3-HFC elf4-2 rep 1	| Symbols: CRB, CSP41B, HIP1.3 | chloroplast RNA binding | chr1:3015473-3018035 FORWARD LENGTH=378	AT1G09340.1	TAIR10_20101214.fasta	42,621.40	100.00%	6	6	6	0.04%	25.70%	EAEEVEPILEALPK	99.70%	39.4	29.6	34.5	1,566.83
ELF3-HFC elf4-2 rep 1	| Symbols: CRB, CSP41B, HIP1.3 | chloroplast RNA binding | chr1:3015473-3018035 FORWARD LENGTH=378	AT1G09340.1	TAIR10_20101214.fasta	42,621.40	100.00%	6	6	6	0.04%	25.70%	LEQYIYCSSAGVYLK	99.50%	36.3	31	32.8	1,793.88
ELF3-HFC elf4-2 rep 1	| Symbols: CRB, CSP41B, HIP1.3 | chloroplast RNA binding | chr1:3015473-3018035 FORWARD LENGTH=378	AT1G09340.1	TAIR10_20101214.fasta	42,621.40	100.00%	6	6	6	0.04%	25.70%	QLPGESDQDFADFSSK	99.70%	55.3	27.5	54.3	1,770.78
ELF3-HFC elf4-2 rep 1	| Symbols: CRB, CSP41B, HIP1.3 | chloroplast RNA binding | chr1:3015473-3018035 FORWARD LENGTH=378	AT1G09340.1	TAIR10_20101214.fasta	42,621.40	100.00%	6	6	6	0.04%	25.70%	SSLSAEGFDVVYDINGR	99.70%	47.2	30	38.8	1,828.87
ELF3-HFC elf4-2 rep 1	| Symbols: ELF3, PYK20 | hydroxyproline-rich glycoprotein family protein | chr2:11059459-11063178 FORWARD LENGTH=695	AT2G25930.1	TAIR10_20101214.fasta	77,204.80	100.00%	22	37	51	0.30%	41.70%	DTDNGAESHLATENHSQEGHGSPEDIDNDREYSK	99.70%	43.4	25.4	36.3	3,754.57
ELF3-HFC elf4-2 rep 1	| Symbols: ELF3, PYK20 | hydroxyproline-rich glycoprotein family protein | chr2:11059459-11063178 FORWARD LENGTH=695	AT2G25930.1	TAIR10_20101214.fasta	77,204.80	100.00%	22	37	51	0.30%	41.70%	EEDFAVPVYINSR	99.70%	86.8	30.2	69.4	1,667.79
ELF3-HFC elf4-2 rep 1	| Symbols: ELF3, PYK20 | hydroxyproline-rich glycoprotein family protein | chr2:11059459-11063178 FORWARD LENGTH=695	AT2G25930.1	TAIR10_20101214.fasta	77,204.80	100.00%	22	37	51	0.30%	41.70%	FQEVNQTGSK	99.70%	61.8	29.6	42.9	1,137.55
ELF3-HFC elf4-2 rep 1	| Symbols: ELF3, PYK20 | hydroxyproline-rich glycoprotein family protein | chr2:11059459-11063178 FORWARD LENGTH=695	AT2G25930.1	TAIR10_20101214.fasta	77,204.80	100.00%	22	37	51	0.30%	41.70%	IFQSIQEER	99.70%	63.6	30.2	30.8	1,149.59
ELF3-HFC elf4-2 rep 1	| Symbols: ELF3, PYK20 | hydroxyproline-rich glycoprotein family protein | chr2:11059459-11063178 FORWARD LENGTH=695	AT2G25930.1	TAIR10_20101214.fasta	77,204.80	100.00%	22	37	51	0.30%	41.70%	ILEPMFPR	99.00%	33.1	30.5	13.6	1,018.54
ELF3-HFC elf4-2 rep 1	| Symbols: ELF3, PYK20 | hydroxyproline-rich glycoprotein family protein | chr2:11059459-11063178 FORWARD LENGTH=695	AT2G25930.1	TAIR10_20101214.fasta	77,204.80	100.00%	22	37	51	0.30%	41.70%	LASENAAR	99.00%	61.7	29.1	41.8	831.4318
ELF3-HFC elf4-2 rep 1	| Symbols: ELF3, PYK20 | hydroxyproline-rich glycoprotein family protein | chr2:11059459-11063178 FORWARD LENGTH=695	AT2G25930.1	TAIR10_20101214.fasta	77,204.80	100.00%	22	37	51	0.30%	41.70%	LHVNDADK	99.00%	40.4	27.4	29.7	911.4582
ELF3-HFC elf4-2 rep 1	| Symbols: ELF3, PYK20 | hydroxyproline-rich glycoprotein family protein | chr2:11059459-11063178 FORWARD LENGTH=695	AT2G25930.1	TAIR10_20101214.fasta	77,204.80	100.00%	22	37	51	0.30%	41.70%	LHVNDADKGGPR	99.70%	56.8	30.3	51	1,278.66
ELF3-HFC elf4-2 rep 1	| Symbols: ELF3, PYK20 | hydroxyproline-rich glycoprotein family protein | chr2:11059459-11063178 FORWARD LENGTH=695	AT2G25930.1	TAIR10_20101214.fasta	77,204.80	100.00%	22	37	51	0.30%	41.70%	LIAASPDLLLDEISFLGK	99.70%	92.3	25.3	86	1,915.08
ELF3-HFC elf4-2 rep 1	| Symbols: ELF3, PYK20 | hydroxyproline-rich glycoprotein family protein | chr2:11059459-11063178 FORWARD LENGTH=695	AT2G25930.1	TAIR10_20101214.fasta	77,204.80	100.00%	22	37	51	0.30%	41.70%	LKDTDNGAESHLATENHSQEGHGSPEDIDNDR	99.70%	39.2	27.3	18.3	3,489.50
ELF3-HFC elf4-2 rep 1	| Symbols: ELF3, PYK20 | hydroxyproline-rich glycoprotein family protein | chr2:11059459-11063178 FORWARD LENGTH=695	AT2G25930.1	TAIR10_20101214.fasta	77,204.80	100.00%	22	37	51	0.30%	41.70%	LKDTDNGAESHLATENHSQEGHGSPEDIDNDREYSK	99.70%	67.8	28.3	60.3	3,995.75
ELF3-HFC elf4-2 rep 1	| Symbols: ELF3, PYK20 | hydroxyproline-rich glycoprotein family protein | chr2:11059459-11063178 FORWARD LENGTH=695	AT2G25930.1	TAIR10_20101214.fasta	77,204.80	100.00%	22	37	51	0.30%	41.70%	LLPSEFLVKPPLPHVVVK	99.40%	25.8	25	23	2,012.23
ELF3-HFC elf4-2 rep 1	| Symbols: ELF3, PYK20 | hydroxyproline-rich glycoprotein family protein | chr2:11059459-11063178 FORWARD LENGTH=695	AT2G25930.1	TAIR10_20101214.fasta	77,204.80	100.00%	22	37	51	0.30%	41.70%	MALYEQLSIPSQR	99.50%	34.6	30.7	22.5	1,551.78
ELF3-HFC elf4-2 rep 1	| Symbols: ELF3, PYK20 | hydroxyproline-rich glycoprotein family protein | chr2:11059459-11063178 FORWARD LENGTH=695	AT2G25930.1	TAIR10_20101214.fasta	77,204.80	100.00%	22	37	51	0.30%	41.70%	NLSVQHLDSSAANQATEK	99.70%	83.5	30.9	75.6	1,912.94
ELF3-HFC elf4-2 rep 1	| Symbols: ELF3, PYK20 | hydroxyproline-rich glycoprotein family protein | chr2:11059459-11063178 FORWARD LENGTH=695	AT2G25930.1	TAIR10_20101214.fasta	77,204.80	100.00%	22	37	51	0.30%	41.70%	QGSTGSSPSGPQGISGSK	99.70%	66.8	29	61.1	1,618.77
ELF3-HFC elf4-2 rep 1	| Symbols: ELF3, PYK20 | hydroxyproline-rich glycoprotein family protein | chr2:11059459-11063178 FORWARD LENGTH=695	AT2G25930.1	TAIR10_20101214.fasta	77,204.80	100.00%	22	37	51	0.30%	41.70%	QNVCLATCSKPEVR	99.70%	74.7	30.3	60.1	1,661.81
ELF3-HFC elf4-2 rep 1	| Symbols: ELF3, PYK20 | hydroxyproline-rich glycoprotein family protein | chr2:11059459-11063178 FORWARD LENGTH=695	AT2G25930.1	TAIR10_20101214.fasta	77,204.80	100.00%	22	37	51	0.30%	41.70%	SDNEPAPQQQQQPTK	99.70%	64.6	29.7	49.2	1,695.79
ELF3-HFC elf4-2 rep 1	| Symbols: ELF3, PYK20 | hydroxyproline-rich glycoprotein family protein | chr2:11059459-11063178 FORWARD LENGTH=695	AT2G25930.1	TAIR10_20101214.fasta	77,204.80	100.00%	22	37	51	0.30%	41.70%	SFRPFAAVDEDSNINNAPEQTMTTTTTTTR	99.70%	79.4	31	72.1	3,332.53
ELF3-HFC elf4-2 rep 1	| Symbols: ELF3, PYK20 | hydroxyproline-rich glycoprotein family protein | chr2:11059459-11063178 FORWARD LENGTH=695	AT2G25930.1	TAIR10_20101214.fasta	77,204.80	100.00%	22	37	51	0.30%	41.70%	SNNTSTLVHPGPSSQPCGVER	99.70%	41.1	30.5	37.1	2,224.04
ELF3-HFC elf4-2 rep 1	| Symbols: ELF3, PYK20 | hydroxyproline-rich glycoprotein family protein | chr2:11059459-11063178 FORWARD LENGTH=695	AT2G25930.1	TAIR10_20101214.fasta	77,204.80	100.00%	22	37	51	0.30%	41.70%	TTVTQTTR	99.00%	48.2	28.4	37	907.4844
ELF3-HFC elf4-2 rep 1	| Symbols: ELF3, PYK20 | hydroxyproline-rich glycoprotein family protein | chr2:11059459-11063178 FORWARD LENGTH=695	AT2G25930.1	TAIR10_20101214.fasta	77,204.80	100.00%	22	37	51	0.30%	41.70%	VFAVQLFELHR	99.70%	41.2	28.5	30.4	1,358.76
ELF3-HFC elf4-2 rep 1	| Symbols: ELF3, PYK20 | hydroxyproline-rich glycoprotein family protein | chr2:11059459-11063178 FORWARD LENGTH=695	AT2G25930.1	TAIR10_20101214.fasta	77,204.80	100.00%	22	37	51	0.30%	41.70%	VNDYNASLR	99.70%	72.5	29.1	56.5	1,051.52
ELF3-HFC elf4-2 rep 1	| Symbols: ELF4-L2 | ELF4-like 2 | chr1:27344988-27345347 FORWARD LENGTH=119	AT1G72630.1	TAIR10_20101214.fasta	13,409.00	100.00%	3	4	5	0.03%	47.10%	LLINEINQNHESK	99.70%	58.2	30.4	49	1,551.81
ELF3-HFC elf4-2 rep 1	| Symbols: ELF4-L2 | ELF4-like 2 | chr1:27344988-27345347 FORWARD LENGTH=119	AT1G72630.1	TAIR10_20101214.fasta	13,409.00	100.00%	3	4	5	0.03%	47.10%	SFVQVQDILDQNR	99.70%	84.7	30.7	64.1	1,561.80
ELF3-HFC elf4-2 rep 1	| Symbols: ELF4-L2 | ELF4-like 2 | chr1:27344988-27345347 FORWARD LENGTH=119	AT1G72630.1	TAIR10_20101214.fasta	13,409.00	100.00%	3	4	5	0.03%	47.10%	SVDASSEGESTGTLK	99.70%	107	29.1	97.7	1,467.68
ELF3-HFC elf4-2 rep 1	| Symbols: ELF4-L2 | ELF4-like 2 | chr1:27344988-27345347 FORWARD LENGTH=119	AT1G72630.1	TAIR10_20101214.fasta	13,409.00	100.00%	3	4	5	0.03%	47.10%	TVASLYGDLSHSFAR	99.70%	49.6	30.4	42.9	1,623.81
ELF3-HFC elf4-2 rep 1	| Symbols: ELF4-L3 | ELF4-like 3 | chr2:2459336-2459665 FORWARD LENGTH=109	AT2G06255.1	TAIR10_20101214.fasta	12,311.00	100.00%	2	3	4	0.02%	36.70%	LLINEINQNHESK	99.70%	58.2	30.4	49	1,551.81
ELF3-HFC elf4-2 rep 1	| Symbols: ELF4-L3 | ELF4-like 3 | chr2:2459336-2459665 FORWARD LENGTH=109	AT2G06255.1	TAIR10_20101214.fasta	12,311.00	100.00%	2	3	4	0.02%	36.70%	SFVQVQNILDHNR	99.70%	85	29.4	70.6	1,569.81
ELF3-HFC elf4-2 rep 1	| Symbols: ELF4-L3 | ELF4-like 3 | chr2:2459336-2459665 FORWARD LENGTH=109	AT2G06255.1	TAIR10_20101214.fasta	12,311.00	100.00%	2	3	4	0.02%	36.70%	VAHLYVDLSNNFSK	98.40%	28.9	31	10.8	1,606.82
ELF3-HFC elf4-2 rep 1	| Symbols: ELF4-L4 | ELF4-like 4 | chr1:5997932-5998276 FORWARD LENGTH=114	AT1G17455.1,AT1G17455.2	TAIR10_20101214.fasta	12,663.30	99.80%	1	1	2	0.01%	24.60%	LLINEINQNHESK	99.70%	58.2	30.4	49	1,551.81
ELF3-HFC elf4-2 rep 1	| Symbols: ELF4-L4 | ELF4-like 4 | chr1:5997932-5998276 FORWARD LENGTH=114	AT1G17455.1,AT1G17455.2	TAIR10_20101214.fasta	12,663.30	99.80%	1	1	2	0.01%	24.60%	SVDASSEGESSGTLK	99.70%	89.4	28.2	85.4	1,453.67
ELF3-HFC elf4-2 rep 1	| Symbols: GLP3, GLP3A, GLP3B, ATGER3, GER3 | germin 3 | chr5:6975315-6975950 REVERSE LENGTH=211	AT5G20630.1	TAIR10_20101214.fasta	21,836.90	100.00%	2	3	3	0.02%	16.10%	AAVTPAFAPAYAGINGLGVSLAR	99.70%	75.5	28	72.6	2,187.19
ELF3-HFC elf4-2 rep 1	| Symbols: GLP3, GLP3A, GLP3B, ATGER3, GER3 | germin 3 | chr5:6975315-6975950 REVERSE LENGTH=211	AT5G20630.1	TAIR10_20101214.fasta	21,836.90	100.00%	2	3	3	0.02%	16.10%	GPQSPSGYSCK	99.70%	39.2	25.9	38.9	1,167.51
ELF3-HFC elf4-2 rep 1	| Symbols: PCL1, LUX | Homeodomain-like superfamily protein | chr3:17183248-17184219 FORWARD LENGTH=323	AT3G46640.1,AT3G46640.2	TAIR10_20101214.fasta	34,648.50	100.00%	1	1	5	0.03%	18.60%	ENVASHLQK	99.40%	27.9	27.7	15.7	1,025.54
ELF3-HFC elf4-2 rep 1	| Symbols: PCL1, LUX | Homeodomain-like superfamily protein | chr3:17183248-17184219 FORWARD LENGTH=323	AT3G46640.1,AT3G46640.2	TAIR10_20101214.fasta	34,648.50	100.00%	1	1	5	0.03%	18.60%	FVDVVAHLGIK	99.10%	30.2	25	17.3	1,197.70
ELF3-HFC elf4-2 rep 1	| Symbols: PCL1, LUX | Homeodomain-like superfamily protein | chr3:17183248-17184219 FORWARD LENGTH=323	AT3G46640.1,AT3G46640.2	TAIR10_20101214.fasta	34,648.50	100.00%	1	1	5	0.03%	18.60%	GGSSGPNTSSSNNNVEEEDRVGSSSPGSDSK	99.70%	68.6	26	66	3,025.28
ELF3-HFC elf4-2 rep 1	| Symbols: PCL1, LUX | Homeodomain-like superfamily protein | chr3:17183248-17184219 FORWARD LENGTH=323	AT3G46640.1,AT3G46640.2	TAIR10_20101214.fasta	34,648.50	100.00%	1	1	5	0.03%	18.60%	LVWTPQLHK	97.00%	20.5	25.4	13.2	1,121.65
ELF3-HFC elf4-2 rep 1	| Symbols: PHYB, HY3, OOP1 | phytochrome B | chr2:8140079-8144151 FORWARD LENGTH=1172	AT2G18790.1	TAIR10_20101214.fasta	129,333.90	100.00%	24	33	50	0.30%	36.60%	AIQQYTVDAR	99.70%	74.8	29.9	65	1,164.60

ELF3-HFC elf4-2 rep 1	| Symbols: PHYB, HY3, OOP1 | phytochrome B | chr2:8140079-8144151 FORWARD LENGTH=1172	AT2G18790.1	TAIR10_20101214.fasta	129,333.90	100.00%	24	33	50	0.30%	36.60%	AISQLQALPGGDIK	99.70%	116	26.1	106	1,410.80
ELF3-HFC elf4-2 rep 1	| Symbols: PHYB, HY3, OOP1 | phytochrome B | chr2:8140079-8144151 FORWARD LENGTH=1172	AT2G18790.1	TAIR10_20101214.fasta	129,333.90	100.00%	24	33	50	0.30%	36.60%	AVFVVVNACSSK	99.70%	57.2	29.6	45.5	1,280.67
ELF3-HFC elf4-2 rep 1	| Symbols: PHYB, HY3, OOP1 | phytochrome B | chr2:8140079-8144151 FORWARD LENGTH=1172	AT2G18790.1	TAIR10_20101214.fasta	129,333.90	100.00%	24	33	50	0.30%	36.60%	DMAGEQGIDELGAVAR	99.70%	94.9	28.8	84	1,647.76
ELF3-HFC elf4-2 rep 1	| Symbols: PHYB, HY3, OOP1 | phytochrome B | chr2:8140079-8144151 FORWARD LENGTH=1172	AT2G18790.1	TAIR10_20101214.fasta	129,333.90	100.00%	24	33	50	0.30%	36.60%	DYLNNIVGVCFVGQDVTSQK	99.70%	85.8	31.5	78.1	2,256.10
ELF3-HFC elf4-2 rep 1	| Symbols: PHYB, HY3, OOP1 | phytochrome B | chr2:8140079-8144151 FORWARD LENGTH=1172	AT2G18790.1	TAIR10_20101214.fasta	129,333.90	100.00%	24	33	50	0.30%	36.60%	EITLLNPVWIHSK	99.70%	67.3	26.7	54.1	1,549.87
ELF3-HFC elf4-2 rep 1	| Symbols: PHYB, HY3, OOP1 | phytochrome B | chr2:8140079-8144151 FORWARD LENGTH=1172	AT2G18790.1	TAIR10_20101214.fasta	129,333.90	100.00%	24	33	50	0.30%	36.60%	ELAYICQVIK	99.70%	45.8	28.6	29.8	1,236.67
ELF3-HFC elf4-2 rep 1	| Symbols: PHYB, HY3, OOP1 | phytochrome B | chr2:8140079-8144151 FORWARD LENGTH=1172	AT2G18790.1	TAIR10_20101214.fasta	129,333.90	100.00%	24	33	50	0.30%	36.60%	FANSLLEATDLNEDQK	99.70%	100	30.6	33.7	1,808.86
ELF3-HFC elf4-2 rep 1	| Symbols: PHYB, HY3, OOP1 | phytochrome B | chr2:8140079-8144151 FORWARD LENGTH=1172	AT2G18790.1	TAIR10_20101214.fasta	129,333.90	100.00%	24	33	50	0.30%	36.60%	FHEDEHGEVVAESK	99.70%	103	27.5	94.5	1,612.72
ELF3-HFC elf4-2 rep 1	| Symbols: PHYB, HY3, OOP1 | phytochrome B | chr2:8140079-8144151 FORWARD LENGTH=1172	AT2G18790.1	TAIR10_20101214.fasta	129,333.90	100.00%	24	33	50	0.30%	36.60%	FINIQGDYK	99.70%	50.8	28.1	32.3	1,097.56
ELF3-HFC elf4-2 rep 1	| Symbols: PHYB, HY3, OOP1 | phytochrome B | chr2:8140079-8144151 FORWARD LENGTH=1172	AT2G18790.1	TAIR10_20101214.fasta	129,333.90	100.00%	24	33	50	0.30%	36.60%	FVQALLTANK	99.70%	73.2	26.1	49.2	1,104.64
ELF3-HFC elf4-2 rep 1	| Symbols: PHYB, HY3, OOP1 | phytochrome B | chr2:8140079-8144151 FORWARD LENGTH=1172	AT2G18790.1	TAIR10_20101214.fasta	129,333.90	100.00%	24	33	50	0.30%	36.60%	FVQALLTANKR	99.70%	53.8	25	27.3	1,260.74
ELF3-HFC elf4-2 rep 1	| Symbols: PHYB, HY3, OOP1 | phytochrome B | chr2:8140079-8144151 FORWARD LENGTH=1172	AT2G18790.1	TAIR10_20101214.fasta	129,333.90	100.00%	24	33	50	0.30%	36.60%	IQQLLAEFLLSIIR	99.70%	108	25	108	1,657.00
ELF3-HFC elf4-2 rep 1	| Symbols: PHYB, HY3, OOP1 | phytochrome B | chr2:8140079-8144151 FORWARD LENGTH=1172	AT2G18790.1	TAIR10_20101214.fasta	129,333.90	100.00%	24	33	50	0.30%	36.60%	LHAVFEQSGESGK	99.70%	36.6	29.4	35.4	1,388.68
ELF3-HFC elf4-2 rep 1	| Symbols: PHYB, HY3, OOP1 | phytochrome B | chr2:8140079-8144151 FORWARD LENGTH=1172	AT2G18790.1	TAIR10_20101214.fasta	129,333.90	100.00%	24	33	50	0.30%	36.60%	LIETATVPIFAVDAGGCINGWNAK	99.40%	43.8	31	42.2	2,517.28
ELF3-HFC elf4-2 rep 1	| Symbols: PHYB, HY3, OOP1 | phytochrome B | chr2:8140079-8144151 FORWARD LENGTH=1172	AT2G18790.1	TAIR10_20101214.fasta	129,333.90	100.00%	24	33	50	0.30%	36.60%	LLCDTVVESVR	99.70%	69.5	30.6	49.5	1,290.67
ELF3-HFC elf4-2 rep 1	| Symbols: PHYB, HY3, OOP1 | phytochrome B | chr2:8140079-8144151 FORWARD LENGTH=1172	AT2G18790.1	TAIR10_20101214.fasta	129,333.90	100.00%	24	33	50	0.30%	36.60%	NTGKPFYAILHR	99.70%	44.1	29.3	31.9	1,416.77
ELF3-HFC elf4-2 rep 1	| Symbols: PHYB, HY3, OOP1 | phytochrome B | chr2:8140079-8144151 FORWARD LENGTH=1172	AT2G18790.1	TAIR10_20101214.fasta	129,333.90	100.00%	24	33	50	0.30%	36.60%	QLLETSVSCEK	99.70%	63.2	30.2	45.8	1,293.64
ELF3-HFC elf4-2 rep 1	| Symbols: PHYB, HY3, OOP1 | phytochrome B | chr2:8140079-8144151 FORWARD LENGTH=1172	AT2G18790.1	TAIR10_20101214.fasta	129,333.90	100.00%	24	33	50	0.30%	36.60%	RDDLEPYIGLHYPATDIPQASR	99.70%	47.5	31.3	0	2,527.26
ELF3-HFC elf4-2 rep 1	| Symbols: PHYB, HY3, OOP1 | phytochrome B | chr2:8140079-8144151 FORWARD LENGTH=1172	AT2G18790.1	TAIR10_20101214.fasta	129,333.90	100.00%	24	33	50	0.30%	36.60%	RQDTECFTK	99.70%	29.6	26.1	5.01	1,184.54
ELF3-HFC elf4-2 rep 1	| Symbols: PHYB, HY3, OOP1 | phytochrome B | chr2:8140079-8144151 FORWARD LENGTH=1172	AT2G18790.1	TAIR10_20101214.fasta	129,333.90	100.00%	24	33	50	0.30%	36.60%	SFDYSQSLK	99.70%	44.1	26.9	33	1,074.51
ELF3-HFC elf4-2 rep 1	| Symbols: PHYB, HY3, OOP1 | phytochrome B | chr2:8140079-8144151 FORWARD LENGTH=1172	AT2G18790.1	TAIR10_20101214.fasta	129,333.90	100.00%	24	33	50	0.30%	36.60%	SIEVFGDQIR	99.70%	52.4	30.5	33.3	1,163.61
ELF3-HFC elf4-2 rep 1	| Symbols: PHYB, HY3, OOP1 | phytochrome B | chr2:8140079-8144151 FORWARD LENGTH=1172	AT2G18790.1	TAIR10_20101214.fasta	129,333.90	100.00%	24	33	50	0.30%	36.60%	SLFTSSSSILLER	99.70%	73.6	28.2	67	1,439.77
ELF3-HFC elf4-2 rep 1	| Symbols: PHYB, HY3, OOP1 | phytochrome B | chr2:8140079-8144151 FORWARD LENGTH=1172	AT2G18790.1	TAIR10_20101214.fasta	129,333.90	100.00%	24	33	50	0.30%	36.60%	SLVSDLIYK	99.70%	59.6	26.1	46.1	1,037.59
ELF3-HFC elf4-2 rep 1	| Symbols: PHYB, HY3, OOP1 | phytochrome B | chr2:8140079-8144151 FORWARD LENGTH=1172	AT2G18790.1	TAIR10_20101214.fasta	129,333.90	100.00%	24	33	50	0.30%	36.60%	SLVSDLIYKENEATVNK	99.70%	52.6	30	38.9	1,923.01
ELF3-HFC elf4-2 rep 1	| Symbols: PHYB, HY3, OOP1 | phytochrome B | chr2:8140079-8144151 FORWARD LENGTH=1172	AT2G18790.1	TAIR10_20101214.fasta	129,333.90	100.00%	24	33	50	0.30%	36.60%	SSFQAFLEVVK	99.70%	71.6	26.8	56.2	1,254.67
ELF3-HFC elf4-2 rep 1	| Symbols: PHYB, HY3, OOP1 | phytochrome B | chr2:8140079-8144151 FORWARD LENGTH=1172	AT2G18790.1	TAIR10_20101214.fasta	129,333.90	100.00%	24	33	50	0.30%	36.60%	TEDPALSIAGAVQSQK	99.70%	99.5	29.4	87.1	1,614.83
ELF3-HFC elf4-2 rep 1	| Symbols: PHYB, HY3, OOP1 | phytochrome B | chr2:8140079-8144151 FORWARD LENGTH=1172	AT2G18790.1	TAIR10_20101214.fasta	129,333.90	100.00%	24	33	50	0.30%	36.60%	TFSPELQGK	99.70%	43.2	30	38.2	1,006.52
ELF3-HFC elf4-2 rep 1	| Symbols: PHYB, HY3, OOP1 | phytochrome B | chr2:8140079-8144151 FORWARD LENGTH=1172	AT2G18790.1	TAIR10_20101214.fasta	129,333.90	100.00%	24	33	50	0.30%	36.60%	TTTYGSSVPEQQITAYLSR	99.70%	56.7	31	48.2	2,102.04
ELF3-HFC elf4-2 rep 1	| Symbols: PHYB, HY3, OOP1 | phytochrome B | chr2:8140079-8144151 FORWARD LENGTH=1172	AT2G18790.1	TAIR10_20101214.fasta	129,333.90	100.00%	24	33	50	0.30%	36.60%	VIGAFCFLQIPSPELQQALAVQR	99.70%	50	28.4	1.51	2,586.38
ELF3-HFC elf4-2 rep 1	| Symbols: PHYB, HY3, OOP1 | phytochrome B | chr2:8140079-8144151 FORWARD LENGTH=1172	AT2G18790.1	TAIR10_20101214.fasta	129,333.90	100.00%	24	33	50	0.30%	36.60%	VVDGVVQPCR	98.90%	31.5	27.1	26.4	1,128.58
ELF3-HFC elf4-2 rep 1	| Symbols: PHYB, HY3, OOP1 | phytochrome B | chr2:8140079-8144151 FORWARD LENGTH=1172	AT2G18790.1	TAIR10_20101214.fasta	129,333.90	100.00%	24	33	50	0.30%	36.60%	YAPSQEWVEIHLSQLSK	99.70%	88.4	30.7	76.7	2,015.02
ELF3-HFC elf4-2 rep 1	| Symbols: PHYB, HY3, OOP1 | phytochrome B | chr2:8140079-8144151 FORWARD LENGTH=1172	AT2G18790.1	TAIR10_20101214.fasta	129,333.90	100.00%	24	33	50	0.30%	36.60%	YYPLGVAPSEVQIK	99.70%	59.9	29.5	50.2	1,563.84
ELF3-HFC elf4-2 rep 1	| Symbols: PHYC | phytochrome C | chr5:14008049-14011619 FORWARD LENGTH=1111	AT5G35840.1	TAIR10_20101214.fasta	123,724.60	100.00%	25	34	35	0.21%	31.40%	AAEVTGLAVEQAIGKPVSDLVEDDSVETVK	99.70%	76.6	30.5	76.3	3,069.58
ELF3-HFC elf4-2 rep 1	| Symbols: PHYC | phytochrome C | chr5:14008049-14011619 FORWARD LENGTH=1111	AT5G35840.1	TAIR10_20101214.fasta	123,724.60	100.00%	25	34	35	0.21%	31.40%	AISFLQDLLHSSGLSEDQK	99.70%	61	30.8	54.3	2,088.06
ELF3-HFC elf4-2 rep 1	| Symbols: PHYC | phytochrome C | chr5:14008049-14011619 FORWARD LENGTH=1111	AT5G35840.1	TAIR10_20101214.fasta	123,724.60	100.00%	25	34	35	0.21%	31.40%	AISFLQDLLHSSGLSEDQKR	99.70%	72.3	30.3	58.1	2,244.16
ELF3-HFC elf4-2 rep 1	| Symbols: PHYC | phytochrome C | chr5:14008049-14011619 FORWARD LENGTH=1111	AT5G35840.1	TAIR10_20101214.fasta	123,724.60	100.00%	25	34	35	0.21%	31.40%	AVDFGEISILNPITLHCR	99.70%	84.5	30	83.6	2,055.07
ELF3-HFC elf4-2 rep 1	| Symbols: PHYC | phytochrome C | chr5:14008049-14011619 FORWARD LENGTH=1111	AT5G35840.1	TAIR10_20101214.fasta	123,724.60	100.00%	25	34	35	0.21%	31.40%	CDGAALYYR	99.70%	50.5	25	41.2	1,088.48
ELF3-HFC elf4-2 rep 1	| Symbols: PHYC | phytochrome C | chr5:14008049-14011619 FORWARD LENGTH=1111	AT5G35840.1	TAIR10_20101214.fasta	123,724.60	100.00%	25	34	35	0.21%	31.40%	DGSFIEALLSANKR	99.70%	29.7	29.6	21.3	1,520.81
ELF3-HFC elf4-2 rep 1	| Symbols: PHYC | phytochrome C | chr5:14008049-14011619 FORWARD LENGTH=1111	AT5G35840.1	TAIR10_20101214.fasta	123,724.60	100.00%	25	34	35	0.21%	31.40%	DLIDWVLK	98.30%	41.6	28.4	17.8	1,001.57
ELF3-HFC elf4-2 rep 1	| Symbols: PHYC | phytochrome C | chr5:14008049-14011619 FORWARD LENGTH=1111	AT5G35840.1	TAIR10_20101214.fasta	123,724.60	100.00%	25	34	35	0.21%	31.40%	EAESAVLLK	99.70%	61.5	29.4	36	959.5408
ELF3-HFC elf4-2 rep 1	| Symbols: PHYC | phytochrome C | chr5:14008049-14011619 FORWARD LENGTH=1111	AT5G35840.1	TAIR10_20101214.fasta	123,724.60	100.00%	25	34	35	0.21%	31.40%	EALTIGTDVK	99.20%	29.1	29.8	22.2	1,046.57
ELF3-HFC elf4-2 rep 1	| Symbols: PHYC | phytochrome C | chr5:14008049-14011619 FORWARD LENGTH=1111	AT5G35840.1	TAIR10_20101214.fasta	123,724.60	100.00%	25	34	35	0.21%	31.40%	EGLGLHITQK	99.70%	51.4	25.4	37	1,095.62
ELF3-HFC elf4-2 rep 1	| Symbols: PHYC | phytochrome C | chr5:14008049-14011619 FORWARD LENGTH=1111	AT5G35840.1	TAIR10_20101214.fasta	123,724.60	100.00%	25	34	35	0.21%	31.40%	EVSELTGYDR	99.70%	48	27.8	37.1	1,168.55
ELF3-HFC elf4-2 rep 1	| Symbols: PHYC | phytochrome C | chr5:14008049-14011619 FORWARD LENGTH=1111	AT5G35840.1	TAIR10_20101214.fasta	123,724.60	100.00%	25	34	35	0.21%	31.40%	FHEDGHGEVIAECCR	99.70%	26.8	25	23.5	1,815.75
ELF3-HFC elf4-2 rep 1	| Symbols: PHYC | phytochrome C | chr5:14008049-14011619 FORWARD LENGTH=1111	AT5G35840.1	TAIR10_20101214.fasta	123,724.60	100.00%	25	34	35	0.21%	31.40%	IEEGLVIDLEPVSPDEVPVTAAGALR	99.70%	54.1	28.5	51.9	2,689.43
ELF3-HFC elf4-2 rep 1	| Symbols: PHYC | phytochrome C | chr5:14008049-14011619 FORWARD LENGTH=1111	AT5G35840.1	TAIR10_20101214.fasta	123,724.60	100.00%	25	34	35	0.21%	31.40%	IGFNAVISGQK	99.70%	72.2	28.5	56.8	1,133.63
ELF3-HFC elf4-2 rep 1	| Symbols: PHYC | phytochrome C | chr5:14008049-14011619 FORWARD LENGTH=1111	AT5G35840.1	TAIR10_20101214.fasta	123,724.60	100.00%	25	34	35	0.21%	31.40%	IIHPAPGLPEDLVR	99.70%	53.5	26	46.9	1,526.87
ELF3-HFC elf4-2 rep 1	| Symbols: PHYC | phytochrome C | chr5:14008049-14011619 FORWARD LENGTH=1111	AT5G35840.1	TAIR10_20101214.fasta	123,724.60	100.00%	25	34	35	0.21%	31.40%	ILLGEVFTTDDYGCCLK	99.70%	109	30.2	104	2,003.95
ELF3-HFC elf4-2 rep 1	| Symbols: PHYC | phytochrome C | chr5:14008049-14011619 FORWARD LENGTH=1111	AT5G35840.1	TAIR10_20101214.fasta	123,724.60	100.00%	25	34	35	0.21%	31.40%	LHGNFEESER	99.70%	45.1	25.8	30.1	1,217.55
ELF3-HFC elf4-2 rep 1	| Symbols: PHYC | phytochrome C | chr5:14008049-14011619 FORWARD LENGTH=1111	AT5G35840.1	TAIR10_20101214.fasta	123,724.60	100.00%	25	34	35	0.21%	31.40%	LIDTAAVPIFAVDASGVINGWNSK	99.70%	90.5	30.4	23.3	2,459.28
ELF3-HFC elf4-2 rep 1	| Symbols: PHYC | phytochrome C | chr5:14008049-14011619 FORWARD LENGTH=1111	AT5G35840.1	TAIR10_20101214.fasta	123,724.60	100.00%	25	34	35	0.21%	31.40%	LQQILSETLLSSIR	99.70%	113	25.3	107	1,600.93
ELF3-HFC elf4-2 rep 1	| Symbols: PHYC | phytochrome C | chr5:14008049-14011619 FORWARD LENGTH=1111	AT5G35840.1	TAIR10_20101214.fasta	123,724.60	100.00%	25	34	35	0.21%	31.40%	NMLALALEGSEER	98.40%	30.1	30.4	23	1,432.71
ELF3-HFC elf4-2 rep 1	| Symbols: PHYC | phytochrome C | chr5:14008049-14011619 FORWARD LENGTH=1111	AT5G35840.1	TAIR10_20101214.fasta	123,724.60	100.00%	25	34	35	0.21%	31.40%	SLSQPISLSGSTLR	99.70%	107	29.3	90.1	1,445.80
ELF3-HFC elf4-2 rep 1	| Symbols: PHYC | phytochrome C | chr5:14008049-14011619 FORWARD LENGTH=1111	AT5G35840.1	TAIR10_20101214.fasta	123,724.60	100.00%	25	34	35	0.21%	31.40%	SSPVELVVNTCCSR	99.70%	67	29.4	58.8	1,607.75
ELF3-HFC elf4-2 rep 1	| Symbols: PHYC | phytochrome C | chr5:14008049-14011619 FORWARD LENGTH=1111	AT5G35840.1	TAIR10_20101214.fasta	123,724.60	100.00%	25	34	35	0.21%	31.40%	SSSKPFYAILHR	99.70%	50.8	29	40.5	1,405.76
ELF3-HFC elf4-2 rep 1	| Symbols: PHYC | phytochrome C | chr5:14008049-14011619 FORWARD LENGTH=1111	AT5G35840.1	TAIR10_20101214.fasta	123,724.60	100.00%	25	34	35	0.21%	31.40%	TVVDVPLVDNR	99.70%	45.9	26.3	36.6	1,226.67
ELF3-HFC elf4-2 rep 1	| Symbols: PHYC | phytochrome C | chr5:14008049-14011619 FORWARD LENGTH=1111	AT5G35840.1	TAIR10_20101214.fasta	123,724.60	100.00%	25	34	35	0.21%	31.40%	YACEFLTQVFGVQINK	99.70%	71.9	30.5	57.6	1,916.96
ELF3-HFC elf4-2 rep 1	| Symbols: PHYE | phytochrome E | chr4:10042312-10045948 REVERSE LENGTH=1112	AT4G18130.1	TAIR10_20101214.fasta	122,519.10	100.00%	9	9	11	0.07%	14.00%	AALESLLCK	99.70%	33	30.1	24.7	1,004.54
ELF3-HFC elf4-2 rep 1	| Symbols: PHYE | phytochrome E | chr4:10042312-10045948 REVERSE LENGTH=1112	AT4G18130.1	TAIR10_20101214.fasta	122,519.10	100.00%	9	9	11	0.07%	14.00%	AASFTEISLLNPVLVHSR	99.70%	60.5	27	59.4	1,954.08
ELF3-HFC elf4-2 rep 1	| Symbols: PHYE | phytochrome E | chr4:10042312-10045948 REVERSE LENGTH=1112	AT4G18130.1	TAIR10_20101214.fasta	122,519.10	100.00%	9	9	11	0.07%	14.00%	ESAQSLNELTYVR	99.70%	71.7	30.5	59.1	1,509.75
ELF3-HFC elf4-2 rep 1	| Symbols: PHYE | phytochrome E | chr4:10042312-10045948 REVERSE LENGTH=1112	AT4G18130.1	TAIR10_20101214.fasta	122,519.10	100.00%	9	9	11	0.07%	14.00%	ESFTSSRPVLSGNGVAR	99.50%	29.9	31.3	19.6	1,764.89
ELF3-HFC elf4-2 rep 1	| Symbols: PHYE | phytochrome E | chr4:10042312-10045948 REVERSE LENGTH=1112	AT4G18130.1	TAIR10_20101214.fasta	122,519.10	100.00%	9	9	11	0.07%	14.00%	ILGLSDNSSDFLGLLSLPSTSHSGEFDK	99.70%	70	32	53.9	2,936.45
ELF3-HFC elf4-2 rep 1	| Symbols: PHYE | phytochrome E | chr4:10042312-10045948 REVERSE LENGTH=1112	AT4G18130.1	TAIR10_20101214.fasta	122,519.10	100.00%	9	9	11	0.07%	14.00%	LQSLPGGDIGALCDTVVEDVQR	99.70%	55.3	31.8	53.1	2,342.17
ELF3-HFC elf4-2 rep 1	| Symbols: PHYE | phytochrome E | chr4:10042312-10045948 REVERSE LENGTH=1112	AT4G18130.1	TAIR10_20101214.fasta	122,519.10	100.00%	9	9	11	0.07%	14.00%	NIVNHAPFPNSWVGISISPGQELSR	99.60%	32.3	31.1	21.8	2,719.40
ELF3-HFC elf4-2 rep 1	| Symbols: PHYE | phytochrome E | chr4:10042312-10045948 REVERSE LENGTH=1112	AT4G18130.1	TAIR10_20101214.fasta	122,519.10	100.00%	9	9	11	0.07%	14.00%	QFLETSDACEK	98.90%	27.1	25.9	16.5	1,327.58
ELF3-HFC elf4-2 rep 1	| Symbols: PHYE | phytochrome E | chr4:10042312-10045948 REVERSE LENGTH=1112	AT4G18130.1	TAIR10_20101214.fasta	122,519.10	100.00%	9	9	11	0.07%	14.00%	TLFTPSSGASLSK	99.30%	32.1	29.1	16.2	1,295.68
ELF3-HFC elf4-2 rep 1	| Symbols: RD21, RD21A | Granulin repeat cysteine protease family protein | chr1:17283139-17285609 REVERSE LENGTH=462	AT1G47128.1	TAIR10_20101214.fasta	50,967.20	100.00%	4	4	4	0.02%	9.74%	AVAHQPISIAIEAGGR	99.70%	28.5	28.4	3.55	1,589.88
ELF3-HFC elf4-2 rep 1	| Symbols: RD21, RD21A | Granulin repeat cysteine protease family protein | chr1:17283139-17285609 REVERSE LENGTH=462	AT1G47128.1	TAIR10_20101214.fasta	50,967.20	100.00%	4	4	4	0.02%	9.74%	SWGESGYLR	99.70%	30	27.6	17	1,054.50
ELF3-HFC elf4-2 rep 1	| Symbols: RD21, RD21A | Granulin repeat cysteine protease family protein | chr1:17283139-17285609 REVERSE LENGTH=462	AT1G47128.1	TAIR10_20101214.fasta	50,967.20	100.00%	4	4	4	0.02%	9.74%	VVTIDSYEDVPTYSEESLK	99.70%	49.2	30.9	46.5	2,174.04
ELF3-HFC elf4-2 rep 1	| Symbols: RD21, RD21A | Granulin repeat cysteine protease family protein | chr1:17283139-17285609 REVERSE LENGTH=462	AT1G47128.1	TAIR10_20101214.fasta	50,967.20	100.00%	4	4	4	0.02%	9.74%	VVTIDSYEDVPTYSEESLKK	98.00%	29.8	31.2	25.6	2,302.13
ELF3-HFC elf4-2 rep 1	| Symbols: SPA1 | SPA (suppressor of phyA-105) protein family | chr2:19022572-19026821 REVERSE LENGTH=1029	AT2G46340.1	TAIR10_20101214.fasta	114,689.10	100.00%	4	5	6	0.04%	8.84%	NDLESDVDEDLNR	99.70%	70.6	25.4	60.9	1,533.67
ELF3-HFC elf4-2 rep 1	| Symbols: SPA1 | SPA (suppressor of phyA-105) protein family | chr2:19022572-19026821 REVERSE LENGTH=1029	AT2G46340.1	TAIR10_20101214.fasta	114,689.10	100.00%	4	5	6	0.04%	8.84%	NQETPPEFVSDQDLGSK	99.70%	42.2	29.1	34.3	1,890.87
ELF3-HFC elf4-2 rep 1	| Symbols: SPA1 | SPA (suppressor of phyA-105) protein family | chr2:19022572-19026821 REVERSE LENGTH=1029	AT2G46340.1	TAIR10_20101214.fasta	114,689.10	100.00%	4	5	6	0.04%	8.84%	NYLASTDYDGVVQIWDAGTGQGFSQYTEHQK	99.70%	98	30.1	2.12	3,479.57
ELF3-HFC elf4-2 rep 1	| Symbols: SPA1 | SPA (suppressor of phyA-105) protein family | chr2:19022572-19026821 REVERSE LENGTH=1029	AT2G46340.1	TAIR10_20101214.fasta	114,689.10	100.00%	4	5	6	0.04%	8.84%	SGDLLNSASVVCSLSFDPDEEHIAAAGISK	99.70%	73.8	31.4	62.1	3,089.47
ELF3-HFC elf4-2 rep 1	| Symbols: TIC | time for coffee | chr3:7913181-7918879 FORWARD LENGTH=1550	AT3G22380.1,AT3G22380.2	TAIR10_20101214.fasta	164,452.80	100.00%	3	3	4	0.02%	3.48%	ENATQHSETVGEDSPSTADSR	99.70%	49.2	26.7	44.6	2,217.95
ELF3-HFC elf4-2 rep 1	| Symbols: TIC | time for coffee | chr3:7913181-7918879 FORWARD LENGTH=1550	AT3G22380.1,AT3G22380.2	TAIR10_20101214.fasta	164,452.80	100.00%	3	3	4	0.02%	3.48%	QEAAGNDLTEAAK	99.70%	60.5	28.1	48.8	1,317.63
ELF3-HFC elf4-2 rep 1	| Symbols: TIC | time for coffee | chr3:7913181-7918879 FORWARD LENGTH=1550	AT3G22380.1,AT3G22380.2	TAIR10_20101214.fasta	164,452.80	100.00%	3	3	4	0.02%	3.48%	SSGSGEGNSSVLDSIIPLTR	99.70%	49.6	31.4	45.9	1,975.99
ELF3-HFC elf4-2 rep 1	| Symbols: TZP | zinc knuckle (CCHC-type) family protein | chr5:17527261-17530207 FORWARD LENGTH=831	AT5G43630.1	TAIR10_20101214.fasta	90,615.60	100.00%	13	14	15	0.09%	21.10%	AVFDAVQVLR	99.70%	80.1	28.8	59.6	1,117.64
ELF3-HFC elf4-2 rep 1	| Symbols: TZP | zinc knuckle (CCHC-type) family protein | chr5:17527261-17530207 FORWARD LENGTH=831	AT5G43630.1	TAIR10_20101214.fasta	90,615.60	100.00%	13	14	15	0.09%	21.10%	AWWQSAGK	99.00%	52.6	28.5	35.9	933.4577
ELF3-HFC elf4-2 rep 1	| Symbols: TZP | zinc knuckle (CCHC-type) family protein | chr5:17527261-17530207 FORWARD LENGTH=831	AT5G43630.1	TAIR10_20101214.fasta	90,615.60	100.00%	13	14	15	0.09%	21.10%	DLICFYCGK	99.70%	38.4	25	35.7	1,175.52
ELF3-HFC elf4-2 rep 1	| Symbols: TZP | zinc knuckle (CCHC-type) family protein | chr5:17527261-17530207 FORWARD LENGTH=831	AT5G43630.1	TAIR10_20101214.fasta	90,615.60	100.00%	13	14	15	0.09%	21.10%	DLVQNISVR	99.70%	51.7	28.7	34.7	1,043.58
ELF3-HFC elf4-2 rep 1	| Symbols: TZP | zinc knuckle (CCHC-type) family protein | chr5:17527261-17530207 FORWARD LENGTH=831	AT5G43630.1	TAIR10_20101214.fasta	90,615.60	100.00%	13	14	15	0.09%	21.10%	GNEEDNSPFVALTTTSNANGHGQVNAIVDQQQLSPCCVK	99.70%	38.2	31	0	4,200.92
ELF3-HFC elf4-2 rep 1	| Symbols: TZP | zinc knuckle (CCHC-type) family protein | chr5:17527261-17530207 FORWARD LENGTH=831	AT5G43630.1	TAIR10_20101214.fasta	90,615.60	100.00%	13	14	15	0.09%	21.10%	GVTCLVESQFISNQDFLEEELK	99.70%	45.1	31.7	38.7	2,585.24
ELF3-HFC elf4-2 rep 1	| Symbols: TZP | zinc knuckle (CCHC-type) family protein | chr5:17527261-17530207 FORWARD LENGTH=831	AT5G43630.1	TAIR10_20101214.fasta	90,615.60	100.00%	13	14	15	0.09%	21.10%	KRPGFEEQLIFGSK	99.70%	65	28.3	61	1,635.89
ELF3-HFC elf4-2 rep 1	| Symbols: TZP | zinc knuckle (CCHC-type) family protein | chr5:17527261-17530207 FORWARD LENGTH=831	AT5G43630.1	TAIR10_20101214.fasta	90,615.60	100.00%	13	14	15	0.09%	21.10%	LPISGFTDVPR	99.70%	71.8	29.4	63.4	1,201.66
ELF3-HFC elf4-2 rep 1	| Symbols: TZP | zinc knuckle (CCHC-type) family protein | chr5:17527261-17530207 FORWARD LENGTH=831	AT5G43630.1	TAIR10_20101214.fasta	90,615.60	100.00%	13	14	15	0.09%	21.10%	SVSGLEGFFLR	99.70%	66.6	27.8	46.9	1,211.64
ELF3-HFC elf4-2 rep 1	| Symbols: TZP | zinc knuckle (CCHC-type) family protein | chr5:17527261-17530207 FORWARD LENGTH=831	AT5G43630.1	TAIR10_20101214.fasta	90,615.60	100.00%	13	14	15	0.09%	21.10%	TLNQECLGSTSK	99.70%	68.9	29.5	63.1	1,337.64
ELF3-HFC elf4-2 rep 1	| Symbols: TZP | zinc knuckle (CCHC-type) family protein | chr5:17527261-17530207 FORWARD LENGTH=831	AT5G43630.1	TAIR10_20101214.fasta	90,615.60	100.00%	13	14	15	0.09%	21.10%	VSPCLTNR	98.50%	30.2	28.9	17.8	946.4775
ELF3-HFC elf4-2 rep 1	| Symbols: TZP | zinc knuckle (CCHC-type) family protein | chr5:17527261-17530207 FORWARD LENGTH=831	AT5G43630.1	TAIR10_20101214.fasta	90,615.60	100.00%	13	14	15	0.09%	21.10%	VVFNQTSK	98.20%	25.1	28.5	12.9	922.4992
ELF3-HFC elf4-2 rep 1	| Symbols: TZP | zinc knuckle (CCHC-type) family protein | chr5:17527261-17530207 FORWARD LENGTH=831	AT5G43630.1	TAIR10_20101214.fasta	90,615.60	100.00%	13	14	15	0.09%	21.10%	WEEGLGGTGYYVAR	99.70%	99.2	29.1	90.3	1,557.73
ELF3-HFC elf4-2 rep 2	| Symbols: | BED zinc finger ;hAT family dimerisation domain | chr3:14321838-14323928 FORWARD LENGTH=696	AT3G42170.1	TAIR10_20101214.fasta	78,815.20	100.00%	5	5	5	0.03%	9.63%	AIAGEDPFVTGIAK	99.70%	70.6	30.2	57.1	1,388.74
ELF3-HFC elf4-2 rep 2	| Symbols: | BED zinc finger ;hAT family dimerisation domain | chr3:14321838-14323928 FORWARD LENGTH=696	AT3G42170.1	TAIR10_20101214.fasta	78,815.20	100.00%	5	5	5	0.03%	9.63%	EVFSCLDTADPDYK	99.70%	47.5	25.5	36.7	1,659.72
ELF3-HFC elf4-2 rep 2	| Symbols: | BED zinc finger ;hAT family dimerisation domain | chr3:14321838-14323928 FORWARD LENGTH=696	AT3G42170.1	TAIR10_20101214.fasta	78,815.20	100.00%	5	5	5	0.03%	9.63%	GCNQSFAYSNGNK	99.70%	34.7	25	5.47	1,447.59
ELF3-HFC elf4-2 rep 2	| Symbols: | BED zinc finger ;hAT family dimerisation domain | chr3:14321838-14323928 FORWARD LENGTH=696	AT3G42170.1	TAIR10_20101214.fasta	78,815.20	100.00%	5	5	5	0.03%	9.63%	SQNNASPYVAFNQDK	99.70%	55.8	29	55.7	1,682.78
ELF3-HFC elf4-2 rep 2	| Symbols: | BED zinc finger ;hAT family dimerisation domain | chr3:14321838-14323928 FORWARD LENGTH=696	AT3G42170.1	TAIR10_20101214.fasta	78,815.20	100.00%	5	5	5	0.03%	9.63%	VQEFDVLDWWK	99.40%	35	30.4	9.97	1,464.72
ELF3-HFC elf4-2 rep 2	| Symbols: | C2 calcium/lipid-binding plant phosphoribosyltransferase family protein | chr5:2127200-2129584 REVERSE LENGTH=794	AT5G06850.1	TAIR10_20101214.fasta	91,004.50	100.00%	2	2	2	0.01%	2.90%	VNVIEAQDVEPSDR	99.70%	63.2	29.5	57.8	1,570.77
ELF3-HFC elf4-2 rep 2	| Symbols: | C2 calcium/lipid-binding plant phosphoribosyltransferase family protein | chr5:2127200-2129584 REVERSE LENGTH=794	AT5G06850.1	TAIR10_20101214.fasta	91,004.50	100.00%	2	2	2	0.01%	2.90%	VQVGNQILK	99.70%	34.8	25	24.1	998.5994
ELF3-HFC elf4-2 rep 2	| Symbols: | F-box family protein | chr2:7074940-7079181 FORWARD LENGTH=727	AT2G16365.4	TAIR10_20101214.fasta	82,716.20	100.00%	11	11	14	0.08%	21.60%	ALSGLNPYGLR	99.70%	63.6	29.9	44.2	1,160.64
ELF3-HFC elf4-2 rep 2	| Symbols: | F-box family protein | chr2:7074940-7079181 FORWARD LENGTH=727	AT2G16365.4	TAIR10_20101214.fasta	82,716.20	100.00%	11	11	14	0.08%	21.60%	ENNNLFLEILK	99.70%	64.2	29.6	50.3	1,346.73
ELF3-HFC elf4-2 rep 2	| Symbols: | F-box family protein | chr2:7074940-7079181 FORWARD LENGTH=727	AT2G16365.4	TAIR10_20101214.fasta	82,716.20	100.00%	11	11	14	0.08%	21.60%	ETTTTTTTKPSFHEDVGSSSR	99.70%	55.2	30.6	54.1	2,269.06
ELF3-HFC elf4-2 rep 2	| Symbols: | F-box family protein | chr2:7074940-7079181 FORWARD LENGTH=727	AT2G16365.4	TAIR10_20101214.fasta	82,716.20	100.00%	11	11	14	0.08%	21.60%	IYATVDSVNLPK	99.70%	83.2	28.9	66.3	1,319.72
ELF3-HFC elf4-2 rep 2	| Symbols: | F-box family protein | chr2:7074940-7079181 FORWARD LENGTH=727	AT2G16365.4	TAIR10_20101214.fasta	82,716.20	100.00%	11	11	14	0.08%	21.60%	LDFPIQEK	99.00%	39.2	30.3	9.74	989.5304
ELF3-HFC elf4-2 rep 2	| Symbols: | F-box family protein | chr2:7074940-7079181 FORWARD LENGTH=727	AT2G16365.4	TAIR10_20101214.fasta	82,716.20	100.00%	11	11	14	0.08%	21.60%	LQTIHLSGSISSSSTK	99.70%	37	30.4	30.6	1,645.88
ELF3-HFC elf4-2 rep 2	| Symbols: | F-box family protein | chr2:7074940-7079181 FORWARD LENGTH=727	AT2G16365.4	TAIR10_20101214.fasta	82,716.20	100.00%	11	11	14	0.08%	21.60%	NSSVNPYESAWLGR	99.70%	91.7	30.2	78.9	1,579.75
ELF3-HFC elf4-2 rep 2	| Symbols: | F-box family protein | chr2:7074940-7079181 FORWARD LENGTH=727	AT2G16365.4	TAIR10_20101214.fasta	82,716.20	100.00%	11	11	14	0.08%	21.60%	QGGDDITLLHPWIQR	98.70%	26.5	30.7	15.9	1,748.91
ELF3-HFC elf4-2 rep 2	| Symbols: | F-box family protein | chr2:7074940-7079181 FORWARD LENGTH=727	AT2G16365.4	TAIR10_20101214.fasta	82,716.20	100.00%	11	11	14	0.08%	21.60%	TTQNLLELIRPVR	99.70%	41.8	25	28.9	1,552.92
ELF3-HFC elf4-2 rep 2	| Symbols: | F-box family protein | chr2:7074940-7079181 FORWARD LENGTH=727	AT2G16365.4	TAIR10_20101214.fasta	82,716.20	100.00%	11	11	14	0.08%	21.60%	TTSTDQPTGQEASFEPESHKEFEK	99.70%	54.7	29.1	47	2,710.21
ELF3-HFC elf4-2 rep 2	| Symbols: | F-box family protein | chr2:7074940-7079181 FORWARD LENGTH=727	AT2G16365.4	TAIR10_20101214.fasta	82,716.20	100.00%	11	11	14	0.08%	21.60%	VRLEPEVEASSR	99.70%	47.2	29.1	40.5	1,371.72
ELF3-HFC elf4-2 rep 2	| Symbols: | Protein kinase family protein | chr3:1014412-1018244 REVERSE LENGTH=701	AT3G03940.1	TAIR10_20101214.fasta	77,995.40	99.80%	1	1	2	0.01%	3.14%	ITDETQETLR	99.60%	36.5	30.2	0	1,205.60
ELF3-HFC elf4-2 rep 2	| Symbols: | Protein kinase family protein | chr3:1014412-1018244 REVERSE LENGTH=701	AT3G03940.1	TAIR10_20101214.fasta	77,995.40	99.80%	1	1	2	0.01%	3.14%	LLINLEEDEQPR	99.70%	56.1	29.4	50.2	1,468.76
ELF3-HFC elf4-2 rep 2	| Symbols: ATAN11, LWD1 | Transducin/WD40 repeat-like superfamily protein | chr1:4395114-4396154 REVERSE LENGTH=346	AT1G12910.1	TAIR10_20101214.fasta	39,089.30	100.00%	2	2	4	0.02%	10.70%	DKEHSTIIYESSEPDTPLVR	99.70%	43.3	31.7	35.2	2,316.14
ELF3-HFC elf4-2 rep 2	| Symbols: ATAN11, LWD1 | Transducin/WD40 repeat-like superfamily protein | chr1:4395114-4396154 REVERSE LENGTH=346	AT1G12910.1	TAIR10_20101214.fasta	39,089.30	100.00%	2	2	4	0.02%	10.70%	IGTSSTDTTCTIWDIER	99.70%	71.3	29.7	66.3	1,955.90
ELF3-HFC elf4-2 rep 2	| Symbols: ATARCA, RACK1A_AT, RACK1A | Transducin/WD40 repeat-like superfamily protein | chr1:6222325-6223901 FORWARD LENGTH=327	AT1G18080.1	TAIR10_20101214.fasta	35,748.00	100.00%	2	2	2	0.01%	8.56%	FSPNTLQPTIVSASWDK	98.60%	30.9	31.2	22.4	1,890.96
ELF3-HFC elf4-2 rep 2	| Symbols: ATARCA, RACK1A_AT, RACK1A | Transducin/WD40 repeat-like superfamily protein | chr1:6222325-6223901 FORWARD LENGTH=327	AT1G18080.1	TAIR10_20101214.fasta	35,748.00	100.00%	2	2	2	0.01%	8.56%	LWDLAAGVSTR	99.70%	61.1	30	47.5	1,188.64
ELF3-HFC elf4-2 rep 2	| Symbols: COP1, ATCOP1, DET340, FUS1, EMB168 | Transducin/WD40 repeat-like superfamily protein | chr2:13978000-13983282 FORWARD LENGTH=675	AT2G32950.1	TAIR10_20101214.fasta	76,188.00	100.00%	7	7	7	0.04%	14.70%	AWSVDFSR	98.70%	27	27.2	17.5	967.4631
ELF3-HFC elf4-2 rep 2	| Symbols: COP1, ATCOP1, DET340, FUS1, EMB168 | Transducin/WD40 repeat-like superfamily protein | chr2:13978000-13983282 FORWARD LENGTH=675	AT2G32950.1	TAIR10_20101214.fasta	76,188.00	100.00%	7	7	7	0.04%	14.70%	EGYSNGLADFQSVLTTFTR	99.70%	52.9	30.7	25	2,107.00
ELF3-HFC elf4-2 rep 2	| Symbols: COP1, ATCOP1, DET340, FUS1, EMB168 | Transducin/WD40 repeat-like superfamily protein | chr2:13978000-13983282 FORWARD LENGTH=675	AT2G32950.1	TAIR10_20101214.fasta	76,188.00	100.00%	7	7	7	0.04%	14.70%	FLSNNELASASTDSTLR	99.70%	108	30.5	94.7	1,825.89
ELF3-HFC elf4-2 rep 2	| Symbols: COP1, ATCOP1, DET340, FUS1, EMB168 | Transducin/WD40 repeat-like superfamily protein | chr2:13978000-13983282 FORWARD LENGTH=675	AT2G32950.1	TAIR10_20101214.fasta	76,188.00	100.00%	7	7	7	0.04%	14.70%	GGNFVGNYQNK	99.70%	39	28.4	26.9	1,197.56
ELF3-HFC elf4-2 rep 2	| Symbols: COP1, ATCOP1, DET340, FUS1, EMB168 | Transducin/WD40 repeat-like superfamily protein | chr2:13978000-13983282 FORWARD LENGTH=675	AT2G32950.1	TAIR10_20101214.fasta	76,188.00	100.00%	7	7	7	0.04%	14.70%	IHAQFNDLQECYLQK	99.60%	30.7	30.7	24	1,906.91
ELF3-HFC elf4-2 rep 2	| Symbols: COP1, ATCOP1, DET340, FUS1, EMB168 | Transducin/WD40 repeat-like superfamily protein | chr2:13978000-13983282 FORWARD LENGTH=675	AT2G32950.1	TAIR10_20101214.fasta	76,188.00	100.00%	7	7	7	0.04%	14.70%	NHIASSDYEGIVTVWDVTTR	99.70%	49	31.1	34.4	2,263.10
ELF3-HFC elf4-2 rep 2	| Symbols: COP1, ATCOP1, DET340, FUS1, EMB168 | Transducin/WD40 repeat-like superfamily protein | chr2:13978000-13983282 FORWARD LENGTH=675	AT2G32950.1	TAIR10_20101214.fasta	76,188.00	100.00%	7	7	7	0.04%	14.70%	TASPLDQFR	97.70%	30.8	30.1	8.74	1,034.53
ELF3-HFC elf4-2 rep 2	| Symbols: ELF3, PYK20 | hydroxyproline-rich glycoprotein family protein | chr2:11059459-11063178 FORWARD LENGTH=695	AT2G25930.1	TAIR10_20101214.fasta	77,204.80	100.00%	20	32	41	0.25%	41.30%	EEEDFAVPVYINSR	99.70%	62.6	30.2	51.4	1,667.79
ELF3-HFC elf4-2 rep 2	| Symbols: ELF3, PYK20 | hydroxyproline-rich glycoprotein family protein | chr2:11059459-11063178 FORWARD LENGTH=695	AT2G25930.1	TAIR10_20101214.fasta	77,204.80	100.00%	20	32	41	0.25%	41.30%	FQEVNQTGSK	99.70%	63	29.4	43.9	1,137.55
ELF3-HFC elf4-2 rep 2	| Symbols: ELF3, PYK20 | hydroxyproline-rich glycoprotein family protein | chr2:11059459-11063178 FORWARD LENGTH=695	AT2G25930.1	TAIR10_20101214.fasta	77,204.80	100.00%	20	32	41	0.25%	41.30%	IFQSIQEER	99.70%	60.2	30.2	28.1	1,149.59
ELF3-HFC elf4-2 rep 2	| Symbols: ELF3, PYK20 | hydroxyproline-rich glycoprotein family protein | chr2:11059459-11063178 FORWARD LENGTH=695	AT2G25930.1	TAIR10_20101214.fasta	77,204.80	100.00%	20	32	41	0.25%	41.30%	KAIANQQR	99.00%	40.9	25.8	13.9	928.5323
ELF3-HFC elf4-2 rep 2	| Symbols: ELF3, PYK20 | hydroxyproline-rich glycoprotein family protein | chr2:11059459-11063178 FORWARD LENGTH=695	AT2G25930.1	TAIR10_20101214.fasta	77,204.80	100.00%	20	32	41	0.25%	41.30%	KLLPSEFLVKPPLPHVVVK	99.70%	32.8	25	32.7	2,140.33
ELF3-HFC elf4-2 rep 2	| Symbols: ELF3, PYK20 | hydroxyproline-rich glycoprotein family protein | chr2:11059459-11063178 FORWARD LENGTH=695	AT2G25930.1	TAIR10_20101214.fasta	77,204.80	100.00%	20	32	41	0.25%	41.30%	LASENAAR	99.00%	46.7	29.1	21.3	831.4318
ELF3-HFC elf4-2 rep 2	| Symbols: ELF3, PYK20 | hydroxyproline-rich glycoprotein family protein | chr2:11059459-11063178 FORWARD LENGTH=695	AT2G25930.1	TAIR10_20101214.fasta	77,204.80	100.00%	20	32	41	0.25%	41.30%	LHVNDADK	99.00%	34.5	27.1	24.3	911.4582
ELF3-HFC elf4-2 rep 2	| Symbols: ELF3, PYK20 | hydroxyproline-rich glycoprotein family protein | chr2:11059459-11063178 FORWARD LENGTH=695	AT2G25930.1	TAIR10_20101214.fasta	77,204.80	100.00%	20	32	41	0.25%	41.30%	LHVNDADKGGPR	99.70%	46.4	30.7	36.8	1,278.66
ELF3-HFC elf4-2 rep 2	| Symbols: ELF3, PYK20 | hydroxyproline-rich glycoprotein family protein | chr2:11059459-11063178 FORWARD LENGTH=695	AT2G25930.1	TAIR10_20101214.fasta	77,204.80	100.00%	20	32	41	0.25%	41.30%	LIAASPDLLLDEISFLGK	99.70%	88.9	25.4	85	1,915.08
ELF3-HFC elf4-2 rep 2	| Symbols: ELF3, PYK20 | hydroxyproline-rich glycoprotein family protein | chr2:11059459-11063178 FORWARD LENGTH=695	AT2G25930.1	TAIR10_20101214.fasta	77,204.80	100.00%	20	32	41	0.25%	41.30%	LKDTDNGAESHLATENHSQEGHGSPEDIDNDR	99.70%	52.7	26.9	6.09	3,489.50
ELF3-HFC elf4-2 rep 2	| Symbols: ELF3, PYK20 | hydroxyproline-rich glycoprotein family protein | chr2:11059459-11063178 FORWARD LENGTH=695	AT2G25930.1	TAIR10_20101214.fasta	77,204.80	100.00%	20	32	41	0.25%	41.30%	MALYEQLSIPSQR	99.00%	34.4	31.1	21.2	1,535.79
ELF3-HFC elf4-2 rep 2	| Symbols: ELF3, PYK20 | hydroxyproline-rich glycoprotein family protein | chr2:11059459-11063178 FORWARD LENGTH=695	AT2G25930.1	TAIR10_20101214.fasta	77,204.80	100.00%	20	32	41	0.25%	41.30%	NLSVQHLDSSAANQATEK	99.70%	103	30.9	93	1,912.94
ELF3-HFC elf4-2 rep 2	| Symbols: ELF3, PYK20 | hydroxyproline-rich glycoprotein family protein | chr2:11059459-11063178 FORWARD LENGTH=695	AT2G25930.1	TAIR10_20101214.fasta	77,204.80	100.00%	20	32	41	0.25%	41.30%	QGSTGSSPSGPQGISGSK	99.70%	73.5	29.3	66.2	1,618.77
ELF3-HFC elf4-2 rep 2	| Symbols: ELF3, PYK20 | hydroxyproline-rich glycoprotein family protein | chr2:11059459-11063178 FORWARD LENGTH=695	AT2G25930.1	TAIR10_20101214.fasta	77,204.80	100.00%	20	32	41	0.25%	41.30%	QNVCLATCSKPEVR	99.70%	70.1	30.2	56.7	1,661.81
ELF3-HFC elf4-2 rep 2	| Symbols: ELF3, PYK20 | hydroxyproline-rich glycoprotein family protein | chr2:11059459-11063178 FORWARD LENGTH=695	AT2G25930.1	TAIR10_20101214.fasta	77,204.80	100.00%	20	32	41	0.25%	41.30%	SDNEPAPQQQQQPTK	99.70%	59.5	29.8	52	1,695.79
ELF3-HFC elf4-2 rep 2	| Symbols: ELF3, PYK20 | hydroxyproline-rich glycoprotein family protein | chr2:11059459-11063178 FORWARD LENGTH=695	AT2G25930.1	TAIR10_20101214.fasta	77,204.80	100.00%	20	32	41	0.25%	41.30%	SFRPFAAVDEDSNINNAPEQTMTTTTTTTR	99.70%	51.5	31.1	46.8	3,316.54
ELF3-HFC elf4-2 rep 2	| Symbols: ELF3, PYK20 | hydroxyproline-rich glycoprotein family protein | chr2:11059459-11063178 FORWARD LENGTH=695	AT2G25930.1	TAIR10_20101214.fasta	77,204.80	100.00%	20	32	41	0.25%	41.30%	SNNTSTLVHPGPSSQPCGVER	99.70%	42.8	30.5	39.3	2,224.04
ELF3-HFC elf4-2 rep 2	| Symbols: ELF3, PYK20 | hydroxyproline-rich glycoprotein family protein | chr2:11059459-11063178 FORWARD LENGTH=695	AT2G25930.1	TAIR10_20101214.fasta	77,204.80	100.00%	20	32	41	0.25%	41.30%	TTVTQTTR	99.00%	31.4	28.4	18	907.4844
ELF3-HFC elf4-2 rep 2	| Symbols: ELF3, PYK20 | hydroxyproline-rich glycoprotein family protein | chr2:11059459-11063178 FORWARD LENGTH=695	AT2G25930.1	TAIR10_20101214.fasta	77,204.80	100.00%	20	32	41	0.25%	41.30%	VFAVQLFELHR	99.70%	36	28.4	23.7	1,358.76
ELF3-HFC elf4-2 rep 2	| Symbols: ELF3, PYK20 | hydroxyproline-rich glycoprotein family protein | chr2:11059459-11063178 FORWARD LENGTH=695	AT2G25930.1	TAIR10_20101214.fasta	77,204.80	100.00%	20	32	41	0.25%	41.30%	VNDYNASLR	99.70%	72.4	29.2	57.2	1,051.52
ELF3-HFC elf4-2 rep 2	| Symbols: ELF4-L2 | ELF4-like 2 | chr1:27344988-27345347 FORWARD LENGTH=119	AT1G72630.1	TAIR10_20101214.fasta	13,409.00	100.00%	3	3	5	0.03%	47.10%	LLINEINQNHESK	99.70%	65.8	30.1	58.1	1,551.81
ELF3-HFC elf4-2 rep 2	| Symbols: ELF4-L2 | ELF4-like 2 | chr1:27344988-27345347 FORWARD LENGTH=119	AT1G72630.1	TAIR10_20101214.fasta	13,409.00	100.00%	3	3	5	0.03%	47.10%	SFVQVQDILDQNR	99.70%	66.6	30.9	57.2	1,561.80
ELF3-HFC elf4-2 rep 2	| Symbols: ELF4-L2 | ELF4-like 2 | chr1:27344988-27345347 FORWARD LENGTH=119	AT1G72630.1	TAIR10_20101214.fasta	13,409.00	100.00%	3	3	5	0.03%	47.10%	SVDASSEGESTGTLK	99.70%	107	29	95.6	1,467.68
ELF3-HFC elf4-2 rep 2	| Symbols: ELF4-L2 | ELF4-like 2 | chr1:27344988-27345347 FORWARD LENGTH=119	AT1G72630.1	TAIR10_20101214.fasta	13,409.00	100.00%	3	3	5	0.03%	47.10%	TVASLYGDLSHSFAR	99.70%	45.3	30.3	30.2	1,623.81
ELF3-HFC elf4-2 rep 2	| Symbols: ELF4-L3 | ELF4-like 3 | chr2:2459336-2459665 FORWARD LENGTH=109	AT2G06255.1	TAIR10_20101214.fasta	12,311.00	100.00%	2	3	5	0.03%	37.60%	LLINEINQNHESK	99.70%	65.8	30.1	58.1	1,551.81
ELF3-HFC elf4-2 rep 2	| Symbols: ELF4-L3 | ELF4-like 3 | chr2:2459336-2459665 FORWARD LENGTH=109	AT2G06255.1	TAIR10_20101214.fasta	12,311.00	100.00%	2	3	5	0.03%	37.60%	RVAHLYVDLSNNFSK	99.70%	41.1	30.5	32.9	1,762.92
ELF3-HFC elf4-2 rep 2	| Symbols: ELF4-L3 | ELF4-like 3 | chr2:2459336-2459665 FORWARD LENGTH=109	AT2G06255.1	TAIR10_20101214.fasta	12,311.00	100.00%	2	3	5	0.03%	37.60%	SFVQVQNILDHNR	99.70%	85.2	29.4	70.7	1,569.81
ELF3-HFC elf4-2 rep 2	| Symbols: ELF4-L4 | ELF4-like 4 | chr1:5997932-5998276 FORWARD LENGTH=114	AT1G17455.1,AT1G17455.2	TAIR10_20101214.fasta	12,663.30	100.00%	2	2	4	0.02%	36.00%	LLINEINQNHESK	99.70%	65.8	30.1	58.1	1,551.81
ELF3-HFC elf4-2 rep 2	| Symbols: ELF4-L4 | ELF4-like 4 | chr1:5997932-5998276 FORWARD LENGTH=114	AT1G17455.1,AT1G17455.2	TAIR10_20101214.fasta	12,663.30	100.00%	2	2	4	0.02%	36.00%	SFVDVQDILDQNR	99.70%	91.1	30.7	69.6	1,548.77
ELF3-HFC elf4-2 rep 2	| Symbols: ELF4-L4 | ELF4-like 4 | chr1:5997932-5998276 FORWARD LENGTH=114	AT1G17455.1,AT1G17455.2	TAIR10_20101214.fasta	12,663.30	100.00%	2	2	4	0.02%	36.00%	SVDASSEGESSGTLK	99.70%	61	28.1	58.6	1,453.67
ELF3-HFC elf4-2 rep 2	| Symbols: PCL1, LUX | Homeodomain-like superfamily protein | chr3:17183248-17184219 FORWARD LENGTH=323	AT3G46640.1,AT3G46640.2	TAIR10_20101214.fasta	34,648.50	100.00%	2	2	7	0.04%	22.90%	ENVASHLQK	99.70%	36.6	27.8	21.3	1,025.54
ELF3-HFC elf4-2 rep 2	| Symbols: PCL1, LUX | Homeodomain-like superfamily protein | chr3:17183248-17184219 FORWARD LENGTH=323	AT3G46640.1,AT3G46640.2	TAIR10_20101214.fasta	34,648.50	100.00%	2	2	7	0.04%	22.90%	FVDVVAHLGIK	99.70%	32.7	25	12.5	1,197.70
ELF3-HFC elf4-2 rep 2	| Symbols: PCL1, LUX | Homeodomain-like superfamily protein | chr3:17183248-17184219 FORWARD LENGTH=323	AT3G46640.1,AT3G46640.2	TAIR10_20101214.fasta	34,648.50	100.00%	2	2	7	0.04%	22.90%	GGSSGPNTSSSNNNVEEEDR	99.70%	99.5	25	94.6	2,036.84
ELF3-HFC elf4-2 rep 2	| Symbols: PCL1, LUX | Homeodomain-like superfamily protein | chr3:17183248-17184219 FORWARD LENGTH=323	AT3G46640.1,AT3G46640.2	TAIR10_20101214.fasta	34,648.50	100.00%	2	2	7	0.04%	22.90%	GGSSGPNTSSSNNNVEEEDRVGSSSPGSDSK	99.70%	68.1	26	65	3,025.28

ELF3-HFC elf4-2 rep 2	| Symbols: PCL1, LUX | Homeodomain-like superfamily protein | chr3:17183248-17184219 FORWARD LENGTH=323	AT3G46640.1,AT3G46640.2	TAIR10_20101214.fasta	34,648.50	100.00%	2	2	7	0.04%	22.90%	LVWTPQLHK	96.60%	24.4	25.4	15.9	1,121.65
ELF3-HFC elf4-2 rep 2	| Symbols: PCL1, LUX | Homeodomain-like superfamily protein | chr3:17183248-17184219 FORWARD LENGTH=323	AT3G46640.1,AT3G46640.2	TAIR10_20101214.fasta	34,648.50	100.00%	2	2	7	0.04%	22.90%	LVWTPQLHKR	95.00%	16.8	25	12.7	1,277.75
ELF3-HFC elf4-2 rep 2	| Symbols: PCL1, LUX | Homeodomain-like superfamily protein | chr3:17183248-17184219 FORWARD LENGTH=323	AT3G46640.1,AT3G46640.2	TAIR10_20101214.fasta	34,648.50	100.00%	2	2	7	0.04%	22.90%	TIMQLMNVEGLTR	99.50%	53.5	30.6	15.2	1,506.77
ELF3-HFC elf4-2 rep 2	| Symbols: PHYB, HY3, OOP1 | phytochrome B | chr2:8140079-8144151 FORWARD LENGTH=1172	AT2G18790.1	TAIR10_20101214.fasta	129,333.90	100.00%	29	44	63	0.38%	41.00%	AIQQYTVDAR	99.70%	67.8	29.9	55.6	1,164.60
ELF3-HFC elf4-2 rep 2	| Symbols: PHYB, HY3, OOP1 | phytochrome B | chr2:8140079-8144151 FORWARD LENGTH=1172	AT2G18790.1	TAIR10_20101214.fasta	129,333.90	100.00%	29	44	63	0.38%	41.00%	AISQLQALPGGDIK	99.70%	93.6	26.1	83.7	1,410.80
ELF3-HFC elf4-2 rep 2	| Symbols: PHYB, HY3, OOP1 | phytochrome B | chr2:8140079-8144151 FORWARD LENGTH=1172	AT2G18790.1	TAIR10_20101214.fasta	129,333.90	100.00%	29	44	63	0.38%	41.00%	ALRGDEEKNVEVK	99.70%	46.9	29.8	27.5	1,486.79
ELF3-HFC elf4-2 rep 2	| Symbols: PHYB, HY3, OOP1 | phytochrome B | chr2:8140079-8144151 FORWARD LENGTH=1172	AT2G18790.1	TAIR10_20101214.fasta	129,333.90	100.00%	29	44	63	0.38%	41.00%	AVFVVVNACSSK	99.70%	84.7	29.6	72.8	1,280.67
ELF3-HFC elf4-2 rep 2	| Symbols: PHYB, HY3, OOP1 | phytochrome B | chr2:8140079-8144151 FORWARD LENGTH=1172	AT2G18790.1	TAIR10_20101214.fasta	129,333.90	100.00%	29	44	63	0.38%	41.00%	CDGAAFLYHGK	99.70%	57.8	27.6	42.2	1,238.56
ELF3-HFC elf4-2 rep 2	| Symbols: PHYB, HY3, OOP1 | phytochrome B | chr2:8140079-8144151 FORWARD LENGTH=1172	AT2G18790.1	TAIR10_20101214.fasta	129,333.90	100.00%	29	44	63	0.38%	41.00%	DMAGEQGIDELGAVAR	99.70%	97.6	28.7	85.9	1,647.76
ELF3-HFC elf4-2 rep 2	| Symbols: PHYB, HY3, OOP1 | phytochrome B | chr2:8140079-8144151 FORWARD LENGTH=1172	AT2G18790.1	TAIR10_20101214.fasta	129,333.90	100.00%	29	44	63	0.38%	41.00%	DYLNNIVGVCFVGQDVTSQK	99.70%	58.3	31.5	47.5	2,256.10
ELF3-HFC elf4-2 rep 2	| Symbols: PHYB, HY3, OOP1 | phytochrome B | chr2:8140079-8144151 FORWARD LENGTH=1172	AT2G18790.1	TAIR10_20101214.fasta	129,333.90	100.00%	29	44	63	0.38%	41.00%	EITLLNPVWIHSK	99.70%	62.9	26.8	50.4	1,549.87
ELF3-HFC elf4-2 rep 2	| Symbols: PHYB, HY3, OOP1 | phytochrome B | chr2:8140079-8144151 FORWARD LENGTH=1172	AT2G18790.1	TAIR10_20101214.fasta	129,333.90	100.00%	29	44	63	0.38%	41.00%	ELAYICQVIK	99.70%	47.5	28.6	34.3	1,236.67
ELF3-HFC elf4-2 rep 2	| Symbols: PHYB, HY3, OOP1 | phytochrome B | chr2:8140079-8144151 FORWARD LENGTH=1172	AT2G18790.1	TAIR10_20101214.fasta	129,333.90	100.00%	29	44	63	0.38%	41.00%	FANSLLEATDLNEDQK	99.70%	114	31	87.5	1,807.87
ELF3-HFC elf4-2 rep 2	| Symbols: PHYB, HY3, OOP1 | phytochrome B | chr2:8140079-8144151 FORWARD LENGTH=1172	AT2G18790.1	TAIR10_20101214.fasta	129,333.90	100.00%	29	44	63	0.38%	41.00%	FANSLLEATDLNEDQKQLLETSVSCEK	98.10%	30.4	32	23.2	3,082.49
ELF3-HFC elf4-2 rep 2	| Symbols: PHYB, HY3, OOP1 | phytochrome B | chr2:8140079-8144151 FORWARD LENGTH=1172	AT2G18790.1	TAIR10_20101214.fasta	129,333.90	100.00%	29	44	63	0.38%	41.00%	FHEDEHGEVVAESK	99.70%	87.6	27.6	82.4	1,612.72
ELF3-HFC elf4-2 rep 2	| Symbols: PHYB, HY3, OOP1 | phytochrome B | chr2:8140079-8144151 FORWARD LENGTH=1172	AT2G18790.1	TAIR10_20101214.fasta	129,333.90	100.00%	29	44	63	0.38%	41.00%	FINIQGDYK	99.70%	50.5	28	36.4	1,097.56
ELF3-HFC elf4-2 rep 2	| Symbols: PHYB, HY3, OOP1 | phytochrome B | chr2:8140079-8144151 FORWARD LENGTH=1172	AT2G18790.1	TAIR10_20101214.fasta	129,333.90	100.00%	29	44	63	0.38%	41.00%	FVQALLTANK	99.70%	65.6	26.1	39.2	1,104.64
ELF3-HFC elf4-2 rep 2	| Symbols: PHYB, HY3, OOP1 | phytochrome B | chr2:8140079-8144151 FORWARD LENGTH=1172	AT2G18790.1	TAIR10_20101214.fasta	129,333.90	100.00%	29	44	63	0.38%	41.00%	FVQALLTANKR	99.70%	45.6	25	24.4	1,260.74
ELF3-HFC elf4-2 rep 2	| Symbols: PHYB, HY3, OOP1 | phytochrome B | chr2:8140079-8144151 FORWARD LENGTH=1172	AT2G18790.1	TAIR10_20101214.fasta	129,333.90	100.00%	29	44	63	0.38%	41.00%	LHAVFEQSGESGK	99.70%	58.9	29.4	53	1,388.68
ELF3-HFC elf4-2 rep 2	| Symbols: PHYB, HY3, OOP1 | phytochrome B | chr2:8140079-8144151 FORWARD LENGTH=1172	AT2G18790.1	TAIR10_20101214.fasta	129,333.90	100.00%	29	44	63	0.38%	41.00%	LIETATVPIFAVDAGGCINGWNAK	99.70%	62.5	31.3	22.8	2,518.26
ELF3-HFC elf4-2 rep 2	| Symbols: PHYB, HY3, OOP1 | phytochrome B | chr2:8140079-8144151 FORWARD LENGTH=1172	AT2G18790.1	TAIR10_20101214.fasta	129,333.90	100.00%	29	44	63	0.38%	41.00%	LLCDTVVESVR	99.70%	56	30.8	35.9	1,290.67
ELF3-HFC elf4-2 rep 2	| Symbols: PHYB, HY3, OOP1 | phytochrome B | chr2:8140079-8144151 FORWARD LENGTH=1172	AT2G18790.1	TAIR10_20101214.fasta	129,333.90	100.00%	29	44	63	0.38%	41.00%	MIVDCNATPVLVVQDDR	99.70%	49	30.4	46.1	1,944.95
ELF3-HFC elf4-2 rep 2	| Symbols: PHYB, HY3, OOP1 | phytochrome B | chr2:8140079-8144151 FORWARD LENGTH=1172	AT2G18790.1	TAIR10_20101214.fasta	129,333.90	100.00%	29	44	63	0.38%	41.00%	NTGKPFYAILHR	99.70%	53.1	29.4	46.4	1,416.77
ELF3-HFC elf4-2 rep 2	| Symbols: PHYB, HY3, OOP1 | phytochrome B | chr2:8140079-8144151 FORWARD LENGTH=1172	AT2G18790.1	TAIR10_20101214.fasta	129,333.90	100.00%	29	44	63	0.38%	41.00%	QDTECFTK	99.00%	41.1	25	34	1,028.44
ELF3-HFC elf4-2 rep 2	| Symbols: PHYB, HY3, OOP1 | phytochrome B | chr2:8140079-8144151 FORWARD LENGTH=1172	AT2G18790.1	TAIR10_20101214.fasta	129,333.90	100.00%	29	44	63	0.38%	41.00%	QLLETSVSCEK	99.70%	61.1	30.1	45.9	1,293.64
ELF3-HFC elf4-2 rep 2	| Symbols: PHYB, HY3, OOP1 | phytochrome B | chr2:8140079-8144151 FORWARD LENGTH=1172	AT2G18790.1	TAIR10_20101214.fasta	129,333.90	100.00%	29	44	63	0.38%	41.00%	RDDLEPYIGLHYPATDIPQASR	99.70%	53.9	31.4	0	2,527.26
ELF3-HFC elf4-2 rep 2	| Symbols: PHYB, HY3, OOP1 | phytochrome B | chr2:8140079-8144151 FORWARD LENGTH=1172	AT2G18790.1	TAIR10_20101214.fasta	129,333.90	100.00%	29	44	63	0.38%	41.00%	RGGEQAQSSGTK	99.20%	31.2	29.8	18	1,205.59
ELF3-HFC elf4-2 rep 2	| Symbols: PHYB, HY3, OOP1 | phytochrome B | chr2:8140079-8144151 FORWARD LENGTH=1172	AT2G18790.1	TAIR10_20101214.fasta	129,333.90	100.00%	29	44	63	0.38%	41.00%	RQDTECFTK	99.70%	31.7	26.1	3.7	1,184.54
ELF3-HFC elf4-2 rep 2	| Symbols: PHYB, HY3, OOP1 | phytochrome B | chr2:8140079-8144151 FORWARD LENGTH=1172	AT2G18790.1	TAIR10_20101214.fasta	129,333.90	100.00%	29	44	63	0.38%	41.00%	SFDYSQSLK	99.70%	52.2	27	40.3	1,074.51
ELF3-HFC elf4-2 rep 2	| Symbols: PHYB, HY3, OOP1 | phytochrome B | chr2:8140079-8144151 FORWARD LENGTH=1172	AT2G18790.1	TAIR10_20101214.fasta	129,333.90	100.00%	29	44	63	0.38%	41.00%	SIEVFGDQIR	99.70%	65.4	30.3	44.6	1,163.61
ELF3-HFC elf4-2 rep 2	| Symbols: PHYB, HY3, OOP1 | phytochrome B | chr2:8140079-8144151 FORWARD LENGTH=1172	AT2G18790.1	TAIR10_20101214.fasta	129,333.90	100.00%	29	44	63	0.38%	41.00%	SLFTSSSSILLER	99.70%	89.8	28	75.6	1,439.77
ELF3-HFC elf4-2 rep 2	| Symbols: PHYB, HY3, OOP1 | phytochrome B | chr2:8140079-8144151 FORWARD LENGTH=1172	AT2G18790.1	TAIR10_20101214.fasta	129,333.90	100.00%	29	44	63	0.38%	41.00%	SLVSDLIYK	99.70%	54.6	26.1	39	1,037.59
ELF3-HFC elf4-2 rep 2	| Symbols: PHYB, HY3, OOP1 | phytochrome B | chr2:8140079-8144151 FORWARD LENGTH=1172	AT2G18790.1	TAIR10_20101214.fasta	129,333.90	100.00%	29	44	63	0.38%	41.00%	SLVSDLIYKENEATVNK	99.70%	53.8	30	27.5	1,923.01
ELF3-HFC elf4-2 rep 2	| Symbols: PHYB, HY3, OOP1 | phytochrome B | chr2:8140079-8144151 FORWARD LENGTH=1172	AT2G18790.1	TAIR10_20101214.fasta	129,333.90	100.00%	29	44	63	0.38%	41.00%	SSFQAFLEVVK	99.70%	71.7	26.8	54.6	1,254.67
ELF3-HFC elf4-2 rep 2	| Symbols: PHYB, HY3, OOP1 | phytochrome B | chr2:8140079-8144151 FORWARD LENGTH=1172	AT2G18790.1	TAIR10_20101214.fasta	129,333.90	100.00%	29	44	63	0.38%	41.00%	TEDPALSIAGAVQSQK	99.70%	112	29.4	89.8	1,614.83
ELF3-HFC elf4-2 rep 2	| Symbols: PHYB, HY3, OOP1 | phytochrome B | chr2:8140079-8144151 FORWARD LENGTH=1172	AT2G18790.1	TAIR10_20101214.fasta	129,333.90	100.00%	29	44	63	0.38%	41.00%	TFSPELQGK	99.70%	41.7	30.8	35.4	1,006.52
ELF3-HFC elf4-2 rep 2	| Symbols: PHYB, HY3, OOP1 | phytochrome B | chr2:8140079-8144151 FORWARD LENGTH=1172	AT2G18790.1	TAIR10_20101214.fasta	129,333.90	100.00%	29	44	63	0.38%	41.00%	TTTYGSSVPEQQITAYLSR	99.70%	69.6	31	67.9	2,102.04
ELF3-HFC elf4-2 rep 2	| Symbols: PHYB, HY3, OOP1 | phytochrome B | chr2:8140079-8144151 FORWARD LENGTH=1172	AT2G18790.1	TAIR10_20101214.fasta	129,333.90	100.00%	29	44	63	0.38%	41.00%	VIGAFCFLQIPSPELQQALAVQR	99.70%	51.3	28.5	7.24	2,586.38
ELF3-HFC elf4-2 rep 2	| Symbols: PHYB, HY3, OOP1 | phytochrome B | chr2:8140079-8144151 FORWARD LENGTH=1172	AT2G18790.1	TAIR10_20101214.fasta	129,333.90	100.00%	29	44	63	0.38%	41.00%	VVDGVVQPCR	99.70%	43.3	27.2	36.9	1,128.58
ELF3-HFC elf4-2 rep 2	| Symbols: PHYB, HY3, OOP1 | phytochrome B | chr2:8140079-8144151 FORWARD LENGTH=1172	AT2G18790.1	TAIR10_20101214.fasta	129,333.90	100.00%	29	44	63	0.38%	41.00%	WTSPEGLGLSVCR	99.70%	64.8	29.8	48.2	1,461.72
ELF3-HFC elf4-2 rep 2	| Symbols: PHYB, HY3, OOP1 | phytochrome B | chr2:8140079-8144151 FORWARD LENGTH=1172	AT2G18790.1	TAIR10_20101214.fasta	129,333.90	100.00%	29	44	63	0.38%	41.00%	YAPSQEWVEIHLSQLSK	99.70%	76.9	30.7	57.3	2,015.02
ELF3-HFC elf4-2 rep 2	| Symbols: PHYB, HY3, OOP1 | phytochrome B | chr2:8140079-8144151 FORWARD LENGTH=1172	AT2G18790.1	TAIR10_20101214.fasta	129,333.90	100.00%	29	44	63	0.38%	41.00%	YYPLGVAPSEVQIK	99.70%	76.7	29.5	66.4	1,563.84
ELF3-HFC elf4-2 rep 2	| Symbols: PHYC | phytochrome C | chr5:14008049-14011619 FORWARD LENGTH=1111	AT5G35840.1	TAIR10_20101214.fasta	123,724.60	100.00%	23	32	33	0.20%	26.60%	AAEVTGLAVEQAIGKPVSDLVEDDSVETVK	99.70%	67.3	30.3	62	3,069.58
ELF3-HFC elf4-2 rep 2	| Symbols: PHYC | phytochrome C | chr5:14008049-14011619 FORWARD LENGTH=1111	AT5G35840.1	TAIR10_20101214.fasta	123,724.60	100.00%	23	32	33	0.20%	26.60%	AISFLQDLLHSSGLSEDQK	99.70%	54	30.8	49	2,088.06
ELF3-HFC elf4-2 rep 2	| Symbols: PHYC | phytochrome C | chr5:14008049-14011619 FORWARD LENGTH=1111	AT5G35840.1	TAIR10_20101214.fasta	123,724.60	100.00%	23	32	33	0.20%	26.60%	AISFLQDLLHSSGLSEDQKR	99.70%	58.9	30.4	46.1	2,244.16
ELF3-HFC elf4-2 rep 2	| Symbols: PHYC | phytochrome C | chr5:14008049-14011619 FORWARD LENGTH=1111	AT5G35840.1	TAIR10_20101214.fasta	123,724.60	100.00%	23	32	33	0.20%	26.60%	AVDFGEISILNPITLHCR	99.70%	71.3	30	64.8	2,055.07
ELF3-HFC elf4-2 rep 2	| Symbols: PHYC | phytochrome C | chr5:14008049-14011619 FORWARD LENGTH=1111	AT5G35840.1	TAIR10_20101214.fasta	123,724.60	100.00%	23	32	33	0.20%	26.60%	CDGAALYYR	99.70%	58.3	25	49.4	1,088.48
ELF3-HFC elf4-2 rep 2	| Symbols: PHYC | phytochrome C | chr5:14008049-14011619 FORWARD LENGTH=1111	AT5G35840.1	TAIR10_20101214.fasta	123,724.60	100.00%	23	32	33	0.20%	26.60%	DGSFIEALLSANK	99.70%	75.2	29.7	60.8	1,364.71
ELF3-HFC elf4-2 rep 2	| Symbols: PHYC | phytochrome C | chr5:14008049-14011619 FORWARD LENGTH=1111	AT5G35840.1	TAIR10_20101214.fasta	123,724.60	100.00%	23	32	33	0.20%	26.60%	DGSFIEALLSANKR	99.70%	40.2	29.6	30.6	1,520.81
ELF3-HFC elf4-2 rep 2	| Symbols: PHYC | phytochrome C | chr5:14008049-14011619 FORWARD LENGTH=1111	AT5G35840.1	TAIR10_20101214.fasta	123,724.60	100.00%	23	32	33	0.20%	26.60%	DLIDWVLK	99.00%	43.5	28.4	21.3	1,001.57
ELF3-HFC elf4-2 rep 2	| Symbols: PHYC | phytochrome C | chr5:14008049-14011619 FORWARD LENGTH=1111	AT5G35840.1	TAIR10_20101214.fasta	123,724.60	100.00%	23	32	33	0.20%	26.60%	EAESAVLLK	99.70%	48	30.5	25.5	959.5408
ELF3-HFC elf4-2 rep 2	| Symbols: PHYC | phytochrome C | chr5:14008049-14011619 FORWARD LENGTH=1111	AT5G35840.1	TAIR10_20101214.fasta	123,724.60	100.00%	23	32	33	0.20%	26.60%	EALTIGTDVK	99.60%	36.2	29.8	25.8	1,046.57
ELF3-HFC elf4-2 rep 2	| Symbols: PHYC | phytochrome C | chr5:14008049-14011619 FORWARD LENGTH=1111	AT5G35840.1	TAIR10_20101214.fasta	123,724.60	100.00%	23	32	33	0.20%	26.60%	EGLGLHITQK	99.70%	39.8	25.4	24.8	1,095.62
ELF3-HFC elf4-2 rep 2	| Symbols: PHYC | phytochrome C | chr5:14008049-14011619 FORWARD LENGTH=1111	AT5G35840.1	TAIR10_20101214.fasta	123,724.60	100.00%	23	32	33	0.20%	26.60%	EVSELTGYDR	99.70%	57.7	27.9	39.4	1,168.55
ELF3-HFC elf4-2 rep 2	| Symbols: PHYC | phytochrome C | chr5:14008049-14011619 FORWARD LENGTH=1111	AT5G35840.1	TAIR10_20101214.fasta	123,724.60	100.00%	23	32	33	0.20%	26.60%	FHEDGHGEVIAECCR	99.70%	28.5	25	22.1	1,815.75
ELF3-HFC elf4-2 rep 2	| Symbols: PHYC | phytochrome C | chr5:14008049-14011619 FORWARD LENGTH=1111	AT5G35840.1	TAIR10_20101214.fasta	123,724.60	100.00%	23	32	33	0.20%	26.60%	IGFNAVISGQK	99.70%	72.5	28.5	51.5	1,133.63
ELF3-HFC elf4-2 rep 2	| Symbols: PHYC | phytochrome C | chr5:14008049-14011619 FORWARD LENGTH=1111	AT5G35840.1	TAIR10_20101214.fasta	123,724.60	100.00%	23	32	33	0.20%	26.60%	IIHPAPGLPEDLVR	99.70%	42.4	25.9	35.4	1,526.87
ELF3-HFC elf4-2 rep 2	| Symbols: PHYC | phytochrome C | chr5:14008049-14011619 FORWARD LENGTH=1111	AT5G35840.1	TAIR10_20101214.fasta	123,724.60	100.00%	23	32	33	0.20%	26.60%	ILLGEVFTTDDYGCCLK	99.70%	70.9	30.1	70.9	2,003.95
ELF3-HFC elf4-2 rep 2	| Symbols: PHYC | phytochrome C | chr5:14008049-14011619 FORWARD LENGTH=1111	AT5G35840.1	TAIR10_20101214.fasta	123,724.60	100.00%	23	32	33	0.20%	26.60%	LHGNFEESER	99.70%	60.2	25.8	44.7	1,217.55
ELF3-HFC elf4-2 rep 2	| Symbols: PHYC | phytochrome C | chr5:14008049-14011619 FORWARD LENGTH=1111	AT5G35840.1	TAIR10_20101214.fasta	123,724.60	100.00%	23	32	33	0.20%	26.60%	LIDTAAVPIFAVDASGVINGWNSK	99.70%	104	29.7	97.6	2,458.30
ELF3-HFC elf4-2 rep 2	| Symbols: PHYC | phytochrome C | chr5:14008049-14011619 FORWARD LENGTH=1111	AT5G35840.1	TAIR10_20101214.fasta	123,724.60	100.00%	23	32	33	0.20%	26.60%	LQQILSETLLSSIR	99.70%	109	25.3	102	1,600.93
ELF3-HFC elf4-2 rep 2	| Symbols: PHYC | phytochrome C | chr5:14008049-14011619 FORWARD LENGTH=1111	AT5G35840.1	TAIR10_20101214.fasta	123,724.60	100.00%	23	32	33	0.20%	26.60%	SLSQPISLSGSTLR	99.70%	100	29.3	80.1	1,445.80
ELF3-HFC elf4-2 rep 2	| Symbols: PHYC | phytochrome C | chr5:14008049-14011619 FORWARD LENGTH=1111	AT5G35840.1	TAIR10_20101214.fasta	123,724.60	100.00%	23	32	33	0.20%	26.60%	SSSKPFYAILHR	99.70%	48.8	29.1	35.4	1,405.76
ELF3-HFC elf4-2 rep 2	| Symbols: PHYC | phytochrome C | chr5:14008049-14011619 FORWARD LENGTH=1111	AT5G35840.1	TAIR10_20101214.fasta	123,724.60	100.00%	23	32	33	0.20%	26.60%	TVVDVPLVDNR	99.70%	55.3	26.3	49.9	1,226.67
ELF3-HFC elf4-2 rep 2	| Symbols: PHYC | phytochrome C | chr5:14008049-14011619 FORWARD LENGTH=1111	AT5G35840.1	TAIR10_20101214.fasta	123,724.60	100.00%	23	32	33	0.20%	26.60%	YACEFLTQVFGVQINK	99.70%	98.2	30.4	82.1	1,916.96
ELF3-HFC elf4-2 rep 2	| Symbols: PHYD | phytochrome D | chr4:9195602-9199486 REVERSE LENGTH=1164	AT4G16250.1	TAIR10_20101214.fasta	129,270.30	100.00%	1	1	18	0.11%	11.90%	AIQQYTVDAR	99.70%	67.8	29.9	55.6	1,164.60
ELF3-HFC elf4-2 rep 2	| Symbols: PHYD | phytochrome D | chr4:9195602-9199486 REVERSE LENGTH=1164	AT4G16250.1	TAIR10_20101214.fasta	129,270.30	100.00%	1	1	18	0.11%	11.90%	FHEDEHGEVVAESK	99.70%	87.6	27.6	82.4	1,612.72
ELF3-HFC elf4-2 rep 2	| Symbols: PHYD | phytochrome D | chr4:9195602-9199486 REVERSE LENGTH=1164	AT4G16250.1	TAIR10_20101214.fasta	129,270.30	100.00%	1	1	18	0.11%	11.90%	FINIQGDYK	99.70%	50.5	28	36.4	1,097.56
ELF3-HFC elf4-2 rep 2	| Symbols: PHYD | phytochrome D | chr4:9195602-9199486 REVERSE LENGTH=1164	AT4G16250.1	TAIR10_20101214.fasta	129,270.30	100.00%	1	1	18	0.11%	11.90%	LHAVFEQSGESGK	99.70%	58.9	29.4	53	1,388.68
ELF3-HFC elf4-2 rep 2	| Symbols: PHYD | phytochrome D | chr4:9195602-9199486 REVERSE LENGTH=1164	AT4G16250.1	TAIR10_20101214.fasta	129,270.30	100.00%	1	1	18	0.11%	11.90%	LLCDTVVESVR	99.70%	56	30.8	35.9	1,290.67
ELF3-HFC elf4-2 rep 2	| Symbols: PHYD | phytochrome D | chr4:9195602-9199486 REVERSE LENGTH=1164	AT4G16250.1	TAIR10_20101214.fasta	129,270.30	100.00%	1	1	18	0.11%	11.90%	NDLEPYIGLHYPATDIPQASR	98.80%	29	31.2	0.65	2,371.16
ELF3-HFC elf4-2 rep 2	| Symbols: PHYD | phytochrome D | chr4:9195602-9199486 REVERSE LENGTH=1164	AT4G16250.1	TAIR10_20101214.fasta	129,270.30	100.00%	1	1	18	0.11%	11.90%	QLLETSVSCEK	99.70%	61.1	30.1	45.9	1,293.64
ELF3-HFC elf4-2 rep 2	| Symbols: PHYD | phytochrome D | chr4:9195602-9199486 REVERSE LENGTH=1164	AT4G16250.1	TAIR10_20101214.fasta	129,270.30	100.00%	1	1	18	0.11%	11.90%	RNDLEPYIGLHYPATDIPQASR	99.70%	53.9	31.4	0	2,527.26
ELF3-HFC elf4-2 rep 2	| Symbols: PHYD | phytochrome D | chr4:9195602-9199486 REVERSE LENGTH=1164	AT4G16250.1	TAIR10_20101214.fasta	129,270.30	100.00%	1	1	18	0.11%	11.90%	SFDYSQSLK	99.70%	52.2	27	40.3	1,074.51
ELF3-HFC elf4-2 rep 2	| Symbols: PHYD | phytochrome D | chr4:9195602-9199486 REVERSE LENGTH=1164	AT4G16250.1	TAIR10_20101214.fasta	129,270.30	100.00%	1	1	18	0.11%	11.90%	SSFQTFLEVVK	99.70%	42.9	27.7	32.3	1,284.68
ELF3-HFC elf4-2 rep 2	| Symbols: PHYD | phytochrome D | chr4:9195602-9199486 REVERSE LENGTH=1164	AT4G16250.1	TAIR10_20101214.fasta	129,270.30	100.00%	1	1	18	0.11%	11.90%	TEDPALSIAGAVQSQK	99.70%	112	29.4	89.8	1,614.83
ELF3-HFC elf4-2 rep 2	| Symbols: PHYD | phytochrome D | chr4:9195602-9199486 REVERSE LENGTH=1164	AT4G16250.1	TAIR10_20101214.fasta	129,270.30	100.00%	1	1	18	0.11%	11.90%	WTSPEGLGLSVCR	99.70%	64.8	29.8	48.2	1,461.72
ELF3-HFC elf4-2 rep 2	| Symbols: PHYE | phytochrome E | chr4:10042312-10045948 REVERSE LENGTH=1112	AT4G18130.1	TAIR10_20101214.fasta	122,519.10	100.00%	12	13	13	0.08%	16.20%	AASFTEISLLNPVLVHSR	99.70%	63.7	27	59.2	1,954.08
ELF3-HFC elf4-2 rep 2	| Symbols: PHYE | phytochrome E | chr4:10042312-10045948 REVERSE LENGTH=1112	AT4G18130.1	TAIR10_20101214.fasta	122,519.10	100.00%	12	13	13	0.08%	16.20%	DGWVTPDGLGLK	99.10%	31.2	27.7	13.7	1,257.65
ELF3-HFC elf4-2 rep 2	| Symbols: PHYE | phytochrome E | chr4:10042312-10045948 REVERSE LENGTH=1112	AT4G18130.1	TAIR10_20101214.fasta	122,519.10	100.00%	12	13	13	0.08%	16.20%	ESAQSLNELTYVR	99.70%	71.6	30.5	59.8	1,509.75
ELF3-HFC elf4-2 rep 2	| Symbols: PHYE | phytochrome E | chr4:10042312-10045948 REVERSE LENGTH=1112	AT4G18130.1	TAIR10_20101214.fasta	122,519.10	100.00%	12	13	13	0.08%	16.20%	ESFTSSRPVLSGNGVAR	99.70%	34.8	31.4	28.6	1,763.90
ELF3-HFC elf4-2 rep 2	| Symbols: PHYE | phytochrome E | chr4:10042312-10045948 REVERSE LENGTH=1112	AT4G18130.1	TAIR10_20101214.fasta	122,519.10	100.00%	12	13	13	0.08%	16.20%	ILGLSDNSSDFLGLLSLPSTSHSGEFDK	99.70%	80	32	68.4	2,936.45
ELF3-HFC elf4-2 rep 2	| Symbols: PHYE | phytochrome E | chr4:10042312-10045948 REVERSE LENGTH=1112	AT4G18130.1	TAIR10_20101214.fasta	122,519.10	100.00%	12	13	13	0.08%	16.20%	LLESSEISASQR	99.70%	63.5	30.9	48.8	1,319.68
ELF3-HFC elf4-2 rep 2	| Symbols: PHYE | phytochrome E | chr4:10042312-10045948 REVERSE LENGTH=1112	AT4G18130.1	TAIR10_20101214.fasta	122,519.10	100.00%	12	13	13	0.08%	16.20%	NIVNHAPFPNSWVGISISPGQELSR	99.70%	47.1	31	41.1	2,719.40
ELF3-HFC elf4-2 rep 2	| Symbols: PHYE | phytochrome E | chr4:10042312-10045948 REVERSE LENGTH=1112	AT4G18130.1	TAIR10_20101214.fasta	122,519.10	100.00%	12	13	13	0.08%	16.20%	QFLETSDACEK	99.70%	41.6	25.9	13.4	1,327.58
ELF3-HFC elf4-2 rep 2	| Symbols: PHYE | phytochrome E | chr4:10042312-10045948 REVERSE LENGTH=1112	AT4G18130.1	TAIR10_20101214.fasta	122,519.10	100.00%	12	13	13	0.08%	16.20%	SSFTAFLEVAK	99.70%	76.5	27	67.8	1,199.63
ELF3-HFC elf4-2 rep 2	| Symbols: PHYE | phytochrome E | chr4:10042312-10045948 REVERSE LENGTH=1112	AT4G18130.1	TAIR10_20101214.fasta	122,519.10	100.00%	12	13	13	0.08%	16.20%	TLFTPSSGASLSK	99.70%	44.4	29.1	26.6	1,295.68
ELF3-HFC elf4-2 rep 2	| Symbols: PHYE | phytochrome E | chr4:10042312-10045948 REVERSE LENGTH=1112	AT4G18130.1	TAIR10_20101214.fasta	122,519.10	100.00%	12	13	13	0.08%	16.20%	TTQKPFYAILHR	99.70%	38.3	28.9	30.6	1,474.82
ELF3-HFC elf4-2 rep 2	| Symbols: PHYE | phytochrome E | chr4:10042312-10045948 REVERSE LENGTH=1112	AT4G18130.1	TAIR10_20101214.fasta	122,519.10	100.00%	12	13	13	0.08%	16.20%	VVQSEELK	98.00%	32.2	31.2	17.3	931.5095
ELF3-HFC elf4-2 rep 2	| Symbols: SPA1 | SPA (suppressor of phyA-105) protein family | chr2:19022572-19026821 REVERSE LENGTH=1029	AT2G46340.1	TAIR10_20101214.fasta	114,689.10	100.00%	7	7	7	0.04%	12.40%	IVQGSNNTNVDSPR	99.70%	70.5	29.1	57.4	1,500.74
ELF3-HFC elf4-2 rep 2	| Symbols: SPA1 | SPA (suppressor of phyA-105) protein family | chr2:19022572-19026821 REVERSE LENGTH=1029	AT2G46340.1	TAIR10_20101214.fasta	114,689.10	100.00%	7	7	7	0.04%	12.40%	NDLESDVDEDLNR	99.70%	62.6	25.7	53.6	1,533.67
ELF3-HFC elf4-2 rep 2	| Symbols: SPA1 | SPA (suppressor of phyA-105) protein family | chr2:19022572-19026821 REVERSE LENGTH=1029	AT2G46340.1	TAIR10_20101214.fasta	114,689.10	100.00%	7	7	7	0.04%	12.40%	NQETPPEFVSDQDLGSK	99.70%	49.8	29.3	44.5	1,890.87
ELF3-HFC elf4-2 rep 2	| Symbols: SPA1 | SPA (suppressor of phyA-105) protein family | chr2:19022572-19026821 REVERSE LENGTH=1029	AT2G46340.1	TAIR10_20101214.fasta	114,689.10	100.00%	7	7	7	0.04%	12.40%	NYLASTDYDGVVQIWDAGTGQGFSQYTEHQK	99.70%	59.7	30.4	55.3	3,478.58
ELF3-HFC elf4-2 rep 2	| Symbols: SPA1 | SPA (suppressor of phyA-105) protein family | chr2:19022572-19026821 REVERSE LENGTH=1029	AT2G46340.1	TAIR10_20101214.fasta	114,689.10	100.00%	7	7	7	0.04%	12.40%	SGDLLNSASVVCSLSFDPDEEHIAAAGISK	99.70%	80.2	31.5	75.1	3,089.47
ELF3-HFC elf4-2 rep 2	| Symbols: SPA1 | SPA (suppressor of phyA-105) protein family | chr2:19022572-19026821 REVERSE LENGTH=1029	AT2G46340.1	TAIR10_20101214.fasta	114,689.10	100.00%	7	7	7	0.04%	12.40%	VAEETVATNNIQLK	99.50%	35.3	29.7	31.9	1,529.82
ELF3-HFC elf4-2 rep 2	| Symbols: SPA1 | SPA (suppressor of phyA-105) protein family | chr2:19022572-19026821 REVERSE LENGTH=1029	AT2G46340.1	TAIR10_20101214.fasta	114,689.10	100.00%	7	7	7	0.04%	12.40%	YSSNVSLVR	99.70%	42	27.9	31.5	1,024.54
ELF3-HFC elf4-2 rep 2	| Symbols: TIC | time for coffee | chr3:7913181-7918879 FORWARD LENGTH=1550	AT3G22380.1,AT3G22380.2	TAIR10_20101214.fasta	164,452.80	100.00%	3	3	5	0.03%	3.48%	ENATQHSETVGEDSPSTADSR	99.70%	40.6	26.1	39.8	2,217.95
ELF3-HFC elf4-2 rep 2	| Symbols: TIC | time for coffee | chr3:7913181-7918879 FORWARD LENGTH=1550	AT3G22380.1,AT3G22380.2	TAIR10_20101214.fasta	164,452.80	100.00%	3	3	5	0.03%	3.48%	QEAAGNDLTEAAK	99.70%	62.1	28.2	56.3	1,317.63
ELF3-HFC elf4-2 rep 2	| Symbols: TIC | time for coffee | chr3:7913181-7918879 FORWARD LENGTH=1550	AT3G22380.1,AT3G22380.2	TAIR10_20101214.fasta	164,452.80	100.00%	3	3	5	0.03%	3.48%	SSGSGEGNSSVLDSIIPLTR	99.70%	71.7	31.4	66.7	1,975.99
ELF3-HFC elf4-2 rep 2	| Symbols: TZP | zinc knuckle (CCHC-type) family protein | chr5:17527261-17530207 FORWARD LENGTH=831	AT5G43630.1	TAIR10_20101214.fasta	90,615.60	100.00%	15	17	18	0.11%	23.80%	AVFDAVQVLR	99.70%	60.9	28.7	48.2	1,117.64
ELF3-HFC elf4-2 rep 2	| Symbols: TZP | zinc knuckle (CCHC-type) family protein | chr5:17527261-17530207 FORWARD LENGTH=831	AT5G43630.1	TAIR10_20101214.fasta	90,615.60	100.00%	15	17	18	0.11%	23.80%	AWWQSAGK	99.00%	44.2	29.1	30.6	933.4577
ELF3-HFC elf4-2 rep 2	| Symbols: TZP | zinc knuckle (CCHC-type) family protein | chr5:17527261-17530207 FORWARD LENGTH=831	AT5G43630.1	TAIR10_20101214.fasta	90,615.60	100.00%	15	17	18	0.11%	23.80%	DCLEVTDTELR	99.70%	58.7	28.4	39.1	1,350.62
ELF3-HFC elf4-2 rep 2	| Symbols: TZP | zinc knuckle (CCHC-type) family protein | chr5:17527261-17530207 FORWARD LENGTH=831	AT5G43630.1	TAIR10_20101214.fasta	90,615.60	100.00%	15	17	18	0.11%	23.80%	DLVQNISVR	99.70%	59.7	29	49.2	1,043.58
ELF3-HFC elf4-2 rep 2	| Symbols: TZP | zinc knuckle (CCHC-type) family protein | chr5:17527261-17530207 FORWARD LENGTH=831	AT5G43630.1	TAIR10_20101214.fasta	90,615.60	100.00%	15	17	18	0.11%	23.80%	FAAVDAITELVWSPSNGLSLR	97.50%	32.7	29.7	26.5	2,246.18
ELF3-HFC elf4-2 rep 2	| Symbols: TZP | zinc knuckle (CCHC-type) family protein | chr5:17527261-17530207 FORWARD LENGTH=831	AT5G43630.1	TAIR10_20101214.fasta	90,615.60	100.00%	15	17	18	0.11%	23.80%	GNEEDNSPFVALTTTSNANGHGQVNAIVDQQQLSPCCVK	99.70%	62.1	31.1	0.02	4,201.90
ELF3-HFC elf4-2 rep 2	| Symbols: TZP | zinc knuckle (CCHC-type) family protein | chr5:17527261-17530207 FORWARD LENGTH=831	AT5G43630.1	TAIR10_20101214.fasta	90,615.60	100.00%	15	17	18	0.11%	23.80%	GVTCLVESQFISNQDFLEEELK	99.70%	47.4	31.6	25.9	2,585.24
ELF3-HFC elf4-2 rep 2	| Symbols: TZP | zinc knuckle (CCHC-type) family protein | chr5:17527261-17530207 FORWARD LENGTH=831	AT5G43630.1	TAIR10_20101214.fasta	90,615.60	100.00%	15	17	18	0.11%	23.80%	KRPGFEEQLIFGSK	99.70%	51	28.3	37	1,635.89
ELF3-HFC elf4-2 rep 2	| Symbols: TZP | zinc knuckle (CCHC-type) family protein | chr5:17527261-17530207 FORWARD LENGTH=831	AT5G43630.1	TAIR10_20101214.fasta	90,615.60	100.00%	15	17	18	0.11%	23.80%	LPISGFTDVPR	99.70%	76.4	29.1	67.5	1,201.66
ELF3-HFC elf4-2 rep 2	| Symbols: TZP | zinc knuckle (CCHC-type) family protein | chr5:17527261-17530207 FORWARD LENGTH=831	AT5G43630.1	TAIR10_20101214.fasta	90,615.60	100.00%	15	17	18	0.11%	23.80%	RPGFEEQLIFGSK	99.70%	45.7	30.1	31.2	1,507.79
ELF3-HFC elf4-2 rep 2	| Symbols: TZP | zinc knuckle (CCHC-type) family protein | chr5:17527261-17530207 FORWARD LENGTH=831	AT5G43630.1	TAIR10_20101214.fasta	90,615.60	100.00%	15	17	18	0.11%	23.80%	SVSGLEGFFLR	99.70%	62.3	27.8	41.5	1,211.64
ELF3-HFC elf4-2 rep 2	| Symbols: TZP | zinc knuckle (CCHC-type) family protein | chr5:17527261-17530207 FORWARD LENGTH=831	AT5G43630.1	TAIR10_20101214.fasta	90,615.60	100.00%	15	17	18	0.11%	23.80%	TLNQECLGSTSK	99.70%	71.8	29.8	60.2	1,337.64
ELF3-HFC elf4-2 rep 2	| Symbols: TZP | zinc knuckle (CCHC-type) family protein | chr5:17527261-17530207 FORWARD LENGTH=831	AT5G43630.1	TAIR10_20101214.fasta	90,615.60	100.00%	15	17	18	0.11%	23.80%	VSPCLTNR	99.00%	35.9	28.9	22.4	946.4775
ELF3-HFC elf4-2 rep 2	| Symbols: TZP | zinc knuckle (CCHC-type) family protein | chr5:17527261-17530207 FORWARD LENGTH=831	AT5G43630.1	TAIR10_20101214.fasta	90,615.60	100.00%	15	17	18	0.11%	23.80%	VVFNQTSK	99.00%	38.1	28.5	22.1	922.4992
ELF3-HFC elf4-2 rep 2	| Symbols: TZP | zinc knuckle (CCHC-type) family protein | chr5:17527261-17530207 FORWARD LENGTH=831	AT5G43630.1	TAIR10_20101214.fasta	90,615.60	100.00%	15	17	18	0.11%	23.80%	WEEGLGGTGYYVAR	99.70%	97.6	29.1	94.1	1,557.73
ELF3-HFC phyb9 rep 1	| Symbols: ELF3, PYK20 | hydroxyproline-rich glycoprotein family protein | chr2:11059459-11063178 FORWARD LENGTH=695	AT2G25930.1	TAIR10_20101214.fasta	77,204.80	100.00%	18	26	30	0.15%	34.20%	EEEDFAVPVYINSR	99.70%	56.8	30.2	43.5	1,667.79
ELF3-HFC phyb9 rep 1	| Symbols: ELF3, PYK20 | hydroxyproline-rich glycoprotein family protein | chr2:11059459-11063178 FORWARD LENGTH=695	AT2G25930.1	TAIR10_20101214.fasta	77,204.80	100.00%	18	26	30	0.15%	34.20%	EKHTPMVAPSSHHSIR	99.70%	53.8	30.5	48.8	1,829.91
ELF3-HFC phyb9 rep 1	| Symbols: ELF3, PYK20 | hydroxyproline-rich glycoprotein family protein | chr2:11059459-11063178 FORWARD LENGTH=695	AT2G25930.1	TAIR10_20101214.fasta	77,204.80	100.00%	18	26	30	0.15%	34.20%	FQEVNQTGSK	99.70%	58.8	29.4	40.5	1,137.55
ELF3-HFC phyb9 rep 1	| Symbols: ELF3, PYK20 | hydroxyproline-rich glycoprotein family protein | chr2:11059459-11063178 FORWARD LENGTH=695	AT2G25930.1	TAIR10_20101214.fasta	77,204.80	100.00%	18	26	30	0.15%	34.20%	HTPMVAPSSHHSIR	99.70%	43.4	29.4	34.8	1,572.77
ELF3-HFC phyb9 rep 1	| Symbols: ELF3, PYK20 | hydroxyproline-rich glycoprotein family protein | chr2:11059459-11063178 FORWARD LENGTH=695	AT2G25930.1	TAIR10_20101214.fasta	77,204.80	100.00%	18	26	30	0.15%	34.20%	IFQSIQEER	99.70%	58.7	29.9	24.6	1,149.59
ELF3-HFC phyb9 rep 1	| Symbols: ELF3, PYK20 | hydroxyproline-rich glycoprotein family protein | chr2:11059459-11063178 FORWARD LENGTH=695	AT2G25930.1	TAIR10_20101214.fasta	77,204.80	100.00%	18	26	30	0.15%	34.20%	ILEPMFPR	99.00%	36.5	30.3	18.4	1,002.54
ELF3-HFC phyb9 rep 1	| Symbols: ELF3, PYK20 | hydroxyproline-rich glycoprotein family protein | chr2:11059459-11063178 FORWARD LENGTH=695	AT2G25930.1	TAIR10_20101214.fasta	77,204.80	100.00%	18	26	30	0.15%	34.20%	LHVNDADKGGPR	99.70%	37.2	30.3	37.2	1,278.66
ELF3-HFC phyb9 rep 1	| Symbols: ELF3, PYK20 | hydroxyproline-rich glycoprotein family protein | chr2:11059459-11063178 FORWARD LENGTH=695	AT2G25930.1	TAIR10_20101214.fasta	77,204.80	100.00%	18	26	30	0.15%	34.20%	LIAASPDLLLDEISFLGK	99.70%	90.7	25.4	80.7	1,915.08
ELF3-HFC phyb9 rep 1	| Symbols: ELF3, PYK20 | hydroxyproline-rich glycoprotein family protein | chr2:11059459-11063178 FORWARD LENGTH=695	AT2G25930.1	TAIR10_20101214.fasta	77,204.80	100.00%	18	26	30	0.15%	34.20%	MALYEQLSIPSQR	99.70%	55.8	31.3	49.6	1,535.79
ELF3-HFC phyb9 rep 1	| Symbols: ELF3, PYK20 | hydroxyproline-rich glycoprotein family protein | chr2:11059459-11063178 FORWARD LENGTH=695	AT2G25930.1	TAIR10_20101214.fasta	77,204.80	100.00%	18	26	30	0.15%	34.20%	MESSAENVVGR	99.70%	94.2	27.5	82.9	1,194.54
ELF3-HFC phyb9 rep 1	| Symbols: ELF3, PYK20 | hydroxyproline-rich glycoprotein family protein | chr2:11059459-11063178 FORWARD LENGTH=695	AT2G25930.1	TAIR10_20101214.fasta	77,204.80	100.00%	18	26	30	0.15%	34.20%	NLSVQHLDSSAANQATEK	99.70%	106	30.9	102	1,912.94
ELF3-HFC phyb9 rep 1	| Symbols: ELF3, PYK20 | hydroxyproline-rich glycoprotein family protein | chr2:11059459-11063178 FORWARD LENGTH=695	AT2G25930.1	TAIR10_20101214.fasta	77,204.80	100.00%	18	26	30	0.15%	34.20%	QGSTGSSPSGPQGISGSK	99.70%	94.2	29	85.6	1,618.77
ELF3-HFC phyb9 rep 1	| Symbols: ELF3, PYK20 | hydroxyproline-rich glycoprotein family protein | chr2:11059459-11063178 FORWARD LENGTH=695	AT2G25930.1	TAIR10_20101214.fasta	77,204.80	100.00%	18	26	30	0.15%	34.20%	SDNEPAPQQQQQPTK	99.70%	84.3	29.8	74.2	1,695.79
ELF3-HFC phyb9 rep 1	| Symbols: ELF3, PYK20 | hydroxyproline-rich glycoprotein family protein | chr2:11059459-11063178 FORWARD LENGTH=695	AT2G25930.1	TAIR10_20101214.fasta	77,204.80	100.00%	18	26	30	0.15%	34.20%	SFRPFAAVDEDSNINNAPEQTMTTTTTTTR	99.70%	95.4	31.4	0.94	3,317.52
ELF3-HFC phyb9 rep 1	| Symbols: ELF3, PYK20 | hydroxyproline-rich glycoprotein family protein | chr2:11059459-11063178 FORWARD LENGTH=695	AT2G25930.1	TAIR10_20101214.fasta	77,204.80	100.00%	18	26	30	0.15%	34.20%	SNNTSTLVHPGPSSQPCGVER	97.30%	32.1	30.4	32.1	2,224.04
ELF3-HFC phyb9 rep 1	| Symbols: ELF3, PYK20 | hydroxyproline-rich glycoprotein family protein | chr2:11059459-11063178 FORWARD LENGTH=695	AT2G25930.1	TAIR10_20101214.fasta	77,204.80	100.00%	18	26	30	0.15%	34.20%	TDQHKMESSAENVVGR	99.70%	44.5	28.7	35.8	1,803.83
ELF3-HFC phyb9 rep 1	| Symbols: ELF3, PYK20 | hydroxyproline-rich glycoprotein family protein | chr2:11059459-11063178 FORWARD LENGTH=695	AT2G25930.1	TAIR10_20101214.fasta	77,204.80	100.00%	18	26	30	0.15%	34.20%	VFAVQLFELHR	99.70%	65.3	28.3	45.6	1,358.76
ELF3-HFC phyb9 rep 1	| Symbols: ELF3, PYK20 | hydroxyproline-rich glycoprotein family protein | chr2:11059459-11063178 FORWARD LENGTH=695	AT2G25930.1	TAIR10_20101214.fasta	77,204.80	100.00%	18	26	30	0.15%	34.20%	VNDYNASLR	99.70%	65.3	29.2	49.7	1,051.52
ELF3-HFC phyb9 rep 1	| Symbols: ELF4 | Protein of unknown function (DUF1313) | chr2:16734545-16734880 REVERSE LENGTH=111	AT2G40080.1	TAIR10_20101214.fasta	12,375.40	100.00%	5	7	9	0.05%	67.60%	NVAEEAEQGEDPAMWENLDR	99.70%	111	26.7	102	2,302.99
ELF3-HFC phyb9 rep 1	| Symbols: ELF4 | Protein of unknown function (DUF1313) | chr2:16734545-16734880 REVERSE LENGTH=111	AT2G40080.1	TAIR10_20101214.fasta	12,375.40	100.00%	5	7	9	0.05%	67.60%	NVALIQELNGNISK	99.70%	80.7	28.7	20.8	1,513.82
ELF3-HFC phyb9 rep 1	| Symbols: ELF4 | Protein of unknown function (DUF1313) | chr2:16734545-16734880 REVERSE LENGTH=111	AT2G40080.1	TAIR10_20101214.fasta	12,375.40	100.00%	5	7	9	0.05%	67.60%	QVQSVLDR	98.10%	29.8	29.3	0	944.516
ELF3-HFC phyb9 rep 1	| Symbols: ELF4 | Protein of unknown function (DUF1313) | chr2:16734545-16734880 REVERSE LENGTH=111	AT2G40080.1	TAIR10_20101214.fasta	12,375.40	100.00%	5	7	9	0.05%	67.60%	SLIQQVNDNHQSR	99.70%	62.8	30.4	55.8	1,538.77
ELF3-HFC phyb9 rep 1	| Symbols: ELF4 | Protein of unknown function (DUF1313) | chr2:16734545-16734880 REVERSE LENGTH=111	AT2G40080.1	TAIR10_20101214.fasta	12,375.40	100.00%	5	7	9	0.05%	67.60%	VVNMYSDLNTSFSSGFHGGK	99.70%	85.5	29.8	78.8	2,146.99
ELF3-HFC phyb9 rep 1	| Symbols: PCL1, LUX | Homeodomain-like superfamily protein | chr3:17183248-17184219 FORWARD LENGTH=323	AT3G46640.1,AT3G46640.2	TAIR10_20101214.fasta	34,648.50	100.00%	1	1	5	0.03%	15.20%	ENVASHLQK	99.70%	46.7	27.7	23.5	1,025.54
ELF3-HFC phyb9 rep 1	| Symbols: PCL1, LUX | Homeodomain-like superfamily protein | chr3:17183248-17184219 FORWARD LENGTH=323	AT3G46640.1,AT3G46640.2	TAIR10_20101214.fasta	34,648.50	100.00%	1	1	5	0.03%	15.20%	FVDVVAHLGIK	99.70%	48.2	25	44.7	1,197.70
ELF3-HFC phyb9 rep 1	| Symbols: PCL1, LUX | Homeodomain-like superfamily protein | chr3:17183248-17184219 FORWARD LENGTH=323	AT3G46640.1,AT3G46640.2	TAIR10_20101214.fasta	34,648.50	100.00%	1	1	5	0.03%	15.20%	GGSSGPNTSSSNNNVEEEDR	99.70%	82.1	25	72.9	2,036.84
ELF3-HFC phyb9 rep 1	| Symbols: PCL1, LUX | Homeodomain-like superfamily protein | chr3:17183248-17184219 FORWARD LENGTH=323	AT3G46640.1,AT3G46640.2	TAIR10_20101214.fasta	34,648.50	100.00%	1	1	5	0.03%	15.20%	LVWTPQLHK	98.70%	27.3	25.4	19.1	1,121.65
ELF3-HFC phyb9 rep 1	| Symbols: TIC | time for coffee | chr3:7913181-7918879 FORWARD LENGTH=1550	AT3G22380.1,AT3G22380.2	TAIR10_20101214.fasta	164,452.80	100.00%	2	2	2	0.01%	2.32%	SSGSGEGNSSVLDSIIPLTR	99.70%	58.7	31.3	48.8	1,975.99
ELF3-HFC phyb9 rep 1	| Symbols: TIC | time for coffee | chr3:7913181-7918879 FORWARD LENGTH=1550	AT3G22380.1,AT3G22380.2	TAIR10_20101214.fasta	164,452.80	100.00%	2	2	2	0.01%	2.32%	SSPSPAPVSPLVSTWK	99.70%	61.5	28.7	49	1,639.87
ELF3-HFC phyb9 rep 2	| Symbols: | BED zinc finger ;hAT family dimerisation domain | chr3:14321838-14323928 FORWARD LENGTH=696	AT3G42170.1	TAIR10_20101214.fasta	78,815.20	100.00%	6	6	7	0.04%	12.50%	AIAGEDPFVTGIAK	99.70%	68.3	30.2	55	1,388.74
ELF3-HFC phyb9 rep 2	| Symbols: | BED zinc finger ;hAT family dimerisation domain | chr3:14321838-14323928 FORWARD LENGTH=696	AT3G42170.1	TAIR10_20101214.fasta	78,815.20	100.00%	6	6	7	0.04%	12.50%	EVFSCLDTADPDYK	99.70%	54.8	25.9	39.6	1,659.72
ELF3-HFC phyb9 rep 2	| Symbols: | BED zinc finger ;hAT family dimerisation domain | chr3:14321838-14323928 FORWARD LENGTH=696	AT3G42170.1	TAIR10_20101214.fasta	78,815.20	100.00%	6	6	7	0.04%	12.50%	GCNQSFAYSNGNK	99.70%	29.4	25	2.19	1,447.59
ELF3-HFC phyb9 rep 2	| Symbols: | BED zinc finger ;hAT family dimerisation domain | chr3:14321838-14323928 FORWARD LENGTH=696	AT3G42170.1	TAIR10_20101214.fasta	78,815.20	100.00%	6	6	7	0.04%	12.50%	NPGILDGQLVIGNCVAR	99.70%	47	29.6	41.9	1,795.95
ELF3-HFC phyb9 rep 2	| Symbols: | BED zinc finger ;hAT family dimerisation domain | chr3:14321838-14323928 FORWARD LENGTH=696	AT3G42170.1	TAIR10_20101214.fasta	78,815.20	100.00%	6	6	7	0.04%	12.50%	SELDQYLDETLLPR	99.70%	58.2	30.9	50.6	1,691.85
ELF3-HFC phyb9 rep 2	| Symbols: | BED zinc finger ;hAT family dimerisation domain | chr3:14321838-14323928 FORWARD LENGTH=696	AT3G42170.1	TAIR10_20101214.fasta	78,815.20	100.00%	6	6	7	0.04%	12.50%	SQNNASPYVAFNQDK	99.70%	59.2	29	51.2	1,682.78
ELF3-HFC phyb9 rep 2	| Symbols: | Homeodomain-like superfamily protein | chr5:24004047-24004943 FORWARD LENGTH=298	AT5G59570.1	TAIR10_20101214.fasta	32,366.70	99.90%	1	1	5	0.03%	13.10%	ASQITLSSLR	99.70%	78.6	28.3	57.5	1,075.61
ELF3-HFC phyb9 rep 2	| Symbols: | Homeodomain-like superfamily protein | chr5:24004047-24004943 FORWARD LENGTH=298	AT5G59570.1	TAIR10_20101214.fasta	32,366.70	99.90%	1	1	5	0.03%	13.10%	ENVASHLQK	99.70%	37.2	27.8	24.4	1,025.54
ELF3-HFC phyb9 rep 2	| Symbols: | Homeodomain-like superfamily protein | chr5:24004047-24004943 FORWARD LENGTH=298	AT5G59570.1	TAIR10_20101214.fasta	32,366.70	99.90%	1	1	5	0.03%	13.10%	FVDVVAHLGIK	99.70%	62.3	25	51.4	1,197.70
ELF3-HFC phyb9 rep 2	| Symbols: | Homeodomain-like superfamily protein | chr5:24004047-24004943 FORWARD LENGTH=298	AT5G59570.1	TAIR10_20101214.fasta	32,366.70	99.90%	1	1	5	0.03%	13.10%	LVWTPQLHK	99.70%	35.8	25.4	27.6	1,121.65
ELF3-HFC phyb9 rep 2	| Symbols: ATARCA, RACK1A_AT, RACK1A | Transducin/WD40 repeat-like superfamily protein | chr1:6222325-6223901 FORWARD LENGTH=327	AT1G18080.1	TAIR10_20101214.fasta	35,748.00	100.00%	2	2	2	0.01%	8.56%	FSPNTLQPTIVSASWDK	99.70%	37	31.1	30.5	1,890.96
ELF3-HFC phyb9 rep 2	| Symbols: ATARCA, RACK1A_AT, RACK1A | Transducin/WD40 repeat-like superfamily protein | chr1:6222325-6223901 FORWARD LENGTH=327	AT1G18080.1	TAIR10_20101214.fasta	35,748.00	100.00%	2	2	2	0.01%	8.56%	LWDLAAGVSTR	99.70%	46	29.9	38.5	1,188.64

ELF3-HFC phyb9 rep 2	| Symbols: ELF3, PYK20 | hydroxyproline-rich glycoprotein family protein | chr2:11059459-11063178 FORWARD LENGTH=695	AT2G25930.1	TAIR10_20101214.fasta	77,204.80	100.00%	19	28	35	0.21%	38.00%	EEEDFAVPVYINSR	99.70%	63.5	30.2	51	1,667.79
ELF3-HFC phyb9 rep 2	| Symbols: ELF3, PYK20 | hydroxyproline-rich glycoprotein family protein | chr2:11059459-11063178 FORWARD LENGTH=695	AT2G25930.1	TAIR10_20101214.fasta	77,204.80	100.00%	19	28	35	0.21%	38.00%	FQEVNQTGSK	99.70%	67.5	29.4	41.4	1,137.55
ELF3-HFC phyb9 rep 2	| Symbols: ELF3, PYK20 | hydroxyproline-rich glycoprotein family protein | chr2:11059459-11063178 FORWARD LENGTH=695	AT2G25930.1	TAIR10_20101214.fasta	77,204.80	100.00%	19	28	35	0.21%	38.00%	IFQSIQEER	99.70%	57.2	30.1	20.9	1,149.59
ELF3-HFC phyb9 rep 2	| Symbols: ELF3, PYK20 | hydroxyproline-rich glycoprotein family protein | chr2:11059459-11063178 FORWARD LENGTH=695	AT2G25930.1	TAIR10_20101214.fasta	77,204.80	100.00%	19	28	35	0.21%	38.00%	LASENAAR	99.00%	57.8	29.1	33	831.4318
ELF3-HFC phyb9 rep 2	| Symbols: ELF3, PYK20 | hydroxyproline-rich glycoprotein family protein | chr2:11059459-11063178 FORWARD LENGTH=695	AT2G25930.1	TAIR10_20101214.fasta	77,204.80	100.00%	19	28	35	0.21%	38.00%	LHVNDADK	99.00%	36.7	27	26	911.4582
ELF3-HFC phyb9 rep 2	| Symbols: ELF3, PYK20 | hydroxyproline-rich glycoprotein family protein | chr2:11059459-11063178 FORWARD LENGTH=695	AT2G25930.1	TAIR10_20101214.fasta	77,204.80	100.00%	19	28	35	0.21%	38.00%	LHVNDADKGGPR	99.70%	43.6	30.7	33.3	1,278.66
ELF3-HFC phyb9 rep 2	| Symbols: ELF3, PYK20 | hydroxyproline-rich glycoprotein family protein | chr2:11059459-11063178 FORWARD LENGTH=695	AT2G25930.1	TAIR10_20101214.fasta	77,204.80	100.00%	19	28	35	0.21%	38.00%	LIAASPDLLLDEISFLGK	99.70%	85.9	25	83.6	1,915.08
ELF3-HFC phyb9 rep 2	| Symbols: ELF3, PYK20 | hydroxyproline-rich glycoprotein family protein | chr2:11059459-11063178 FORWARD LENGTH=695	AT2G25930.1	TAIR10_20101214.fasta	77,204.80	100.00%	19	28	35	0.21%	38.00%	LKDTDNGAESHLATENHSQEGHGSPEDIDNDR	99.70%	51	27.8	34.6	3,488.52
ELF3-HFC phyb9 rep 2	| Symbols: ELF3, PYK20 | hydroxyproline-rich glycoprotein family protein | chr2:11059459-11063178 FORWARD LENGTH=695	AT2G25930.1	TAIR10_20101214.fasta	77,204.80	100.00%	19	28	35	0.21%	38.00%	LKDTDNGAESHLATENHSQEGHGSPEDIDNDREYSK	99.70%	86.1	28.5	78.6	3,995.75
ELF3-HFC phyb9 rep 2	| Symbols: ELF3, PYK20 | hydroxyproline-rich glycoprotein family protein | chr2:11059459-11063178 FORWARD LENGTH=695	AT2G25930.1	TAIR10_20101214.fasta	77,204.80	100.00%	19	28	35	0.21%	38.00%	MALYEQLSIPSQR	99.50%	32.9	30.7	23.9	1,551.78
ELF3-HFC phyb9 rep 2	| Symbols: ELF3, PYK20 | hydroxyproline-rich glycoprotein family protein | chr2:11059459-11063178 FORWARD LENGTH=695	AT2G25930.1	TAIR10_20101214.fasta	77,204.80	100.00%	19	28	35	0.21%	38.00%	NLSVQHLDSSAANQATEK	99.70%	117	30.9	113	1,912.94
ELF3-HFC phyb9 rep 2	| Symbols: ELF3, PYK20 | hydroxyproline-rich glycoprotein family protein | chr2:11059459-11063178 FORWARD LENGTH=695	AT2G25930.1	TAIR10_20101214.fasta	77,204.80	100.00%	19	28	35	0.21%	38.00%	QGSTGSSPSGPQGISGSK	99.70%	64.6	29.1	56	1,618.77
ELF3-HFC phyb9 rep 2	| Symbols: ELF3, PYK20 | hydroxyproline-rich glycoprotein family protein | chr2:11059459-11063178 FORWARD LENGTH=695	AT2G25930.1	TAIR10_20101214.fasta	77,204.80	100.00%	19	28	35	0.21%	38.00%	QNVCLATCSKPEVR	99.70%	68.9	30.2	55.1	1,661.81
ELF3-HFC phyb9 rep 2	| Symbols: ELF3, PYK20 | hydroxyproline-rich glycoprotein family protein | chr2:11059459-11063178 FORWARD LENGTH=695	AT2G25930.1	TAIR10_20101214.fasta	77,204.80	100.00%	19	28	35	0.21%	38.00%	SDNEPAPQQQQQPTK	99.70%	58.7	29.8	48.7	1,695.79
ELF3-HFC phyb9 rep 2	| Symbols: ELF3, PYK20 | hydroxyproline-rich glycoprotein family protein | chr2:11059459-11063178 FORWARD LENGTH=695	AT2G25930.1	TAIR10_20101214.fasta	77,204.80	100.00%	19	28	35	0.21%	38.00%	SFRPFAAVDEDSNINNAPEQTMTTTTTTTR	99.70%	64.6	31.1	55.3	3,332.53
ELF3-HFC phyb9 rep 2	| Symbols: ELF3, PYK20 | hydroxyproline-rich glycoprotein family protein | chr2:11059459-11063178 FORWARD LENGTH=695	AT2G25930.1	TAIR10_20101214.fasta	77,204.80	100.00%	19	28	35	0.21%	38.00%	SNNTSTLVHPGPSSQPCGVER	99.70%	64.6	30.5	61	2,224.04
ELF3-HFC phyb9 rep 2	| Symbols: ELF3, PYK20 | hydroxyproline-rich glycoprotein family protein | chr2:11059459-11063178 FORWARD LENGTH=695	AT2G25930.1	TAIR10_20101214.fasta	77,204.80	100.00%	19	28	35	0.21%	38.00%	TTVTQTTR	99.00%	50	28	40.8	907.4844
ELF3-HFC phyb9 rep 2	| Symbols: ELF3, PYK20 | hydroxyproline-rich glycoprotein family protein | chr2:11059459-11063178 FORWARD LENGTH=695	AT2G25930.1	TAIR10_20101214.fasta	77,204.80	100.00%	19	28	35	0.21%	38.00%	VFAVQLFELHR	99.70%	39.4	28.7	23.6	1,358.76
ELF3-HFC phyb9 rep 2	| Symbols: ELF3, PYK20 | hydroxyproline-rich glycoprotein family protein | chr2:11059459-11063178 FORWARD LENGTH=695	AT2G25930.1	TAIR10_20101214.fasta	77,204.80	100.00%	19	28	35	0.21%	38.00%	VNDYNASLR	99.70%	72.5	29.3	56.2	1,051.52
ELF3-HFC phyb9 rep 2	| Symbols: ELF4 | Protein of unknown function (DUF1313) | chr2:16734545-16734880 REVERSE LENGTH=111	AT2G40080.1	TAIR10_20101214.fasta	12,375.40	100.00%	4	8	12	0.07%	60.40%	NVAEEAEQGEDPAMWENLDR	99.70%	70.6	25.7	64.5	2,318.98
ELF3-HFC phyb9 rep 2	| Symbols: ELF4 | Protein of unknown function (DUF1313) | chr2:16734545-16734880 REVERSE LENGTH=111	AT2G40080.1	TAIR10_20101214.fasta	12,375.40	100.00%	4	8	12	0.07%	60.40%	NVALIQELNGNISK	99.70%	105	28.6	32	1,513.82
ELF3-HFC phyb9 rep 2	| Symbols: ELF4 | Protein of unknown function (DUF1313) | chr2:16734545-16734880 REVERSE LENGTH=111	AT2G40080.1	TAIR10_20101214.fasta	12,375.40	100.00%	4	8	12	0.07%	60.40%	SLIQQVNDNHQSR	99.70%	55.3	30.4	47.4	1,538.77
ELF3-HFC phyb9 rep 2	| Symbols: ELF4 | Protein of unknown function (DUF1313) | chr2:16734545-16734880 REVERSE LENGTH=111	AT2G40080.1	TAIR10_20101214.fasta	12,375.40	100.00%	4	8	12	0.07%	60.40%	VVNMYSDLNTSFSSGFHGGK	99.70%	56.3	29.4	48.1	2,162.98
ELF3-HFC phyb9 rep 2	| Symbols: PCL1, LUX | Homeodomain-like superfamily protein | chr3:17183248-17184219 FORWARD LENGTH=323	AT3G46640.1,AT3G46640.2	TAIR10_20101214.fasta	34,648.50	100.00%	1	2	6	0.04%	18.60%	ENVASHLQK	99.70%	37.2	27.8	24.4	1,025.54
ELF3-HFC phyb9 rep 2	| Symbols: PCL1, LUX | Homeodomain-like superfamily protein | chr3:17183248-17184219 FORWARD LENGTH=323	AT3G46640.1,AT3G46640.2	TAIR10_20101214.fasta	34,648.50	100.00%	1	2	6	0.04%	18.60%	FVDVVAHLGIK	99.70%	62.3	25	51.4	1,197.70
ELF3-HFC phyb9 rep 2	| Symbols: PCL1, LUX | Homeodomain-like superfamily protein | chr3:17183248-17184219 FORWARD LENGTH=323	AT3G46640.1,AT3G46640.2	TAIR10_20101214.fasta	34,648.50	100.00%	1	2	6	0.04%	18.60%	GGSSGPNTSSSNNNVEEEDRVGSSSPGSDSK	99.70%	80	27	0	3,026.27
ELF3-HFC phyb9 rep 2	| Symbols: PCL1, LUX | Homeodomain-like superfamily protein | chr3:17183248-17184219 FORWARD LENGTH=323	AT3G46640.1,AT3G46640.2	TAIR10_20101214.fasta	34,648.50	100.00%	1	2	6	0.04%	18.60%	LVWTPQLHK	99.70%	35.8	25.4	27.6	1,121.65
ELF3-HFC phyb9 rep 2	| Symbols: TIC | time for coffee | chr3:7913181-7918879 FORWARD LENGTH=1550	AT3G22380.1,AT3G22380.2	TAIR10_20101214.fasta	164,452.80	100.00%	4	4	5	0.03%	4.13%	ENATQHSETVGEDSPSTADSR	99.70%	54.3	26.4	43.6	2,217.95
ELF3-HFC phyb9 rep 2	| Symbols: TIC | time for coffee | chr3:7913181-7918879 FORWARD LENGTH=1550	AT3G22380.1,AT3G22380.2	TAIR10_20101214.fasta	164,452.80	100.00%	4	4	5	0.03%	4.13%	NIQSHQQFTK	98.70%	30.9	29	19.1	1,230.62
ELF3-HFC phyb9 rep 2	| Symbols: TIC | time for coffee | chr3:7913181-7918879 FORWARD LENGTH=1550	AT3G22380.1,AT3G22380.2	TAIR10_20101214.fasta	164,452.80	100.00%	4	4	5	0.03%	4.13%	QEAAGNDLTEAAK	99.70%	53.7	28.3	44.9	1,317.63
ELF3-HFC phyb9 rep 2	| Symbols: TIC | time for coffee | chr3:7913181-7918879 FORWARD LENGTH=1550	AT3G22380.1,AT3G22380.2	TAIR10_20101214.fasta	164,452.80	100.00%	4	4	5	0.03%	4.13%	SSGSGEGNSSVLDSIIPLTR	99.70%	65.1	31.3	56.3	1,975.99
ELF3-HFC phyb9 rep 2	| Symbols: TOC1, APRR1, PRR1, AtTOC1 | CCT motif -containing response regulator protein | chr5:24675540-24678176 FORWARD LENGTH=618	AT5G61380.1	TAIR10_20101214.fasta	69,195.40	100.00%	3	3	4	0.02%	7.28%	FQVVASEGINNTK	99.70%	86.3	30.2	64.9	1,406.73
ELF3-HFC phyb9 rep 2	| Symbols: TOC1, APRR1, PRR1, AtTOC1 | CCT motif -containing response regulator protein | chr5:24675540-24678176 FORWARD LENGTH=618	AT5G61380.1	TAIR10_20101214.fasta	69,195.40	100.00%	3	3	4	0.02%	7.28%	GNLSHQENEWSVATAPVHAR	99.70%	41.7	31.1	34	2,203.06
ELF3-HFC phyb9 rep 2	| Symbols: TOC1, APRR1, PRR1, AtTOC1 | CCT motif -containing response regulator protein | chr5:24675540-24678176 FORWARD LENGTH=618	AT5G61380.1	TAIR10_20101214.fasta	69,195.40	100.00%	3	3	4	0.02%	7.28%	NSNPAQFSSAPK	99.70%	42.4	29.5	31.5	1,247.60
GFP-HFC Col rep 1	| Symbols: ELF4 | Protein of unknown function (DUF1313) | chr2:16734545-16734880 REVERSE LENGTH=111	AT2G40080.1	TAIR10_20101214.fasta	12,375.40	100.00%	2	6	7	0.04%	30.60%	NVALIQELNGNISK	99.70%	84.2	27.2	70.7	1,512.84
GFP-HFC Col rep 1	| Symbols: ELF4 | Protein of unknown function (DUF1313) | chr2:16734545-16734880 REVERSE LENGTH=111	AT2G40080.1	TAIR10_20101214.fasta	12,375.40	100.00%	2	6	7	0.04%	30.60%	VVNMYSDLNTSFSSGFHGGK	99.70%	81.3	30.1	81.3	2,146.99
GFP-HFC Col rep 1	| Symbols: PHYE | phytochrome E | chr4:10042312-10045948 REVERSE LENGTH=1112	AT4G18130.1	TAIR10_20101214.fasta	122,519.10	100.00%	2	4	6	0.03%	3.87%	SNTAQYSVDAALFADFAQSIYTGK	99.70%	101	31.9	101	2,568.23
GFP-HFC Col rep 1	| Symbols: PHYE | phytochrome E | chr4:10042312-10045948 REVERSE LENGTH=1112	AT4G18130.1	TAIR10_20101214.fasta	122,519.10	100.00%	2	4	6	0.03%	3.87%	VMVYQFHEDDHGEVVSEIR	99.70%	37.3	30.5	34.5	2,289.06
GFP-HFC Col rep 2	| Symbols: ELF4 | Protein of unknown function (DUF1313) | chr2:16734545-16734880 REVERSE LENGTH=111	AT2G40080.1	TAIR10_20101214.fasta	12,375.40	100.00%	3	8	23	0.11%	48.60%	NVAEEAEQGEDPAMWENLDR	99.70%	77.3	25.5	77.3	2,318.98
GFP-HFC Col rep 2	| Symbols: ELF4 | Protein of unknown function (DUF1313) | chr2:16734545-16734880 REVERSE LENGTH=111	AT2G40080.1	TAIR10_20101214.fasta	12,375.40	100.00%	3	8	23	0.11%	48.60%	NVALIQELNGNISK	99.70%	132	27.2	117	1,512.84
GFP-HFC Col rep 2	| Symbols: ELF4 | Protein of unknown function (DUF1313) | chr2:16734545-16734880 REVERSE LENGTH=111	AT2G40080.1	TAIR10_20101214.fasta	12,375.40	100.00%	3	8	23	0.11%	48.60%	VVNMYSDLNTSFSSGFHGGK	99.70%	99.4	29.4	97.4	2,162.98
GFP-HFC Col rep 3	| Symbols: ELF4 | Protein of unknown function (DUF1313) | chr2:16734545-16734880 REVERSE LENGTH=111	AT2G40080.1	TAIR10_20101214.fasta	12,375.40	100.00%	4	10	28	0.15%	49.50%	NVAEEAEQGEDPAMWENLDR	99.70%	89.1	26.6	89.1	2,302.99
GFP-HFC Col rep 3	| Symbols: ELF4 | Protein of unknown function (DUF1313) | chr2:16734545-16734880 REVERSE LENGTH=111	AT2G40080.1	TAIR10_20101214.fasta	12,375.40	100.00%	4	10	28	0.15%	49.50%	NVALIQELNGNISK	99.70%	107	27.2	96.6	1,512.84
GFP-HFC Col rep 3	| Symbols: ELF4 | Protein of unknown function (DUF1313) | chr2:16734545-16734880 REVERSE LENGTH=111	AT2G40080.1	TAIR10_20101214.fasta	12,375.40	100.00%	4	10	28	0.15%	49.50%	RNVAEEAEQGEDPAMWENLDR	99.10%	36.9	28.6	10.9	2,460.07
GFP-HFC Col rep 3	| Symbols: ELF4 | Protein of unknown function (DUF1313) | chr2:16734545-16734880 REVERSE LENGTH=111	AT2G40080.1	TAIR10_20101214.fasta	12,375.40	100.00%	4	10	28	0.15%	49.50%	VVNMYSDLNTSFSSGFHGGK	99.70%	100	29.7	99.2	2,146.99

Supplementary Figure S1

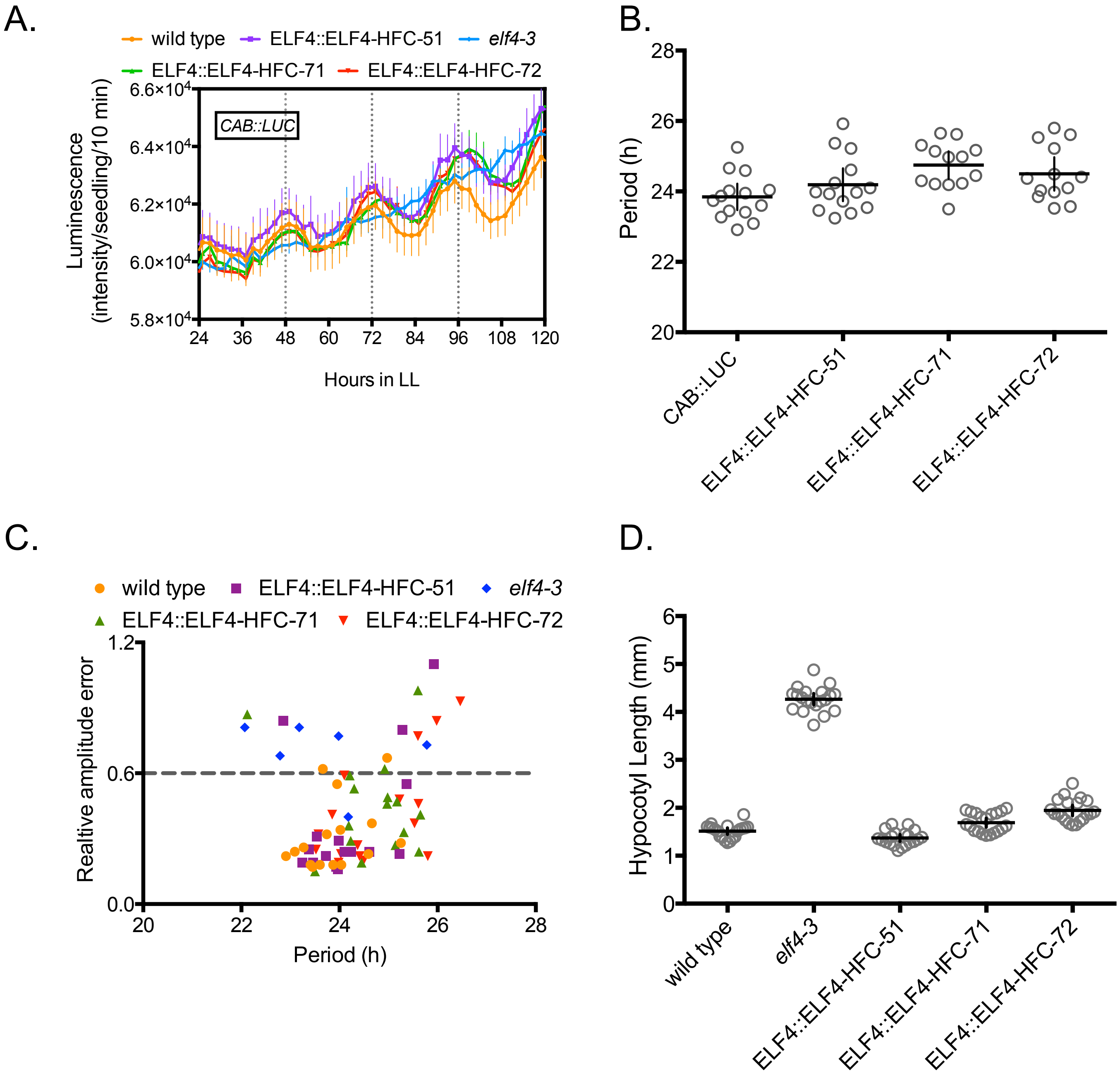

Supplementary Figure S2

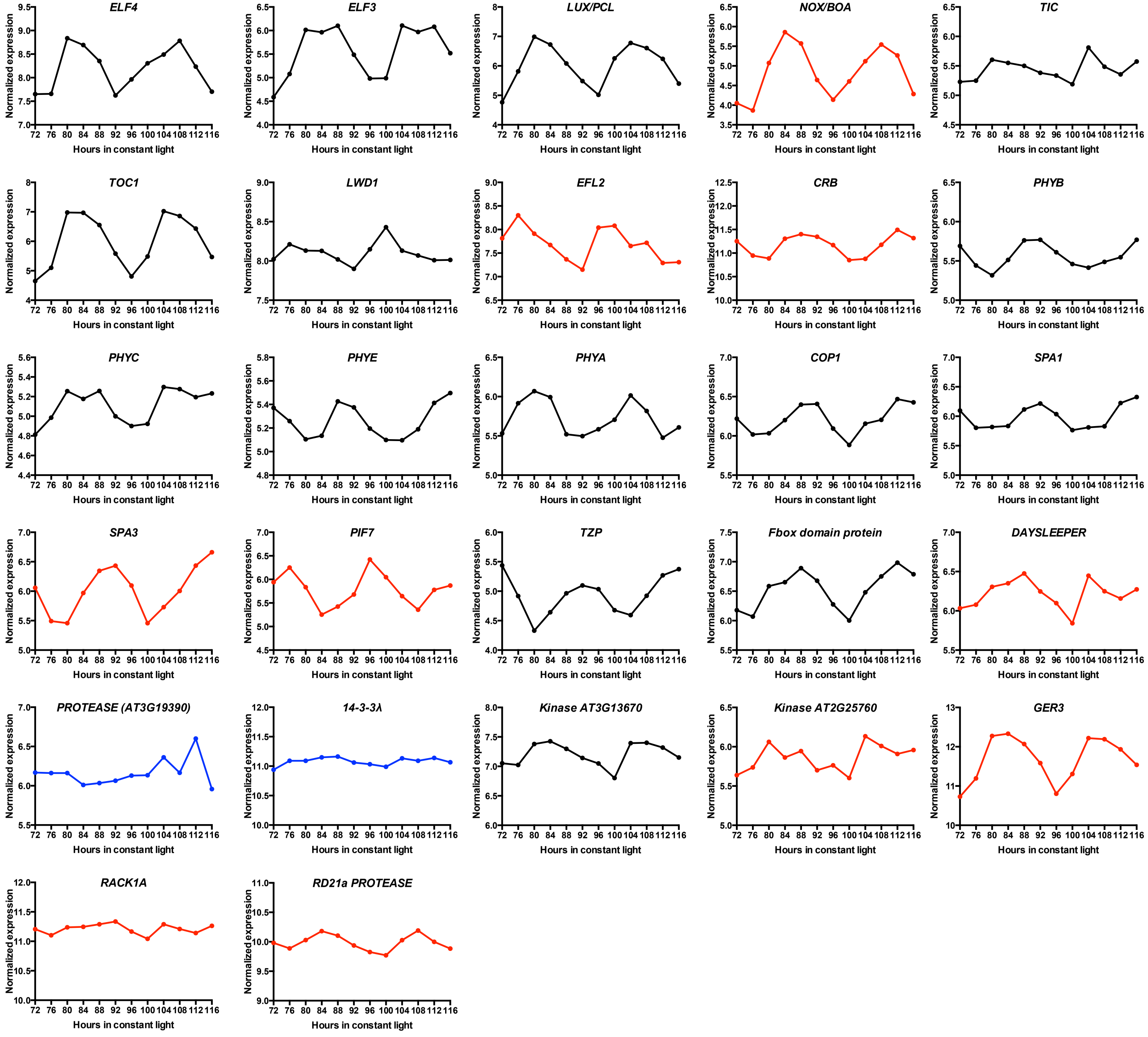

Supplementary Figure S3

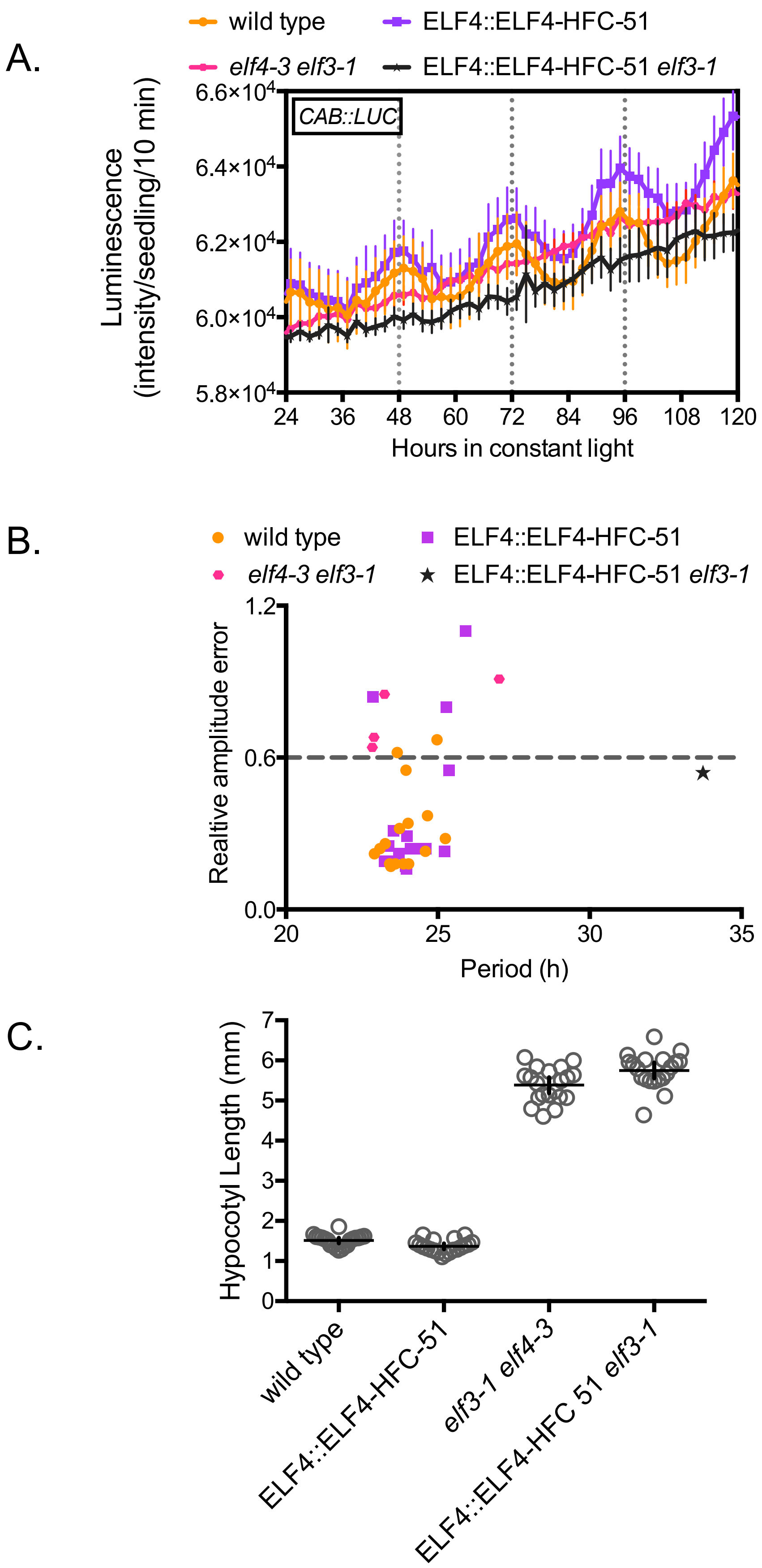

Supplementary Figure S4

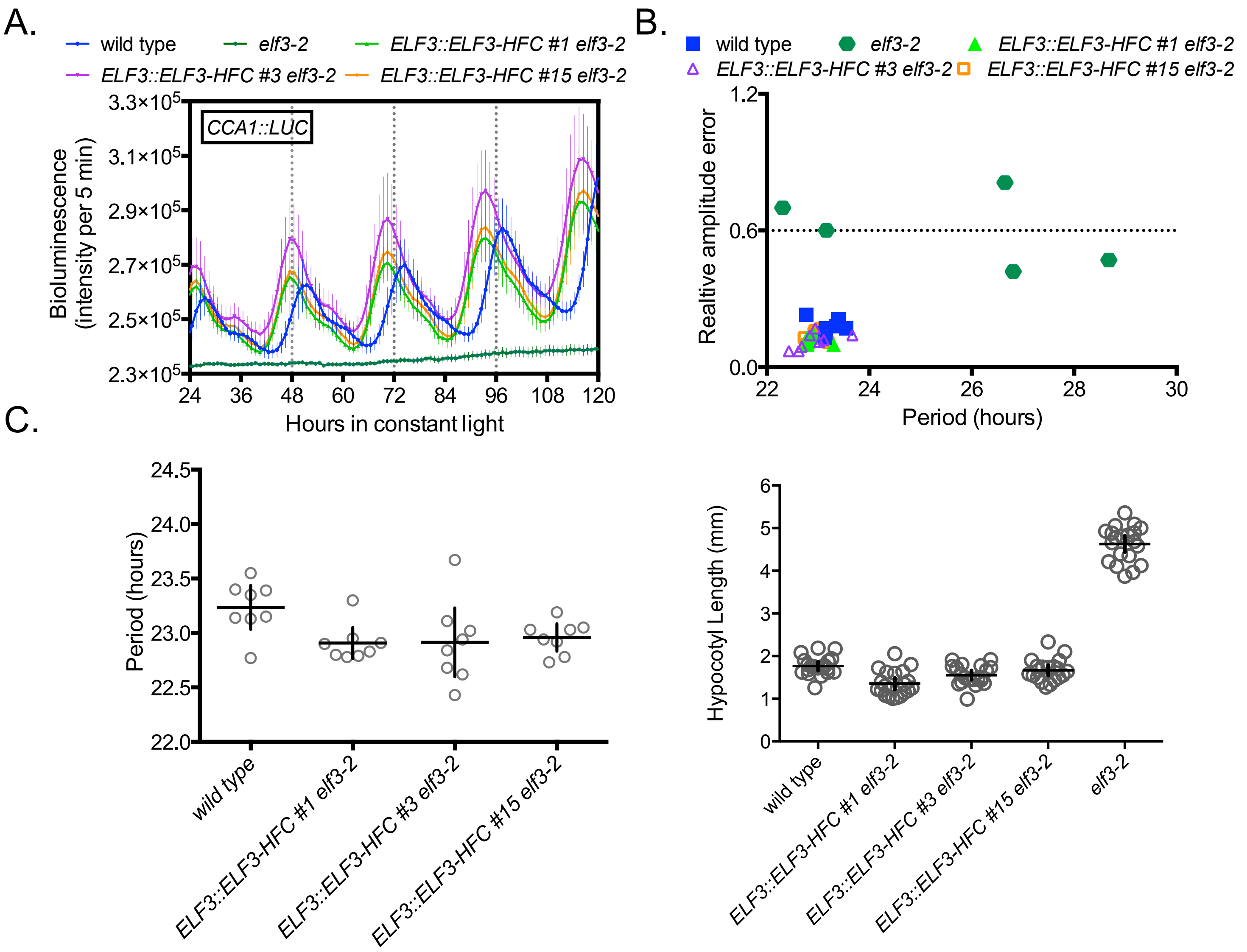

Supplementary Figure S5

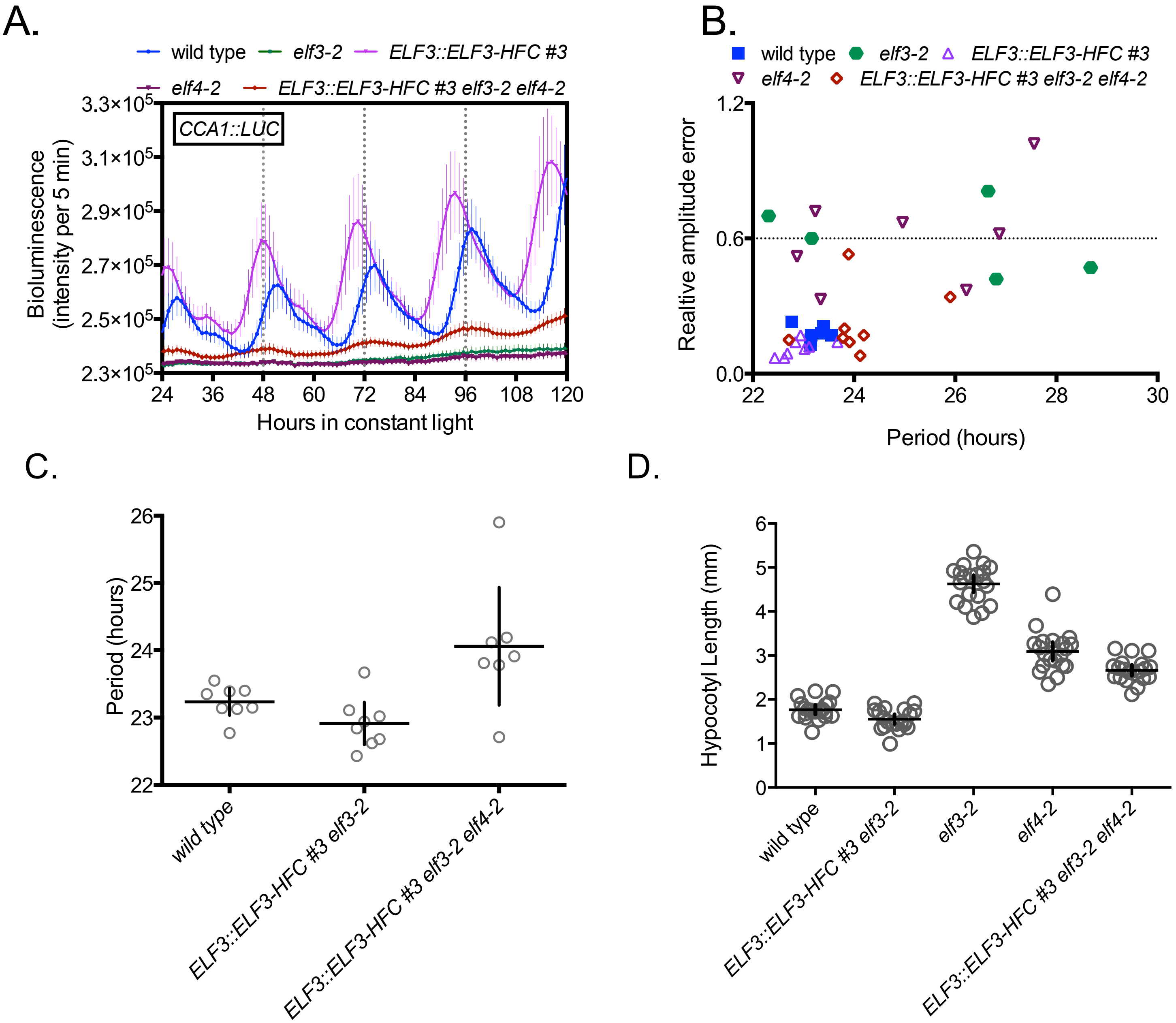

Supplementary Figure S6

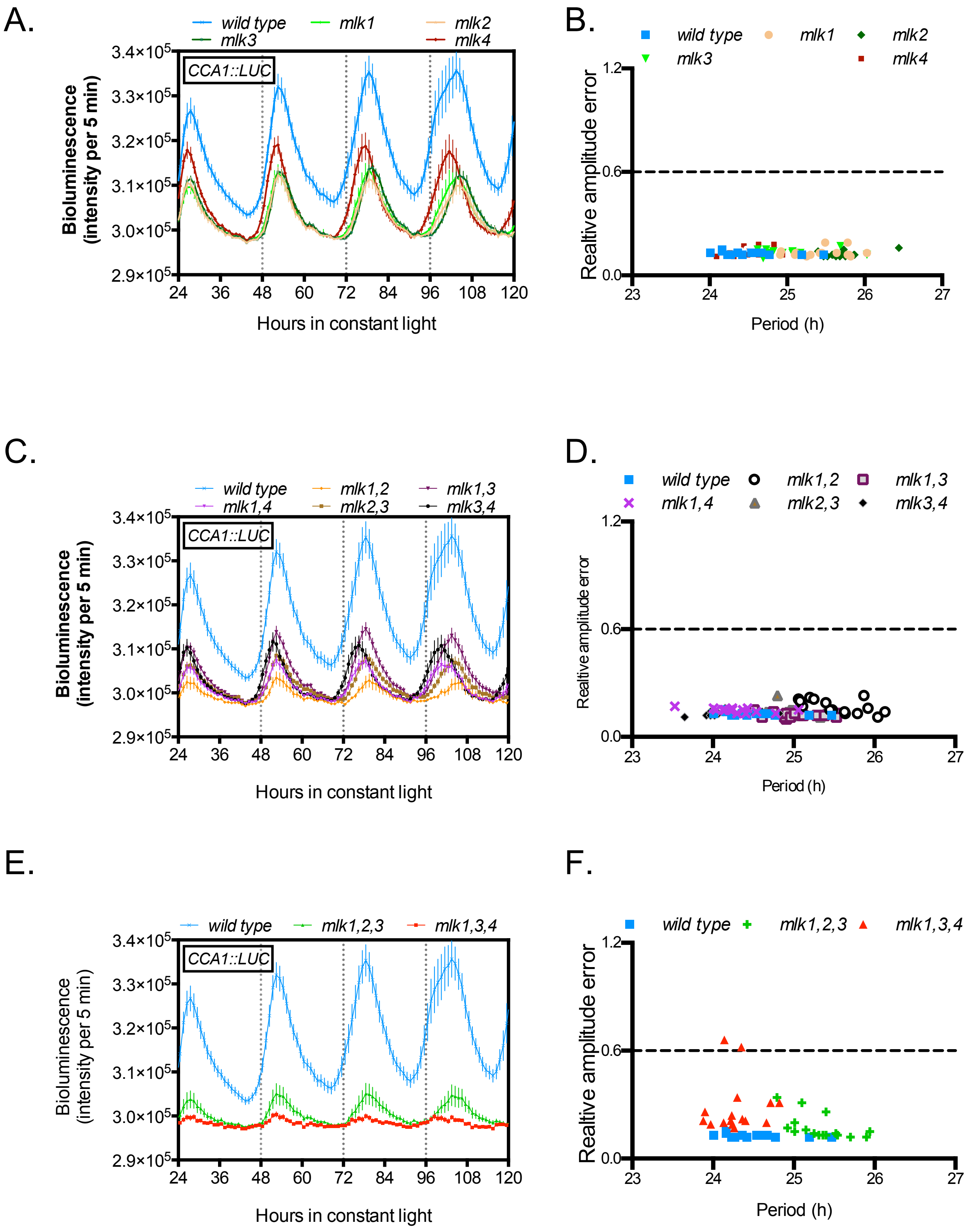

Supplement: Supplemental Data [file 10.1074_M115.054064_mcp.M115.054064-1.pdf]
